# A de novo transcriptional atlas in *Danaus plexippus* reveals variability in dosage compensation across tissues

**DOI:** 10.1038/s42003-021-02335-3

**Published:** 2021-06-25

**Authors:** José M. Ranz, Pablo M. González, Bryan D. Clifton, Nestor O. Nazario-Yepiz, Pablo L. Hernández-Cervantes, María J. Palma-Martínez, Dulce I. Valdivia, Andrés Jiménez-Kaufman, Megan M. Lu, Therese A. Markow, Cei Abreu-Goodger

**Affiliations:** 1 Department of Ecology and Evolutionary Biology, University of California Irvine, Irvine, CA USA; 2 Unidad de Genómica Avanzada (Langebio), CINVESTAV, Irapuato, GTO Mexico; 3 Section of Cell and Developmental Biology, Division of Biological Sciences, University of California San Diego, La Jolla, CA USA; 4 Present Address: Wellcome Trust Sanger Institute, Wellcome Trust Genome Campus, Hinxton, UK; 5 Present Address: Institute of Evolutionary Biology, School of Biological Sciences, University of Edinburgh, Edinburgh, UK

**Keywords:** Gene expression, Molecular evolution

## Abstract

A detailed knowledge of gene function in the monarch butterfly is still lacking. Here we generate a genome assembly from a Mexican nonmigratory population and used RNA-seq data from 14 biological samples for gene annotation and to construct an atlas portraying the breadth of gene expression during most of the monarch life cycle. Two thirds of the genes show expression changes, with long noncoding RNAs being particularly finely regulated during adulthood, and male-biased expression being four times more common than female-biased. The two portions of the monarch heterochromosome *Z*, one ancestral to the Lepidoptera and the other resulting from a chromosomal fusion, display distinct association with sex-biased expression, reflecting sample-dependent incompleteness or absence of dosage compensation in the ancestral but not the novel portion of the *Z*. This study presents extended genomic and transcriptomic resources that will facilitate a better understanding of the monarch’s adaptation to a changing environment.

## Abstract (web summary)

Ranz et al. present a high-quality genome assembly for the monarch butterfly *Danaus plexippus* and a comprehensive set of RNA sequencing data across different tissues and life stages. The authors construct an atlas portraying the breadth of gene expression during the monarch life cycle, which will facilitate a better understanding of *D. plexippus*’ adaptation to a changing environment.

## Introduction

The monarch butterfly *Danaus plexippus* is best known for its spectacular annual migration across North America^1,2^, although its contemporary geographic range also includes different areas of the Northern and Southern hemispheres^3^. Currently, the well-documented population decline of this species is of increasing concern. For example, the census of monarchs across overwintering sites in Mexico during the 2013–2014 period was only ~10% of the average over the last 20 years^4^. This trend is largely explained by a loss of the Mexican overwintering habitat^4^, agricultural practices that reduce the populations of the most suitable *Asclepias* milkweed species for female oviposition^5^ (Supplementary Fig. 1), and their replacement by non-optimal alternative hosts such as the invasive milkweed species *Gomphocarpus physocarpus*^6^.

Central to preserving the future of natural *D. plexippus* populations is an accurate understanding of its unique host specificity and potential for host shifts, the basis for its pesticide and parasite resistance, and other uncommon aspects of this species’ biology compared to other Lepidoptera. An increasing number of studies have attempted to address these questions^7–9^, some developed more within the context of comparative genomics^10,11^. Unfortunately, the monarch research community still relies upon a single *D. plexippus* genome assembly, which was only recently upgraded to reference-quality standards^8,12^. Other reference-quality genome assemblies from different populations are needed in order to have a more reliable delineation of the gene complement of the species, gain insights about the overall organization of structurally dynamic regions of its genome, and categorize minor and major alleles across populations^13–15^. Equally important, and despite relevant efforts to characterize gene function in *D. plexippus*^9,16–19^, a comprehensive expression atlas during the life cycle of this species is still missing. This represents a fundamental gap in knowledge as the larva and pupa stages are key in the context of host adaptation^20^ and interactions with parasites^21^. Furthermore, different sets of genes such as long noncoding RNA genes (lncRNAs) are virtually absent from the existing gene annotation *D. plexippus*^8^, despite being increasingly recognized based on their functional and phenotypic effects^22–24^.

The interplay between chromosome organization and gene expression in *D. plexippus* has also been recently examined through the mechanism of dosage compensation^12^. Lepidoptera (butterflies and moths) predominantly possess a *WZ/ZZ* female-heterogametic system^25^, showing variable diversity in the degree of dosage compensation across species^26–29^, and differing markedly from other female-heterogametic species^30^. In addition, *D. plexippus* possess a heterochromosome *Z* that is a byproduct of a recent fusion between the Lepidoptera ancestral *Z* and an autosome^10^, showing a dual system of incomplete dosage compensation. Specifically, the ancestral portion displays downregulation in males—like other Lepidoptera—while the neo-portion shows upregulation in females, which is reminiscent of the hypertranscription of the single *X* chromosome in *D. melanogaster* males^12^. As some Lepidoptera have shown contrasting patterns for the degree of dosage compensation across tissues^31^, and the observation in *D. plexippus* was made in head samples, its generality remains uncertain.

To fill the above-mentioned gaps in knowledge, we have generated extended genomic and transcriptomic resources that better reflect the underlying genetic diversity of *D. plexippus* while being more accurate about gene function throughout its life cycle. These resources are generated in the context of a non-migratory population of *D. plexippus* in Guanajuato that exhibits patterns of genetic differentiation from migratory populations, including also others from central Mexico, which is indicative of restricted genetic admixture and gene flow^32^. Specifically, we aim at: 1) generating a reference-quality genome assembly, i.e. highly contiguous and complete, and then having this assembly reliably assigned to the *D. plexippus* chromosomal complement; 2) obtaining a gene annotation that leverages on RNA-seq data from 14 different biological conditions representing different life stages and body parts from young adult males and females, and constructing a transcriptome atlas that includes lncRNA genes; 3) providing a comprehensive portrait of the transcriptional program of *D. plexippus* throughout most of its life cycle, paying special attention to larval and pupa stages as well as to differences between the sexes during the adulthood; and 4) examining the degree of dosage compensation across different morphological sections from adult individuals and determining how sex-biased genes in expression are distributed across the two portions of the *Z*. The generated ‘omic resources, and a more comprehensive knowledge on gene and chromosome functionality, will facilitate a broad variety of studies, ultimately helping understand the genetic basis of the monarch’s adaptation to a changing environment as well as the unique aspects of its ecology and behavior.

## Results and discussion

### De novo genome assembly

A single pupa of *D. plexippus* was collected at Irapuato and sequenced using both Illumina PE-150 and PacBio Single Molecule Real-Time (SMRT) technologies under strict conditions to prevent contamination from unintended species (Material and Methods; Supplementary Note 1; Supplementary Fig. 2). A total of ~97.3 Gb of raw Illumina data were generated and filtered resulting into ~72.4 Gb of high-quality trimmed reads, representing a 255x sequence coverage—assuming a genome size of 284 Mb^33^. In parallel, using PacBio, we achieved a 193x sequence coverage (subreads > 1 kb only) and an NR50 (the median read length above which half of the total coverage is contained; Supplementary Fig. 3) of 22.6 kb, a value higher than that associated with recently published reference-quality genome assemblies of *D. melanogaster*^34^. To generate a de novo, reference-quality genome assembly, we adopted different computational strategies that ultimately led to a limited set of genome assemblies, from which one was selected (Supplementary Table 1; Supplementary Fig. 4). This assembly exhibits enhanced contiguity (Supplementary Fig. 5a), encompassing 108 contigs polished at different stages with the Illumina sequencing data, with an additional contig (Sc0000031) very likely representing a different haplotype for Sc0000030 (Supplementary Note 2; Supplementary Fig. 6). In total, 78 of the 108 contigs could be merged into 36 scaffolds (see below). The final assembly, DpMex_v1, features a scaffold N50 of 8.16 Mb (Table 1), and a heterozygosity of 2.15% (Supplementary Fig. 5b). Further, we evaluated the genome assembly and gene set prediction completeness. In the first case, we mapped DNA Illumina sequencing reads from 72 samples back onto the DpMex_v1. The global fraction of mapped sequencing reads was 93.6%, with a further 3.7% mapping discordantly (i.e. paired reads not mapping with the expected orientation and/or separation). Both mapping percentages were very similar to those obtained against the previously generated assembly Dpv3^7^ (Supplementary Table 2). Next, gene-level completeness was ascertained using the nearly-universal set of single-copy genes using BUSCO v4.0.5^35^. We recovered 98% complete BUSCOs in the Lepidoptera gene set (lepidotpera_odb10, *n* = 5,826), with an additional 0.5% in multiple copies (Table 1). Together, these results provide an unphased haploid monarch genome assembly, DpMex_v1, which is highly contiguous and virtually complete.

**Table 1:** Salient features of the *D. plexippus* genome assembly obtained here compared to other relevant ones.

	*D. plexippus*	*D. plexippus*	*D. plexippus* ^a^	*M. cinxia* ^a^	*H. melpomene* ^a^	*B. mori* ^a^
Assembly Identifier	DpMex_v1	Dpv4	Dpv3	v1.0	Hmel2.5	ASM15162v1
Span (Mb)	248.571	248.676	248.564	389.9	275.246	480.5
GC content (%)	32.2	31.6	31.6	32.6	32.8	37.7
Contigs						
Number	108	10,682	15,441	48,180	3,126	88,673
N50 (kb) / NumN50	3,940/21	111.0/548	63.6/906	14.1/7,366	328.9/214	1.6/8,076
Scaffolds						
Number	65	4,115	5,397	8,261	332	43,463
N50 (kb) / NumN50	8,158.1/13	9,209.9/12	716.0/101	119.33/970	14,309.0/9	4,008.4/38
N90 (kb) / NumN90	3,498.3/30	5,644.642/25	160.5/366	29.60/3,396	9,045/19	61.1/258
Longest (Mb)	16.34	15.62	6.2	0.67	18.1	16.2
Ns (%)	0.002	2.73	2.7	7.42	0.4	10.4
CEGMA (*n* = 248) ^b^	C: 93.15% P: 96.8%	C: 92.34% P: 96.8%	C: 90.3% P: 96.8%	C: 68.55% F: 86.7%	C: 88.71 P: 96.77%	C: 76.6% P: 96.8%
BUSCO (*n* = 5286) ^b^	C: 98.5% D: 0.5% F: 0.5%	C: 98.3% D: 1.4% F: 0.5%	C: 98.5% D: 1.6% F: 0.5%	C: 91.8% D: 0.5% F: 3.7%	C: 98.8% D: 0.6% F: 0.3%	C: 95.3% D: 0.3% F: 1.7%

^a^Retrieved from *ensemble.lepbase.org* (http://ensembl.lepbase.org/index.html) as of Sept 1 2019, with the exception of the search for almost-universal orthologs in different assemblies, which was done here with the same sets of query genes.

^b^Using complete gene evidence only. CEGMA: C, complete; P: partial. BUSCO: C, complete (uni and multicopy); D, duplicated or multicopy; F: fragmented. The number of complete unicopy BUSCOs can be calculated as the difference between the total number of complete BUSCOs and the number of multicopy BUSCOs.

### Comparison to other *D. plexippus* genome assemblies

Although the assemblies DpMex_v1, Dpv3^8^, and its derivative Dpv4^12^, have virtually the same span (~249 Mb), all of them are still smaller than the genome size of 284 Mb estimated by flow cytometry^33^. A plausible explanation is that all three assemblies reflect reasonably well the euchromatic but not the heterochromatic genome portion, including most of the exceptionally large heterochromosome *W* in this species^10^, as it requires specialized sequencing approaches due to its repetitive content^36^.

The DpMex_v1 assembly exhibits a highly improved contiguity compared to the Dpv3 assembly as shown by the number of contigs and the contig N50 value (Table 1). Relative to the Dpv4 assembly, which implements Hi-C to scaffold the Dpv3 assembly, the scaffold N50 value for DpMex_v1 is 1 Mb lower (8.16 vs 9.21 Mb) although its longest scaffold, which corresponds to the heterochromosome *Z*, is 727 kb longer (16.34 Mb vs 15.62 Mb). Unlike the assembly Dpv4, DpMex_v1 includes only 23 chromosome-length scaffolds, with the remaining seven chromosomes represented by more than one scaffold (see below). Overall, the high contiguity of both DpMex_v1 and Dpv4 places both assemblies together with that of *H. melpomene* among the few lepidopteran genomes with multi-megabase N50.

At a whole-chromosome scale, we found a high level of collinearity between DpMex_v1 and Dpv4 (Supplementary Fig. 7), which is not reproduced to the same extent at a finer scale due to discrepancies in internal order and orientation of scaffolds, in part due to misassemblies in Dpv3 (Supplementary Data 1; Supplementary Note 3). In addition, we observed differences in the K-mer spectra composition (Supplementary Fig. 8) and a higher gene-level completeness compared to Dpv3 and Dpv4 (Table 1; Supplementary Table 3; Supplementary Data 2; Supplementary Note 3).

### Repeat and gene annotation

We annotated transposable element (TE) insertions and low-complexity repeat sequences in the DpMex_v1 assembly. In total, ~42.9 Mb (17.26%) of the assembly was populated by repeats, with 19.25 Mb (7.75%) corresponding to interspersed repeats (Supplementary Table 4). Subsequently, we generated a new gene annotation (OGS1_DpMex) by considering different types of support: (i) RNA-seq data from 14 different types of biological samples from larval, pupal, and adult stages (see below); (ii) by identifying a homolog in at least one of six other lepidopteran species or *D. melanogaster*; and (iii) by having an equivalent gene model in the previous annotation OGS2^8^. The OGS1_DpMex annotation includes models for 15,995 protein-coding genes (Table 2; Supplementary Fig. 9a), with 82.23% of the gene models being supported by both RNA-seq data and by homologous sequences. Overall, one third of the genome (32.1% or 78.5 Mb) is transcribed into primary transcripts with 10.8% being associated with mature transcripts, and CDS sequences representing 8.9% of the genome.

**Table 2:** Salient features of a previous and our gene annotation of *D. plexippus*.

	OGS2	OGS1_DpMex
*Protein-coding genes* ^a^		
Number	15,130	15,955
Intronless only	1,461	1,096
Average±SD length (kb)	6.00 ± 10.49	4.56 ± 5.02
Min/Max (kb)	0.05/331.24	0.15/87.07
Average±SD encoded protein (aa)	459.94 ± 521.41	454.8 ± 504.19
Supported by RNA-seq	13,960	14,289
Supported by orthology calls	13,730	14,330
Supported by both	13,048	13,152
Supported by only RNA-seq / orthology calls	912/682	1,137/1,178
Supported only by presence in alternative assembly	488	398
Supported only by computational prediction	0	130
*Exons*		
Number	101,578	107,673
Average±SD number per gene	6.71 ± 7.09	6.65 ± 6.79
Average±SD length (kb)	0.21 ± 0.3	0.25 ± 0.4
*Introns*		
Number	86,448	91,678
Average±SD number per gene	5.71 ± 7.09	5.66 ± 6.78
Average±SD length (kb)	0.81 ± 3.47	0.57 ± 0.96
*ncRNAs*	622	1,031
tRNAs	379	689
rRNAs	127	191
miRNAs	116	151
lncRNAs (>200 nt)	na	625
Intersecting repetitive elements	na	227
Intergenic	na	463

^a^In OGS2^8^, only one transcript was annotated per gene. In OGS1_DpMex, this work, alternative splicing is considered, but only the longest transcript was used for multiply spliced genes.

Approximately, 10% of the protein-gene models part of OGS2 were not found in OGS1_DpMex and vice versa. Sequence similarity searches using proteomic data from six other Lepidoptera (Table 2 and Supplementary Table 5) revealed that the proportion of unique models to one of the annotations with homology support is higher for OGS1_DpMex (437 or 40.6% vs 834 or 49.7%). A complementary analysis in which we compared the absolute number of gene models from both assemblies for which it was possible to find at least one homolog with OrthoFinder^37^ also indicated that a larger fraction of the gene models in OGS1_DpMex had significant matches in other species (Supplementary Fig. 10).

Among the gene models found in both OGS2 and OGS1_DpMex, 8,861 correspond to single-copy gene entities while the remaining 2,411 gene entities appear in the form of two or more copies in at least one of the annotations. Among the latter, 664 gene entities show the same number of copies in OGS2 and OGS1_DpMex (1556) while the other 1,747 gene entities differ in copy number between assemblies, with a net difference of 313 (3901—3588) for OGS1_DpMex. Overall, OGS1_DpMex shows an increase in the number of multicopy gene entities, including those that are single copy in OGS2 (2-sample test for equality of proportions, Χ^2^ = 6.164, *d.f.* = 1, *P* = 0.013). These differences can represent an overall more accurate assembly, true differences in copy number between the individuals sequenced, or fragmented predicted models. An extreme example corresponds to a gene model that exhibits significant sequence similarity by BLASTP against the protein-coding gene Cation-Channel complex subunit UNC-79 from the butterfly *Vanessa tameamea*. This gene is single copy in OGS2 whereas it is present in 25 copies in OGS1_DpMex scattered through 16 contigs.

Regarding ncRNA genes, our annotation includes 1656 gene models, i.e. a 2.7-fold increase relative to OGS2 (Table 2). The increase is consistent across all categories of small ncRNA genes, i.e. tRNAs, rRNAs, and miRNAs. Additionally, we used ribodepleted stranded RNA-Seq of pooled samples spanning the larval, pupal, and adult stages to help predict lncRNA genes, which were omitted in OGS2, via a dedicated pipeline. Under conservative criteria (Material and Methods), we annotated 625 lncRNA gene models (Table 2), 463 of them being intergenic, 134 being antisense to coding sequences, and 28 residing in introns of models of protein-coding genes. As in humans and other insects, a sizable number of the lncRNA models (227 or 36.3%) overlap with TE sequences^38,39^. In summary, the gene annotation OGS1_DpMex encompasses 17,651 models, i.e. 1,899 more than OGS2, of which 865 correspond to protein-coding genes and 1,034 to ncRNA genes (Supplementary Fig. 9b).

### Genome assembly assignment to chromosomes

**Fig. 1:**
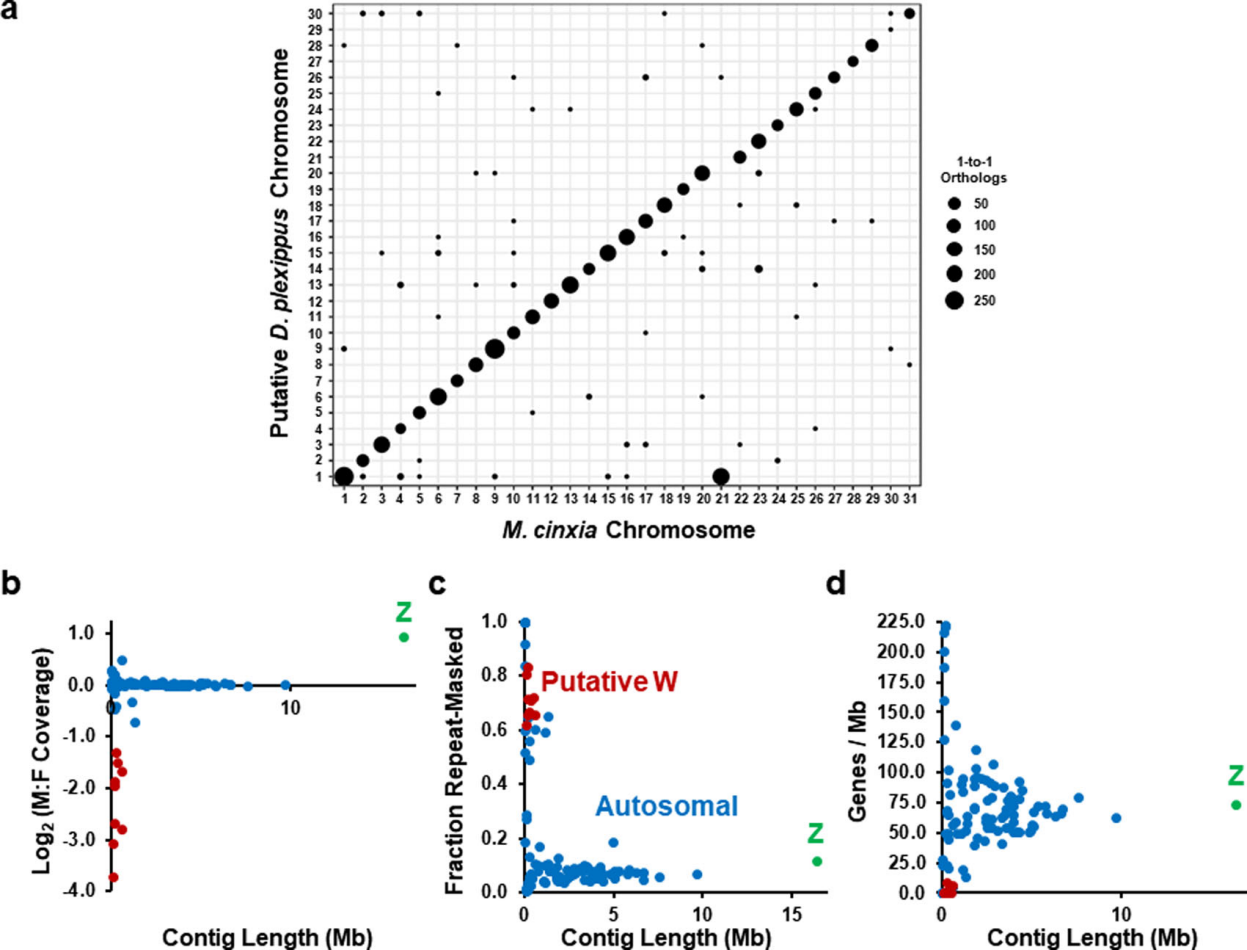
Assignation of the DpMex_v1 genome assembly to the chromosomes of *D. plexippus*. **a** Chromosomal anchoring of contigs based on the chromosome of *M. cinxia* showing the highest number of 1-to-1 orthologs for protein-coding genes in our OGS1_DpMex. Overall, 97.02% (4,855 out of 5,004) orthologs mapped to chromosomes of *M. cinxia* provided coherent cytological information about to which chromosomes the contigs should be anchored. The diameter of the circles denotes the number of such 1-to-1 orthologs. Overall, high synteny conservation between the two species can be observed, which supports that the gene content of the ancestral chromosomal elements to these two species remained essentially intact. It can also be observed that chromosome *1* in *D. plexippus* encompasses genes from chromosomes *1* and *21* of *M. cinxia*, reflecting the outcome of a fusion event. For each of the 108 contigs part of the DpMex_v1 assembly, (**b**) the log_2_ of the male to female coverage, (**c**) the repeat-masked fraction, and (**d**) gene density, is plotted against its length. A color code is used to distinguish the contigs categorized as autosomal, *Z*-related, and potentially *W*-related. The raw data for these plots are provided in Supplementary Data 3.

Contig anchoring to the *D. plexippus* chromosomes was performed assuming a high level of chromosome conservation of gene content, i.e. macrosynteny, which is supported by comparative analysis involving *Melitaea cinxia*, *Heliconious melpomene*, and other Lepidoptera^10,40–43^. Each DpMex_v1 contig was anchored to the *M. cinxia* chromosome that harbored the highest number of 1-to-1 orthologs between protein-coding genes in the OGS1_DpMex and those of *M. cinxia*. This species presumably preserves the ancestral lepidopteran karyotype *n* = 31^44,45^, being phylogenetically close to *D. plexippus*, which has 30 chromosomes^10,11^. The anchoring process was based on positional information from 5,004 1-to-1 orthologs, which resulted in 74 out of 108 contigs being anchored directly to chromosomes (Fig. 1a; Supplementary Fig. 11a; Supplementary Table 6; Supplementary Data 3), and six more indirectly as they are scaffolded with some of the former using RaGOO. Importantly, these 80 contigs span ~238.4 Mb, i.e. 97.2% of the total assembly length, and include 97.02% (i.e. 4855) of the 1-to-1 orthologs mapped. The remaining 149 1-to-1 orthologs could have been involved in interchromosomal gene transposition events.

All but chromosomes *3*, *9*, *13*, *16*, *19*, *21*, and *28* are represented by a single scaffold. Crucially, all contigs of the same scaffold, if mapped, agreed in their chromosomal assignment. The largest chromosome spans 16.34 Mb and includes genes from chromosome *1* and *21* of *M. cinxia*, confirming a previously inferred fusion event that predated the radiation of the genus *Danaus*^10^ (Fig. 1a). Such chromosomes correspond to the ancestral- and the neo-portion of the heterochromosome *Z* of *D. plexippus*, respectively^10,44^.

Further examination of the microsynteny conservation at longer phylogenetic distances (Supplementary Note 4; Supplementary Fig. 12) and the contig assignment to homo- and heterochromosomes by calculating the log_2_ male to female coverage for every contig using genomic DNA sequencing data^7^ as well as other genomic features (Fig. 1b–c; Supplementary Note 4; Supplementary Fig. 11b and 13; Supplementary Data 3-4), strongly supported the reliability of the anchoring process of our genome assembly to the autosomes and the heterochromosome *Z* of *D. plexippus*.

### Transcriptome atlas

**Fig. 2:**
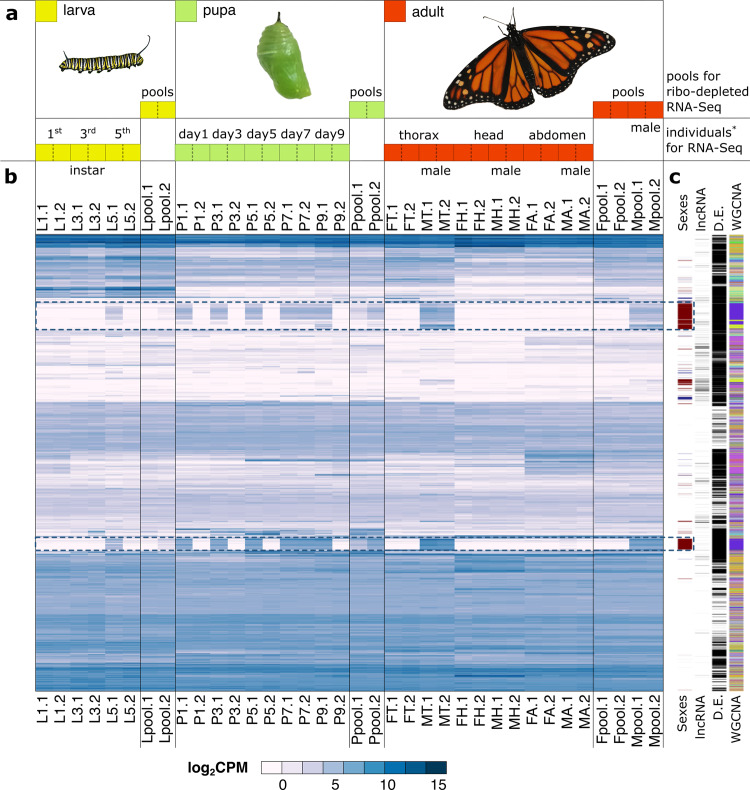
Transcriptome atlas of *D. plexippus*. **a** Fourteen specific stages and anatomical parts were RNA-sequenced. These include: 1st, 3rd, and 5th instar larvae (yellow boxes); day 1, 3, 5, 7, and 9 for pupae (green boxes); and thorax, head, and abdomen from 2-day-old posteclosion males and females (red boxes). Two biological replicates of these 14 sample types were sequenced to an average of 20.7 million PE reads each. In addition, total RNA from four additional sample types corresponding to broadly defined developmental stages (larva, pupa, adult males, and females) were ribodepleted and sequenced to an average of 43.3 million PE reads. These samples consisted of pools of individuals of the same stage, for which two biological replicates were also included. In total, 926 million strand-specific paired-end (PE) reads resulting in 157 Gb of sequence data were obtained. **b** Heatmap of library-size normalized gene-level log_2_CPM of 14,865 expressed genes (rows) across 36 samples sequenced (columns; 18 sample types × 2 replicates each) (Supplementary Data 6-7). The replicate number (.1, .2) is indicated at the end of their names. Dotted boxes highlight two groups of genes with marked male-biased expression (Supplementary Note 5). **c** From more internal to more external, selected gene categories: genes with sex-biased expression (male-biased dark red, female-biased dark blue); lncRNA genes; D.E., differentially expressed genes in at least one contrast; WGCNA, genes clustered according to this methodology.

We sequenced poly(A) + and non-poly(A+) transcripts from 18 different types of biological samples (each with two replicates), including larval stages, pupal stages, and anatomical parts of 2-day-old posteclosion individuals (Fig. 2a). Only reads that could be assigned to protein-coding, lncRNA, and miRNA genes were considered in our analyses (Material and Methods and Supplementary Data 5). After gene-level quantification and normalization (see Material and Methods), and requiring ≥1 count-per-million (CPM) per sample, we found 7,475 genes expressed in all 36 samples and 14,839 genes if detection in at least two samples is required (Supplementary Fig. 14; Supplementary Data 6). Across different types of biological samples, we observed substantial differences, with the four head samples featuring the lowest (9,007–9,816) and the two whole-body male pooled samples featuring the highest (13,157, and 13,284) number of expressed genes (Supplementary Fig. 15). Multidimensional Scaling (MDS) analysis largely corroborated the developmental relationship among the sequenced samples and the replicate consistency (Supplementary Fig. 16), while revealing the marked signature of sex-biased expression on the global profile, which is also reflected as a distinctive trend shown by particular sets of genes (Fig. 2b, c, dotted boxes; Supplementary Note 5).

### Expression patterns across the transcriptome atlas

We next sought to investigate patterns of differential expression that could be particularly important for the developmental requirements and ecological pressures associated with broadly defined life stages in *D. plexippus*. For this, we avoided performing all possible pairwise comparisons among the 18 types of biological samples profiled (i.e. 153 comparisons), and instead focused, with a few exceptions, on comparisons among samples from the same stage (Supplementary Table 7; Supplementary Data 7). Requiring >2-fold expression difference at a 5% false discovery rate (FDR), and upon omitting genes only showing differences in our technical contrast (*Source* in Supplementary Data 7), 9469 genes –63.8% of all genes expressed– showed statistically significant differential expression in at least one of the contrasts performed, with 1549 (10.4%) only in one. The contrast corresponding to the transition between larva and pupa (P1:L5) displayed the largest number of expression changes, with 1366 genes increasing and 2126 decreasing in their expression (Supplementary Data 7; Supplementary Fig. 17).

**Fig. 3:**
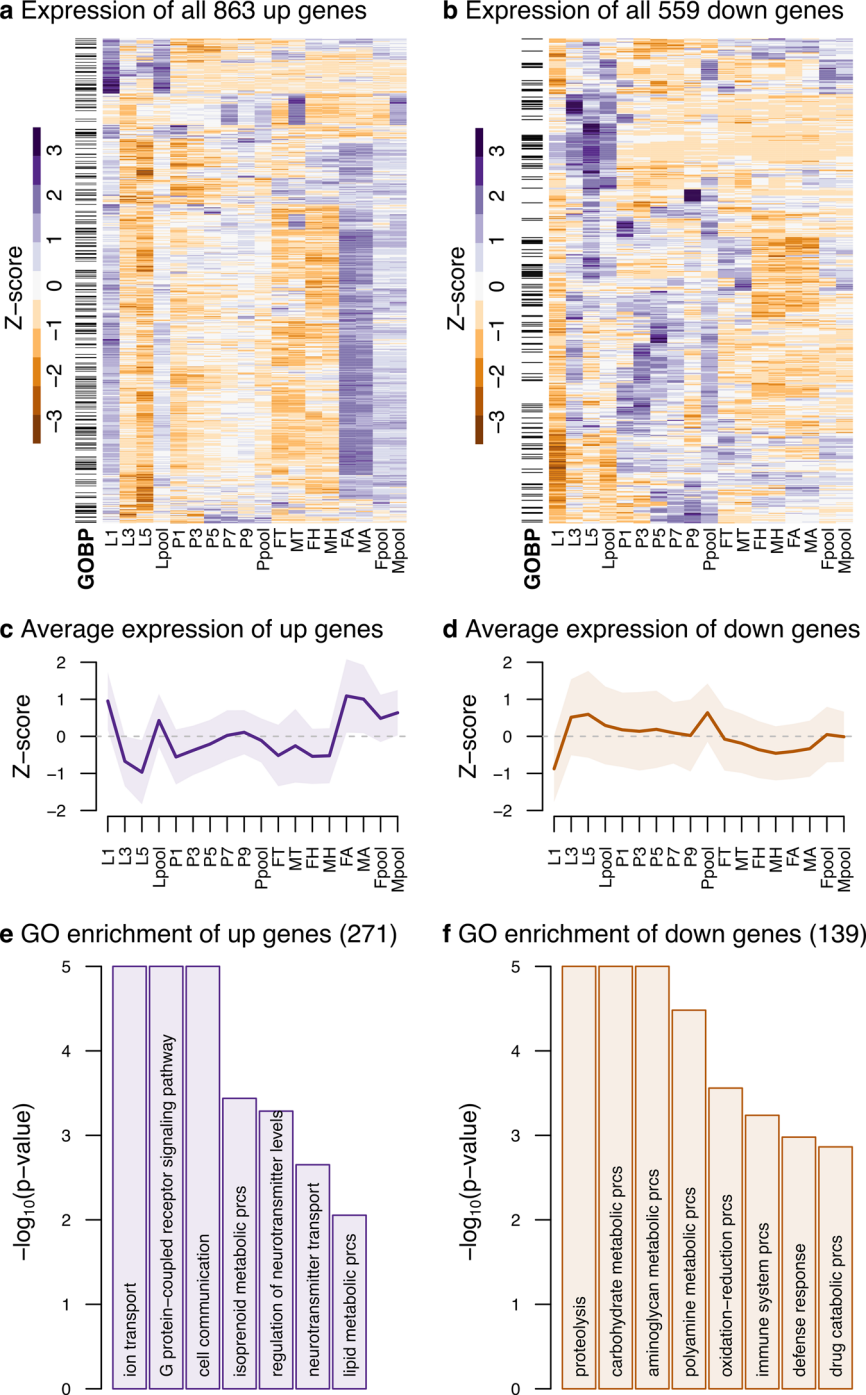
Differential expression results for the L1:L contrast. **a**, **b** Heatmaps of differentially upregulated and downregulated genes (5% FDR) in L1 relative to the average of L3 and L5. The average of each pair of replicates is used for the columns, and the rows are scaled using a Z-score. **c**, **d** Average gene expression trend based on the Z-score scaled expression of all upregulated and downregulated genes across all samples, respectively. The shaded area represents ±1 SD from the mean. **e**, **f** Topmost significantly enriched Biological Process GO terms amongst upregulated and downregulated genes, respectively. Only GO terms with an associated q-value cutoff smaller or equal to 0.2 are plotted. Raw data for these figures can be found in Supplementary Data 6-8.

As *D. plexippus* oviposition occurs in milkweed host plants that contain toxic cardenolides, larval instars are crucial in the context of host adaptation, particularly because sequestration of cardenolides is higher during early than late instars^20^. Therefore, transcriptome characterization of larval instars is vital to understand how gene function changes in the context of for example invasive species^46^. In total, 2,730 genes were differentially expressed in at least one of the three contrasts comparing the individual larval instars to the average expression across the other larval instars (*L1:L, L3:L*, and *L5:L*; Supplementary Data 7; Supplementary Fig. 17, 18). For example, in the contrast *L1:L*, which entails the comparison of L1 to the average of L3 and L5 (Fig. 3), we identified 863 genes as upregulated and 559 as downregulated in L1 (Fig. 3a, b; Supplementary Data 7). Although there is some overlap in the identity of the differentially expressed genes of the three possible larval contrasts, many of these genes are not exclusively expressed in larval stages. This is apparent when examining the patterns of the differentially expressed genes in one of the contrasts in the context of the remaining samples (Fig. 3c, d; Supplementary Fig. 18). For instance, L1 upregulated genes are also highly expressed in abdomen samples of both sexes. In this case, although we cannot discard some influence by early sexually differentiated genes, the observed pattern could represent a second wave of differential expression during adult tissue differentiation as observed in *D. melanogaster*^47^. Functional enrichment analyses also confirmed the differential overrepresentation of GO terms of the Biological Process ontology (*P*_adj_ < 0.2) across the three contrasts. For example, genes significantly upregulated in L1 relative to L3 and L5 appear to be preferentially related to signal transduction, neurotransmitter transport, and cell communication, while those downregulated were large to metabolic processes and immune response. These trends are compatible with a relative metabolic activation once the caterpillars start to feed and grow (thus lower expression in L1 but higher in L3 and L5), which is concomitant to a reduction of signaling processes required for very early development (Supplementary Fig. 18 and Supplementary Data 8). Importantly, when we compared all larval instars to the remaining individual samples (pupa and adult parts), we detected a significant enrichment for members of gene families implicated in detoxification. Thus, we found 74 genes encoding proteins that include a Major Facilitator Superfamily domain and 37 containing a Cytochrome P450 domain, while 14 genes encoded UDP-glucosyl transferases. When we analyzed the individual larval instars, Cytochrome P450 genes are relatively depleted in L1 but enriched in L5, highlighting further the differential transcriptome deployment of gene functions across larva instars.

With the end of the larval stage, the development of adult body structures is the primary process during pupation, which can be severely perturbed by parasites such as *Ophryocystis elektroscirrha*^21^. We predicted that expression patterns would differ markedly from those in larvae, as well as between early and late pupae. In good agreement, the contrast *P1:L5* revealed the upregulation in P1 of genes related to developmental and signaling pathways. Likewise, the contrasts *P1:P* and *P9:P* differ substantially in the functional attributes of the upregulated and downregulated genes. For example, while immune system and defense response are strongly associated with upregulated genes in *P1*, these appear not only to be downregulated in *P9* but replaced by others related to the nervous system and a wide variety of metabolic processes (Supplementary Fig. 18; Supplementary Data 8). In total, a minimum of 971 (*P5*:*P*) and a maximum of 2,626 (*P9*:*P*) genes were found to be differentially expressed across the contrasts only involving pupa samples (Supplementary Data 7), with a total of 3,942 genes differentially expressed in at least one of these contrasts (Supplementary Fig. 17).

To gain some insights into the 5,370 genes not called differentially expressed in any contrast, we performed a Weighted Gene Correlation Network Analysis (WGCNA) using all expressed genes^48^. Twenty-seven clusters were delineated (Fig. 2c; Supplementary Table 8). These clusters showed a variable degree of overlap with different sets of differentially expressed genes as well as conspicuous patterns of expression and functional enrichment (Supplementary Note 6; Supplementary Fig. 19; Supplementary Data 7, 8). Together, the patterns documented represent a rich dynamic portrait of the regulation of gene expression during most of the life cycle of *D. plexippus*.

### Sex-biased gene expression and dosage compensation

We found 14.45% (2,144/14,839) differentially expressed genes between the sexes in at least one of the four types of adult samples assayed (Supplementary Data 7; Supplementary Fig. 20). We observed very limited overlap among sample types, denoting differences in tissue composition and potential to harbor sex-biased expression. Further, in species with a *WZ/ZZ* female-heterogametic system, sex-biased expression results primarily from genes located on the heterochromosome *W*, only present in females, and from genes located on the heterochromosome *Z* in the absence of dosage compensation, i.e. the lack of whole-chromosome upregulation of this heterochromosome in females^49,50^. Recently, a comparison of the brain transcriptome between adult males and females documented a difference in dosage compensation between the anc*-Z* and neo*-Z*. The anc*-Z* showed roughly half of the expression of the autosomes in both males and females due to downregulation in males while the neo*-Z* showed almost equal level of expression relative to the autosomes in both sexes through a newly evolved upregulation in females^12^. Here, we examined the reproducibility of such patterns across the adult sample types assayed.

**Fig. 4:**
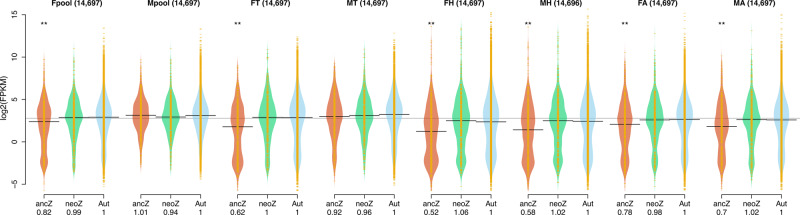
Whole-chromosome expression in females (*ZW*) and males (*ZZ*) in four sample types of *D. plexippus*. Sampled assayed: pools of whole-body males and females (Mpool, Fpool); individual samples of thorax (MT, FT), abdomen (MA, FA), and heads (MH, FH). The bean plots show the distribution of absolute normalized log_2_ expression values in FPKM for the ancestral (anc-) and neo (neo-) portions of chromosome 1 (i.e. the *Z* chromosome) and the autosomes. The horizontal line in each plot corresponds to the median expression value. A global median value across sample types is shown with a gray line in the background. The median *Z*:*A* ratios are shown at the bottom. For each sample type, statistical significance was established according to Wilcoxon signed-rank tests and upon applying a Bonferroni correction (**, *P* < 0.01). The number of genes considered is indicated on top of each bean plot. A minimum expression threshold of 0.01 FPKM was required. The results with other thresholds and for the autosomes considered separately can be found in Supplementary Figs. 21, 22 respectively. Raw data for this and related figures can be found in Supplementary Data 6-7.

First, we compared the median absolute expression *Z*:*A* ratio within each sex finding both commonalities and differences across sample types relative to the reported pattern^12^ (Fig. 4). For female whole-bodies and individual anatomical parts (heads, thorax, and abdomen), we observed a significantly lower median expression level for the anc*-Z*, but not for the neo*-Z*, relative to the autosomes, corroborating first the lack of complete dosage compensation for the anc*-Z*, and second the newly evolved complete dosage compensation for the neo*-Z* in this sex. Nevertheless, the median expression ratio of the anc*-Z* compared to the autosomes goes from nearly half in heads to values closer to 1 (maximum = 0.82, whole-bodies), denoting that absolute lack of dosage compensation happens only in heads. In contrast, for males, the presumed repression of the two Z chromosomes that should result in also a significantly lower expression level relative to the autosomes is observed in heads and, to some degree, in the abdomen as reported in other Lepidoptera^27,31,51^ but not for thorax and whole-bodies, suggesting that this pattern is likely tissue-dependent and therefore obscured in those sample types in which the repression mechanism does not predominate. Importantly, these patterns are robust across several expression thresholds (Supplementary Fig. 21) and are not the result of collapsing all the autosomes (Supplementary Fig. 22).

Next, we examined the median expression ratios between females and males for the autosomes (AA:AA) and the two portions of the chromosome *Z* (anc*-Z*:anc*-Z*anc*-Z*; neo*-Z*:neo*-Z*neo*-Z*) to assess the degree of expression equalization between the sexes, which is determined by the degree of dosage compensation of the chromosome *Z* in females and the downregulation of the chromosome *Z* in males (Supplementary Fig. 23-24). In good agreement with the observations above, the female to male ratios for the anc*-Z* are no different or slightly—but significantly—lower to those for the neo*-Z* and the autosomes in the abdomen and heads. In contrast, for the whole-body and thorax samples, the female to male ratio for the anc*-Z* shows only partial evidence of equalization between the sexes, being significantly lower. Only in whole-body samples, the neo*-Z* shows a slightly—but significantly—lower female to male ratio relative to the autosomes. Collectively, all these results underscore their sample-dependent nature while highlighting that dosage compensation is either absent or incomplete in the ancestral portion of *D. plexippus Z* chromosome, which is also reflected on the lack of perfect expression equalization between the sexes, this last pattern less acutely detected for the neo-portion of the *Z* chromosome.

Lastly, we examined the chromosomal distribution of sex-biased genes across the autosomes, the anc*-Z*, and the neo*-Z* in whole-body and thorax samples as those are the ones with more sex-biased genes (see above) and therefore we have more statistical power (Supplementary Table 9). Based on the incomplete lack of dosage compensation featured by the anc*-Z*, we predicted an enrichment of male-biased genes for this portion of the *Z* chromosome but not for the neo*-Z*, in good agreement with previous observations in other Lepidoptera^27,52,53^. Nevertheless, some interspecific variation relative to this pattern of enrichment has been observed^31^, underscoring the influence of other factors, mainly sexually antagonistic selection^54,55^, which might or might not align with the expectation based on the lack of complete dosage compensation^54,55^. For whole-body, we found statistically significant evidence of a global non-random distribution of male- and female-biased genes across the different portions of the heterochromosome *Z* and the autosomes, a pattern robust across different thresholds of minimum expression (Cochran–Mantel–Haenszel test; whole-body, Χ_MH_^2^ = 56.15, d.f.=2, *P* = 6.41×10^−13^; Supplementary Table 9). Analysis of the adjusted standardized residuals^56^ confirmed that the anc*-Z* exhibited enrichment for male-biased genes and depletion for female-biased genes, with the autosomes harboring comparatively a significantly lower proportion of the first gene category and a higher of the second. The neo*-Z* showed no bias of any kind. For thorax, support for the same global non-random distribution is found (Cochran–Mantel–Haenszel test; thorax, Χ_MH_^2^ = 21.87, d.f.=2, *P* = 1.79×10^−5^), although its statistical significance did not hold when the different thresholds of minimum expression were examined, arguably due to a more limited statistical power (Supplementary Table 9). Overall, our results adhere to the expected enrichment for male-biased genes in expression in the portion of the heterochromosome *Z* (anc-*Z*) that shows incomplete dosage compensation^27,31^. The neo-*Z* portion not only does not show the same pattern but also exhibits a significantly lower global fraction of sex-biased genes, in fact, even lower than the autosomes (Cochran–Mantel–Haenszel test; whole-body, anc-*Z* vs neo-*Z* vs A: Χ_MH_^2^ = 183.96, d.f. = 2, *P* = 2.2 × 10^−16^; neo-*Z* vs A: Χ_MH_^2^ = 21.17, d.f. = 1, *P* = 4.20 × 10^−6^), which holds across thresholds of minimum expression (*P*_adj_ < 0.05 for each threshold; Supplementary Table 10).

### Long non-coding RNAs

Owing to the limited functional characterization of lncRNAs beyond model organisms^57,58^ and the limited possibility to transfer information from other species due to poor sequence conservation^59^, we explored the lncRNA expression throughout our transcriptome atlas. As in other species^58,60,61^, we found that lncRNAs are expressed at a significantly lower level during the life cycle of *D. plexippus* (Supplementary Table 11), and exhibit more restricted expression profiles than protein-coding genes (Wilcoxon rank-sum test, *P* = 2.2 × 10^−16^; Supplementary Fig. 25). The 492 lncRNA gene models (78.7% of the 625 annotated) found expressed did not fall randomly across our sets of differentially expressed genes or WGCNA clusters (Fig. 2c). For example, lncRNAs were overrepresented in two clusters (*clust10* and *clust26*) (one-tailed Fisher’s exact test, *P*_adj_ < 0.05; Supplementary Table 8). Similarly, certain sets of differentially expressed genes were also enriched for differentially expressed lncRNAs (Supplementary Table 7; Supplementary Data 7). This was particularly evident in the comparison of pooled whole-body adult females compared to males (contrast *Sexes*). Among the differentially expressed genes in this contrast, and relative to protein-coding genes, we did not detect differences in the proportion of lncRNAs genes between females and males (20/245 vs 81/1,564, two-tailed Fisher’s exact test, *P* = 0.1). In sharp contrast, however, and again relative to protein-coding genes, we did detect a significant increase of differentially expressed lncRNAs when we compared pooled male and female adults against pooled larva and pupa (contrast *Adulthood*, Supplementary Data 7; 71/1,245 vs 33/1,228, two-tailed Fisher’s exact test, *P* = 4.0 × 10^−4^). Thus, while 23.37% (115/492) of the lncRNAs shows statistically significant sex-biased expression, only 14.15% (2,027/14,327) of the protein-coding genes do (2-sample test for equality of proportions with continuity correction, Χ^2^ = 32.0, d.f.=1, *P* = 1.54 × 10^−8^).

Further examination of the GO terms associated with the protein-coding genes enriched in the same clusters as lncRNA genes allowed the tentative functional categorization of the latter. For example, *clust26* includes a low number of genes but harbors the highest fraction of lncRNAs (19/37, 51.3%), showing high expression in most of the pooled samples. Interestingly, *clust26* shows increased expression during larva development, and higher expression in heads compared to thorax. Although only four genes have an annotated GO Biological process, two of them are annotated with the nitrogen compound transport term and one with the RNA transport term (q-value=0.02 for both). In summary, lncRNAs likely participate in essential biological processes during the whole life cycle of *D. plexippus*, are more finely regulated during adulthood than during previous developmental stages, and are more heavily influenced by the differences between the sexes compared to protein-coding genes.

## Conclusion

A better understanding of the adaptation of *D. plexippus* to a changing environment requires both the use of genomic resources that represent more accurately the population genetic diversity of the species, and more comprehensive knowledge about gene function and regulation during the life cycle. The reference-quality genome assembly from a non-migrating population reported here will help mitigate the insufficiencies derived from depending on a single reference-quality genome assembly, including the presence of minor alleles for a set of loci, missing sequence, and the underrepresentation of genetic diversity at structurally dynamic regions^13–15^. Further, the portrait of the transcriptome program obtained here can serve as a baseline for the future exploration of commonalities and differences across non-migratory populations, and among these and migratory populations. Likewise, this portrait will facilitate the study of the transcriptome responses underlying genotype-by-environmental interactions in the context of different host species^46,62–64^ as understanding the developmental transcriptome should clarify the interplay between gene regulation and viability on alternative hosts.

## Methods

### Butterfly husbandry

Newly hatched larvae of *D. plexippus* were collected from wild *Asclepias curassavica* on the campus of the National Laboratory for Genomics of Biodiversity in Irapuato, Guanajuato, México. The early first instar caterpillars were placed in individual vials and fed with fresh *A. curassavica* leaves ad libitum on a 12–12 h light-dark cycle at 25 °C and ~50% relative humidity until adults emerged. All stages were precisely identified by measuring head capsules left after molting.

### Genomic DNA extraction and sequencing

A two-day-old pupa was fast frozen in liquid nitrogen and preserved at −70 °C until DNA extraction. Genomic DNA was extracted with the Blood and Cell Culture DNA Kit (QIAGEN). All equipment was cleaned with DNAaway (Thermo-Fisher Scientific) prior to grinding the specimen in a mortar kept cold with liquid nitrogen. Powdered pupal tissue was incubated with RNAse-A and Protease for 2 h while gently rocking. DNA was purified by gravity flow with QIAGEN Genomic-tips, precipitated with isopropanol, and washed twice with cold 70% ethanol. All centrifugation steps were performed in 15 ml tubes in a pre-chilled centrifuge at 4 °C. The DNA pellet was resuspended in QIAGEN EB buffer overnight. One μg of unsheared genomic DNA was saved for Illumina PE-150 sequencing in a HiSeq 4000 instrument over one lane. The remaining genomic DNA was sheared with blunt-end needles as reported^65^; except for 20 pumps with a 21 gauge 1.5” blunt end needle, followed by 10 pumps with a 24 gauge 1.5” blunt end needle (Jensen Global, Santa Barbara, CA). Ten μg of sheared DNA were used to make the SMRT-bell template library following manufacturer specifications. The library was size selected (15–80 kb) using the Blue Pippin size selection instrument (Sage Science) and then sequenced on six SMRT cells (one cell at 2 pM and five cells at 6 pM) using 1 M v2 chemistry on a PacBio Sequencing Sequel instrument with a 10 h movie time. Concentration and purity of all the genomic DNA submitted for sequencing were determined using a Qubit v2 fluorometer (Life Technologies) and an 8000 NanoDrop (Thermo Scientific), respectively. All genome sequencing was performed at the UCI Genomics High-Throughput Facility.

### Total RNA extraction and sequencing

Fourteen specific stages and anatomical parts were RNA-sequenced: 1st (pools of six to obtain sufficient RNA), 3rd and 5th instar larvae; day 1, 3, 5, 7, and 9 pupae; 2-day-old adult female and male thoraces, heads, and abdomens. Adult individuals were anesthetized in a cage at -20C for five minutes, sexed, and the wings removed, after which they were dissected into the indicated anatomical sections. All samples were fast frozen in liquid nitrogen and preserved at −70 °C until RNA extraction. With the exception of 1st instar larvae, which were mechanically homogenized in TRIzol using Teflon homogenizers, the rest of sample types were weighed after pestle homogenization in a ceramic mortar using liquid nitrogen, adjusting for sample quantity prior to be stored in TRIzol. Total RNA was subsequently extracted using Direct-zol RNA MiniPrep extraction kit (Zymo Research) according to manufacturer recommendations, except for all pupa samples and adult abdomens which were first extracted with TRIzol also following manufacturer recommendations and then purified with Direct-zol columns. RNA yield, purity, and integrity were evaluated with conventional methods, using a Qubit 2.0 Fluorometer, a NanoDrop 8000 Spectrophotometer, 1% agarose gels, and RNA 6000 Pico and RNA 6000 Nano kits—depending on the experiment—with a BioAnalyzer 2100 (Agilent Technologies Inc.). Libraries for each sample type were prepared using the TruSeq Stranded Total RNA Library Prep Kit (Illumina), multiplexed and 75 bp paired-end sequenced over 4 lanes on an Illumina NextSeq 500 Sequencing System at the sequencing core facility at LANGEBIO. Aliquots from samples of pooled individuals belonging to the same broadly defined developmental stage (Lpool = 1st, 3rd, and 5th instar larvae; Ppool = 1, 3, 5, 7, and 9-day pupae; Mpool = heads, abdomens, and thoraces from adult males; Fpool = heads, abdomens, and thoraces from adult females) were mixed equimolarly. For these pooled samples, non-poly(A) enriched stranded libraries were constructed using the TruSeq Stranded Total RNA Library Prep Kit (Illumina) and the Ribo-Zero Gold Set A kit (Epicenter). These libraries were subsequently multiplexed and 100 bp paired-end sequenced over one lane on an Illumina HiSeq 2500 instrument at the University of California Irvine Genomics High Throughput Facility.

### De novo genome assembly construction

We generated different assemblies to be used in different analyses or to be associated with different stages part of the same computational pipeline. Illumina paired-end reads were trimmed and filtered out for low-quality base calls and undesired adapter presence using Trimmomatic v.0.35^66^ and FastQC 0.11.5^67^, and used to generate an assembly with Platanus v.1.2.1^68^, which can accommodate any residual heterozygosity, using default parameters. The quality of the Platanus assembly was confirmed upon finding that the mapping back rate, i.e. the percentage of reads aligned against the constituent collection of contigs, was ~99.4%. A K-mer analysis was performed to estimate the level of heterozygosity in our sequenced sample and to recalculate the genome size, as a control, of *D. plexippus*, using GenomeScope^69^. Additional K-mer spectra visualizations were done with KAT v2.4.1^70^.

A draft genome assembly based on PacBio raw sequencing reads was used using Canu v1.6^71^ specifying a genome size of 250 Mb, a corrected error rate of 0.045, a raw error rate of 0.3, a minimum overlap length of 500 nt, and a minimum read length of 1000 nt. The resulting assembly featured an NG50 = 3.3 Mb (NG50 refers to the length of the smallest contig added to cover 50% of all nt estimated in the genome^72^), a total size of 458.6 Mb, and an error rate upon self-correction of 0.045. This intermediate assembly was subsequently polished through four rounds of Pilon v1.22^73^ using the alignment information from Illumina trimmed reads generated with bwa v0.7.17-r1188^74^. Redundancy minimization was performed with HalploMerger2_20180603^75^ using default parameters except for splitting the target in fast files of 5 × 10^6^ nt instead of 5 × 10^7^ nt and with a query size of 160 × 10^6^ nt instead of 160 × 10^7^ nt. Then, FinisherSC^76^, along with MUMmer v4.0.0beta1^77^, was used to upgrade the haploid assembly using all raw PacBio reads (NG50 = 5 Mb, total size = 434.9 Mb). At this stage, we polished our expanded diploid assembly again through five additional rounds of Pilon v1.22, followed by HalploMerger2_20180603, to generate the final haploid collection of contigs. These contigs were scaffolded with RaGOO^78^ using the most contiguous of our intermediate assemblies as a reference (Quickmerge in Supplementary Table 1). This Quickmerge assembly was obtained in the course of our attempts to enhance contiguity and resulted from merging our polished DBG2OLC assembly, which combined the Illumina-based Platanus assembly and raw PacBio reads, with our polished Haplomerger2 canu-derived assembly. We chose the Quickmerge assembly as opposed to the most contiguous assembly because the former had a higher BUSCO completeness score (Supplementary Table 1).

Quality metrics of the selected and non-selected assemblies, as well as key intermediate assemblies generated through different approaches, were extracted using the script NX.pl (https://github.com/YourePrettyGood/RandomScripts/blob/master/NX.pl). Genome assembly completeness was assessed by calculating different mapping back rates of sequencing reads from 72 Illumina genomic DNA sequencing libraries^7^ that were considered suitable (see below). Read mapping was done with bwa v0.7.17-r1188 using the parameter *-h 99999* to avoid discarding multimapping reads. The different rates calculated using the counts for mapped, properly paired, and total reads were obtained with SAMtools v1.9^79^. Gene-level completeness was evaluated through CEGMA v1.0^80^, and BUSCO v2.0.1 and BUSCO v4.0.5^35^, using the gene sets of Endopterygota_odb9 (*n* = 2,442) and Lepidoptera_odb10 (*n* = 5,286), respectively. Lastly, differences in scaffolding between DpMex_v1 and Dpv3 were determined with RaGOO^78^ using the former as a reference, which allowed the identification of chimeric contigs in Dpv3. Briefly, if a Dpv3 scaffold aligns against two different DpMex_v1 contigs over at least 10 kb in each case, and the span covered of these contigs was in both cases greater than 100 kb and 5% of the contig span, the Dpv3 scaffold was dubbed as chimeric.

### Repeat annotation

Ab initio repeat modeling was done with RepeatModeler v.1.0.11^81^. The filtered RepeatModeler database was combined with consensus Lepidopteran repeats found at Dfam_Consensus-20170127^82^ or RepBase-20170127^83^ databases. The global set of repeats, including low-complexity regions and simple repeats, was used to feed RepeatMasker v.4.0.7^84^ to softmask the final polished genome assembly.

### Gene annotation

Funannotate v1.5.3 docker image^85^ was used to train Augustus v3.2.3^86^, predict gene models, and perform functional annotation. As input for optimizing the performance of Augustus v3.2.3^86^, funannotate used 2,404 PASA v2.3.3 gene models^87^. To obtain this training gene model set, transcripts were de novo assembled with Trinity v2018-2.8.3 under settings–SS_lib_type RF^88^, using all poly(A) RNA-seq paired reads after adapter removal with Trimmomatic v0.32^66^. These transcripts were aligned to the genome under PASA using BLAT v36^89^, obtaining a first set of gene models. The 500 longest non-redundant ORFs associated with the PASA gene models were used to train TransDecoder v5.2.0^90^. Then the gene models were selected according to their abundance as estimated by Kallisto v0.44.0^91^ under settings—rf-stranded using the Trinity normalized reads. Ultimately, BRAKER v2.0.3b^92^ trained Augustus with the retained gene models.

For gene prediction, funannotate aligned mRNAs and proteins from the previous annotation (official gene set 2, OGS2)^8^ with minimap v2.14-r883^93^ under settings -ax splice --cs -u b -G 3000, and Diamond blastx v0.8.22^94^, respectively. Protein alignments were further refined by funannotate, including 3 kb upstream and downstream of the region of alignment, and subsequently executing Exonerate v2.4.0^95^. Additionally, funannotate parsed the introns supported by alignments of poly(A) RNA-seq reads generated with HISAT v2.1^96^ under settings–rna-strandness RF–max-intronlen 10,000. This combination of hints (protein alignments, transcript alignments, and intron locations) was used by Augustus to predict a second set of 16,756 gene models. Of them, 9,695 were dubbed as highly supported, i.e. had more than 90% of their model supported either by intron hints, transcript alignments, or protein alignments. GeneMark-ET v4.35^97^, under settings–max_intron 3,000–soft_mask 2,000, was also run independently to predict a third set of gene models but only relying on intron hints.

The PASA, Augustus highly supported, Augustus not highly supported, and GeneMark prediction sets were combined by EVidenceModeler^98^, assigning them 10, 5, 1, and 1 relative weights, respectively. The predictions were further filtered by removing genes shorter than 50 aa in length, or that had high sequence similarity (diamond blastp --sensitive --evalue 1e-10) to the repeat database included in funannotate, or that had more than 90% of the model intersecting regions masked by RepeatMasker. The filtered set of gene models was updated in order to include UTR information by two executions of the PASA annotation comparison using the Trinity transcripts and filtering gene models according to transcripts per million as calculated by Kallisto. Alternative transcripts were only kept if they were at least 10% as highly expressed as the most highly expressed transcript per gene.

Non-coding genes were annotated with the following tools: tRNA genes, tRNAscan-SE v.2.0^99^; rRNA genes, RNAmmer v.1.2^100^; and for a variety of other RNA genes, Infernal v1.1.1^101^. Specifically, for miRNA-encoding genes, we used BLASTn to locate the most recent annotation of these genes^102^. In addition, FEELnc^103^ classified lncRNAs from the transcripts assembled by StringTie v1.3.2d^104^, and considering protein-coding predictions described above. LncRNA gene models were required to generate transcripts longer than 200 nt, encompass at least one splicing junction, and be antisense if overlapping with a protein-coding gene model. Finally, any protein-coding gene that overlapped with a rRNA gene on either strand was discarded from our transcriptome analyses.

### Homology identification

The set of protein-coding genes as from funannotate was then used by OrthoFinder v2.2.6^105^ under the settings –S diamond –M msa to establish orthologous calls across protein sets from 7 other species, which were retrieved either from NCBI or lepBase (Supplementary Table 5). Only the longest predicted protein per gene model was used in the analysis. Orthogroups with other lepidopterans and *D. melanogaster* were identified independently for our gene predictions and the annotation of the previous assembly, i.e. OGS2. Also, when identifying gene correspondence between our predictions and OGS2, all other species were excluded from the input to OrthoFinder. 1-to-1 orthologs were grouped in microsynteny blocks by DAGchainer^106^ under default parameters. The software Circos^107^ was used to graphically represent the chromosomal mapping of microsynteny blocks between lepidopterans.

### Sex-dependent sequence coverage analysis

Illumina genomic DNA sequencing data from 80 *D. plexippus* individuals were retrieved^7^. Supplementary Data 4 lists their GenBank SRA accession numbers. Sequencing reads from each sample were aligned against the DpMex_v1 assembly using bwa v0.7.17-r1188 under the *-M* option. Contig median coverage was calculated using mosdepth^108^. We calculated a normalized contig coverage for each sample as the contig scaffold coverage divided by a weighted average, according to the total number of reads mapped to each contig, of the median contig coverage. Five samples (SRR1548577, SRR1549538, SRR1552003, SRR1552104, and SRR1552110) were filtered out due to having less than an average coverage of 5, which was coincidental with an unusual distribution of their normalized coverage relative to the rest of samples. To estimate the male:female (M:F) coverage ratio, we averaged the normalized coverage per contig per sex as reported^10^. Further, the cumulative fraction of the *Z* chromosome covered for at least a given normalized coverage value was plotted to explore the presence of outlier samples. Two samples labeled as female (SRR1552102 and SRR1552103) stood out as they had a normalized coverage above 0.98 for more than 50% of the heterochromosome *Z*, which is similar to the typical distribution for male samples. Similarly, one sample labeled as male (SRR1552006) had a normalized coverage distribution that resembled that of females (Supplementary Fig. 13). Lastly, two additional samples (SRR1548506 and SRR1549526) exhibited highly heterogeneous median coverage among contigs. These five samples were also excluded from further analyses.

### RNA-Seq quality control and alignment

The raw sequencing data in fastq files were preprocessed with fastqc^67^ and multiqc^109^ to verify that the sequencing was of sufficient quality; all files passed visual inspection. Reads were aligned to the genome based on a two-pass strategy using STAR v2.7.3a^110^. The genome was first indexed for STAR including the exons from the GFF annotation file. During the first pass of alignments, only the Splice-Junction files were stored. The complete set of Splice-Junction files were used during the second pass to inform the final alignments. Non-default parameters used during both passes are: --outFilterMultimapNmax 500, --outFilterMismatchNoverReadLmax 0.1, --alignIntronMin 5, and --alignIntronMax 20000. Adapter sequences were trimmed during alignment, using the parameter --clip3pAdapterSeq AGATCGGAAGAGCACACGT AGATCGGAAGAGCGTCGTG.

### Gene-level expression quantification

The annotation file was first processed to remove overlaps, using the R package GenomicRanges^111^. As protein-coding and lncRNAs were our focal interest (first set), they were considered separately from rRNA, tRNA, and miRNAs (second set). Any genomic coordinate overlaps between the second and the first gene types were deleted from the first. All the remaining coordinates in both sets were collapsed at the exon level. Introns were determined as the spaces left between collapsed exons for every gene. The resulting annotation was used as the input for featureCounts^112^ in order to determine separate exon and intron gene expression counts for each library. Non-default parameters were: largestOverlap=TRUE, fraction=TRUE, strandSpecific=2, and isPairedEnd=TRUE. On average, 87.4% of the reads from each sample mapped to our genome assembly, with 76.7% of all sequencing reads being confidently assigned to the annotated fraction of the assembly. Reads that only mapped to introns (2.4%), rRNA (32.7%) and tRNA (<0.1%) were discarded before further processing. In total, 41.6% of the reads, those that mapped to protein-coding, lncRNA, and miRNA genes were considered in downstream analyses (Supplementary Data 5). Exonic gene-level expression for the three indicated classes of genes were stored as log_2_CPMs (Supplementary Data 6).

### Differential expression analysis

Raw counts were further processed using the edgeR package^113^. Each type of sample (*e.g*. L1) was assigned to a distinct factor level. Only genes with ≥1 count-per-million (CPM) in at least two samples were kept. Normalization factors were calculated with the calcNormFactors function and the TMM method^114^. Normalized log_2_CPM, as well as fragments-per-kilobase-per-million (FPKM) expression, values were saved for subsequent analyses. Multidimensional Scaling plots were used to determine the relationship between samples and grouping of replicates. During analyses, several samples from individuals were determined to have a strong male-specific expression profile. These samples (L5.1, P1.1, P3.1, P5.1, P7.1, P7.2, P9.1, MT.1, MT.2) were assigned to an extra *male* batch factor. Negative binomial dispersion values were calculated, used to fit generalized linear models, and to test for differential expression with glmTreat^115^. This approach tests for differences in expression that are significantly higher than a threshold, in this case a fold-change of 2. Finally, to select differentially expressed genes, a 0.05 False-Discovery Rate (FDR) threshold was used, according to the Benjamini-Hochberg method^116^. In some analyses, a less strict likelihood ratio test was also performed to find fold-changes significantly higher than 0. For both approaches, 24 differential expression contrasts were chosen to represent individual samples and transitions across the atlas (Supplementary Data 7).

### Transcriptional network and clustering

Weighted Gene Correlation Network Analysis (WGCNA)^117^ was used to generate a transcriptional network, considering all 14,839 genes that were also used for differential expression. The library-size normalized log_2_CPM gene expression values were used (Supplementary Data 6). The pooled samples sequenced at the UCI facility (Supplementary Data 5) were first removed to avoid correlations between different sequencing facilities and library prep methods affecting the network. As recommended by the package authors, a range of soft-threshold values were explored, and 14 was selected to optimize the fit of the network to a scale-free topology. A topological overlap similarity matrix was calculated, preserving the sign of the correlations. Hierarchical clustering with the average agglomeration method, and a dynamic tree cutting procedure were used to obtain gene clusters. To allow for relatively smaller clusters, the minimum module size was set to 10, which resulted in 27 clusters.

### GO enrichment analysis

For each group of genes resulting from differential expression or network clustering, a test for enrichment of GO terms was performed using clusterProfiler^118^. All GO terms assigned in our annotation were considered, as well as all their ancestor terms. During each enrichment test, GO terms with less than 5 or more than 500 genes were ignored. Although GO terms are not independent due to their hierarchical nature, multiple-testing correction using the q-value method was performed^119^. A *q*-value cutoff of 0.2 was used as a threshold for GO term enrichment.

### Gene expression specificity

The tau index, a measure of sample expression specificity^120^, was calculated considering the 36 RNA-seq samples and using log_2_CPM expression values. Tau ranges from 0 to 1, with values closer to 1 indicating more restricted expression and values closer to 0 indicating more widespread expression.

### Statistics and reproducibility

Most statistical analyses were executed primarily in R^121^. Plotting was also performed using base R functions, as well as with those included in the R packages beanplot^122^, ggplot2^123^, ggVennDiagram^124^, pheatmap^125^, cowplot^126^, and gridGraphics^127^. Individual statistical tests, parameters, thresholds, and statistically significant results are indicated in the corresponding figure or table. The number of individuals sequenced from different developmental stages as well as the level of replication in RNA-seq are indicated in the corresponding sections of the Methods and in Fig. 2. All code used is available upon request.

### Reporting summary

Further information on research design is available in the Nature Research Reporting Summary linked to this article.

## Supplementary information

Supplementary Information

Details of Supplementary Data files

Supplementary Data 1

Supplementary Data 2

Supplementary Data 3

Supplementary Data 4

Supplementary Data 5

Supplementary Data 6

Supplementary Data 7

Supplementary Data 8

Reporting Summary

## Data Availability

All raw sequencing data were deposited as part of the NCBI BioProject PRJNA663267. The Whole Genome Shotgun project has been deposited at DDBJ/ENA/GenBank under the accession JAEQBL000000000. The version described in this paper is version JAEQBL010000000. The annotation file for the sequenced assembly is available at Zenodo^128^ (10.5281/zenodo.4470132).
