# Supplementary material for: A de novo transcriptional atlas in Danaus plexippus reveals variability in dosage compensation across tissues
Source: Commun Biol. 2021 Jun 25;4:791. doi: 10.1038/s42003-021-02335-3 (PMC8233437; doi:10.1038/s42003-021-02335-3)
Supplement: Supplementary file 1 — Supplementary Information [file 42003_2021_2335_MOESM1_ESM.pdf]

SUPPLEMENTARY INFORMATION

A *de novo* transcriptional atlas in *Danaus plexippus* reveals variability in dosage compensation across tissues

José M. Ranz [1*], Pablo M. González [2†], Bryan D. Clifton [1], Nestor O. Nazario Nazario-Yepiz [2], Pablo L. Hernández-Cervantes [2], María J. Palma-Martínez [2], Dulce I. Valdivia [2], Andrés Jiménez-Kaufman [2], Megan M. Lu [1], Therese A. Markow [2,3], Cei Abreu-Goodger [2‡*]

[1] Department of Ecology and Evolutionary Biology, University of California Irvine, Irvine CA 92647, USA

[2] Unidad de Genómica Avanzada (Langebio), CINVESTAV, Irapuato GTO 36824, México

[3] Section of Cell and Developmental Biology, Division of Biological Sciences, University of California San Diego, La Jolla CA 92093, USA

* E-mail: jranz@uci.edu, cei.abreu@cinestav.mx

† Present address: Wellcome Trust Sanger Institute, Wellcome Trust Genome Campus, Hinxton, UK.

‡ Present address: Institute of Evolutionary Biology, School of Biological Sciences, University of Edinburgh, Edinburgh, UK.

SUPPLEMENTARY TEXT

Supplementary Note 1: Contamination assessment

Despite all the measures adopted to avoid DNA contamination from other unintended species, we interrogated for any contamination of our sample by using BlobTools [1]. A BLASTN search [2] for each contig was performed against the NCBI nucleotide dataset (nt) and diamond BLASTX against the UniProt database. Read coverage was calculated with our Illumina reads aligned using BWA and GC content was calculated for each contig and together plotted as blobplots (Supplementary Fig. 2a). For DpMex_v1, visual inspection of its graph revealed some traces of Nematoda, Streptophyta, and Proteobacteria among others but found no evidence of any non-arthropod major source of contamination [3-5]. BLAST searches and read coverage of the previous genome assembly, Dpv3 [6], yielded a plot with a similar taxonomic composition although the fraction of contigs with no significant hit (primarily as a result of short length) is proportionally larger than in the case of DpMex_v1 (Supplementary Fig. 2b). For none of the assemblies, it is possible to distinguish distinctive clustering of non-arthropod-related taxonomically set of contigs*, i.e.* contigs with non-arthropod related sequences are primarily interspersed with the crowd of contigs enriched for arthropod-related contigs. This pattern not only is consistent with a similar taxonomic composition of both assemblies but also that the odds of non-arthropod traces being true contamination are very low unless they came from an obligate endosymbiont.

Supplementary Note 2: Presence of alternative haplotypes

The possibility of preservation of alternative haplotypes in the final assembly was evaluated through the examination of dot plots generated between DpMex_v1 and Dpv4 [7] and the search for overrepresentation of BUSCO duplicates. Both analyses, in good agreement, pointed to contigs Sc0000030 and Sc0000031 as those most likely representing different haplotypes. Specifically, the BUSCO output revealed 41 duplication events involving 124 duplicates. Eight duplication events occurred at intracontig level while the rest (80.5%) involved different contigs. Of all duplicates, 42 (33.9%) involved Sc0000030 and Sc0000031, which showed a virtually perfect alignment (Supplementary Fig. 6). The next pair of contigs harboring more

duplicates was Sc000002 and Sc000040, with seven, which represents just 5.6% of all duplicates. The dot plot of these two contigs (not shown) does not support that they represent different haplotypes.

Supplementary Note 3: Further feature comparison among *D. plexippus* genome assemblies

The DpMex_v1 assembly has a similar span as Dpv3 and Dpv4 but virtually no gaps, whereas 2.3% of Dpv3 and Dpv4 correspond to gaps. With regards to the level of agreement in internal order and orientation of scaffolds, we detected 131 differential sequence discontinuities in Dpv3 relative to DpMex_v1 affecting 61 Dpv3 scaffolds (Supplementary Data 1). Importantly, three of these discontinuities were already reported as misassemblies of Dpv3 [8]. Relative to Dpv4, the two genomes exhibit a high degree of collinearity at a whole-chromosome scale so that large-scale misassemblies in any of them are unlikely (Supplementary Fig. 7). Nevertheless, at a finer scale, multiple small differences were uncovered. At this time, it is not apparent what is the relative contribution of *bona fide* genetic differences between the individuals sequenced and misassembly errors. The differences observed in the K-mer spectra between both assemblies (Supplementary Fig. 8), particularly the underrepresentation of high multiplicity k-mers, *i.e.* those less likely to represent sequencing errors, which are present in the sequencing data generated for the DpMex_v1 assembly and absent in Dpv3, is striking.

Lastly, DpMex_v1 exhibits increased gene completeness compared to Dpv3 and Dpv4, and substantially higher than that of for example *H. melpomene* v2.5, according to the BUSCO analysis. Using CEGMA, which uses a different reference gene set, the trend was also found (Table 1). The DpMex_v1 assembly harbors more unicopy complete (54 and 55) and fewer (51 and 58) duplicate BUSCOs than Dpv3 and Dpv4, respectively. Relative to the missing BUSCOs between DpMex_v1 and Dpv3/Dpv4, we found an inconsistent outcome even for the two last assemblies (Supplementary Table 3), which presumably only differ in the scaffolding steps. Through TBLASTN (<E10^{-5} and at least 50% of the coverage of the BUSCO protein), we confirmed the presence of significant hits for 48 of the 57 missing BUSCOs, including the 20 BUSCOs undetected across all three *D. plexippus* assemblies (Supplementary Table 3). These were also missed in the genome assembly of *H. melpomene* v2.5, confirming that the DpMex_v1 assembly is the

most complete among those available in *D. plexippus* (Supplementary Note 3; Supplementary Table 3; Supplementary Data 2).

Supplementary Note 4: Additional controls to the genome assembly anchoring process to the *D. plexippus* chromosomes

To substantiate further our anchoring process, we used two approaches. First, we reasoned that if the overall macrosynteny conservation assumption is correct, the majority of the gene content in the chromosomes of *D. plexippus* should also be found in species more distantly related species compared to *M. cinxia.* Using the DpMex_v1 assembly, we explored the degree of chromosome-level synteny between *D. plexippus* and two other increasingly distant Lepidoptera: *H. melpomene*, another member of the family Nymphalidae; and the silkmoth *B. mori,* a representative of the moths and whose ancestor split from that of butterflies at ~100 mya, *i.e.* during the Cretaceous period [9]. Based on 1-to-1 orthologs, we found marked patterns of synteny conservation between *D. plexippus* and both *H. melpomene* and *B. mori* (Supplementary Fig. 12). Essentially, at least 97% of gene content of each *D. plexippus* chromosome is found in one chromosome of *H. melpomene* (96% relative to those of *B. mori*), with the exception of chromosome *1*, which is the result of a *D. plexippus* lineage specific fusion and therefore is associated with two chromosomes in the other species (chromosomes *2* and *21* of *H. melpomene*; chromosomes *1* and *16* of *B. mori*). Similarly, it is not unusual that the gene content of two chromosomes of *D. plexippus* reside in one particular chromosome of either *H. melpomene* or *B. mori*, denoting lineage-specific fusions during the evolution of these species. This is the case for the 9 fusions occurred in the lineage of *H. melpomene* (*1: 2-27; 6: 10-31; 7: 12-28; 10: 6-22; 12: 13-25; 13: 11-26; 17: 15-29; 18: 14-24; 19: 4-23*) and for the two fusions occurred in the lineage of *B. mori* (*11*: *12-31*; *24*: *27-29*) that do not involve the ancestral chromosome *30* of *M. cinxia* [10,11], as for its ortholog chromosome in *D. plexippus* we do not have 1-to-1 orthologs with any of these three species. Overall, we find that only 0.52% of the 1-to-1 orthologs between *D. plexippus* and *H. melpomene,* and 1.30% of those between *D. plexippus* and *B. mori,* might reflect gene transposition events involving different chromosomal elements of the ancestor to these species.

Our second approach focused on the reliability of our contig anchoring process in relation to the autosomes and the heterochromosomes. For that, we calculated the log_2 male:female (M:F) coverage ratio for every contig by mapping Illumina genomic DNA sequencing data from 70 libraries corresponding to 36 males and 34 females [12] (Fig. 1b; Methods; Supplementary Fig. 11b and 13; Supplementary Data 4). Only a twofold difference in coverage, *i.e.* a log_2(M:F)=1, is expected for the heterochromosome *Z* as it is present in a single copy in the heterogametic sex *ZW* [8,13]. Only the contig corresponding to chromosome *Z* featured a log_2 ratio close to 1, as expected according to its two copies in males but only one in females [8,13]. Nine additional contigs, none of them anchored to any chromosome, exhibit log2 values < -1, which opens the possibility that they can be components of the *W* chromosome. These contigs are mainly composed of genome-wide repeats, are relatively short (<0.59 Mb; median size 0.14 Mb), and exhibit very low gene densities; in fact, seven of the contigs harbor no annotated gene (Fig. 1c-d; Supplementary Data 3). Collectively, the results from these two analyses strongly support the reliability of the anchoring process of the DpMex_v1 genome assembly to the autosomes and the heterochromosome *Z* of *D. plexippus.*

Supplementary Note 5: Signature of sex-biased expression

The male-biased gene sets highlighted in Fig. 2b exhibit the same bias in thorax but not in head or abdomen, which could plausibly result from a comparatively delayed sexual maturation of those anatomical parts in relation to thorax. Our initial MDS analysis indicated that several samples were grouping in a sex-specific fashion instead of according to developmental stage, leading to some replicates not being positioned closely together (Supplemental Fig. 16a). Once the sex-biased genes were excluded, the MDS grouped all samples consistently according to their stage, and biological replicates were also positioned systematically closer (Supplementary Fig. 16b). Although larval samples did not perfectly separate by instar, the pupa samples followed a consistent bidimensional trajectory, starting at day 1 (P1) and ending at day 9 (P9).

Supplementary Note 6: Weighted Gene Correlation Network Analysis (WGCNA)

A WGCNA [14] was performed with all expressed genes to uncover functional trends not captured by our differential expression analysis (see main text). Twenty-seven clusters were delineated (Supplementary Table 8), with some of them corresponding quite tightly to particular sets of differentially expressed genes (*e.g. clust19* and *Sexes.up*; Fig. 2c), while others overlapped with different sets of differentially expressed genes in Supplementary Data 7 (*e.g. clust03* and *clust05*). The 5,370 genes not associated with any contrast in our differentially expression analysis were found over 26 of the 27 delineated WGCNA clusters. Notably, this complementary clustering approach revealed additional biologically coherent expression patterns. For instance, *clust07* included 633 genes that progressively diminish their expression during all larva and pupa stages and are further diminished in the adult samples (Supplementary Fig. 19). These genes are enriched in GO terms related to ncRNA metabolism, RNA processing and DNA replication, and PFAM RNA-binding domains. Another cluster, *clust09*, consists of 504 genes that follow almost the opposite pattern: increasing during development and reaching their highest level in the adult samples (Supplementary Fig. 19; Supplementary Data 8). Interestingly, these genes show few signatures of common functionality, except for those involved in ion transport. This cluster contains a relatively small fraction of genes with a GO annotation (137/504 for Biological Process), providing an opportunity to study genes with new functions.

Supplementary Table 1. Some salient features of the best *D. plexippus* genome assemblies obtained using different computational pipelines

	**Assembly Identifiers**								
**Feature**	**Canu**	**Falcon**	**Falcon_unzip**	**Wtdbg2**	**Quickmerge**	**Dbg2olc**	**Purge_haplotigs**	**Haplomerger2**	**CanuPiFSC**
Span (Mb)	458.592	451.806	449.886	246.882	254.382	255.261	242.717	252.195	248.571
Contigs									
Number	1158	639	588	607	158	281	187	229	109
Longest (Mb)	16.319	12.04	12.123	13.089	16.344	10.062	16.319	16.344	16.343
N50 (Mb)	1.222	1.863	1.874	4.41	8.064	2.718	2.746	3.097	3.93
BUSCO †									
Complete	97.70%	88.60%	88.50%	93.37%	96.64%	93.70%	96.20%	97.67%	97.79%
Duplicated	63.50%	26.40%	25.90%	0.53%	2.95%	2.50%	2.50%	2.95%	2.70%
Missing	1.40%	4.70%	4.90%	2.29%	1.39%	2.50%	1.70%	1.19%	1.15%

† Using BUSCO v2.0.1 [15] and the Endopterygota_odb9 gene set.

Supplementary Table 2. Mapping-back rates of DNA Illumina sequencing reads

**Type of Average Rate ***	**Dpv3 †**	**DpMex_v1 ‡**
Mapped Reads	94.43%	93.60%
Multimapping reads	12.84%	14.34%
Discordantly mapped reads	4.87%	3.67%

*Across Illumina genomic DNA libraries [12].

† [6,7].

‡ This work.

Supplementary Table 3. Missing BUSCOs across different *D. plexippus* assemblies

DpvMex_v1 / Dpv3 / Dpv4 *	Count
Complete / Complete / Missing	13
Complete / Missing / Complete	9
Complete / Missing / Missing	13
Missing / Complete / Complete	18
Missing / Complete / Missing	11
Missing / Missing / Complete	6
Missing / Missing / Missing	20

* Using BUSCO 4.0.5 and the Lepidoptera_odb10 (n=5286) gene set.

Supplementary Table 4. Repeat content annotation in the DpMex_v1 genome assembly

Repeat Class	Span (Mb)	Genome Fraction (%)
SINE	0	0
LINE	12.4	4.99
LTR Elements	3.52	1.42
DNA Elements	3.33	1.34
Small RNA	0	0
Satellites	0.01	0.01
Simple Repeats	4.33	4.33
Low Complexity	0.71	0.71
Unclassified	18.69	7.52
Total	42.99	17.3

Supplementary Table 5. Lepidopteran and *D. melanogaster* proteomes used

Organism	URL
Drosophila melanogaster	ftp://ftp.flybase.net/genomes/Drosophila_melanogaster/dmel_r6.26_FB2019_01/fasta/dmel-all-translation-r6.26.fasta.gz
Heliconius melpomene	http://download.lepbase.org/v4/sequence/Heliconius_melpomene_melpomene_Hmel2_-_proteins.fa.gz
Bombyx mori	http://sgp.dna.affrc.go.jp/ComprehensiveGeneSet/files/GenesetA.pp.fasta.zip
Melitaea cinxia	http://download.lepbase.org/v4/sequence/Melitaea_cinxia_-_proteins.fa.gz
Plutella xylostella	ftp://ftp.ncbi.nlm.nih.gov/genomes/refseq/invertebrate/Plutella_xylostella/latest_assembly_versions/GCF_000330985.1_DBM_FJ_V1.1/GCF_000330985.1_DBM_FJ_V1.1_translated_cds.faa.gz
Papilio xuthus	ftp://ftp.ncbi.nlm.nih.gov/genomes/all/GCF/000/836/235/GCF_000836235.1_Pxut_1.0/GCF_000836235.1_Pxut_1.0_protein.faa.gz
Amyelois transitella	ftp://ftp.ncbi.nlm.nih.gov/genomes/all/GCF/001/186/105/GCF_001186105.1_ASM118610v1/GCF_001186105.1_ASM118610v1_protein.faa.gz

Supplementary Table 6. Contig and gene anchoring to the *D. plexippus* chromosomes

Metric	Number
One-to-one orthologs between *D. plexippus* and *M. cinxia*	7,347
One-to-one orthologs mapped onto *M. cinxia* chromosomes	5,004 (68.1%) / 4,855 (97.0% *)
Contigs mapped directly onto *D. plexippus* chromosomes by harboring one-to-one orthologs	74 (68.52%)
Contigs mapped indirectly onto *D. plexippus* chromosomes ¦	6
Protein-coding genes mapped onto *D. plexippus* chromosomes	15,815 (98.9% †)
lncRNA genes mapped onto *D. plexippus* chromosomes	599 (95.8% †)

* Those that support preferential mapping onto a particular *M. cinxia* chromosome relative to the total mapped onto chromosomes.

¦ Via their merging with other directly mapped contigs.

† Relative to the particular class of genes considered.

Supplementary Table 7. Salient features of differential expression contrasts (FDR < 0.05)

Contrast ID	Total (Up + Down)	lncRNA Only (Up +Down)	Compared Samples	Source
L1:L	1422 (863 + 559)	52 (20 + 32)	L1 vs L3+L5	Langebio
L3:L	1194 (595 + 599)	21 (8 + 13)	L3 vs L1+L5	Langebio
L5:L	1420 (999 + 421)	46 (45 + 1)	L5 vs L1+L3	Langebio
Lpool:Other	3038 (1377 + 1661)	95 (34 + 61)	Lpool vs Ppool+Fpool+Mpool	UCI
P1:L5	3492 (1366 + 2126)	83 (29 + 54)	P1 vs L5	Langebio
P1:P	1772 (716 + 1056)	59 (32 + 27)	P1 vs P3+P5+P7+P9	Langebio
P3:P	1203 (323 + 880)	23 (8 + 15)	P3 vs P1+P5+P7+P9	Langebio
P5:P	971 (667 + 304)	17 (12 + 5)	P5 vs P1+P3+P7+P9	Langebio
P7:P	1035 (887 + 148)	31 (28 + 3)	P7 vs P1+P3+P5+P9	Langebio
P9:P	2626 (1967 + 659)	87 (74 + 13)	P9 vs P1+P3+P5+P7	Langebio
Ppool:Other	3206 (2003 + 1203)	89 (60 + 29)	Ppool vs Lpool+Fpool+Mpool	UCI
FT:AdultParts	2616 (1621 + 995)	70 (52 + 18)	FT vs MT+FH+MH+FA+MA	Langebio
MT:AdultParts	2429 (1694 + 735)	87 (61 + 26)	MT vs FT+FH+MH+FA+MA	Langebio
FMH:AdultParts	2689 (1233 + 1456)	62 (41 + 21)	FH+MH vs FT+MT+FA+MA	Langebio
FMA:AdultParts	2614 (1644 + 970)	95 (56 + 39)	FA+MA vs FT+MT+FH+MH	Langebio
FT:MT	672 (243 + 429)	21 (9 + 12)	FT vs MT	Langebio
FH:MH	86 (42 + 44)	6 (1 + 5)	FH vs MH	Langebio
FA:MA	57 (38 + 19)	8 (6 + 2)	FA vs MA	Langebio
L:Other	2607 (1654 + 953)	75 (39 + 36)	L1-5 vs P1-9+ FMT+FMH+FMA	Langebio
P:Other	2156 (1162 + 994)	61 (42 + 19)	P1-9 vs L1-5+ FMT+FMH+FMA	Langebio
AdultParts:Other	2287 (1151 + 1136)	60 (43 + 17)	FMT+FMH+FMA vs L1-5+P1-9	Langebio
Sexes	1809 (245 + 1564)	101 (20* + 81*)	Fpool vs Mpool	UCI
Adult:Other	2473 (1245 + 1228)	104 (71* + 33)	Fpool+Mpool vs Lpool+Ppool	UCI
Source †	2574 (2489 + 85)	226 (225* + 1)	Lpool+Ppool+Fpool+Mpool vs L1-5+P1-9+FMT+FMH+FMA	UCI & Langebio

* Significantly higher than random expectation at P_{adj}<0.05 (one-tailed Fisher's exact test, with Bonferroni correction).
† Technical contrast in which the step of library preparation for transcriptome sequencing of the pooled samples at UCI was purposely enriched for lncRNAs by minimizing loss of non-polyadenylated transcripts.

L, larva; P, pupa; F, female; M, male; T, thorax; A, abdomen; H, head. AdultParts=FT, MT, FH, MH, FA, MA.

Supplementary Table 8. Salient features of WGCNA clusters

Cluster	All Genes	lncRNAs	Differentially Expressed Genes	Jaccard Overlap with DE > 10%
clust01	2374	103	1740	Sexes.down(42.5%)|Ppool:Other.up(29.7%)|Source.up(25.3%)|Lpool:Other.down(19.1%)|MT:AdultParts.up(11.6%)
clust02	2046	49	889	P1:L5.up(24.4%)|P:Other.up(11.5%)
clust03	2022	55	1341	FMH:AdultParts.up(41.2%)|P:Other.down(18.4%)|P1:L5.down(17.2%)|P9:P.up(15.9%)|AdultParts:Other.up(14.8%)|FT:AdultParts.down(11.3%)|MT:AdultParts.down(10.4%)
clust04	1926	64	1444	FMA:AdultParts.up(50.5%)|L1:L.up(27.2%)|FMH:AdultParts.down(20.4%)|FT:AdultParts.down(16.7%)|Adult:Other.up(13.3%)|Ppool:Other.down(13.1%)|MT:AdultParts.down(10.6%)
clust05	944	20	731	Lpool:Other.up(36.3%)|L:Other.up(30.9%)|P1:L5.down(21.6%)|Adult:Other.down(18.7%)|L5:L.up(12.8%)|L3:L.down(11.3%)|Ppool:Other.down(11.1%)
clust06	644	7	238	
clust07	633	8	307	Lpool:Other.up(12%)|L:Other.up(10.8%)
clust08	519	13	386	FT:AdultParts.up(17.6%)|MT:AdultParts.up(15.6%)|AdultParts:Other.down(14.1%)|FMA:AdultParts.down(13.1%)
clust09	504	21	340	AdultParts:Other.up(18%)|L:Other.down(12.9%)|Adult:Other.up(11.3%)
clust10	429	45*	360	Sexes.down(18.3%)|FT:MT.down(12.6%)
clust11	390	8	341	FT:AdultParts.up(16.4%)|L:Other.up(14.8%)|MT:AdultParts.up(13%)|P1:L5.down(12.4%)|Ppool:Other.down(11.2%)|Lpool:Other.up(10.5%)
clust12	384	9	131	
clust13	318	10	284	P5:P.up(20.9%)|P3:P.down(16.5%)|P1:P.down(14.9%)|P7:P.up(12%)
clust14	265	12	173	
clust15	215	2	35	
clust16	212	2	147	L3:L.up(15.8%)|P3:P.up(11%)
clust17	187	6	161	P5:P.up(21.1%)
clust18	172	5	149	
clust19	147	11	124	Sexes.up(25.6%)
clust20	109	4	105	Sexes.up(11.7%)|P7:P.up(10.4%)
clust21	80	5	77	
clust22	66	4	48	
clust23	65	5	55	
clust24	64	2	46	
clust25	43	0	36	
clust26	37	19*	31	

Supplementary Table 8. Salient features of WGCNA clusters

Cluster	All Genes	lncRNAs	Differentially Expressed Genes	Jaccard Overlap with DE > 10%
clust27	26	1	26	
TOTAL	14821	490	9745	

* Significantly greater than the random expectation at *Padj*<0.05 (one-tailed Fisher's exact test, with Bonferroni correction).

Supplementary Table 9. Chromosomal distribution of sex and non-sex-biased genes in adult samples of *D. plexippus*

Expression Threshold *	Expression Bias †	Whole-Body			Thorax			Head			Abdomen		
		anc-*Z*	neo-*Z*	A	anc-*Z*	neo-*Z*	A	anc-*Z*	neo-*Z*	A	anc-*Z*	neo-*Z*	A
>0.01	M	123	38	1403	28	9	392	0	0	44	1	2	15
	U	485	459	11992	574	484	12985	607	500	13509	608	499	13534
	F	1	5	191	7	9	209	2	2	33	0	1	37
		Padj = 4.33×10-5			*Padj* = 0.072			na			na		
>0.1	M	123	38	1402	28	9	392	0	0	44	1	2	15
	U	485	459	11978	569	483	12922	580	491	13202	588	494	13300
	F	1	5	191	7	9	208	2	2	33	0	1	37
		Padj = 4.33×10-5			*Padj* = 0.072			na			na		
>0.5	M	122	38	1373	25	9	379	0	0	42	1	2	13
	U	469	446	11432	521	463	11992	433	412	10835	484	428	11351
	F	1	5	184	4	8	197	2	2	31	0	1	37
		Padj = 4.33×10-5			*Padj* = 0.072			na			na		
>1	M	116	33	1334	24	9	356	0	0	42	1	2	13
	U	454	433	10952	494	445	11302	390	378	9792	446	406	10545
	F	1	4	177	4	7	185	2	2	24	0	1	36
		Padj = 7.83×10-5			*Padj* = 0.072			na			na		

P_{adj} refers to the probability of obtaining the observed distribution of male- and female-biased genes across the anc-*Z*, neo-*Z*, and autosomes by just chance alone according to a two-tailed Fisher's exact test and applying the multitest Bonferroni correction. Green and red denote significant enrichment and depletion, respectively, according to the analysis of the adjusted standardized residuals using 1.96 and -1.96 as thresholds for significance [16]. na, statistical analysis not performed due to very limited number of sex-biased genes.

* FPKM.

† M, male-biased; U, unbiased; F, female-biased.

Supplementary Table 10. Chromosomal distribution of sex and non-sex-biased genes in whole-body samples of *D. plexippus*

Expression Threshold *	Expression Bias †	Chromosome Fractions			Contrast	
		anc-*Z*	neo-*Z*	A	anc-*Z* : neo-*Z* : A	neo-*Z* : A
**>0.01**	S	124	43	1594	$P_{adj} = 1.57\times10^{-9}$	$P_{adj} = 0.0324$
	U	485	459	11992		
**>0.1**	S	124	43	1593	$P_{adj} = 1.57\times10^{-9}$	$P_{adj} = 0.0324$
	U	485	459	11978		
**>0.5**	S	123	43	1557	$P_{adj} = 1.57\times10^{-9}$	$P_{adj} = 0.0324$
	U	469	446	11432		
**>1**	S	117	37	1511	$P_{adj} = 1.62\times10^{-9}$	$P_{adj} = 0.0185$
	U	454	433	10952		

P_{adj} refers to the probability of obtaining the observed distribution of sex-biased and unbiased genes across chromosomal fractions by chance alone according to a two-tailed Fisher's exact test and applying the multitest Bonferroni correction. Green and red denote significant enrichment and depletion, respectively, according to the analysis of the adjusted standardized residuals using 1.96 and -1.96 as thresholds for significance [16]. Differential color intensity reflects the rank of the magnitude for enrichment or depletion as shown by the value of the corresponding residual. na, statistical analysis not performed due to very limited number of sex-biased genes.

* FPKM.

† S, sex-biased; U, unbiased.

Supplementary Table 11. Expression metrics for protein-coding and lncRNAs across broadly defined developmental stages

Stage	Type	n	Mean	SD	Min	Q1	Median	Q3	Max
**L1+L3+L5 ***	Coding	13970	594.323	3807.89	0.167	16.167	93.167	295.625	246819.5
	lncRNA	417	434.833	4104.175	0.167	1.5	5.667	21.667	79347.83
	Kruskal-Wallis, Chi-square = 351.19, *P* < 2.2 x 10-16								
**P1+P3+P5+P9 ***	Coding	14256	671.263	3624.835	0.1	34.6	154.6	428.075	216516.6
	lncRNA	480	260.914	2554.722	0.1	2.8	10.45	40.025	53657.9
	Kruskal-Wallis, Chi-square = 458.79, *P* < 2.2 x 10-16								
**FT+FH+FA ***	Coding	13832	703.26	4109.26	0.167	20.5	123	368.542	182935.5
	lncRNA	431	459.196	2920.351	0.167	2.167	9.667	43.75	47706.5
	Kruskal-Wallis, Chi-square = 272.23, *P* < 2.2 x 10-16								
**MT+MH+MA ***	Coding	14117	610.543	3208.695	0.167	34	131.833	358.833	132258.3
	lncRNA	478	365.432	2678.202	0.167	4.167	11.417	46.75	51355.83
	Kruskal-Wallis, Chi-square = 377.49, *P* < 2.2 x 10-16								
**Lpool †**	Coding	14353	1004.2	5708.463	0	33.5	189	593	330385.5
	lncRNA	492	1742.532	13347.99	0	4.375	28.75	119.625	170086
	Kruskal-Wallis, Chi-square = 176.46, *P* < 2.2 x 10-16								
**Ppool †**	Coding	14160	771.859	5046.912	0.5	48.5	199.5	522.5	329200.5
	lncRNA	478	1206.918	9984.424	0.5	9.125	35.5	134.375	180101
	Kruskal-Wallis, Chi-square = 229.16, *P* < 2.2 x 10-16								
**Fpool †**	Coding	13900	1117.682	5840.344	0.5	49	251	725.125	375029
	lncRNA	446	1238.934	7144.161	0.5	16.125	75.5	247.75	122219.5
	Kruskal-Wallis, Chi-square = 104.69, *P* < 2.2 x 10-1								
**Mpool †**	Coding	14158	1372.185	6182.735	0.5	106	368	956.5	295733.5
	lncRNA	480	1107.954	6114.932	0.5	28.375	90	284.875	104848.5

Supplementary Table 11. Expression metrics for protein-coding and lncRNAs across broadly defined developmental stages

Stage	Type	n	Mean	SD	Min	Q1	Median	Q3	Max
	Kruskal-Wallis, Chi-square = 185.56, *P* < 2.2 x 10-16								

Source sequencing sample: * Langebio; † UCI.

For each stage considered, the average expression across the pertinent samples was calculated.

L, larva; P, pupa; F, female; M, male; T, thorax; A, abdomen; H, head. Adult parts: FT, MT, FH, MH, FA, MA.

Padj upon Bonferroni multitest correction.

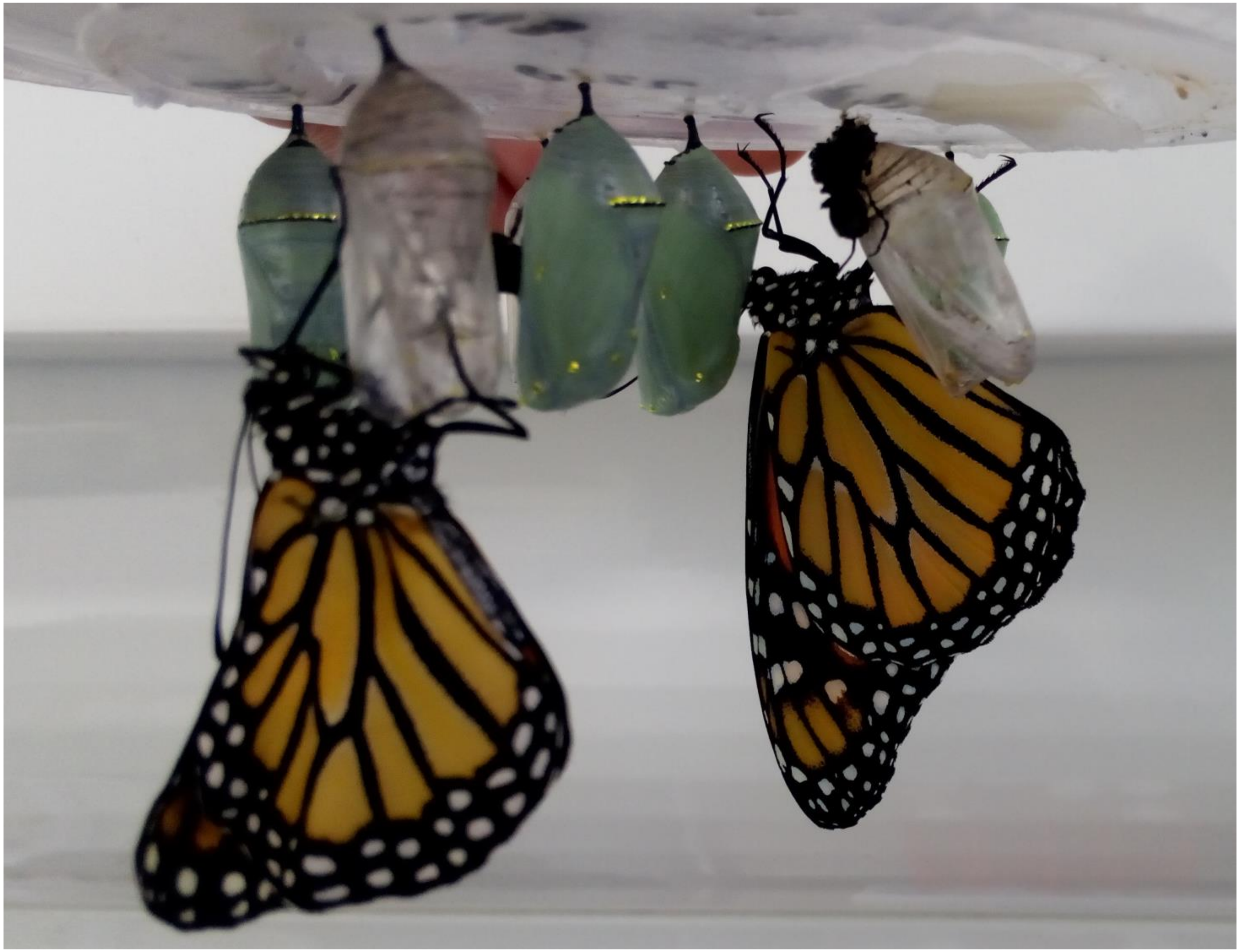

Supplementary Figure 1. **Pupa and adult individuals of the monarch butterfly *D. plexippus***. In nature, *D. plexippus* feeds on a host milkweed plant from the genus *Asclepias*.

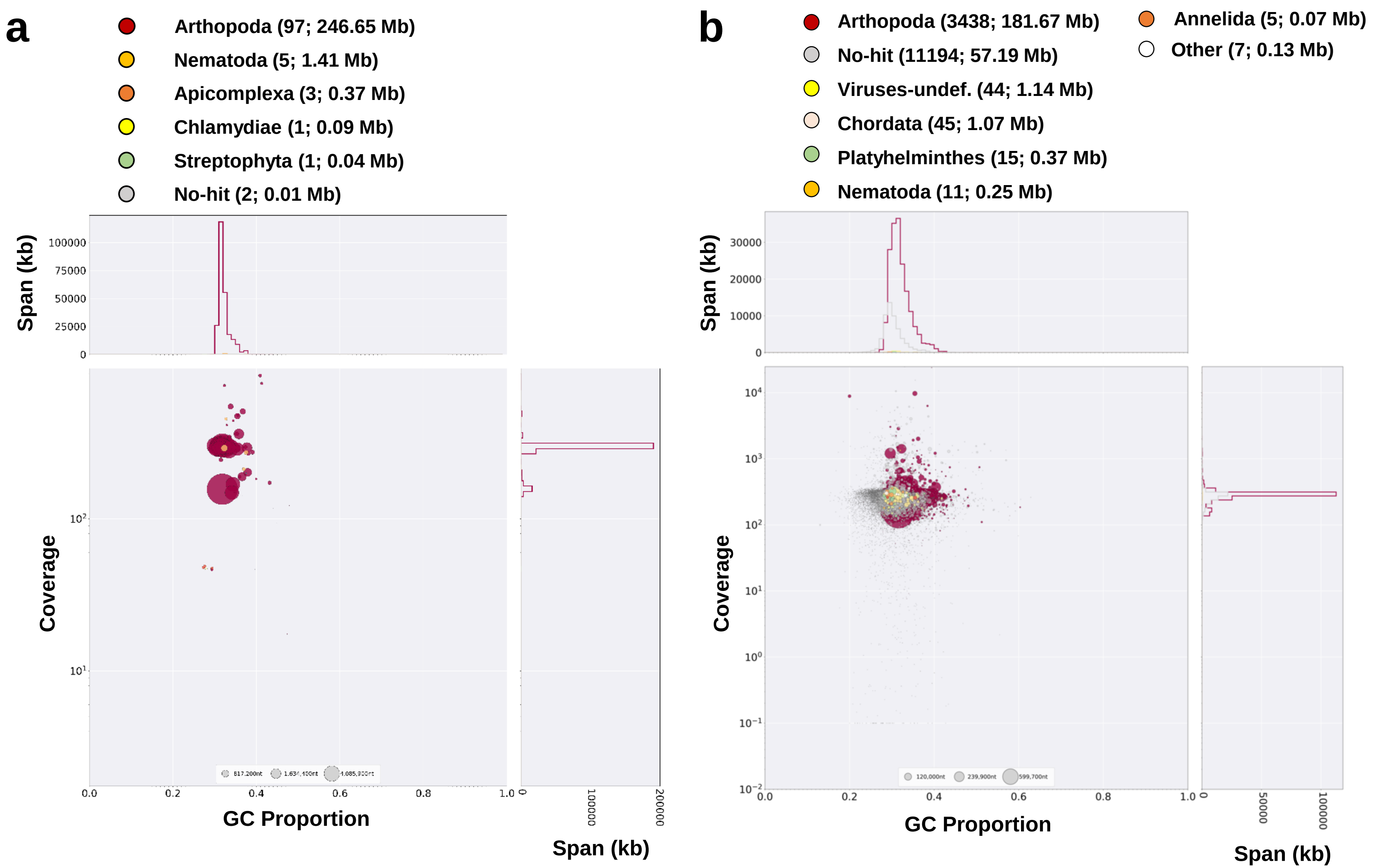

Supplementary Figure 2. Taxon-Annotated GC-Coverage plot for different assemblies of *D. plexippus*. **a** DpMex_v1, this work. **b** *D. plexippus* v3 [6]. In both cases, the lower left panel shows a contig scatterplot according to GC content (x-axis) and sequence coverage (y-axis) according to a color code that informs about the best match of each contig taxonomically annotated sequences databases (legend). The circle size is proportional to the contig span. The upper panel shows the distribution of the total span for a set of contigs related to a given taxonomically relevant group based on their GC content. Contigs with significant similarity against arthropod-related sequences tend to group to the right of the scatterplot, *i.e.* they exhibit higher GC content values. The right panel shows the distribution of the span of contigs for a given taxonomically relevant group based on their coverage. Top, breakdown of the taxonomic preferential association of the contigs in the assembly under examination (number of contigs; total contig span). Bobplots were generated using Blobtools v.1.0 [1].

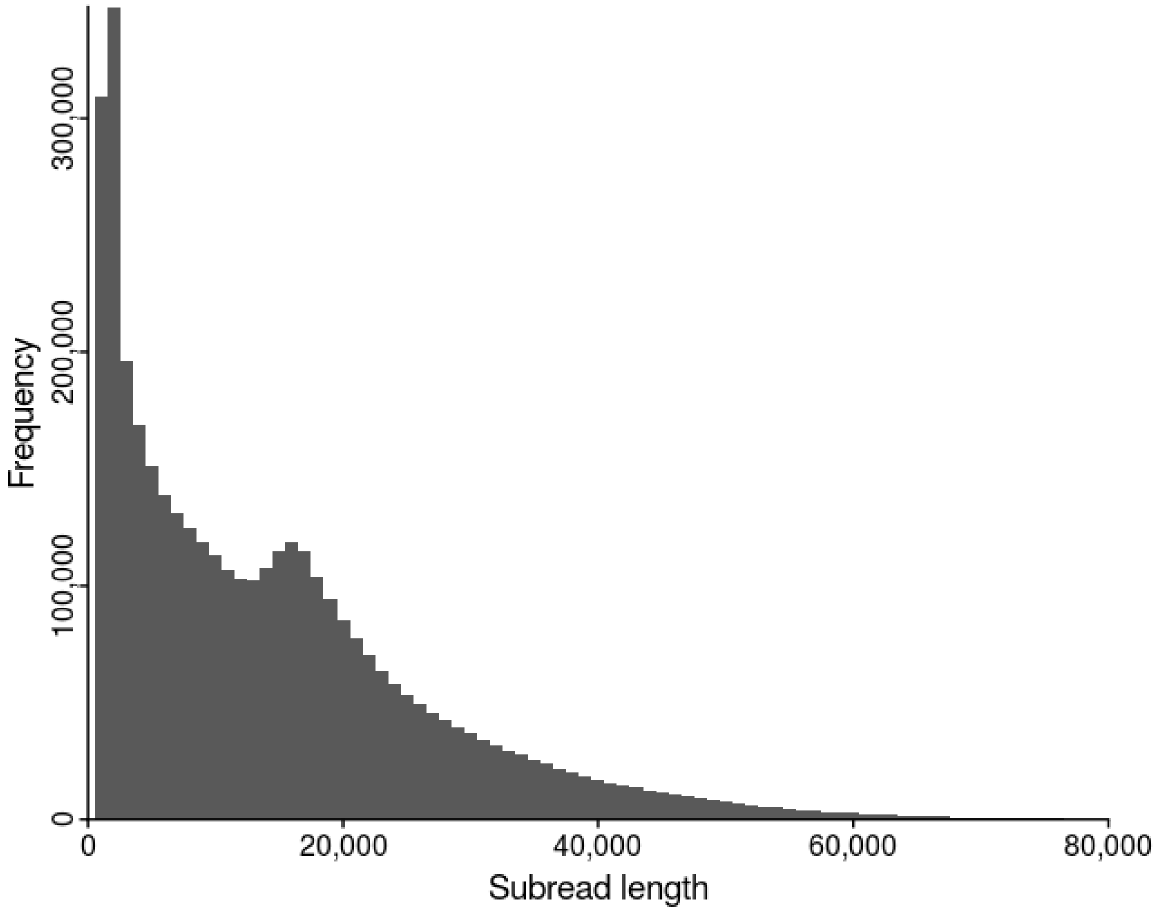

Supplementary Figure 3. Length distribution of PacBio reads above 1 kb. For the dataset generated, NR50 = 22.561 kb; NR50 refers the median read length above which half of the total coverage is contained.

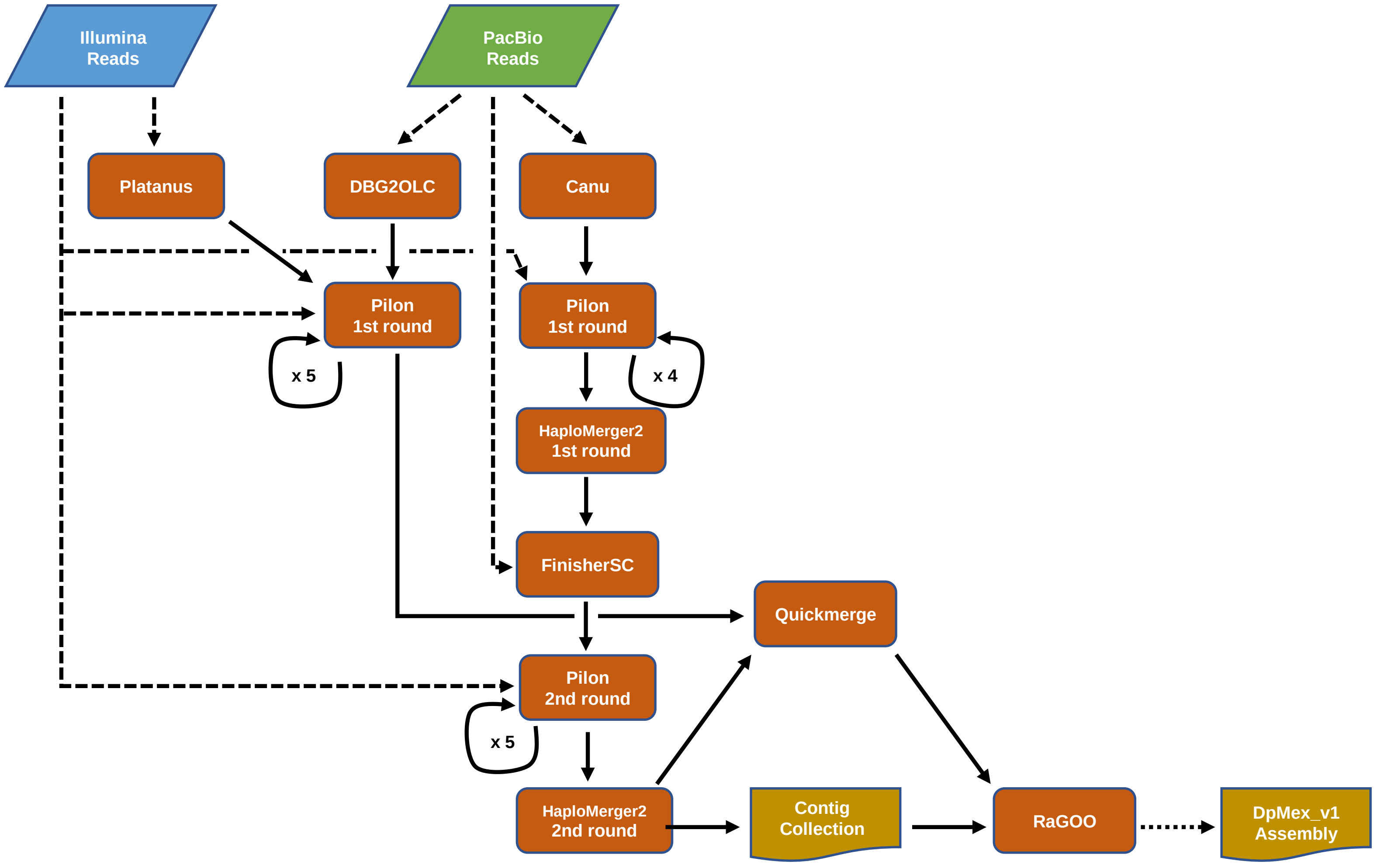

Supplementary Figure 4. Computational strategy to obtain a reference-quality *de novo* genome assembly for *D. plexippus*. Solid lines, programs used in different steps. Dashed lines, sequence information used to feed particular programs. Dotted lines, relevant assembly generated. The final polished assembly DpMex_v1 was subsequently repeat-masked and used in different downstream analyses. The symbol x indicates the number of iterations in the use of particular programs. Additional or alternative steps in the pipeline that led to alternative assemblies either less complete, less contiguous, or both, are not shown.

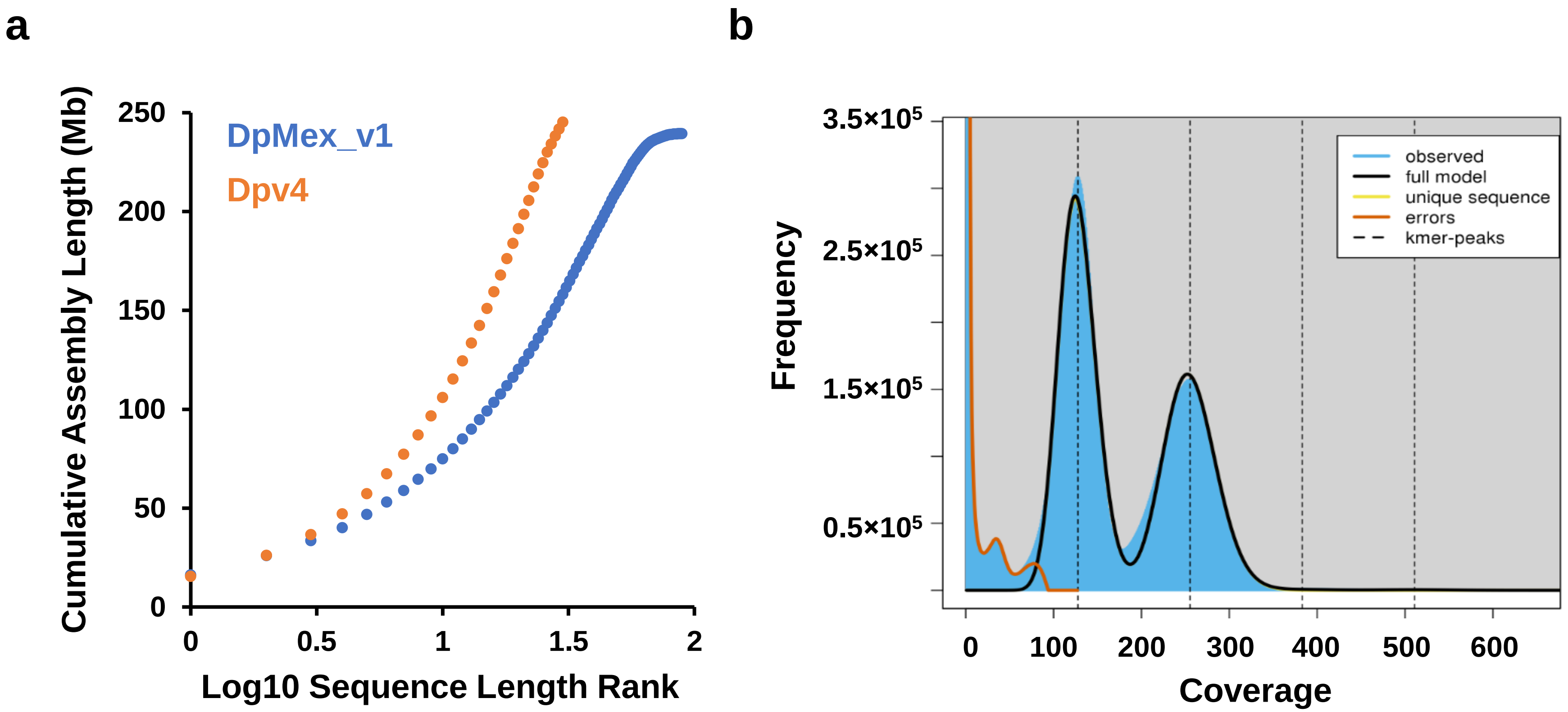

Supplementary Figure 5. Salient features of the new genome assembly of *D. plexippus*. **a** Contiguity sequence length distribution of the assemblies Dpv3 [6] and DpMex_v1, this work. x-axis, log10 of rank of the contig (or scaffold) based on their sequence length; y-axis, cumulative sequence length to the rank on the x-axis. In both cases, the assembly was derived from the female genome. Scaffolds and contigs are used for Dpv3 and DpMex_v1, respectively. An optimal assembly should appear as an almost straight line. Plateaus denote collections of small scaffolds (or contigs). **b** K-mer spectra, *i.e.* the K-mer frequency distribution, for the Illumina PE-150 sequencing output generated in this work. Only filtered sequence data were used. Two peaks at ~133x and ~265x can be distinguished according to GenomeScope.

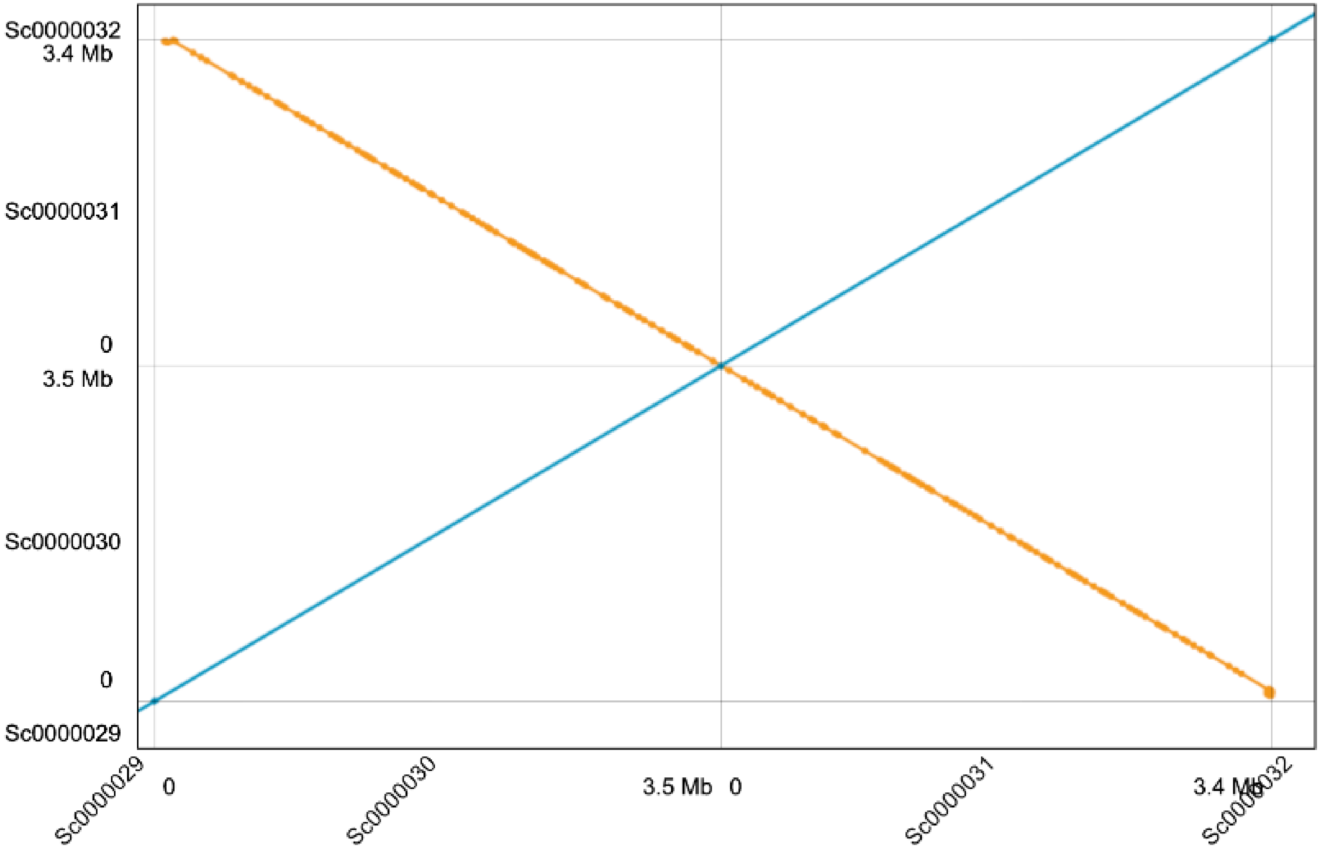

Supplementary Figure 6. Alignment dot plot between two haplotypic contigs. Contigs Sc0000030 and Sc0000031 harbor 42 BUSCOs duplicates, the largest number identified between any two contigs; the pair of contigs with the second highest number of BUSCOs duplicates (7) corresponds to contigs Sc0000002 and Sc0000040. The perfect collinearity shown by the contigs Sc0000030 and Sc0000031 in the dot plot is consistent with the high number of BUSCO duplicates that they share. No other pair of contigs showed this pattern. Dot plots were generated with MUMmer.

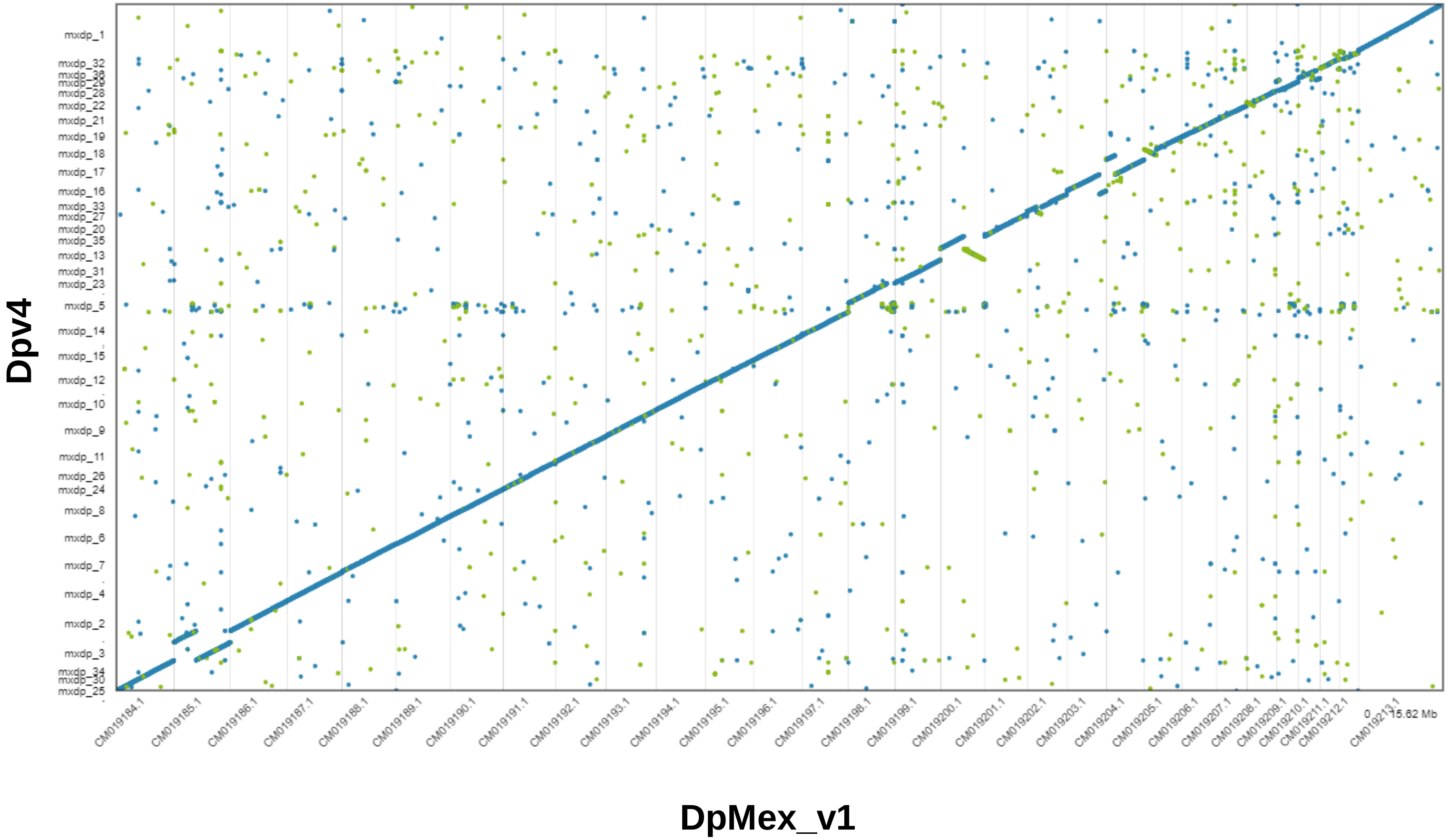

Supplementary Figure 7. **Alignment dot plot between the DpMex_v1 and Dpv4 assemblies.** Small off-diagonal dots typically correspond to differential local repeat composition. Blue, equal orientation; green, reversed orientation. Only scaffolds from contigs mapped to chromosomes were included in the genome-genome alignment. The dot plot was generated with Dot (https://github.com/dnanexus/dot; last accessed May 28, 2020).

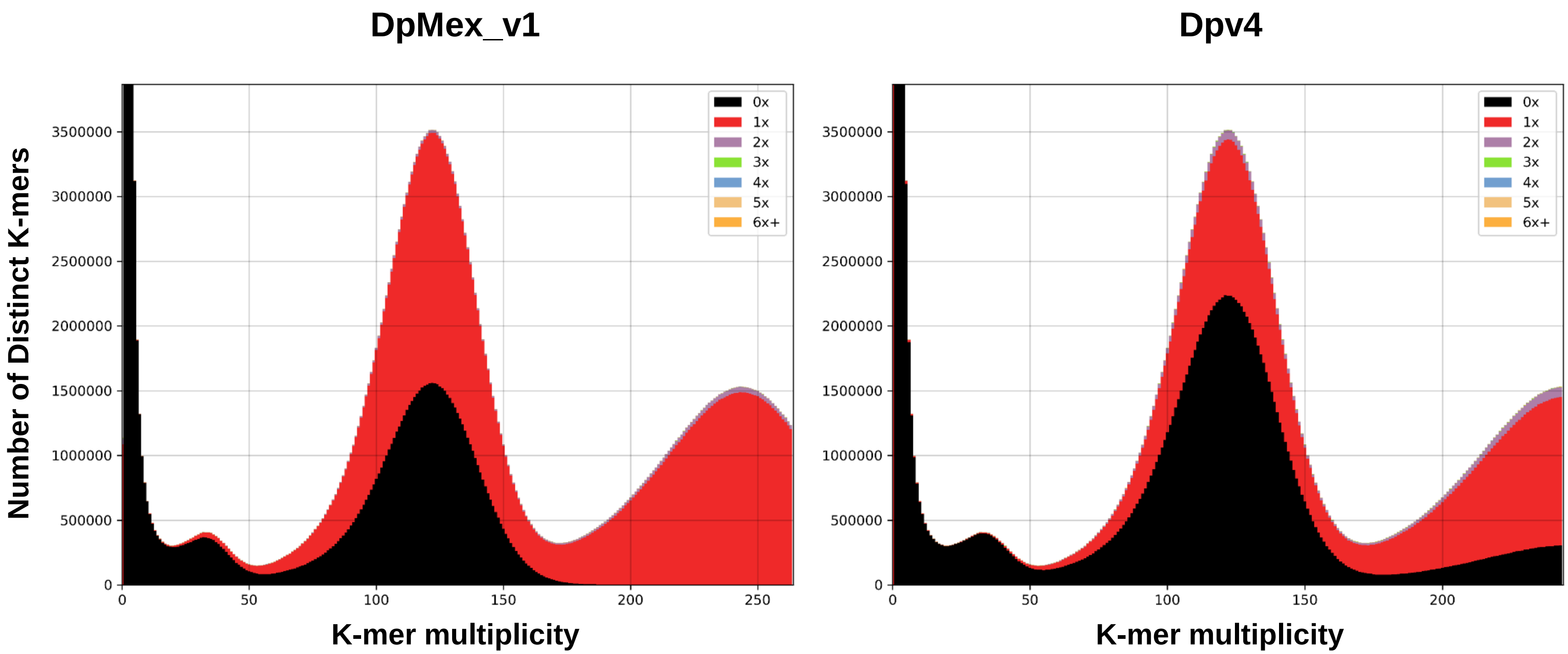

Supplementary Figure 8. K-mer comparison plots for two assemblies of *D. plexippus*. Dpmex_v1, this work; Dpv4, Dpv3 scaffolded and corrected with Hi-C data [7]. Two k-mers multiplicity peaks are typically seen in sequencing reads of heterozygous samples, as it is the case for the sample presented in this work, with the peak on the left representing the heterozygous content and the one on the right representing the homozygous content. The black area depicts the amount of k-mers found in the sequencing reads but not found in the assembly. The amount of missing k-mers, both for the heterozygous and homozygous contents, is higher in the Dpv4 compared to DpMex_v1 assembly. K-mer spectra visualization was performed with KAT v2.4.1.

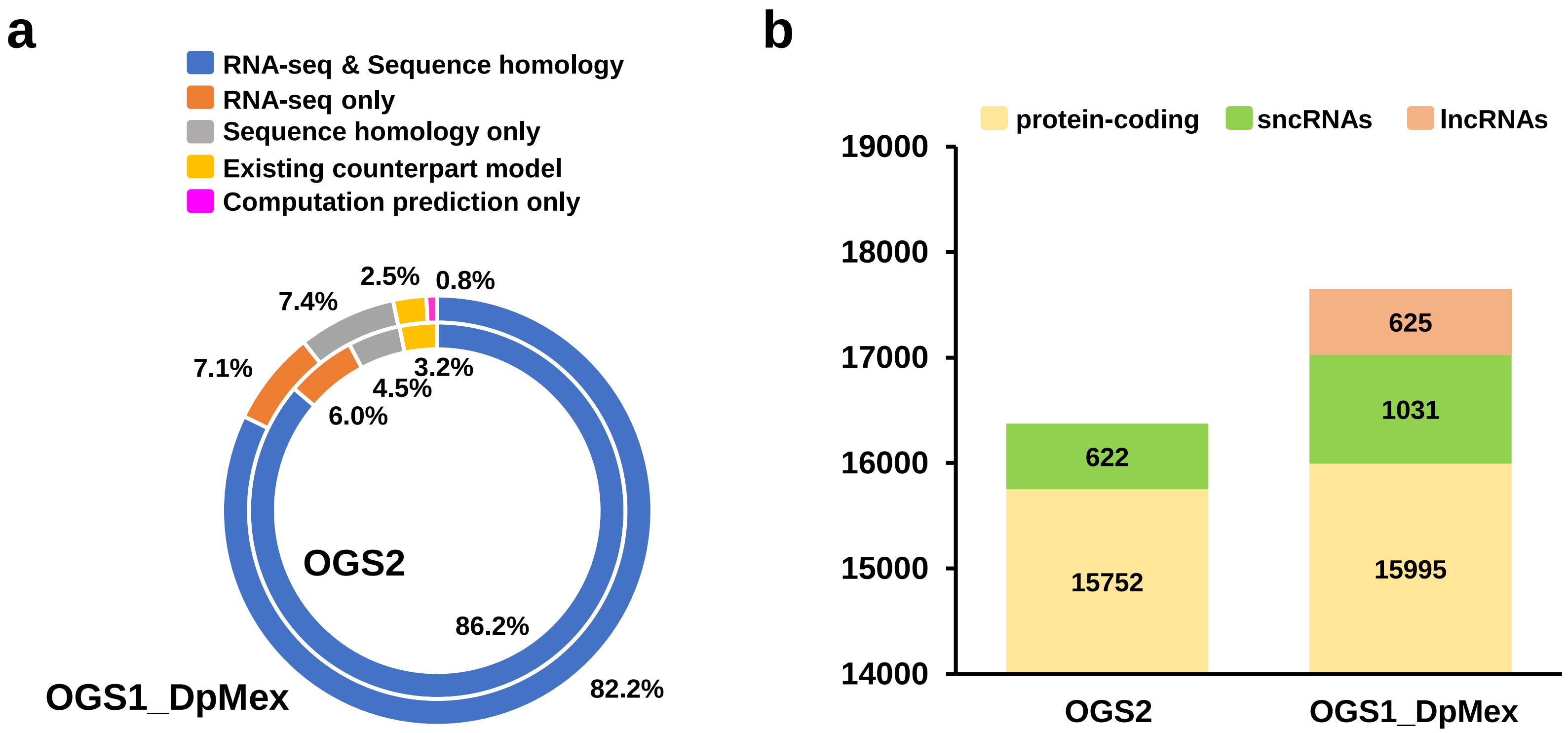

Supplementary Figure 9. Salient features of the new gene annotation of *D. plexippus*. a Doughnut pie charts showing the percentage of protein-coding predicted gene models supported by different type of evidence in the OGS2 and OGS1_Dpmex annotations. **b** Gene type composition of the OGS2 and OGS1_Dpmex annotations. The new annotation, OGS1_Dpmex, not only includes 1,793 additional gene models, but they also contribute to all gene categories, particularly to that of lncRNAs, which were not considered in OGS2.

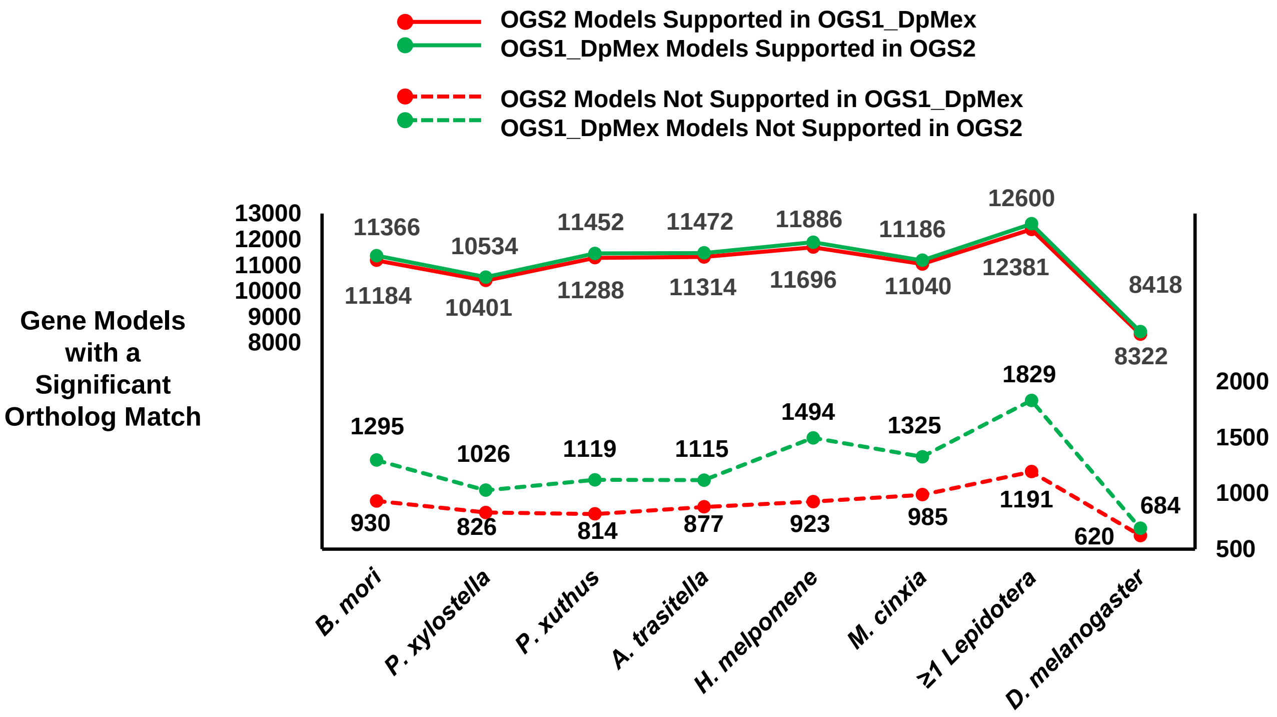

Supplementary Figure 10. Orthology calls for models of protein-coding genes from the OGS2 and OGS1_DpMex annotations across six Lepidoptera species and *D. melanogaster*. The number of orthology calls is partitioned for the gene models found in OGS1_DpMex and OGS2, which corresponds to 14,318 and 14,005 respectively as some might differ in the number of copies between assemblies. Similarly, the numbers of gene models with orthology calls is shown for those present in OGS1_DpMex but not in OGS2 (1,677 or 10.5%) and for those showing the opposite trend (1,077 or 7.1%). When we compared the absolute number of gene models from both assemblies for which it was possible to find at least one homolog with OrthoFinder, a higher number of matches for the OGS1_DpMex gene models regardless of the species is found, reaching a maximum difference in the case of *H. melpomene* with 585 additional gene models in OGS1_DpMex relative to OGS2.

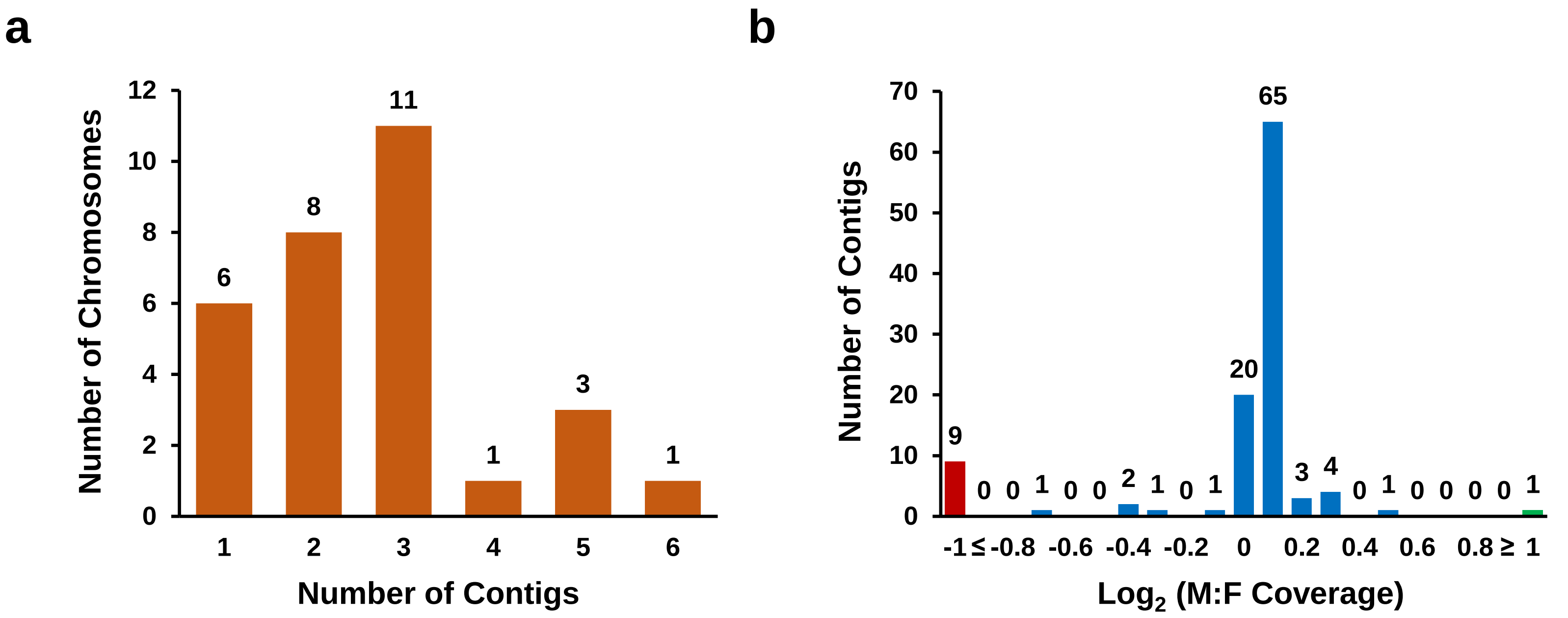

Supplementary Figure 11. Additional features of the DpMex_v1 assembly assignation to chromosomes. a Number of chromosomes plotted against the number of constituent contigs. The chromosomes of *D. plexippus* contain from 1-6 contigs, with 25 chromosomes of *D. plexippus* having associated 1-3 contigs. Chromosomes *1 (i.e.* the heterochromosome *Z*), *15*, *22*, *23*, and *30* are also associated with a single contig and scaffold. **b** Histogram showing the number of contigs in relation to their $\log_2$ (M:F) coverage. The most external bins collapse all those contigs showing the most extreme coverage values, *i.e.* ≤-1 and ≥1.

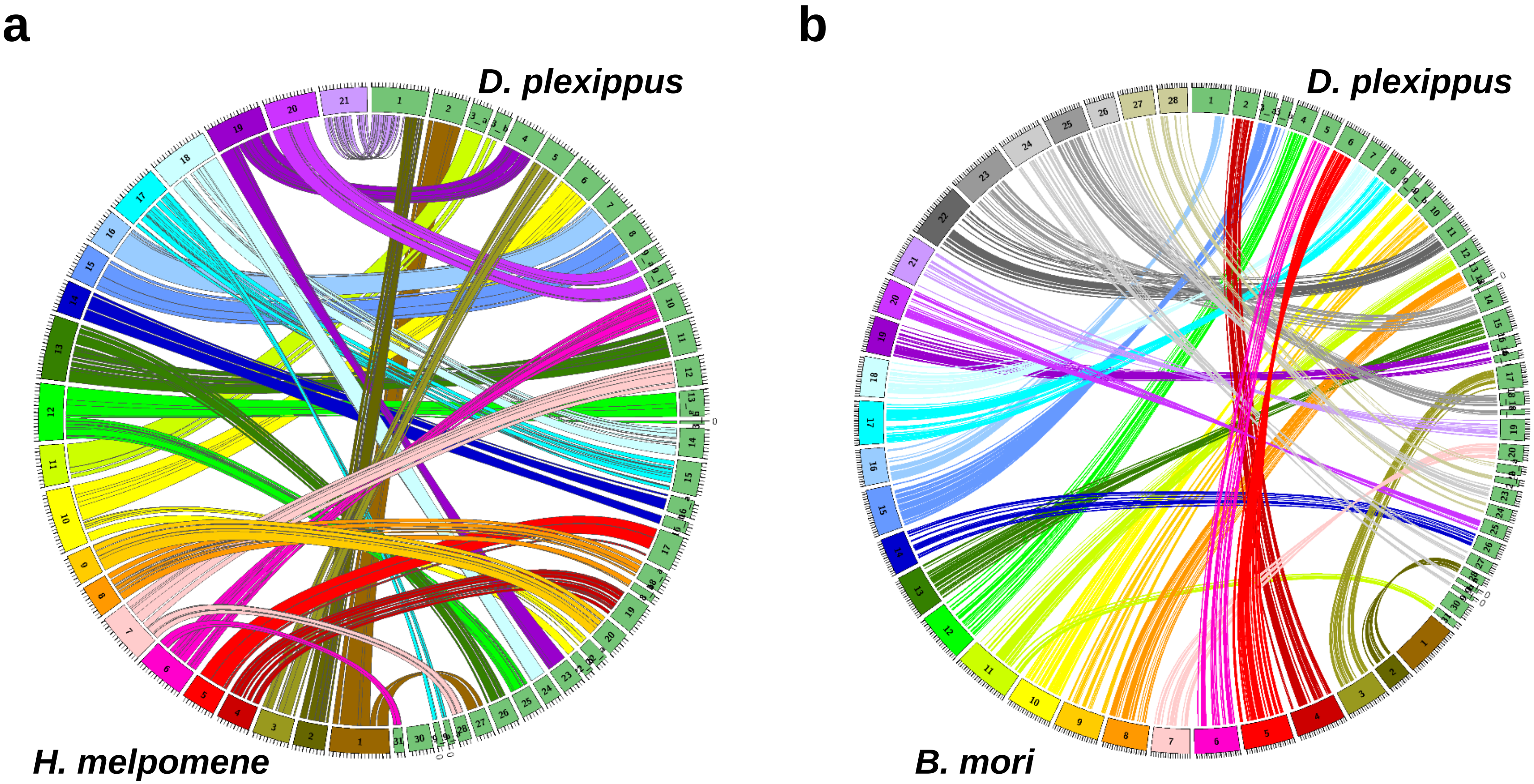

Supplementary Figure 12. Chromosomal mapping of *D. plexippus* to *H. melpomene* and *B. mori*. a-b 5,607 and 6,928 1:1 orthologs found in microsynteny blocks connect the chromosomes of *D. plexippus* to those of *H. melpomene* and *B. mori,* respectively. The chromosomes of *D. plexippus* are numbered according to their relationship with those of *M. cinxia,* except for chromosome *1, i.e.* the heterochromosome *Z*, which is the result of a fusion between chromosomes *1* and *21* of *M. cinxia.* The relationships of gene content across chromosomes are consistent with the fusions in the lineage of *H. melpomene* of the following *M. cinxia* chromosomes: *2* and *27*; *4* and *23*; *6* and *10*; *10* and *31*; *11* and *26*; *12* and *28*; *13* and *25*; *14* and *24*; and *15* and *29*. Similarly in the case of lineage of *B. mori*, the patterns documented are consistent with the fusions of the chromosomes: *12* and *31*; and *27* and *29*. Further, most genes within chromosome *30* of *D. plexippus* are part of multiparalog groups, which map to chromosomes *20* and *23* of *H. melpomene* and *B. mori*, respectively.

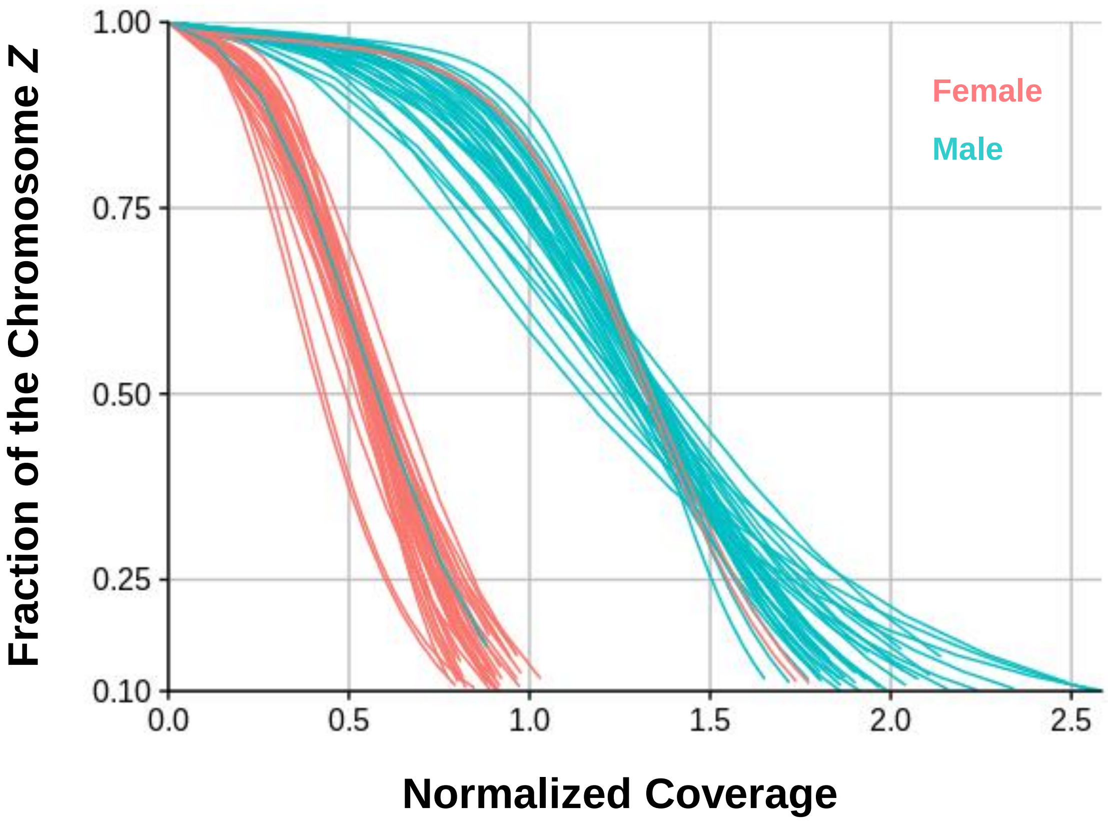

Supplementary Figure 13. Fraction of heterochromosome *Z* covered by a given normalized coverage value for 80 initially considered DNA sequencing samples. These samples were retrieved from Zhan et al. [12]. Ultimately, only 70 samples fulfilled the necessary requirements to be used in the analysis. Male and female samples are color-coded.

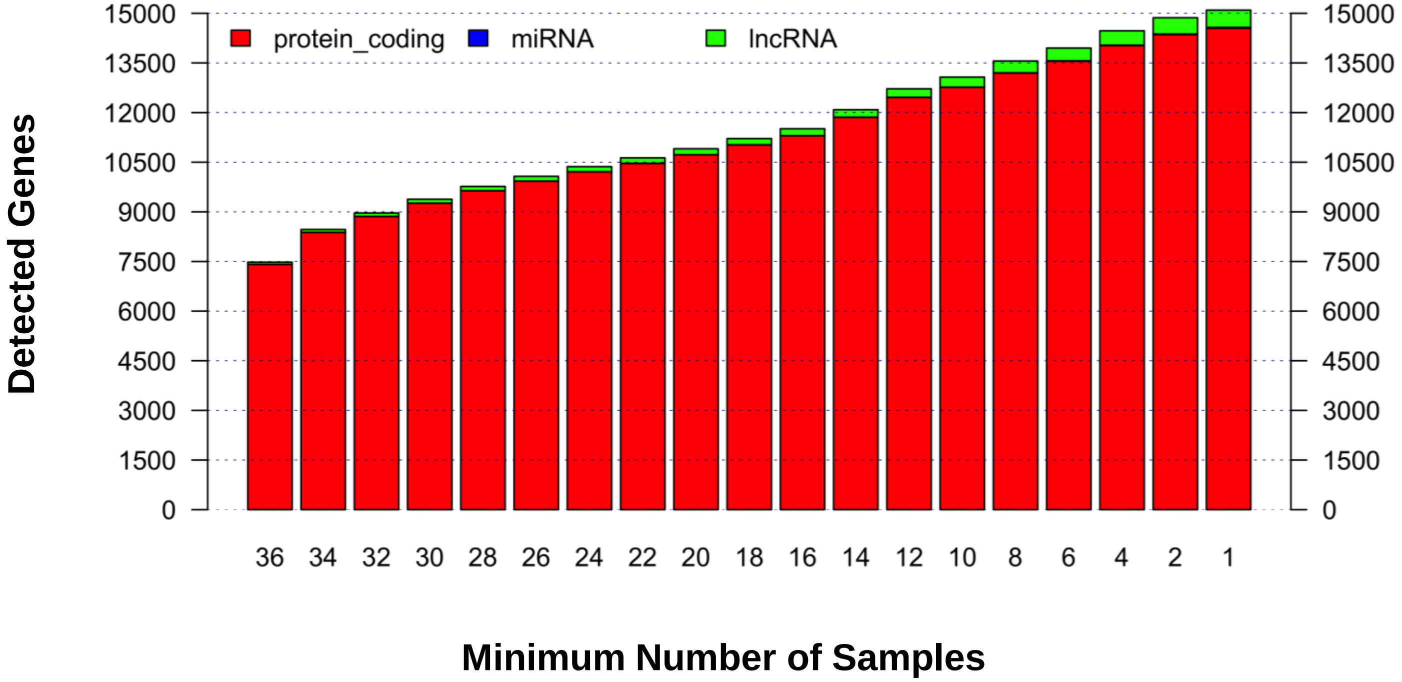

Supplementary Figure 14. Number of genes found expressed across a decreasing number of samples. Genes are deemed as showing evidence of biological meaningful expression with ≥1 count-per-million (CPM) in at least the number of samples shown on the x-axis.

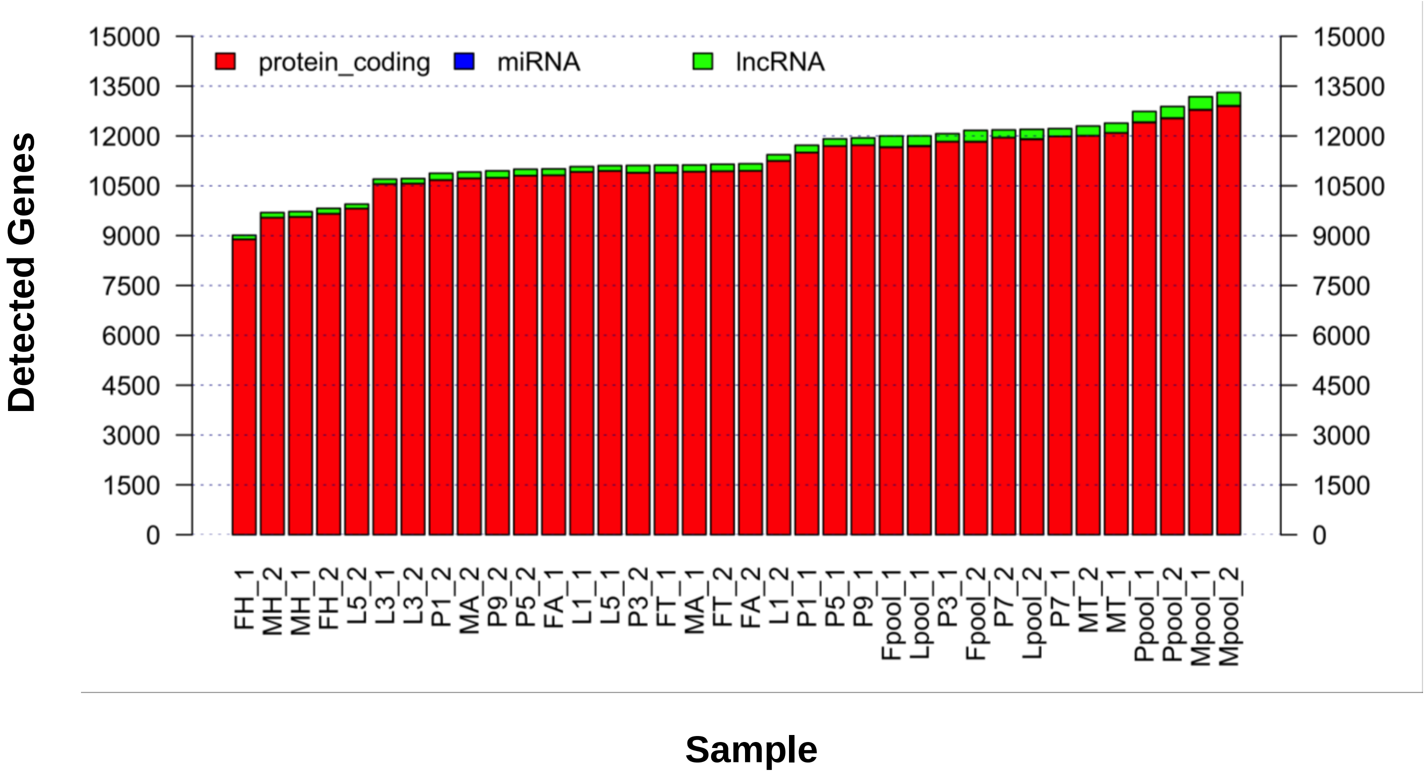

Supplementary Figure 15. Number of genes expressed in each sample. Genes are counted as expressed with ≥1 count-per-million (CPM) in the sample shown on the x-axis.

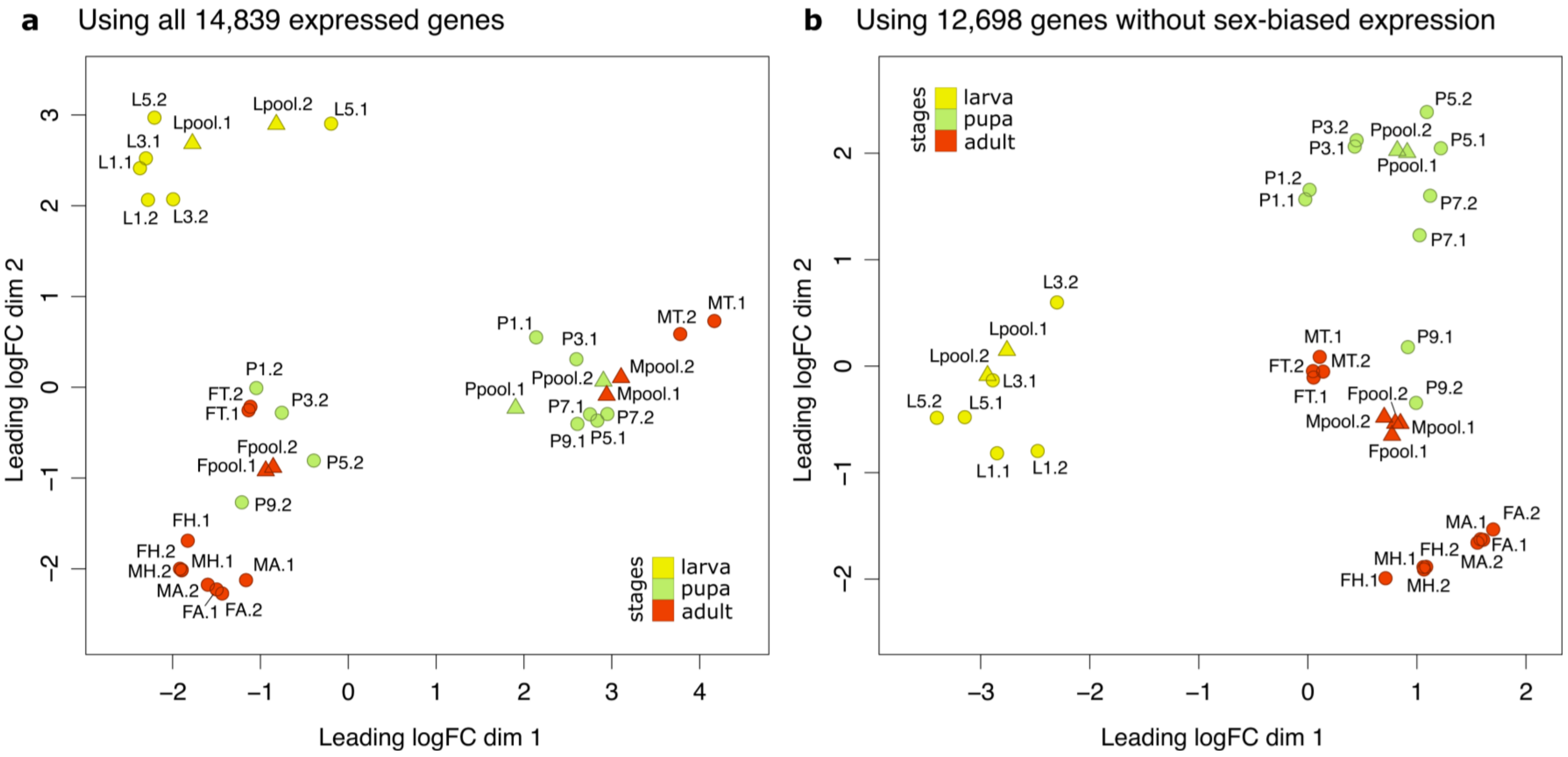

Supplementary Figure 16. Sample relationships using Multidimensional Scaling. a MDS plot of all 36 biological samples from the transcriptome atlas, using the top 2,000 genes with highest fold-changes between pairs of samples. **b** The same MDS plot but first excluding the 2,143 genes that are differentially expressed between pooled females and males. The first two dimensions are shown, with distances representing the $\log_2$(fold-change) of the selected genes between samples. In **a**, sample separation fits developmental differences for larva, however, the pupa samples in particular show unexpected localizations based on their developmental stage, which results from the effect of sex-biased expression. Once genes with sex-biased expression are excluded, sample separation is in agreement with developmental stage as shown in **b**. Samples from different developmental stages were color-coded as in Fig. 2 (larva, yellow; pupa, green; and adult, red). Circles show individual samples sequenced at Langebio (L, larva; P, pupa; F, female; M, male; T, thorax; A, abdomen; H, head) while triangles represent pooled samples sequenced at UC Irvine (Lpool, Ppool, Mpool, Fpool).

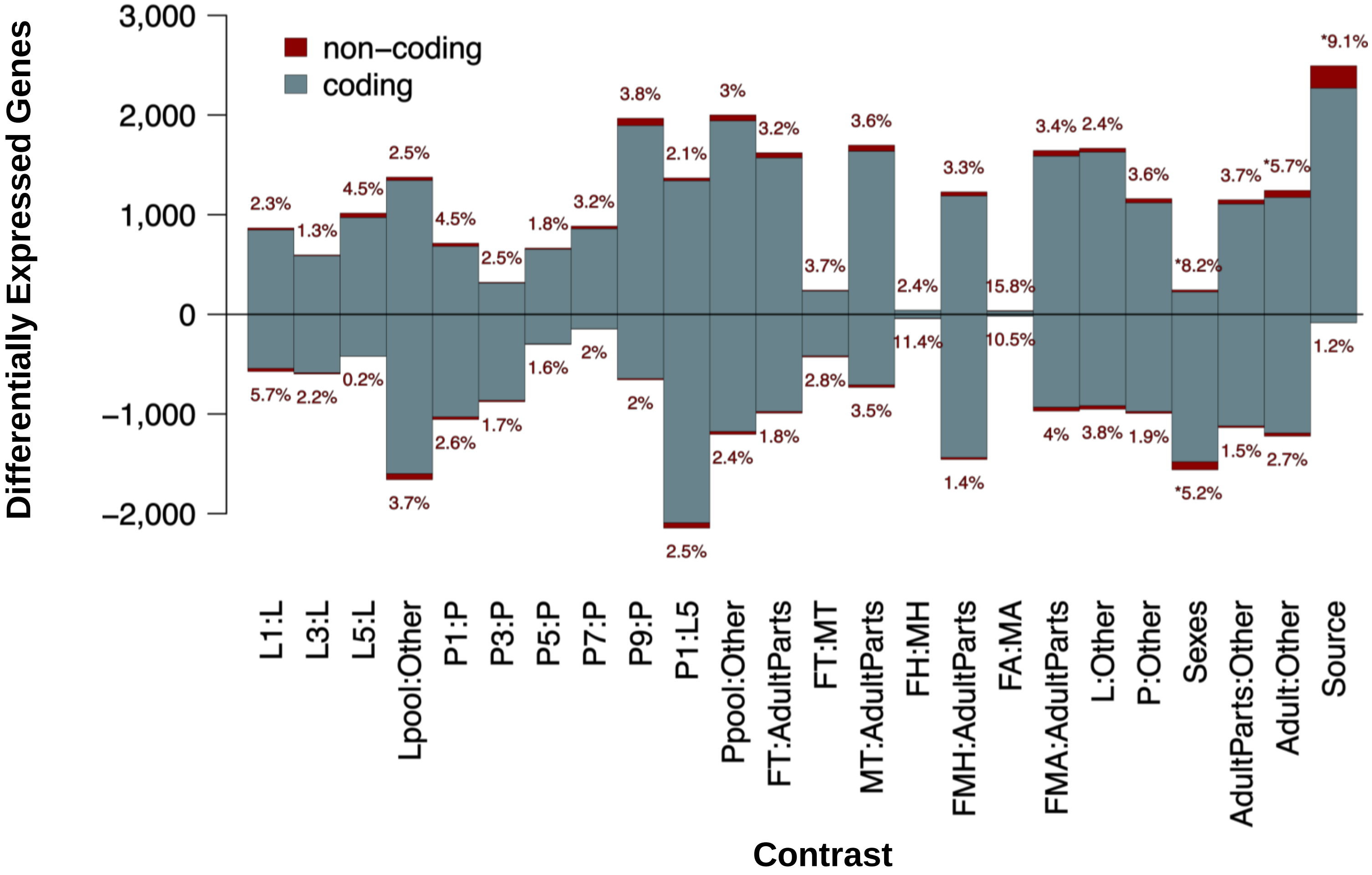

Supplementary Figure 17. Differential expression changes across *D. plexippus* transcriptional atlas. The barplots show the number of differentially over- (y-axis positive values) or under-expressed (y-axis negative values) genes (FDR 5%) between the two samples compared in each of the 24 contrasts (x-axis) performed (Supplementary Table 13). Protein-coding and lncRNA genes are shown in dark grey and red respectively, and the percentage of lncRNAs contributing to differential expression in indicated (* denotes significantly higher presence than the random expectation as in Supplementary Table 13). In total, 9,749 genes were found differentially expressed in at least one of the contrasts. Across contrasts, a total of 9,162 and 7,770 genes experienced overexpression and underexpression, respectively, between the samples compared.

Supplementary Figure 18. ***L1:L***

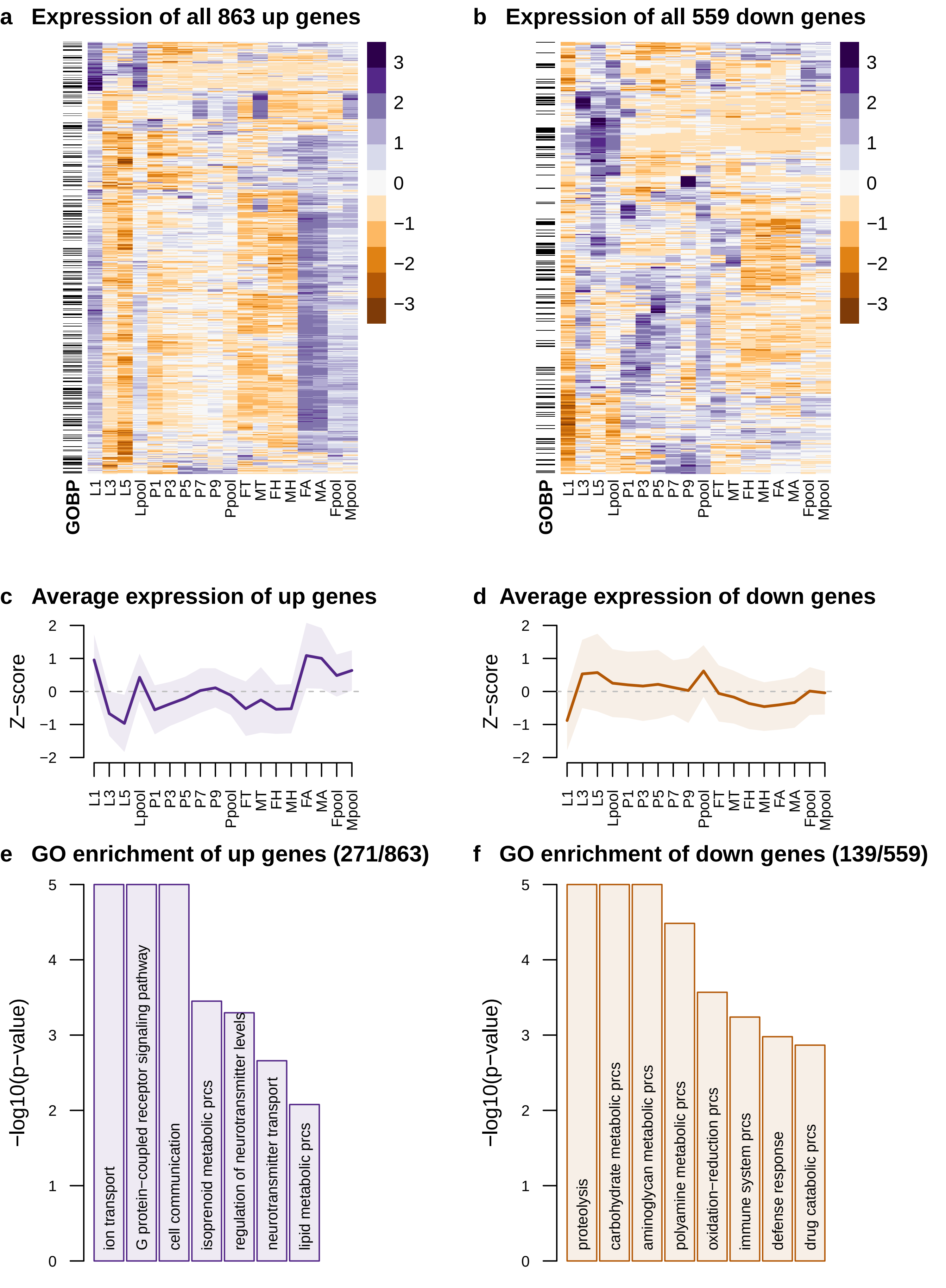

Supplementary Figure 18. ***L3:L***

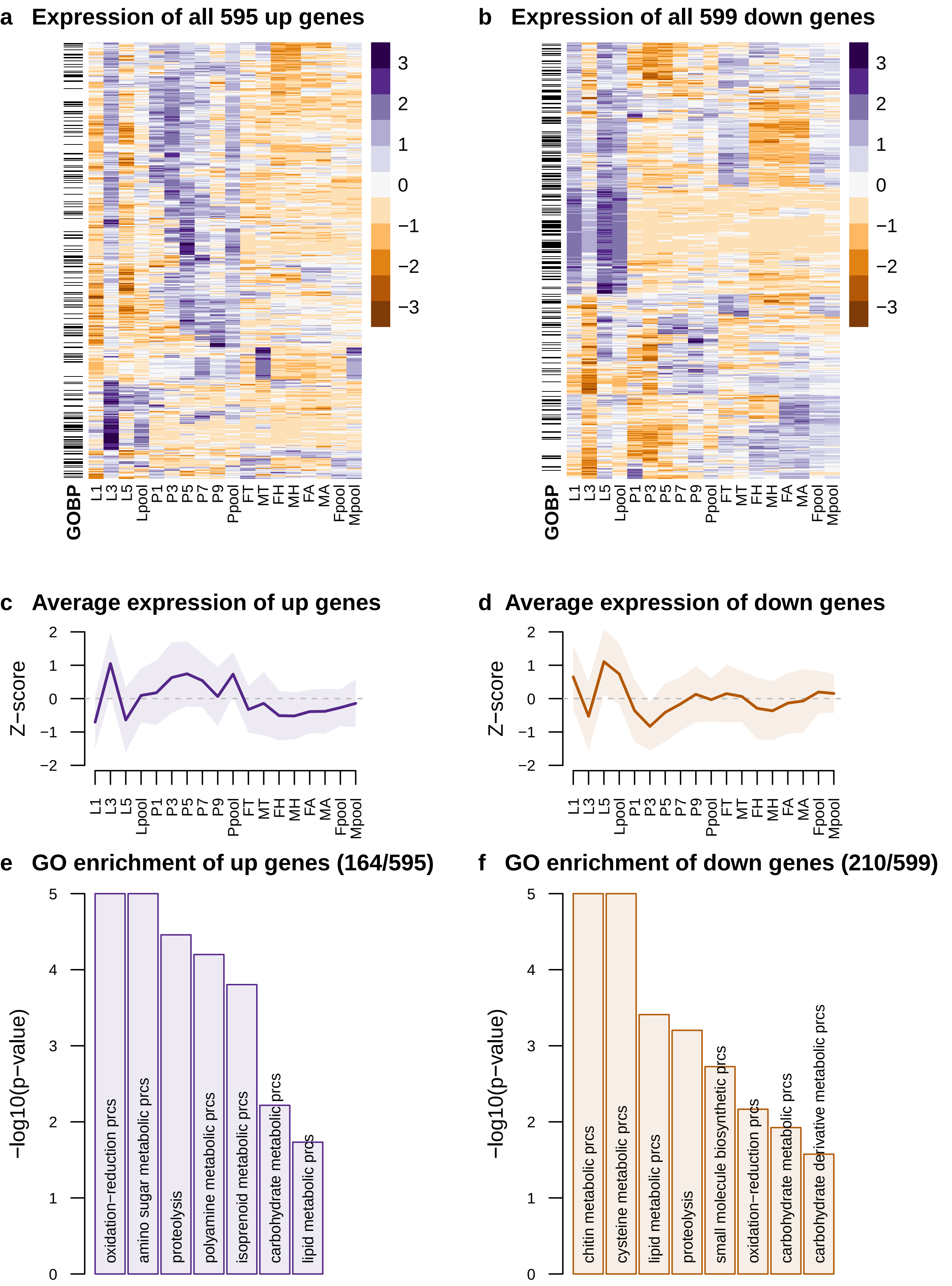

Supplementary Figure 18. *L5:L*

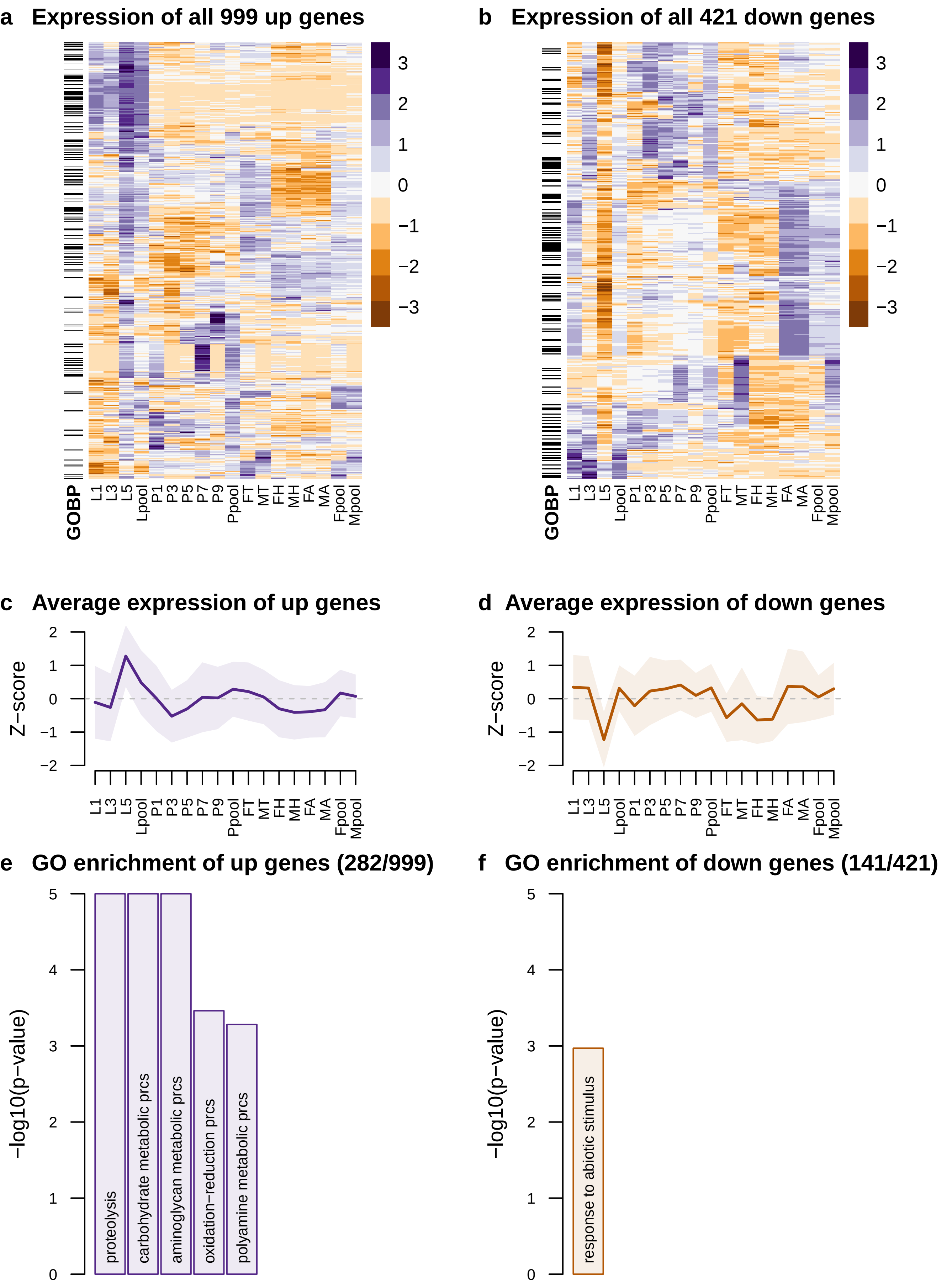

Supplementary Figure 18. *Lpool:Other*

a **Expression of all 1377 up genes**

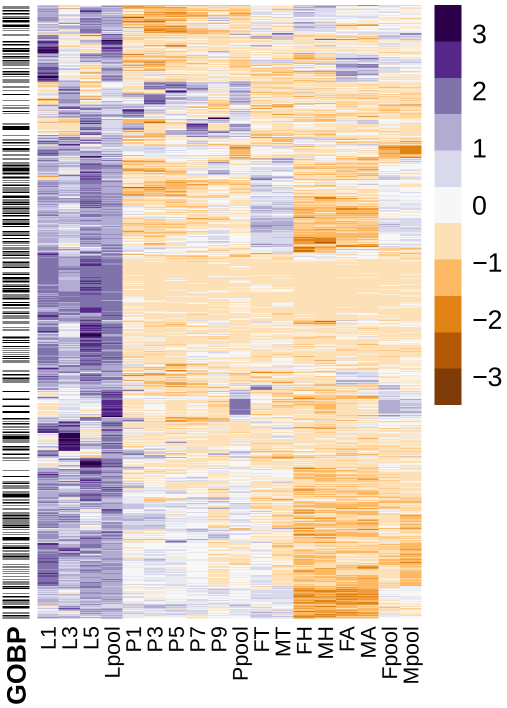

b **Expression of all 1661 down genes**

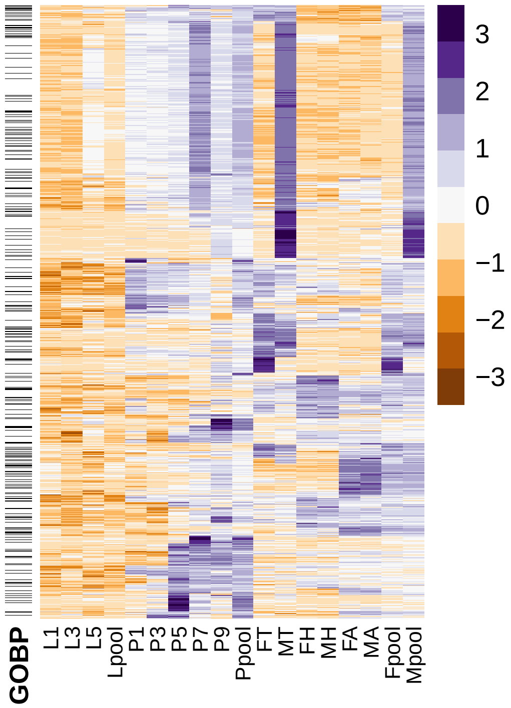

c **Average expression of up genes**

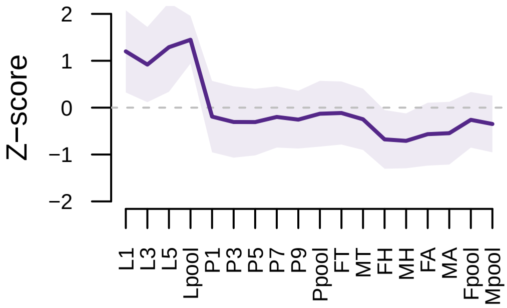

d **Average expression of down genes**

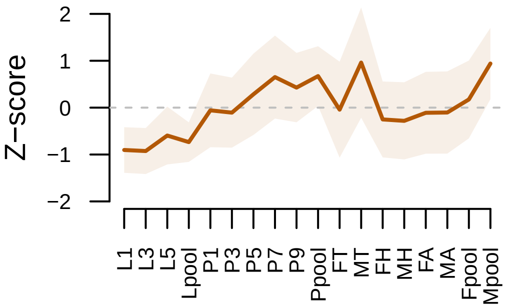

e **GO enrichment of up genes (517/1377)**

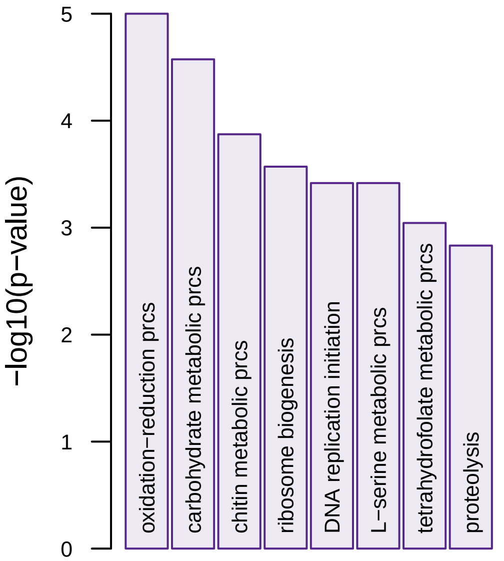

f **GO enrichment of down genes (320/1661)**

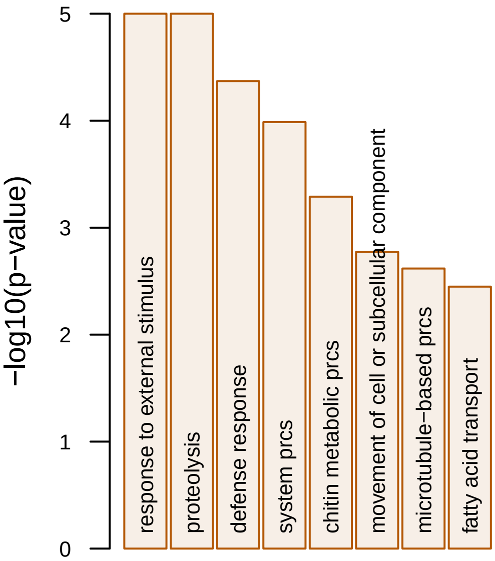

Supplementary Figure 18. ***P1:P***

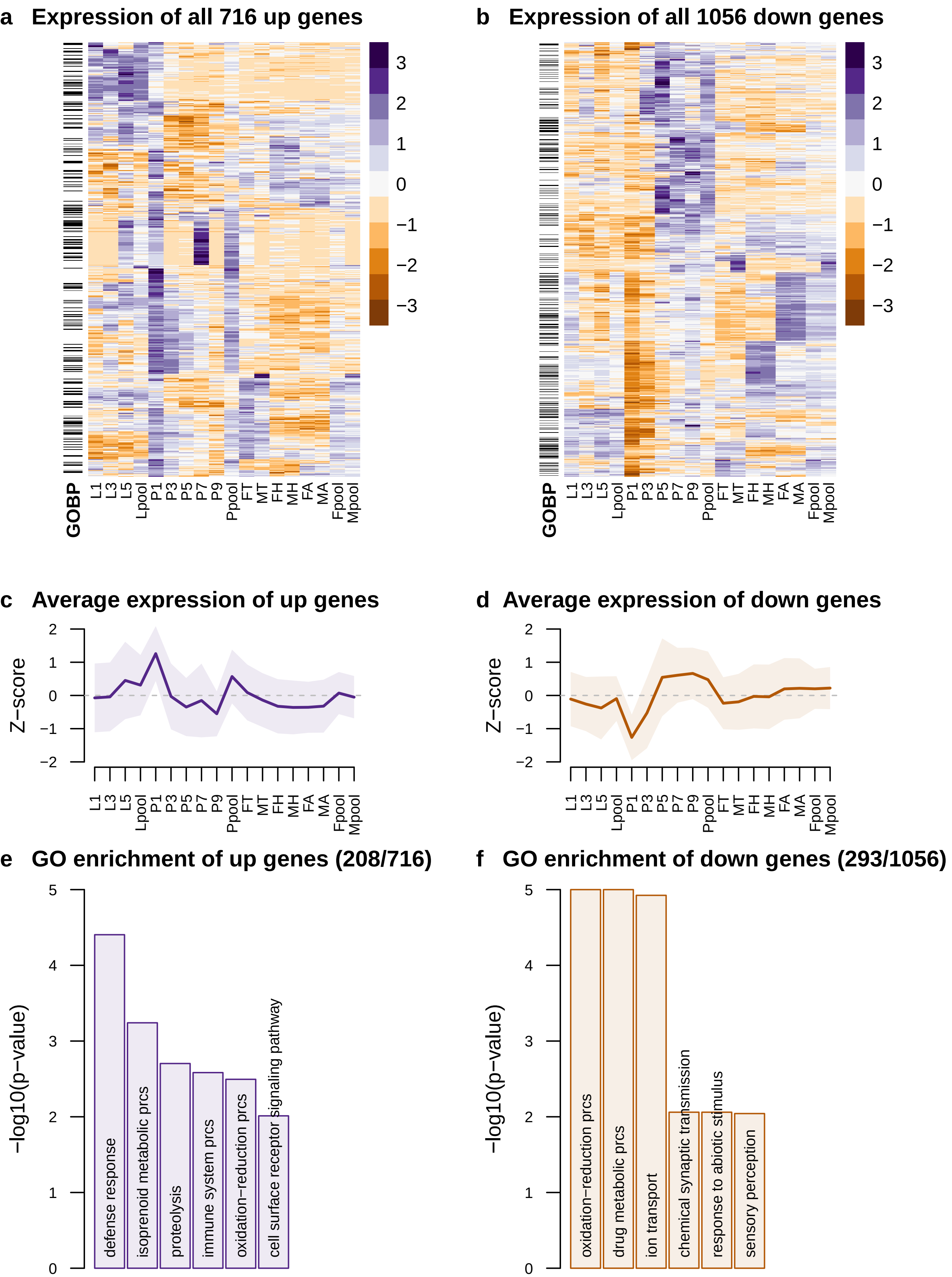

Supplementary Figure 18. ***P3:P***

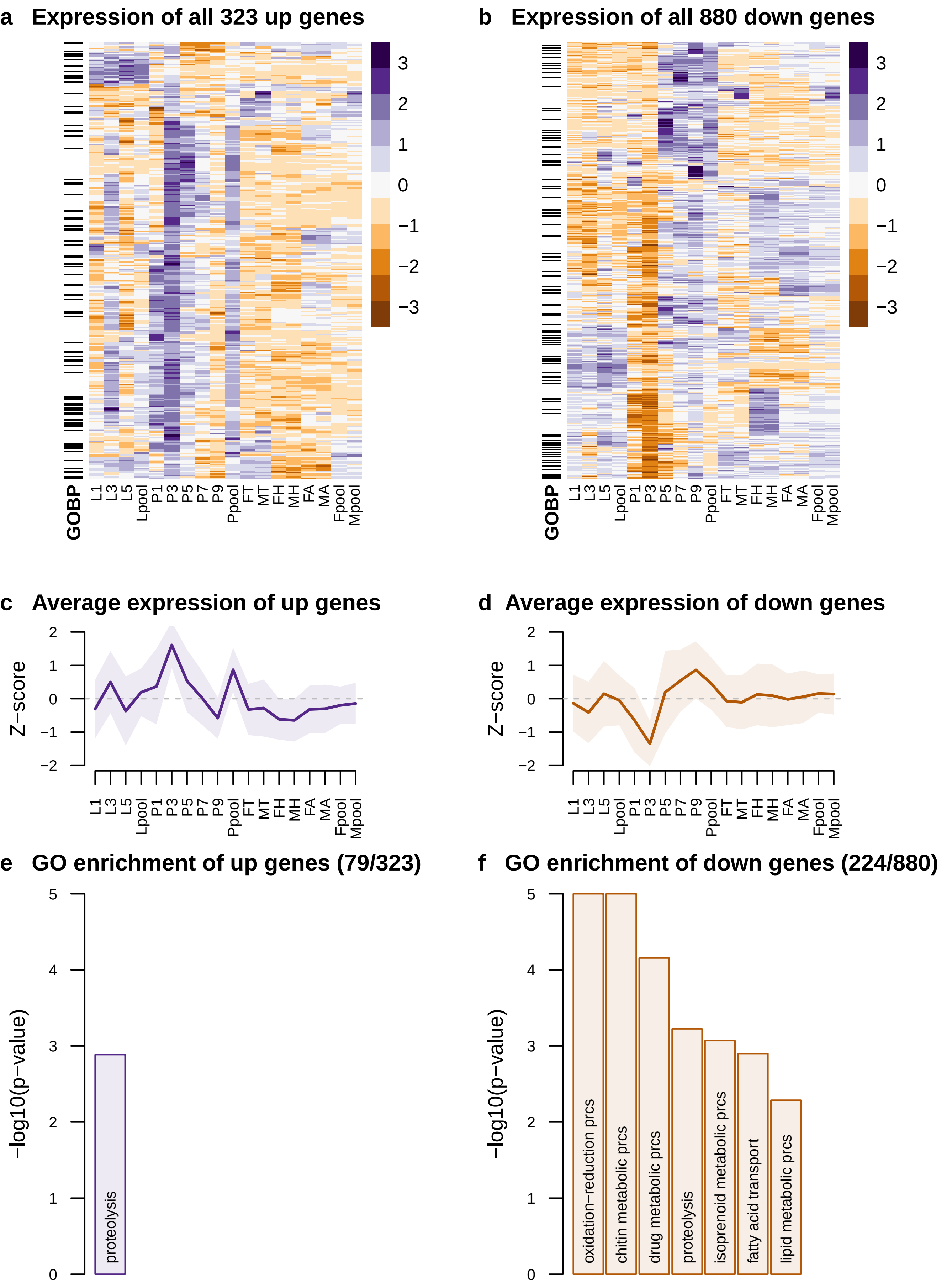

Supplementary Figure 18. *P5:P*

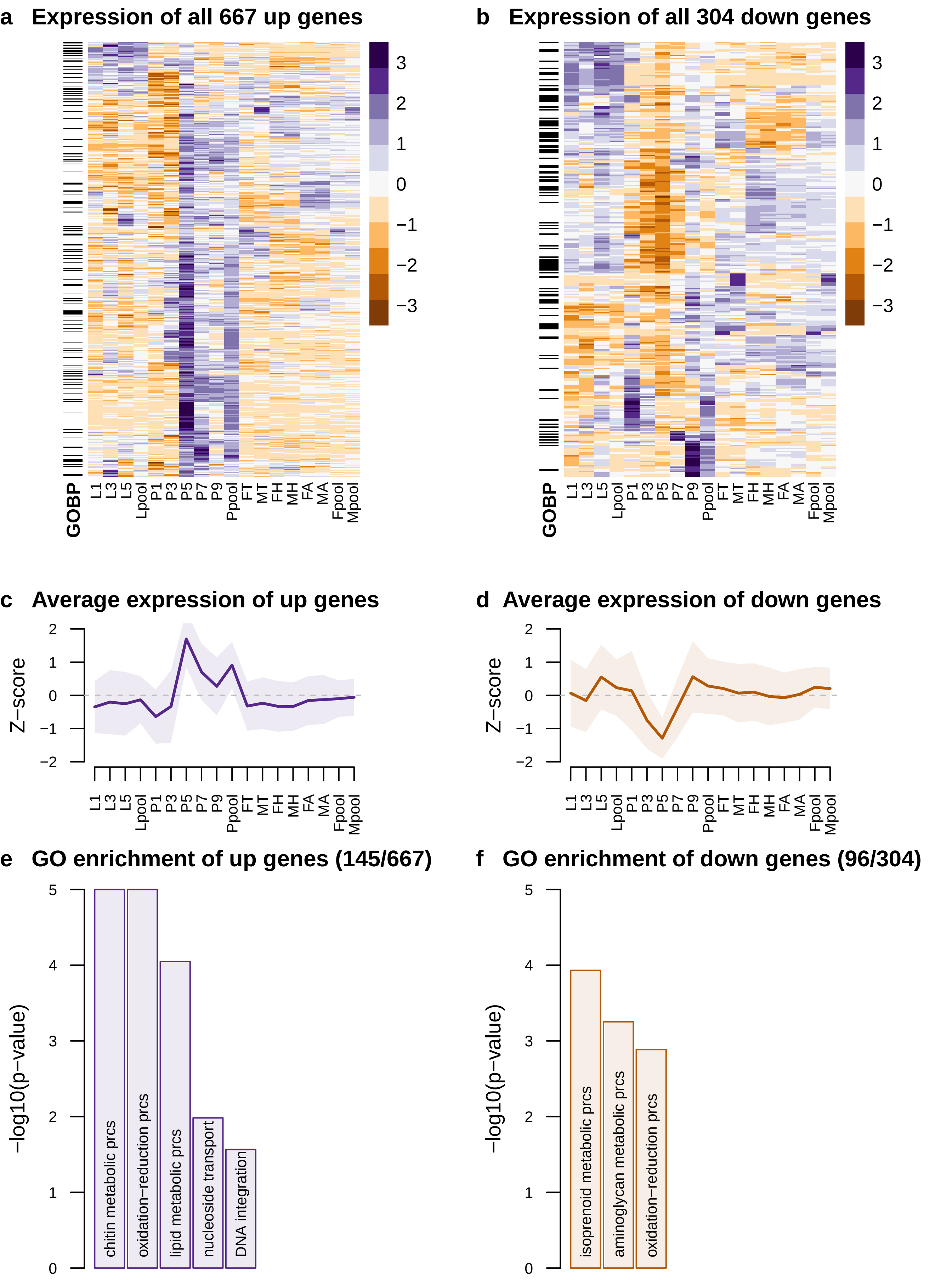

Supplementary Figure 18. *P7:P*

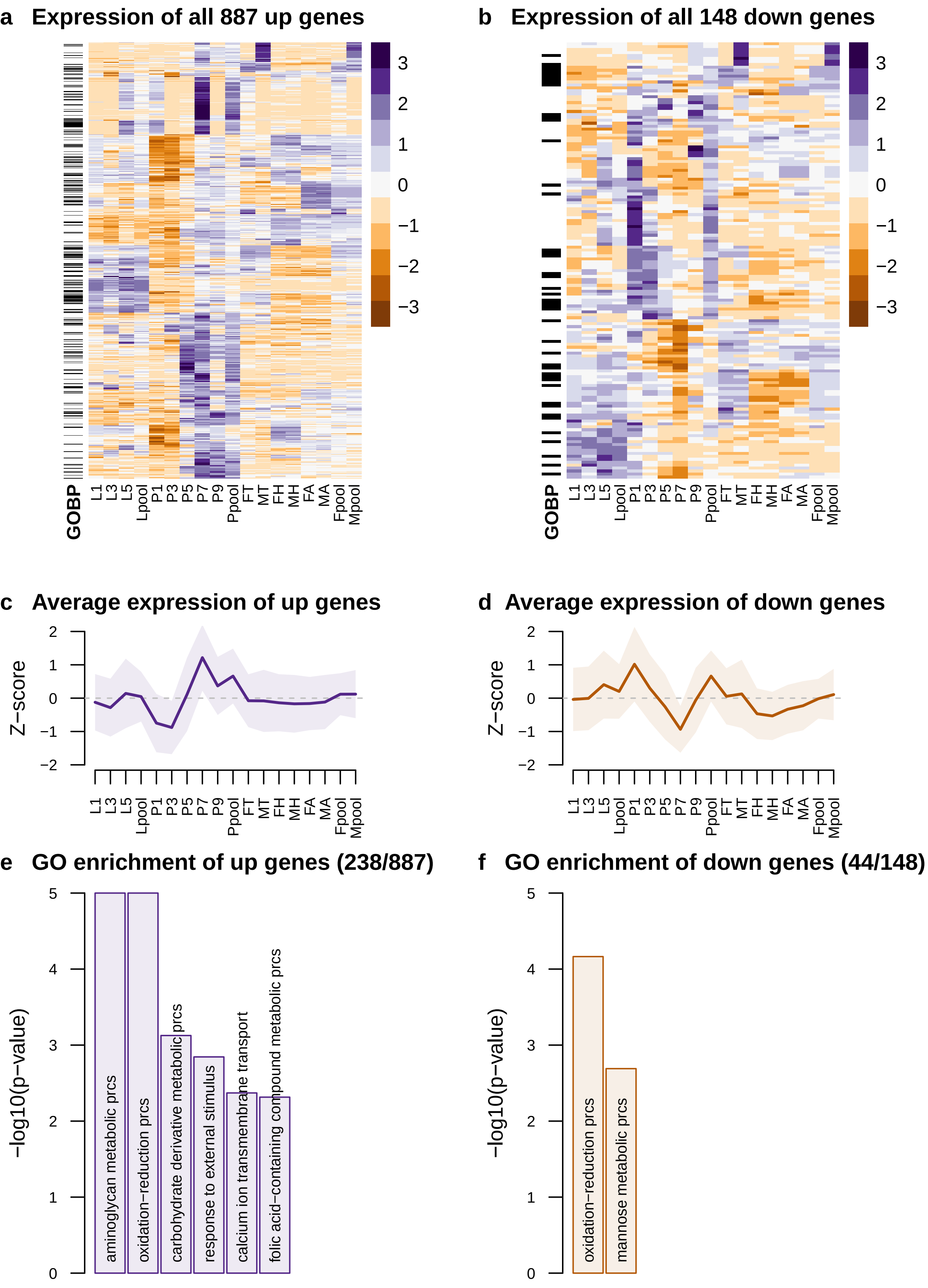

Supplementary Figure 18. *P9:P*

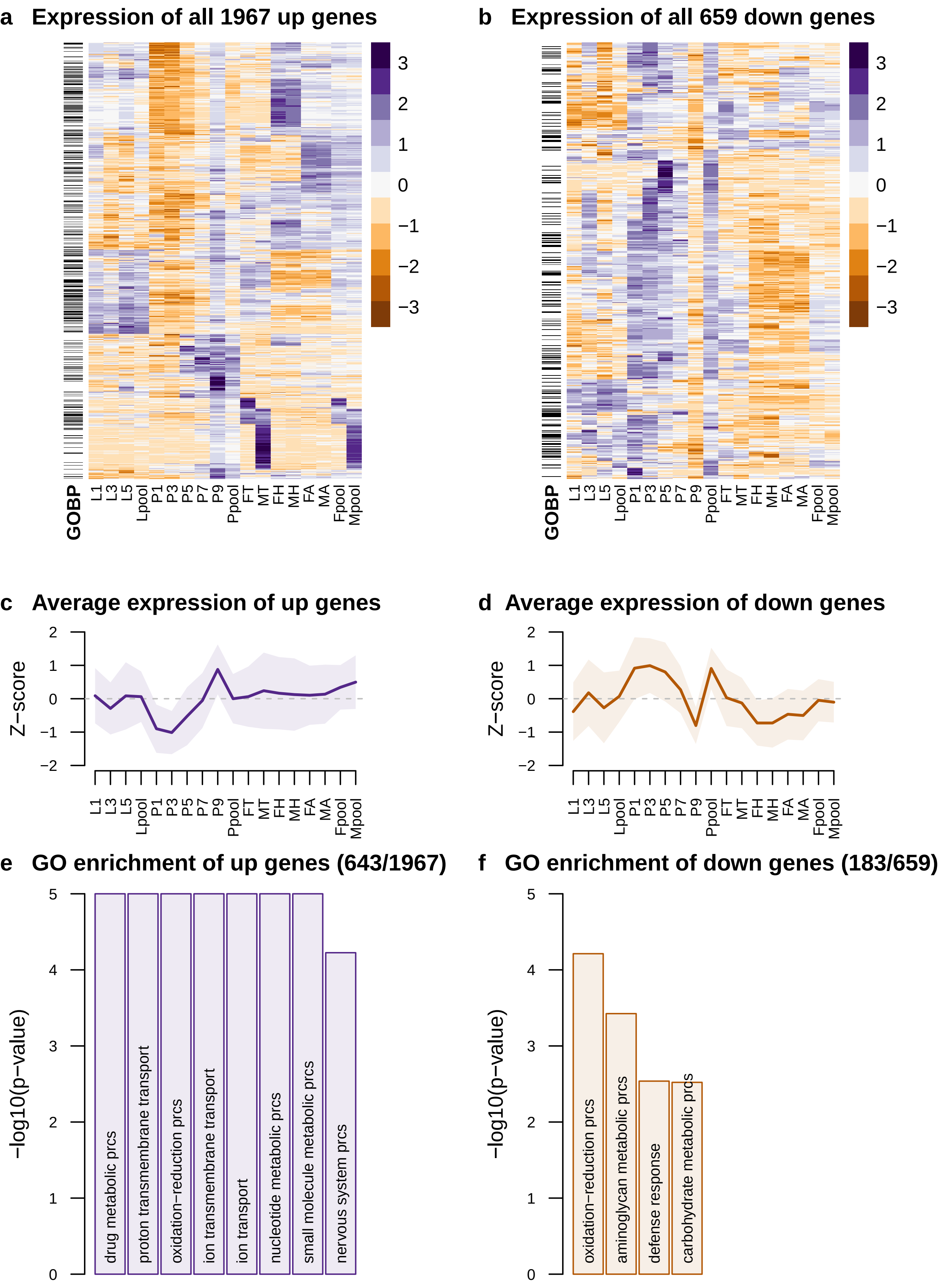

Supplementary Figure 18. *P1:L5*

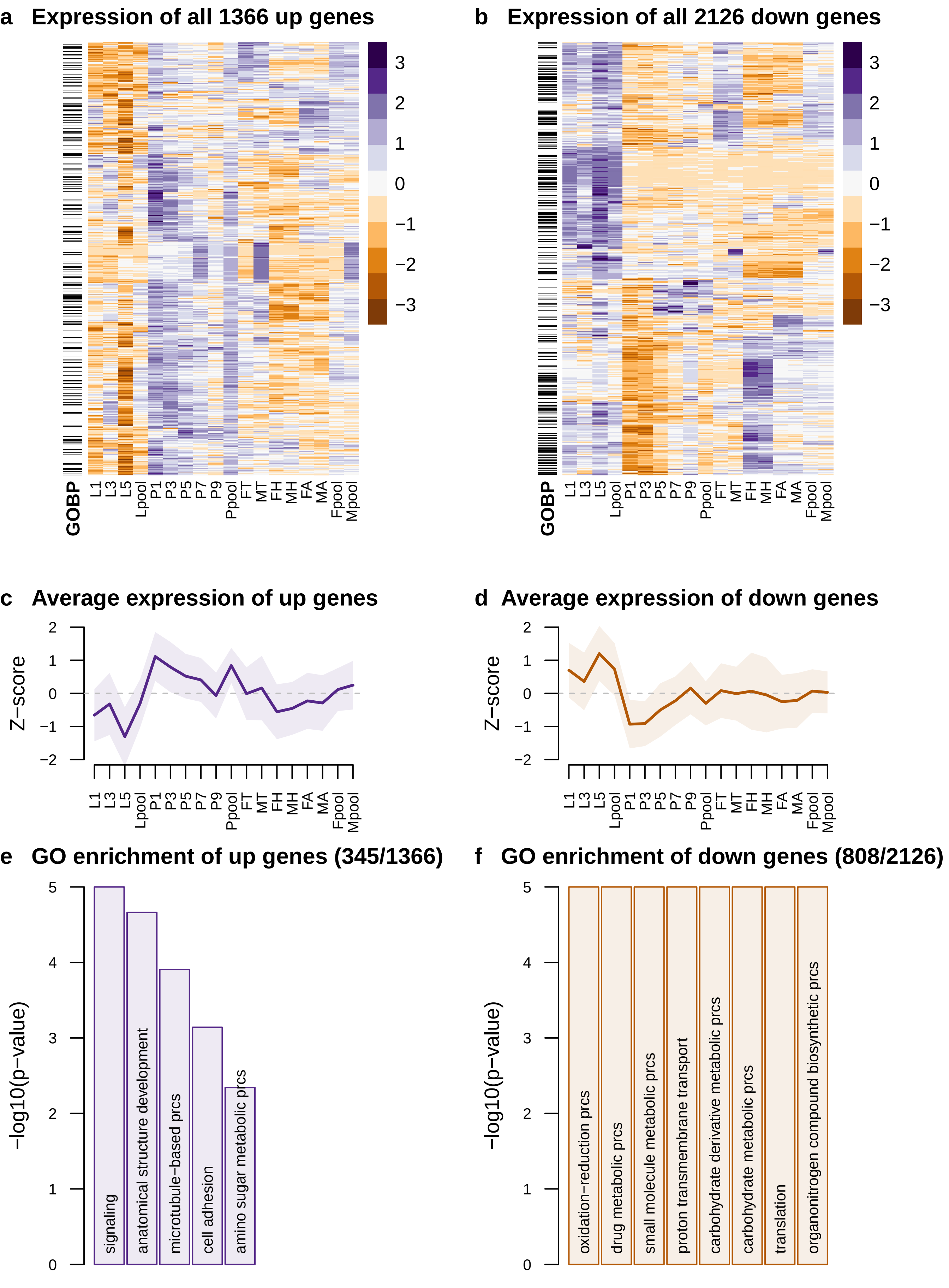

Supplementary Figure 18. *Ppool:Other*

a Expression of all 2003 up genes

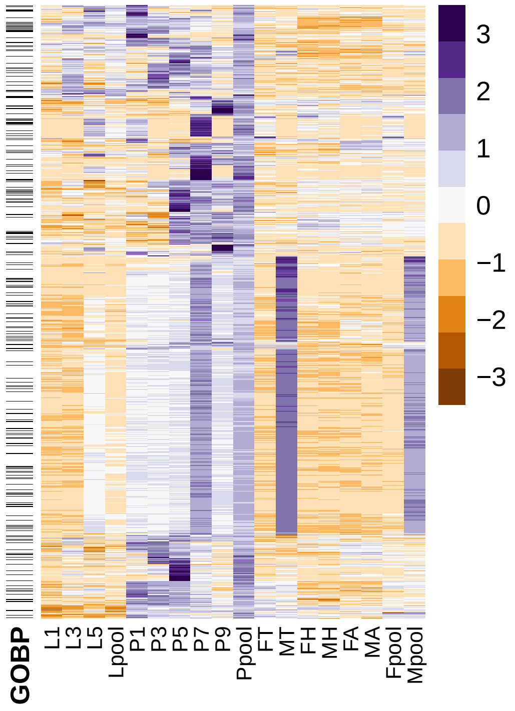

b Expression of all 1203 down genes

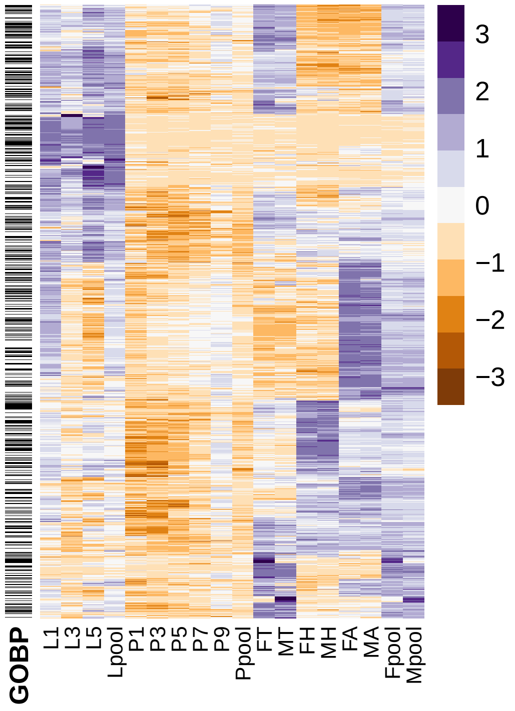

c Average expression of up genes

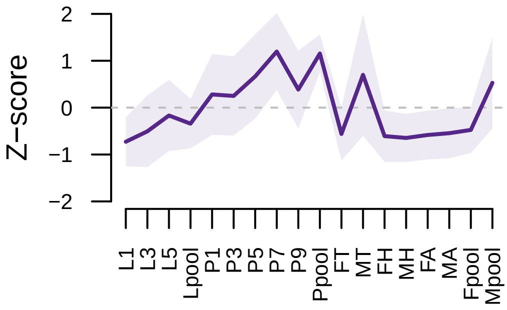

d Average expression of down genes

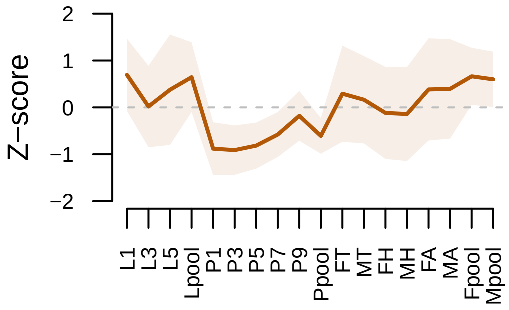

e GO enrichment of up genes (383/2003)

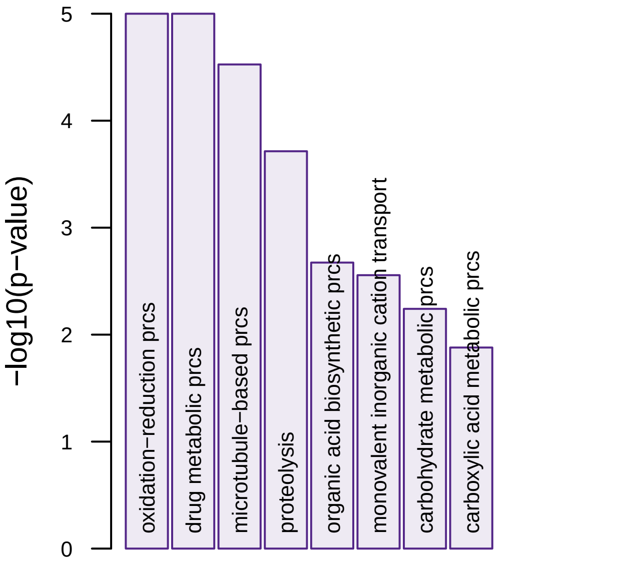

f GO enrichment of down genes (507/1203)

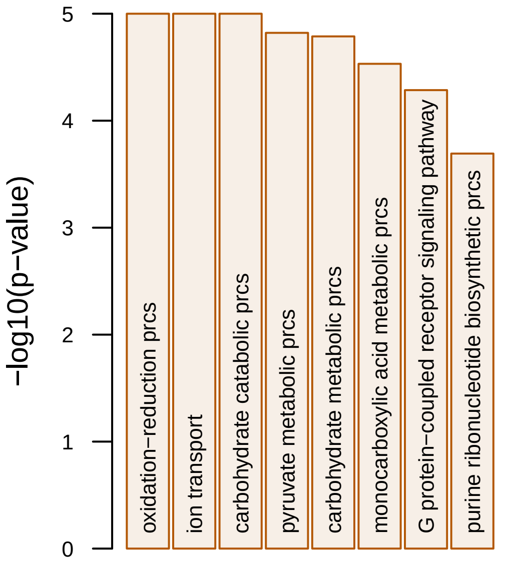

Supplementary Figure 18. *FT:AdultParts*

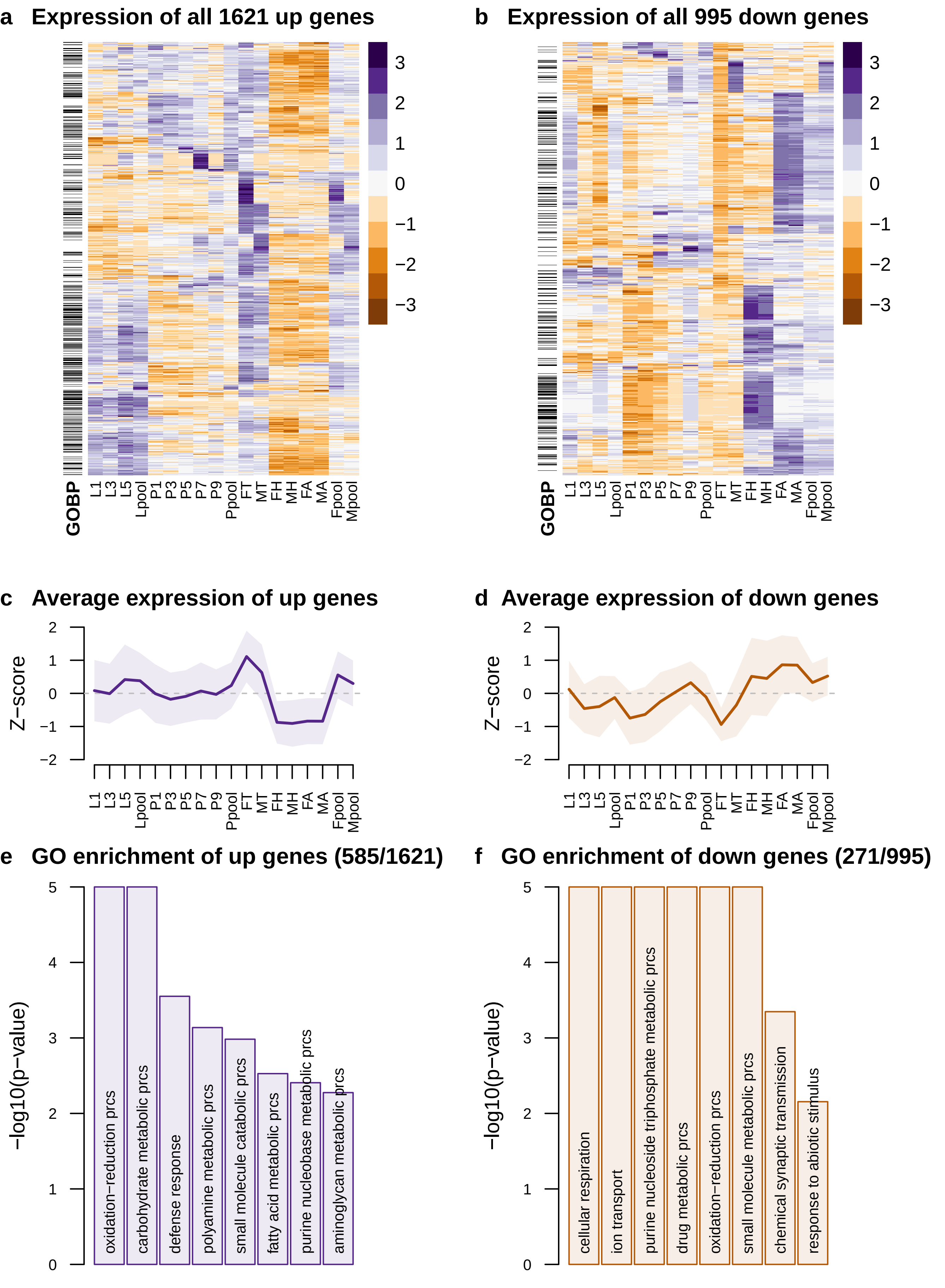

Supplementary Figure 18. *FT:MT*

a Expression of all 243 up genes

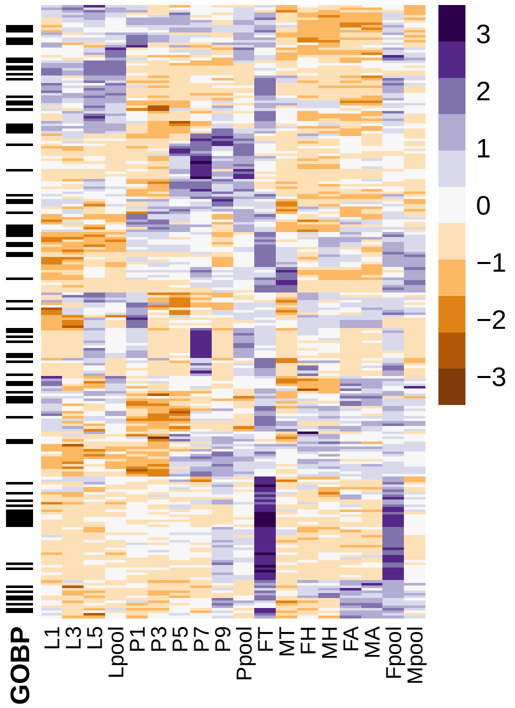

b Expression of all 429 down genes

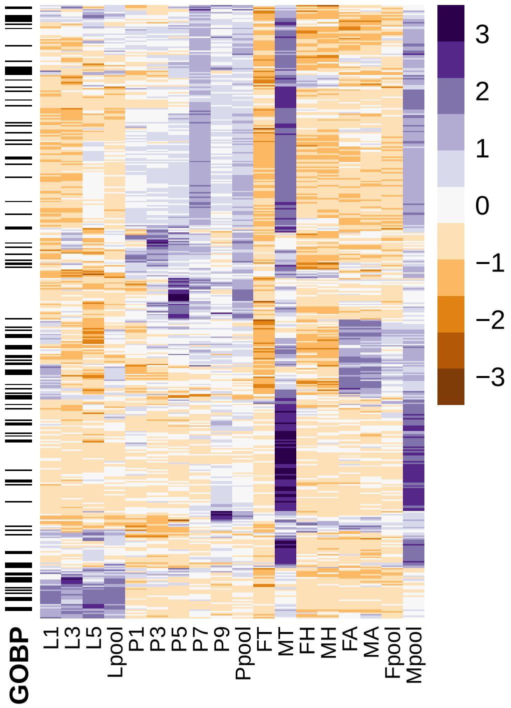

c Average expression of up genes

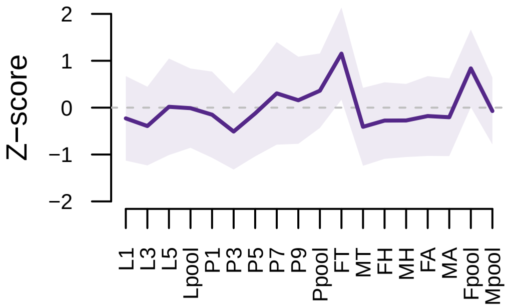

d Average expression of down genes

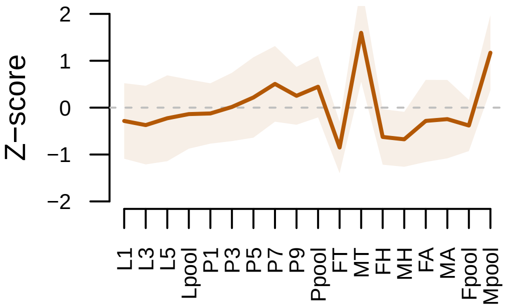

e GO enrichment of up genes (75/243)

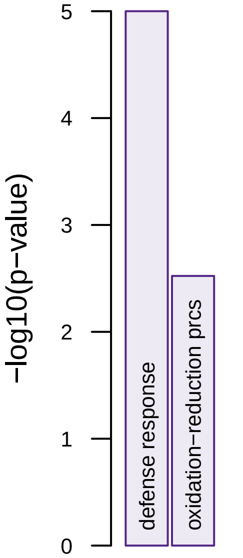

f GO enrichment of down genes (104/429)

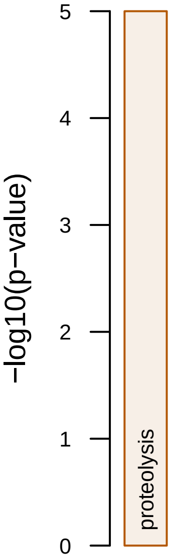

Supplementary Figure 18. *MT:AdultParts*

a Expression of all 1694 up genes

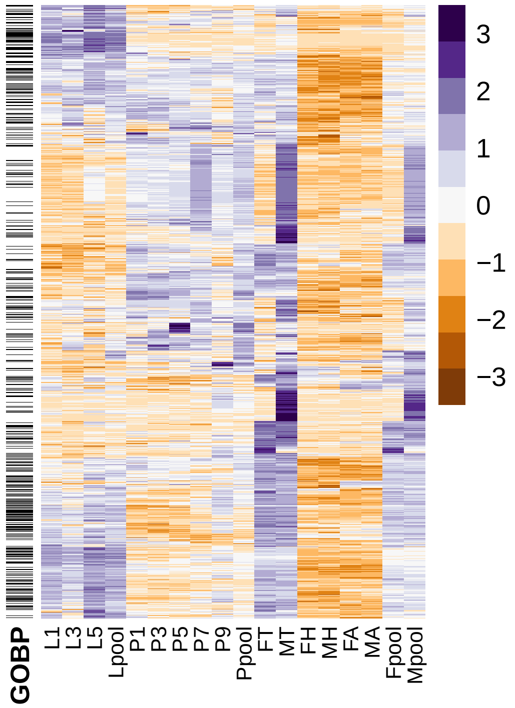

b Expression of all 735 down genes

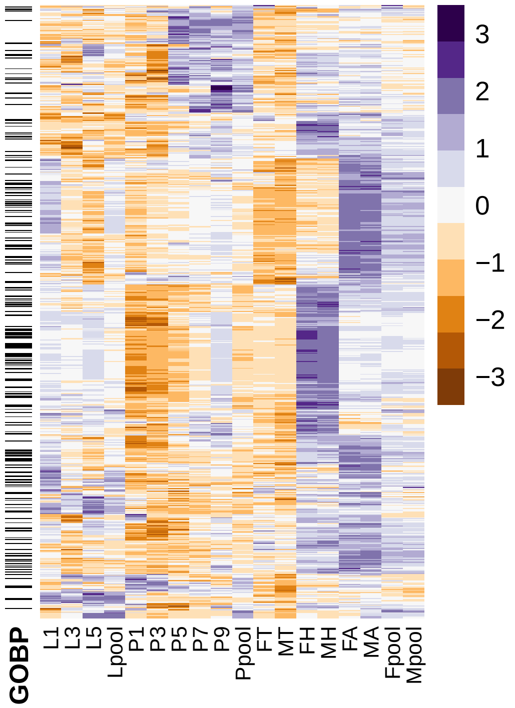

c Average expression of up genes

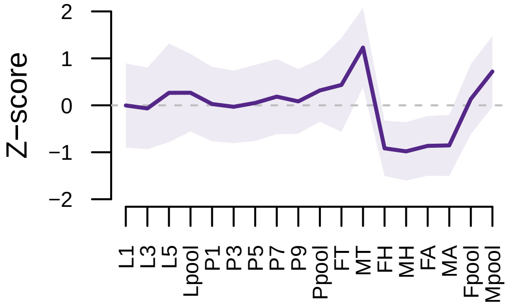

d Average expression of down genes

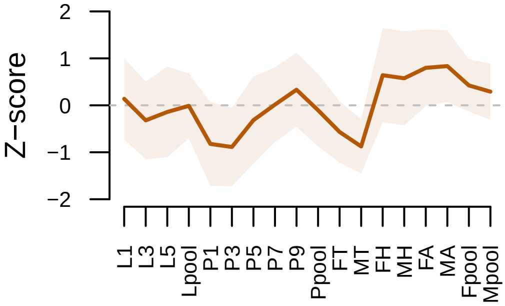

e GO enrichment of up genes (564/1694)

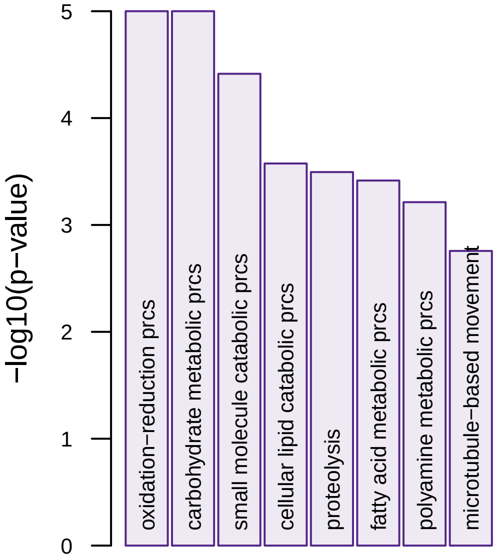

f GO enrichment of down genes (202/735)

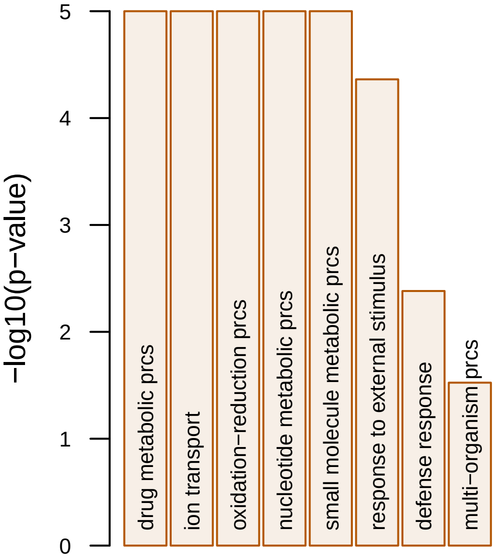

Supplementary Figure 18. *FH:MH*

a Expression of all 42 up genes

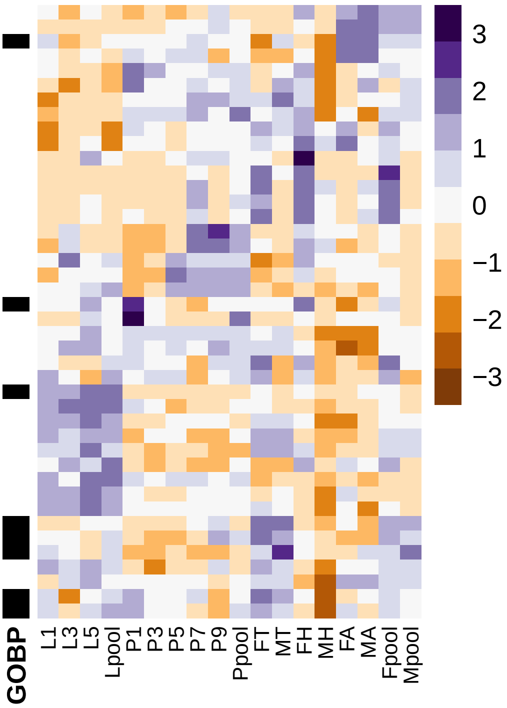

b Expression of all 44 down genes

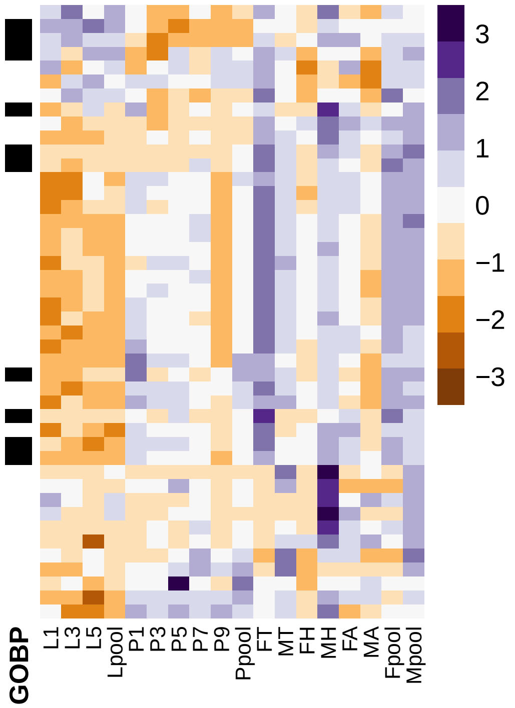

c Average expression of up genes

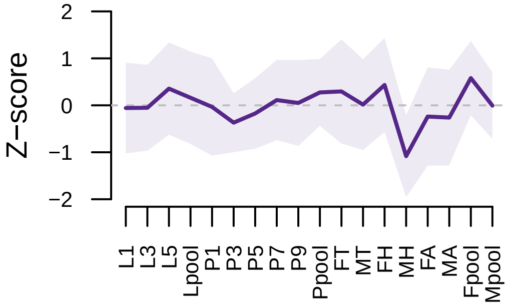

d Average expression of down genes

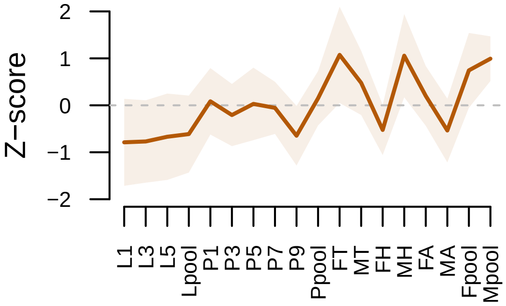

e GO enrichment of up genes (8/42)

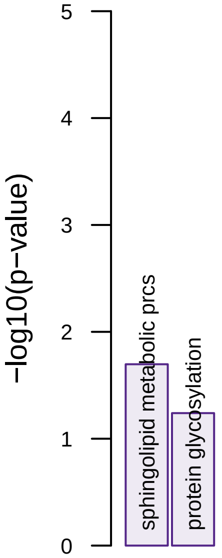

f GO enrichment of down genes (10/44)

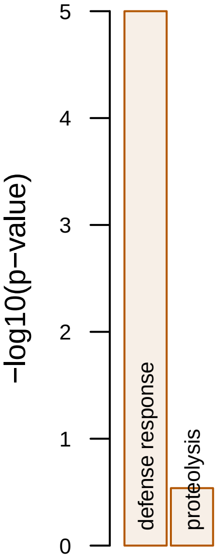

Supplementary Figure 18. *FMH:AdultParts*

a Expression of all 1233 up genes

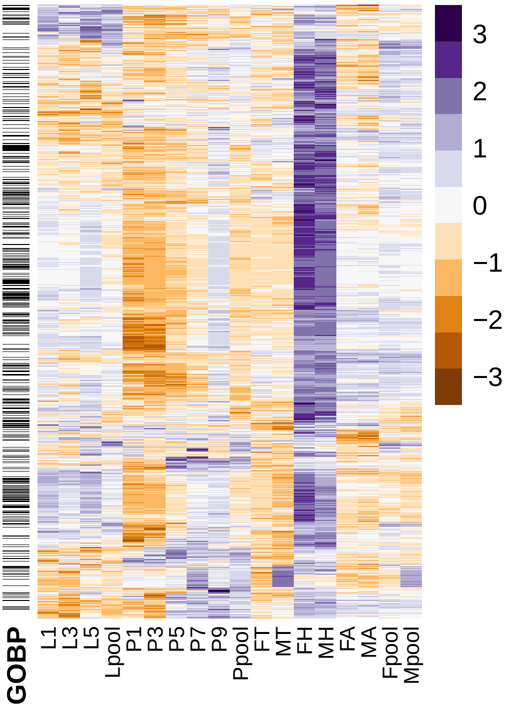

b Expression of all 1456 down genes

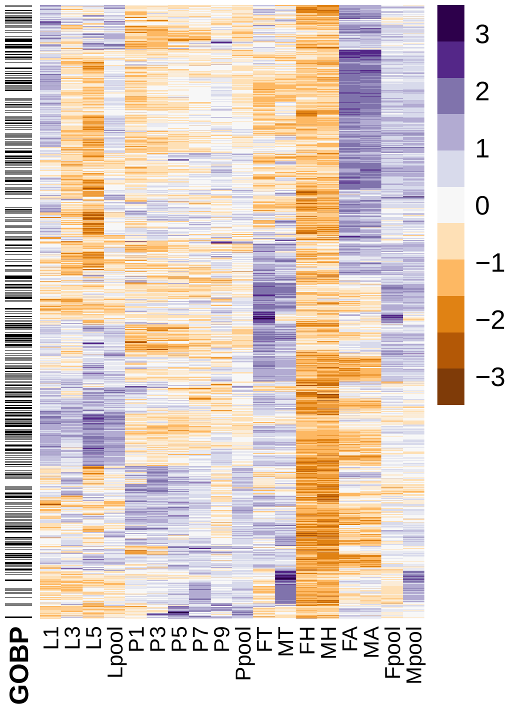

c Average expression of up genes

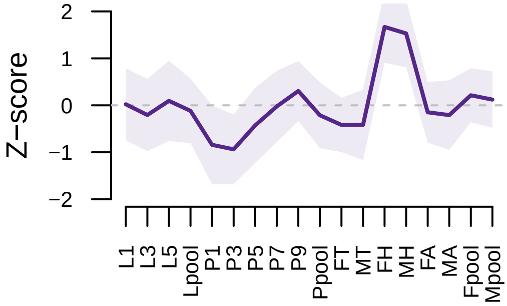

d Average expression of down genes

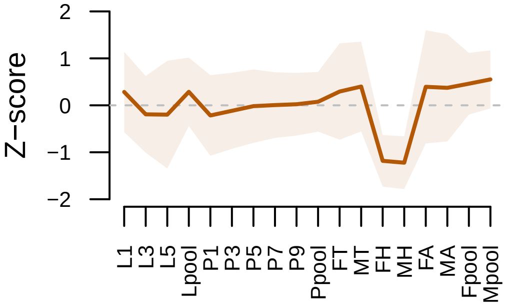

e GO enrichment of up genes (391/1233)

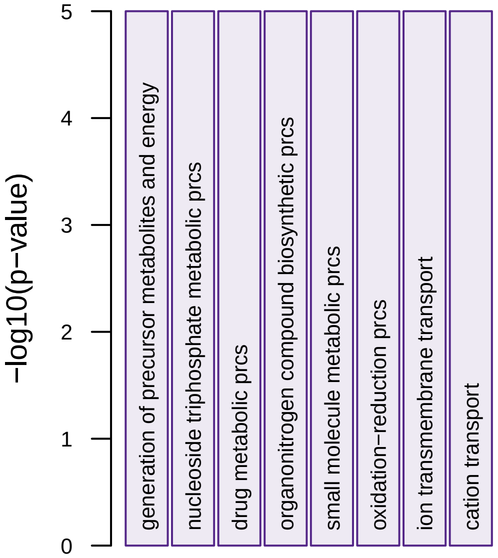

f GO enrichment of down genes (536/1456)

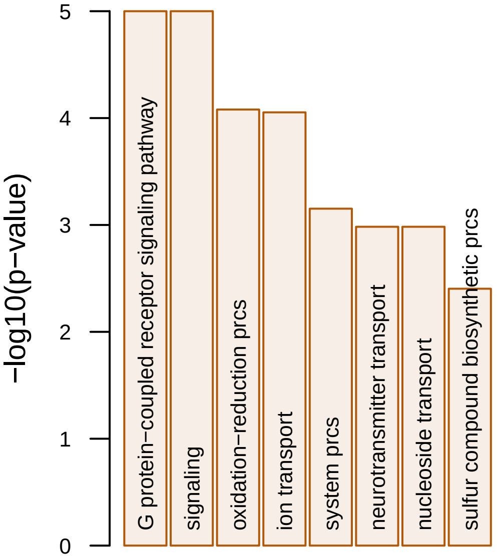

Supplementary Figure 18. *FA:MA*

a Expression of all 38 up genes

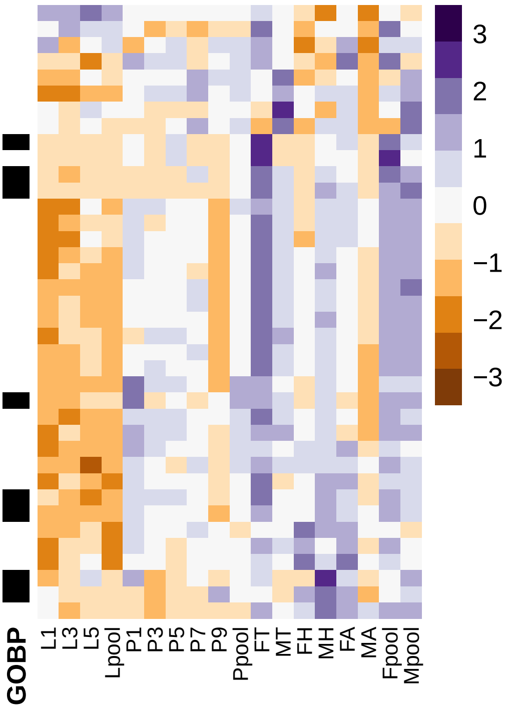

b Expression of all 19 down genes

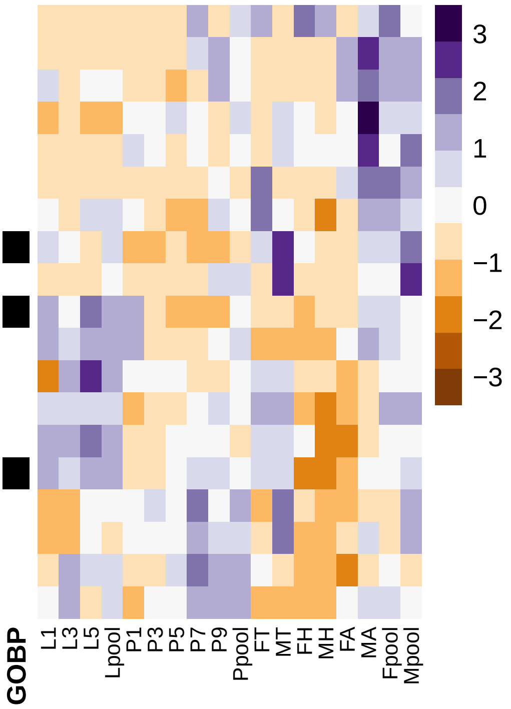

c Average expression of up genes

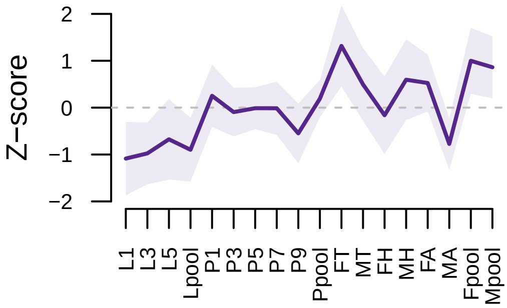

d Average expression of down genes

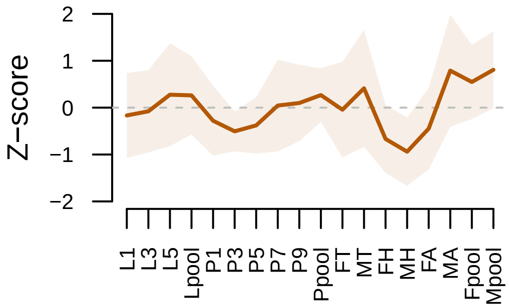

e GO enrichment of up genes (8/38)

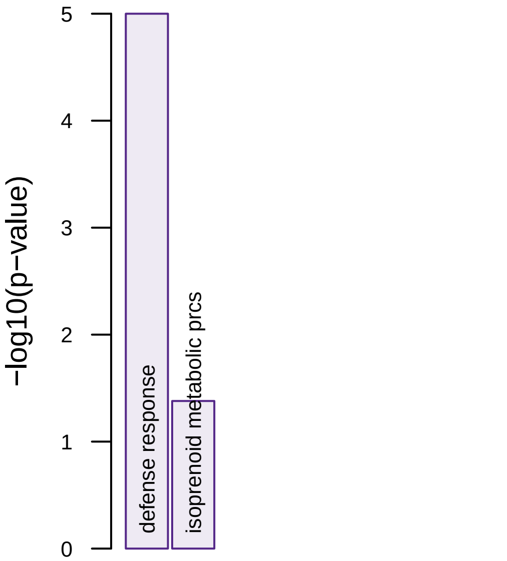

f GO enrichment of down genes (3/19)

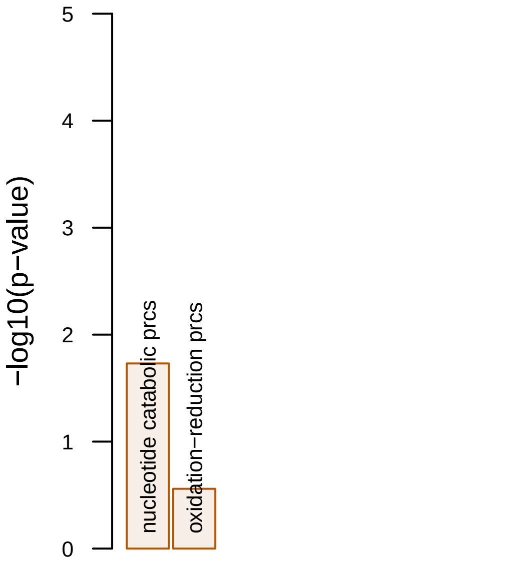

Supplementary Figure 18. *FMA:AdultParts*

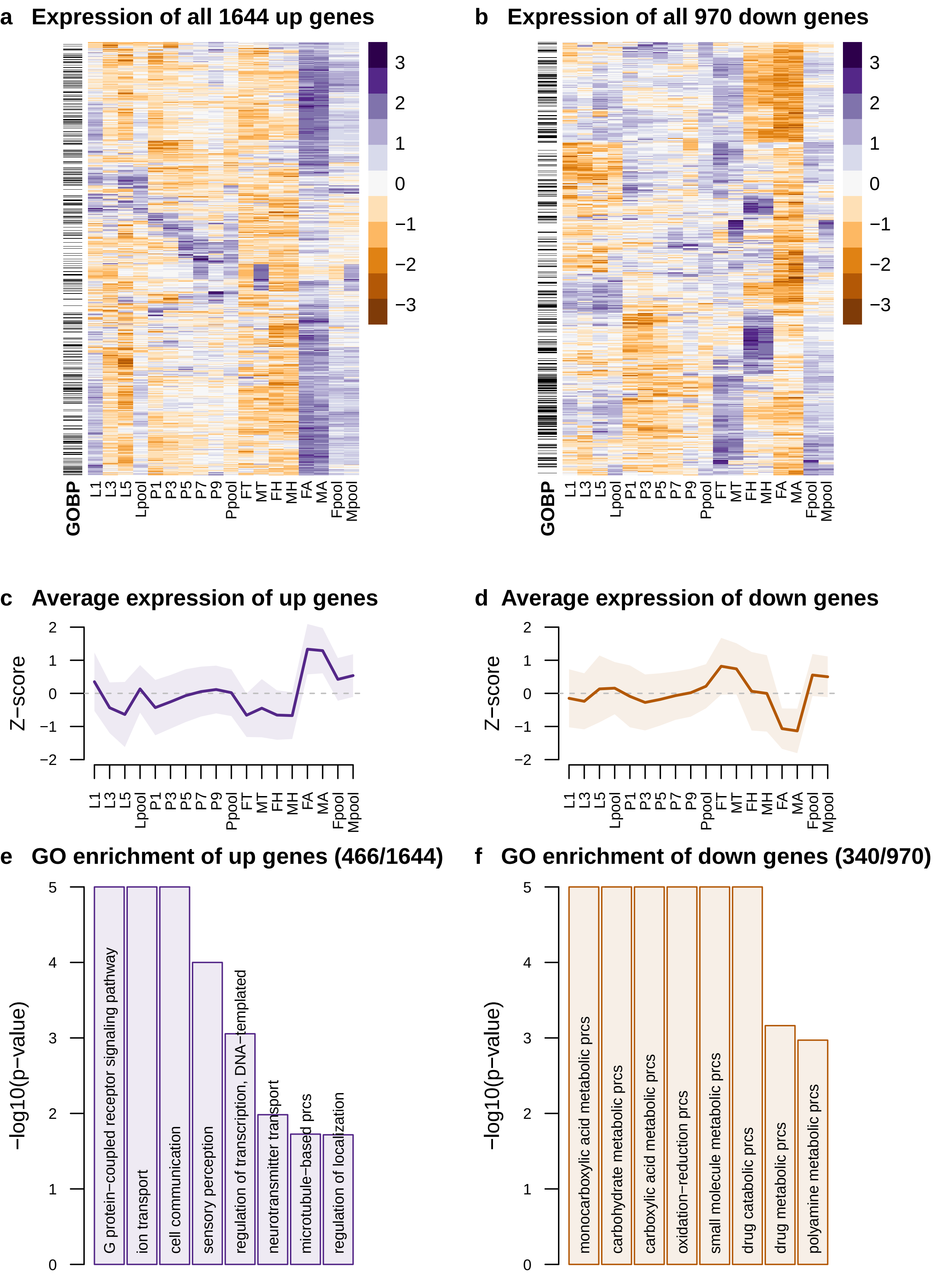

Supplementary Figure 18. *L:Other*

a Expression of all 1654 up genes

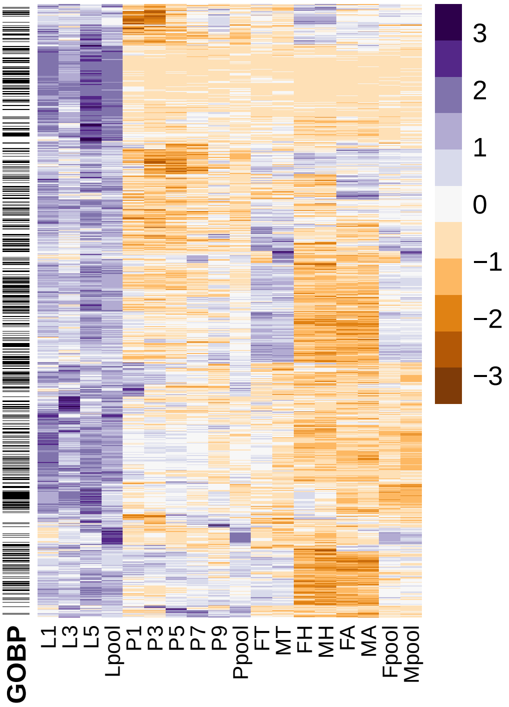

b Expression of all 953 down genes

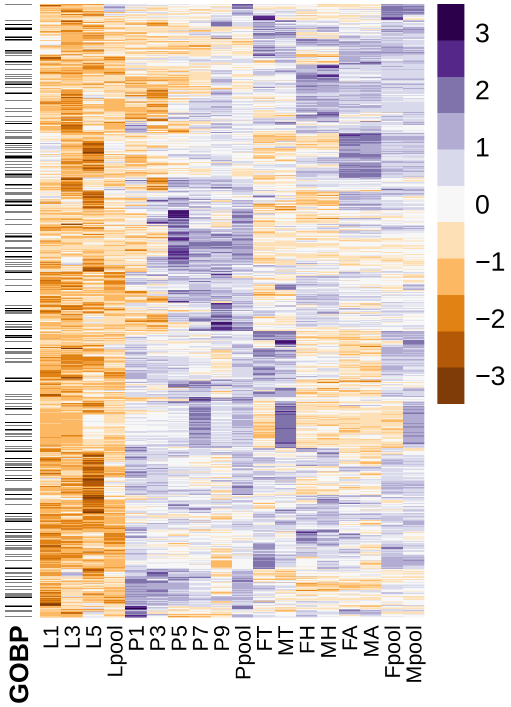

c Average expression of up genes

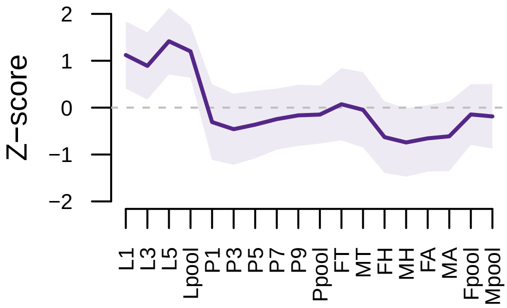

d Average expression of down genes

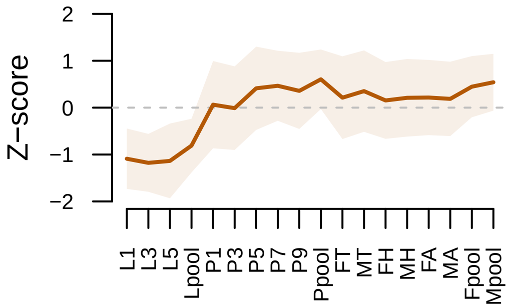

e GO enrichment of up genes (663/1654)

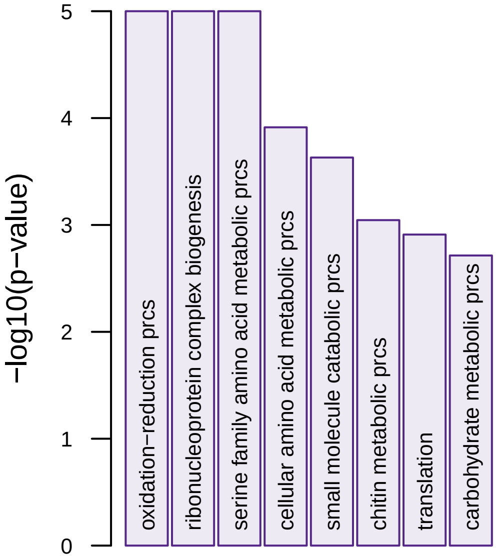

f GO enrichment of down genes (190/953)

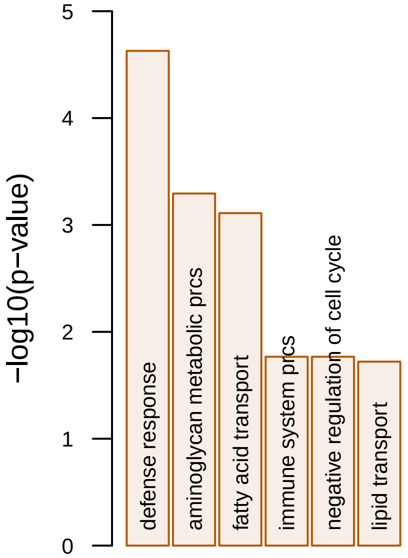

Supplementary Figure 18. *P:Other*

a **Expression of all 1162 up genes**

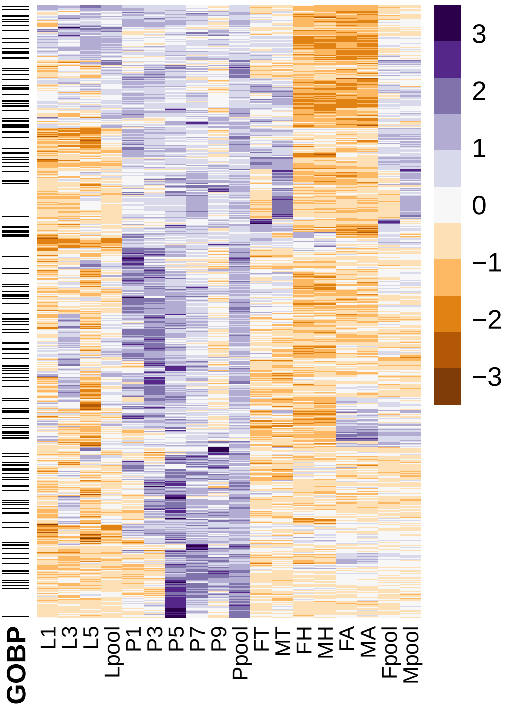

b **Expression of all 994 down genes**

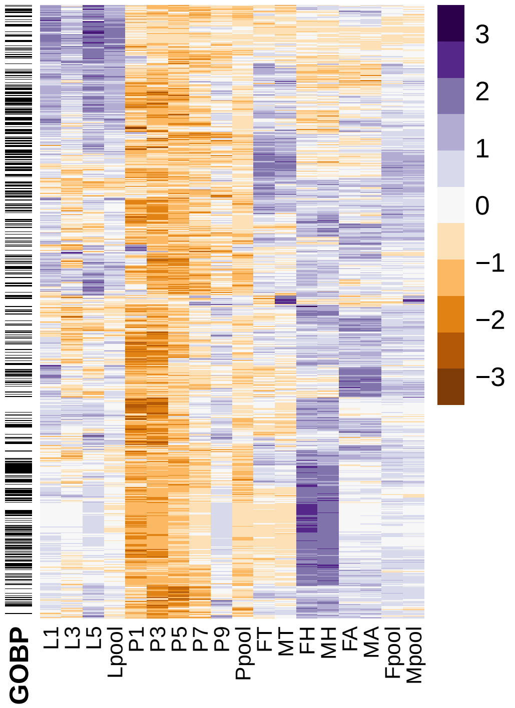

c **Average expression of up genes**

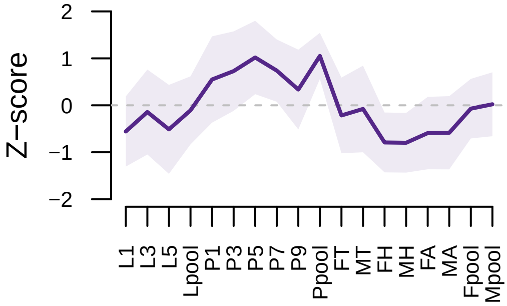

d **Average expression of down genes**

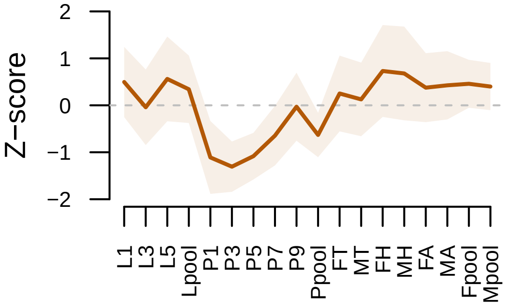

e **GO enrichment of up genes (295/1162)**

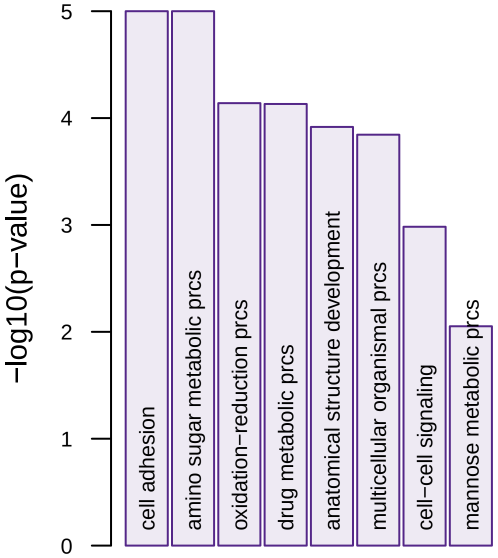

f **GO enrichment of down genes (394/994)**

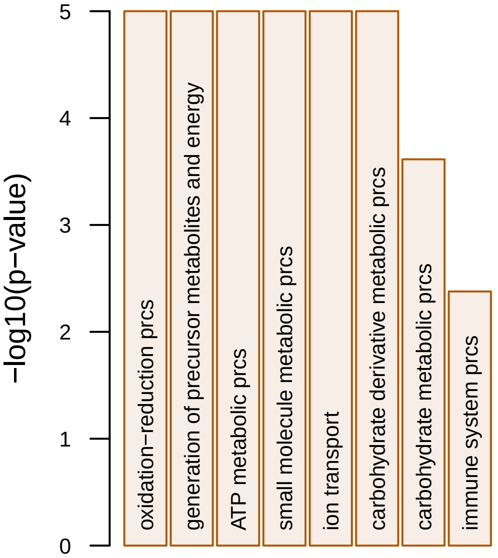

Supplementary Figure 18. *Sexes*

a Expression of all 245 up genes

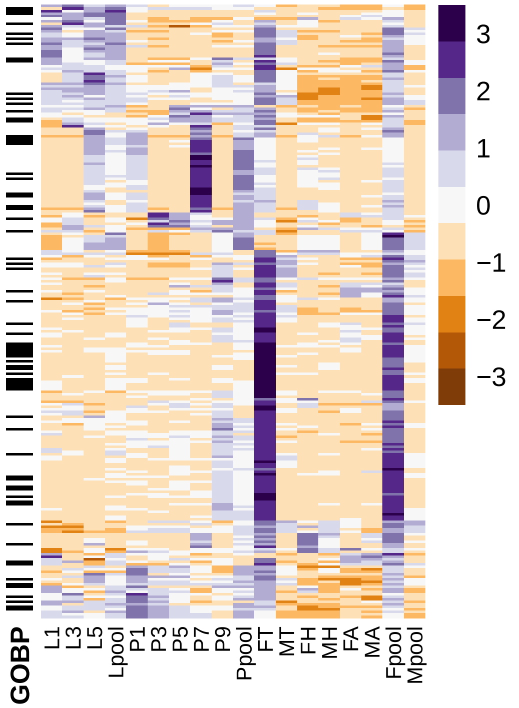

b Expression of all 1564 down genes

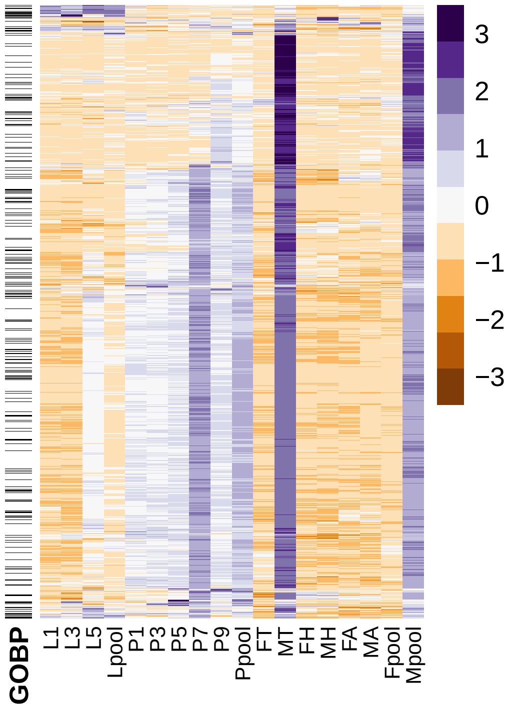

c Average expression of up genes

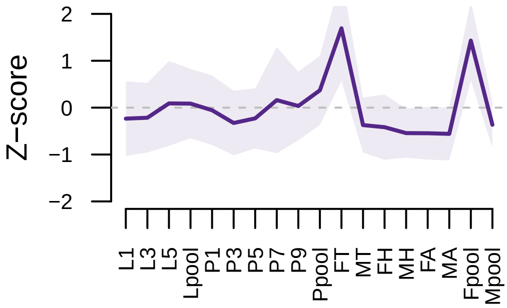

d Average expression of down genes

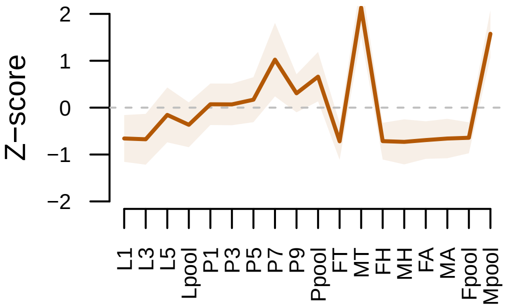

e GO enrichment of up genes (70/245)

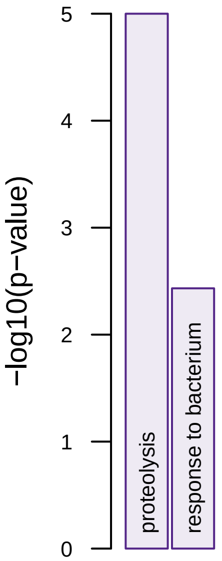

f GO enrichment of down genes (289/1564)

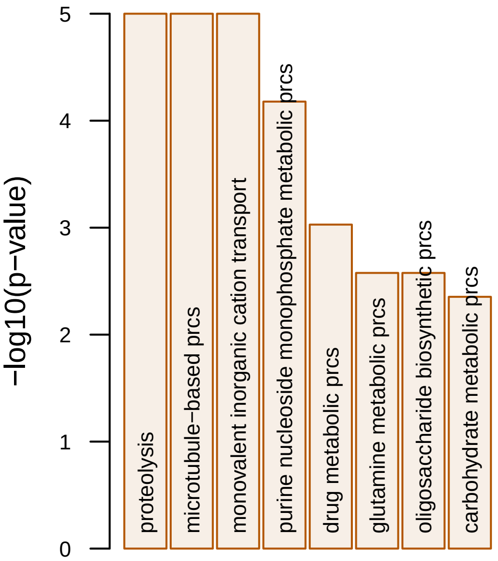

Supplementary Figure 18. *AdultParts:Other*

a Expression of all 1151 up genes

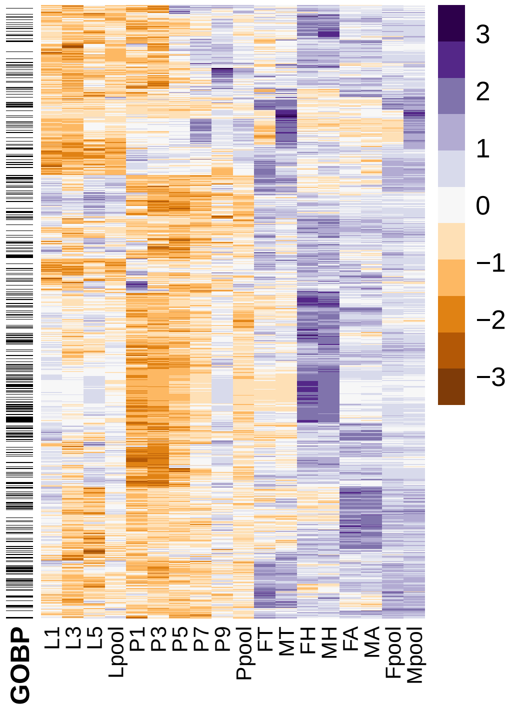

b Expression of all 1136 down genes

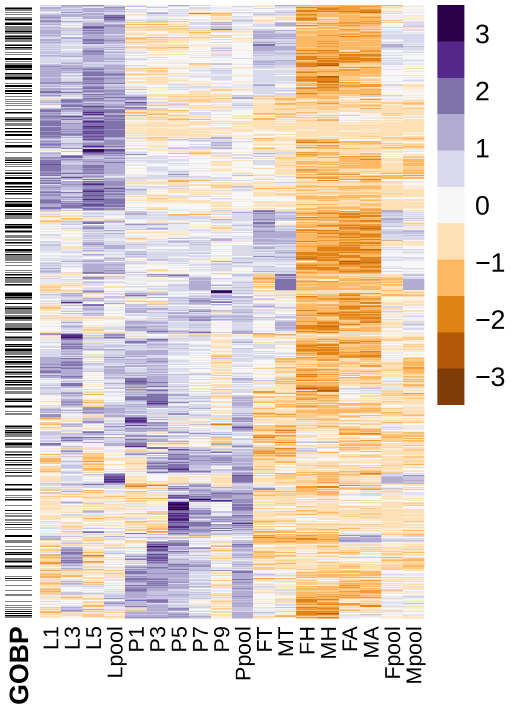

c Average expression of up genes

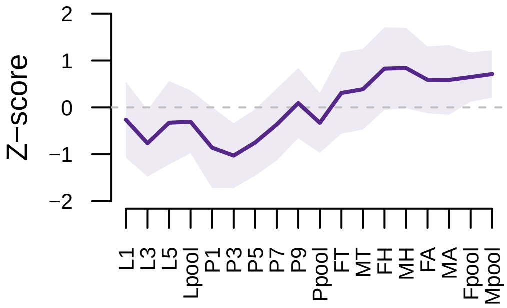

d Average expression of down genes

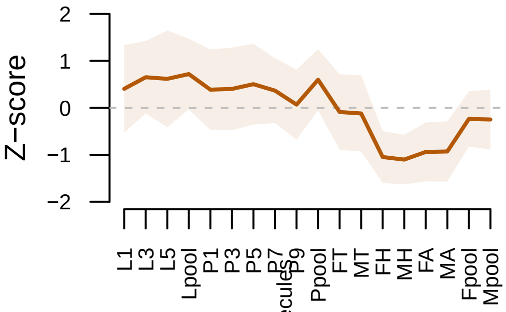

e GO enrichment of up genes (351/1151)

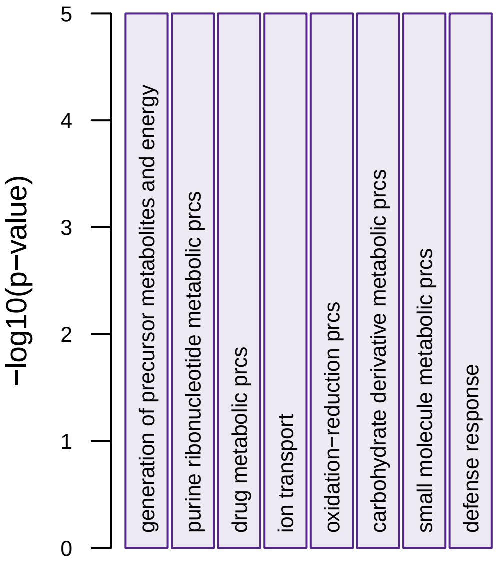

f GO enrichment of down genes (410/1136)

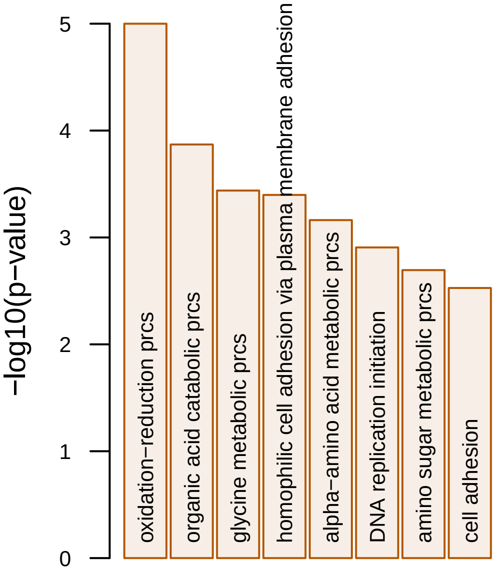

Supplementary Figure 18. *Adult:Other*

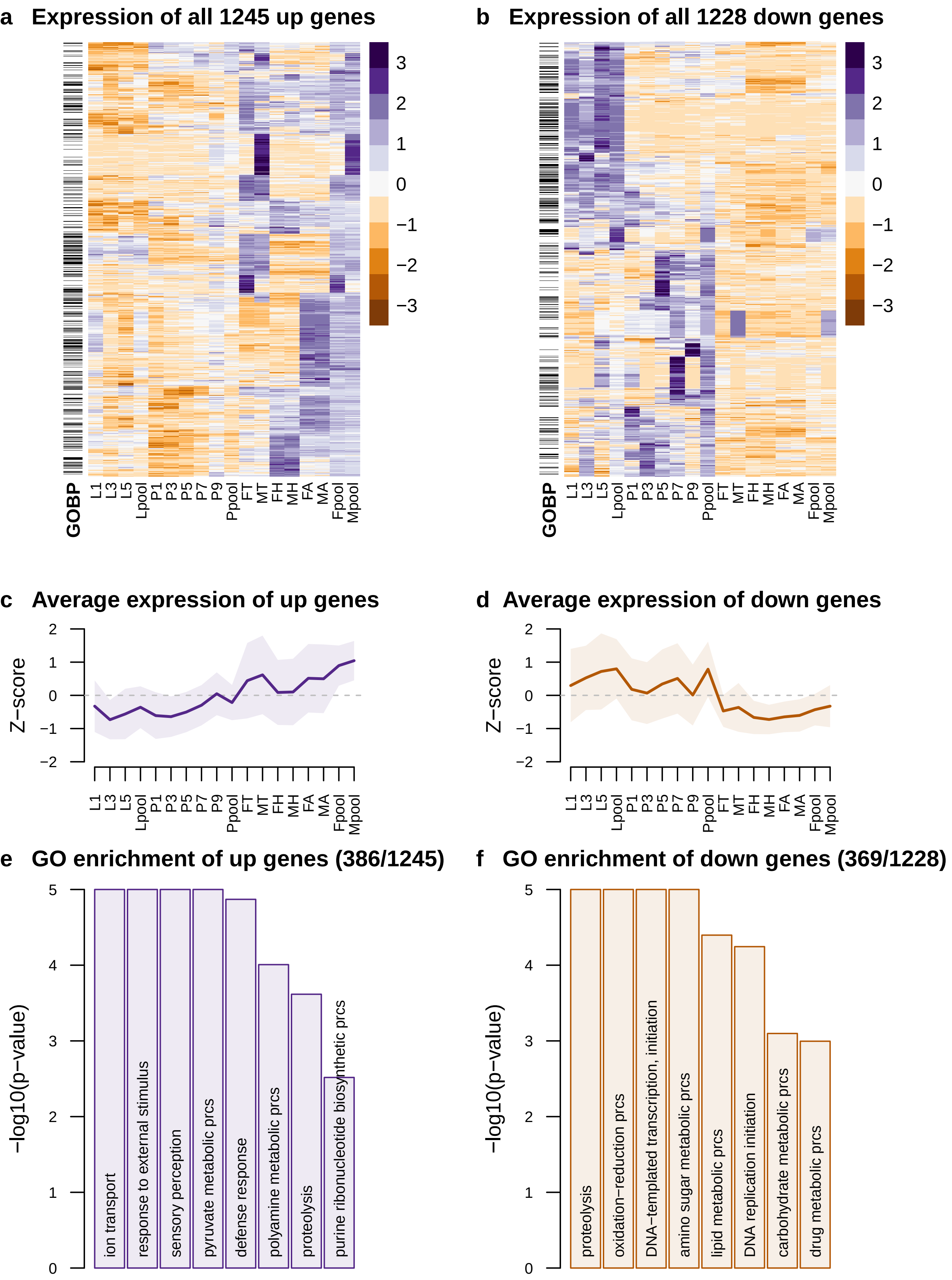

Supplementary Figure 18.

Source

a Expression of all 2489 up genes

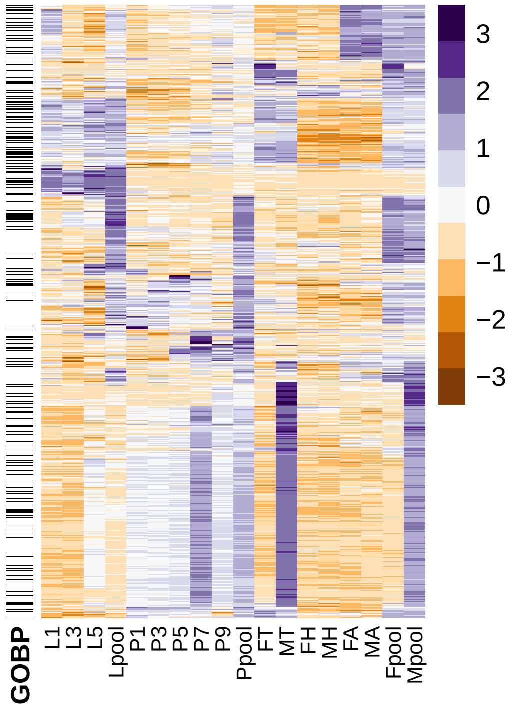

b Expression of all 85 down genes

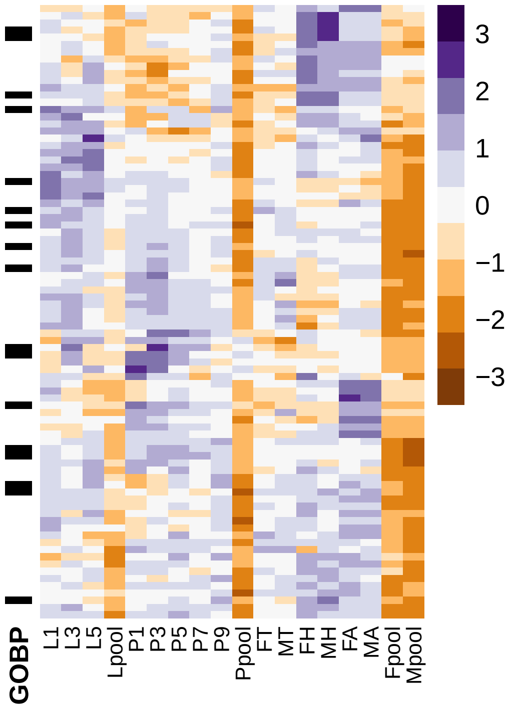

c Average expression of up genes

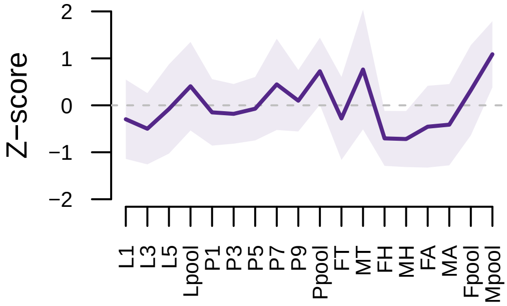

d Average expression of down genes

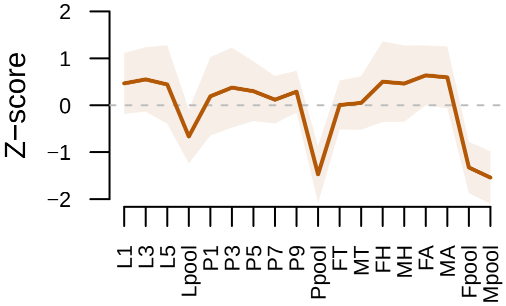

e GO enrichment of up genes (602/2489)

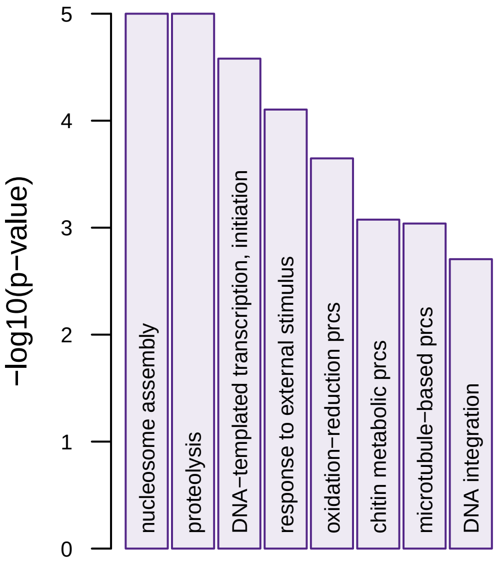

f GO enrichment of down genes (17/85)

Supplementary Figure 18. Differential expression results for 24 contrasts performed. The two samples compared in each contrast follow the format “condition 1: condition 2”. **a**-**b** Heatmaps of differentially upregulated and downregulated genes (5% FDR) in condition 1 relative to condition 2. The average of each pair of replicates is used for the columns, and the rows are scaled using a Z-score. **c-d** Average gene expression trend based on the Z-score scaled expression of all upregulated and downregulated genes across all samples, respectively. The shaded area represents ±1 SD from the mean. **e-f** Topmost significantly enriched Biological Process GO terms amongst upregulated and downregulated genes, respectively. Contributing genes to enrichment patterns are indicated in the leftmost column of **a-b**. Only GO terms with an associated q-value cutoff smaller or equal to 0.2 are plotted.

Supplementary Figure 19. *clust01*

a Expression of all 2374 genes

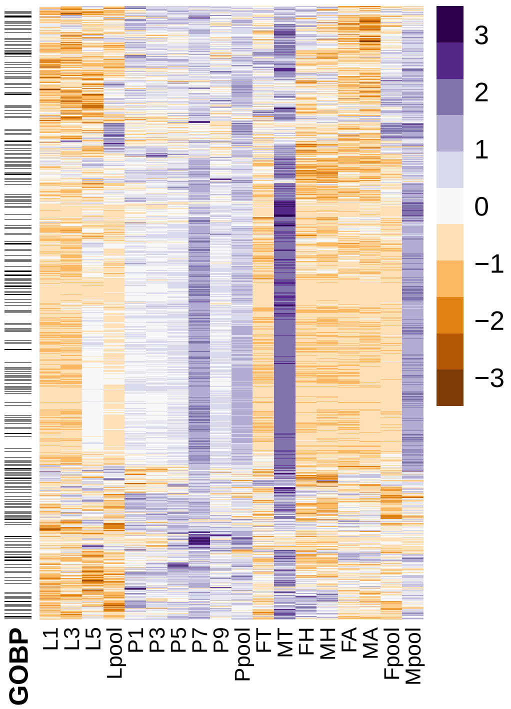

b Average expression of clustered genes

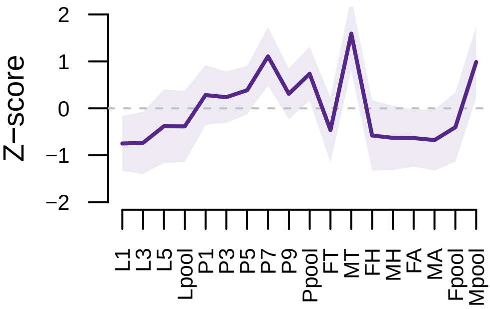

c GO enrichment of clustered genes (484/2374)

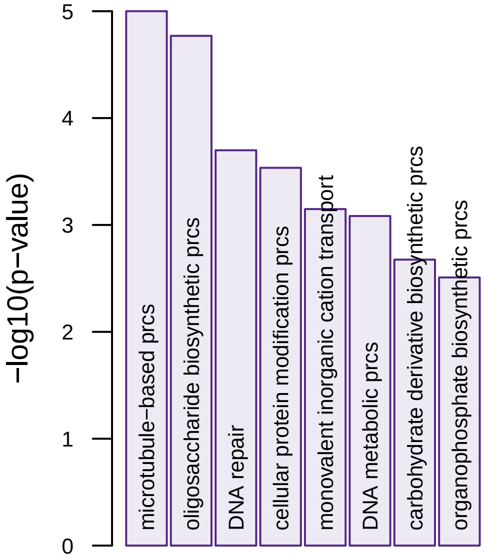

Supplementary Figure 19. *clust02*

a **Expression of all 2046 genes**

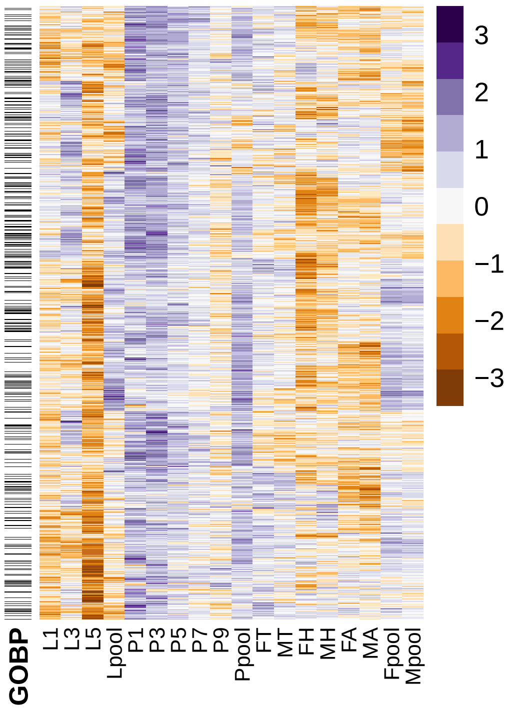

b **Average expression of clustered genes**

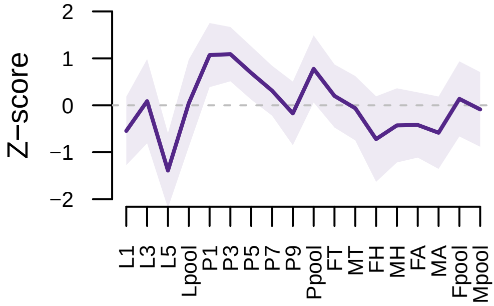

c **GO enrichment of clustered genes (514/2046)**

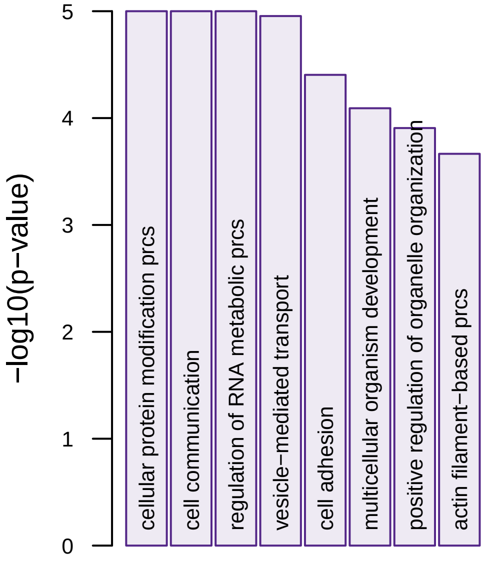

Supplementary Figure 19. *clust03*

a Expression of all 2022 genes

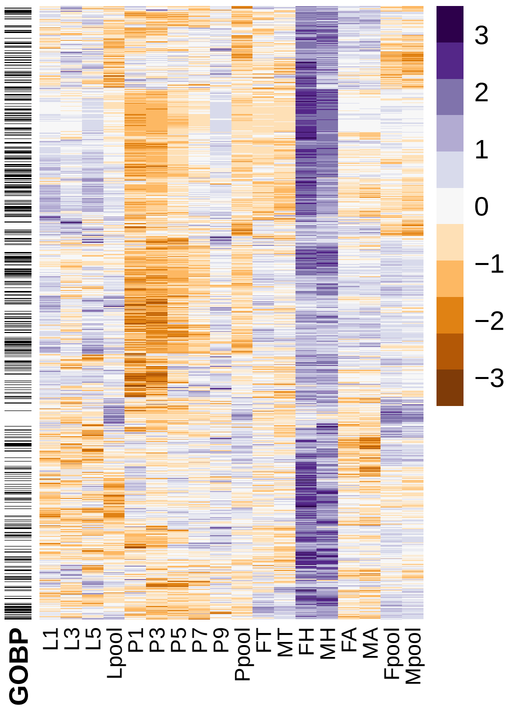

b Average expression of clustered genes

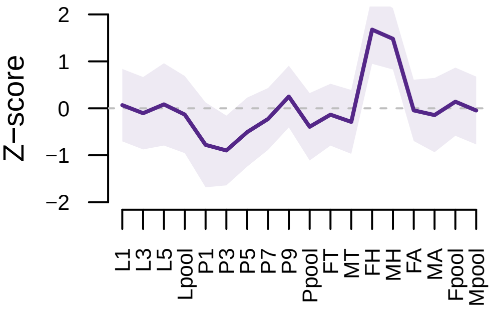

c GO enrichment of clustered genes (671/2022)

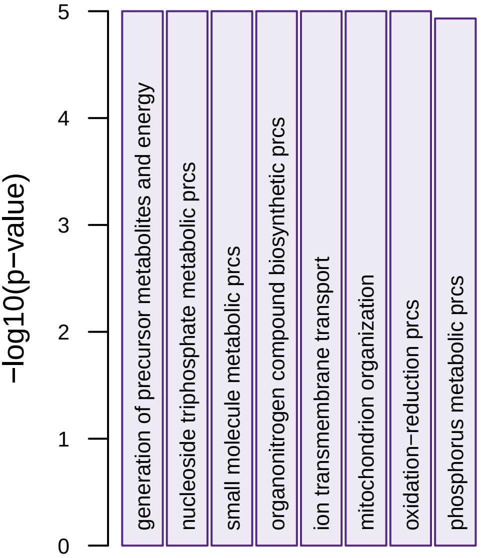

Supplementary Figure 19. *clust04*

a Expression of all 1926 genes

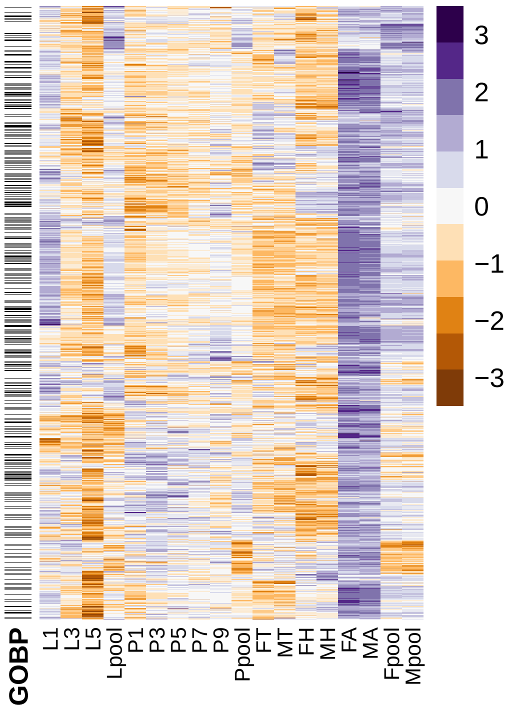

b Average expression of clustered genes

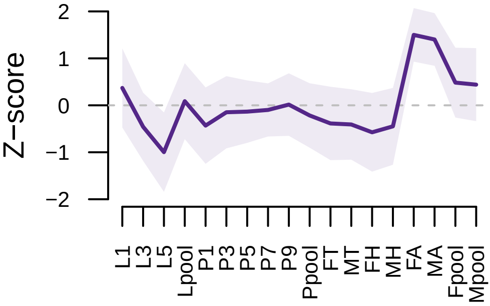

c GO enrichment of clustered genes (522/1926)

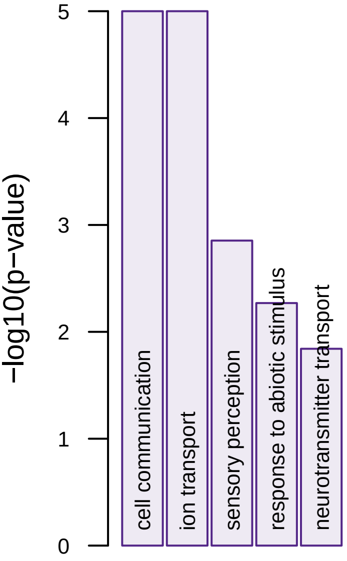

Supplementary Figure 19. *clust05*

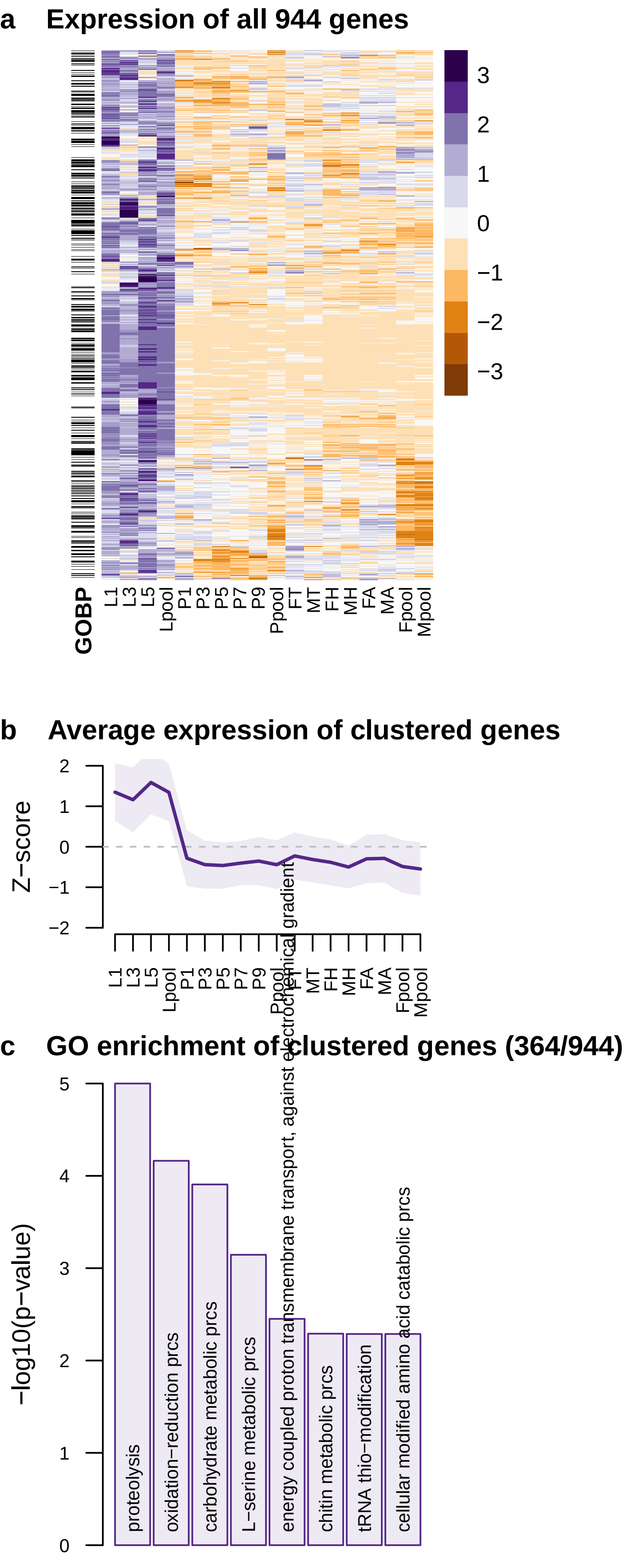

Supplementary Figure 19. *clust06*

a Expression of all 644 genes

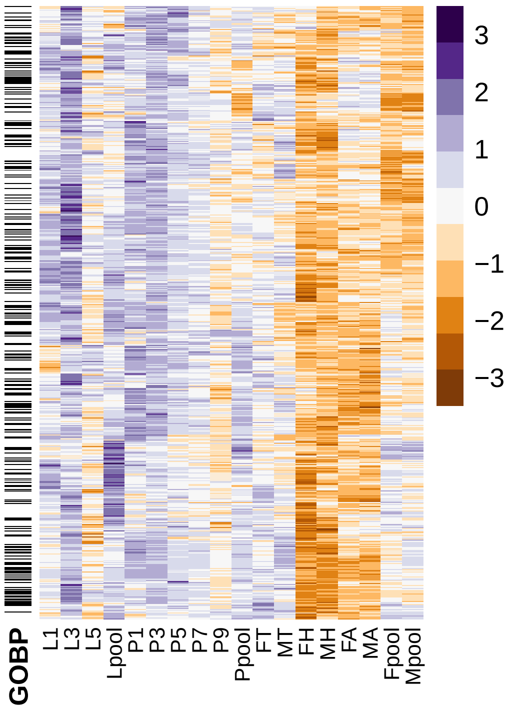

b Average expression of clustered genes

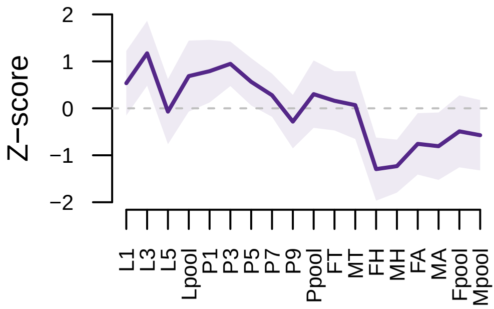

c GO enrichment of clustered genes (232/644)

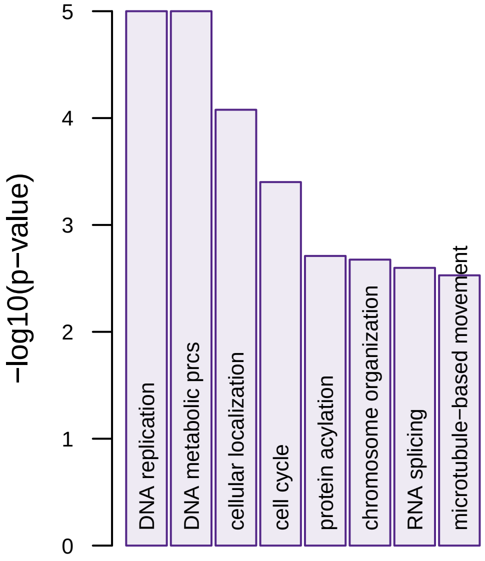

Supplementary Figure 19. *clust07*

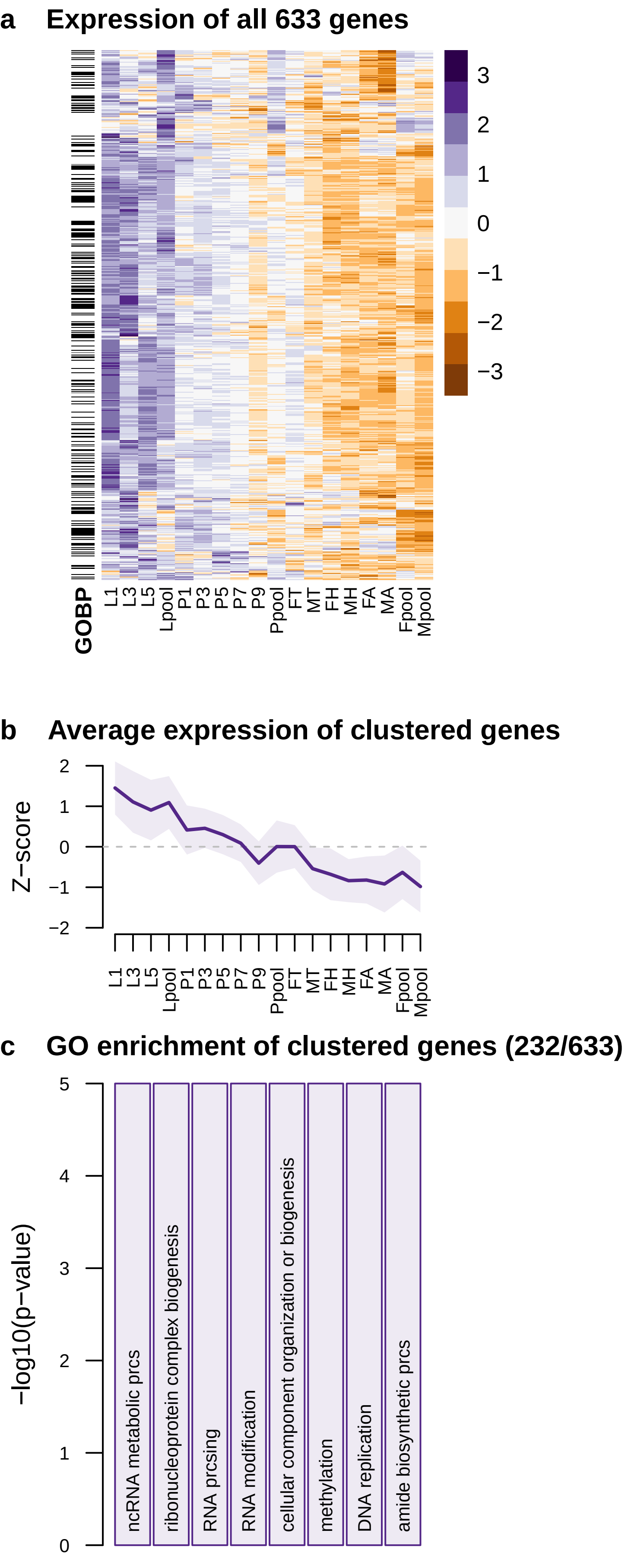

Supplementary Figure 19. *clust08*

a **Expression of all 519 genes**

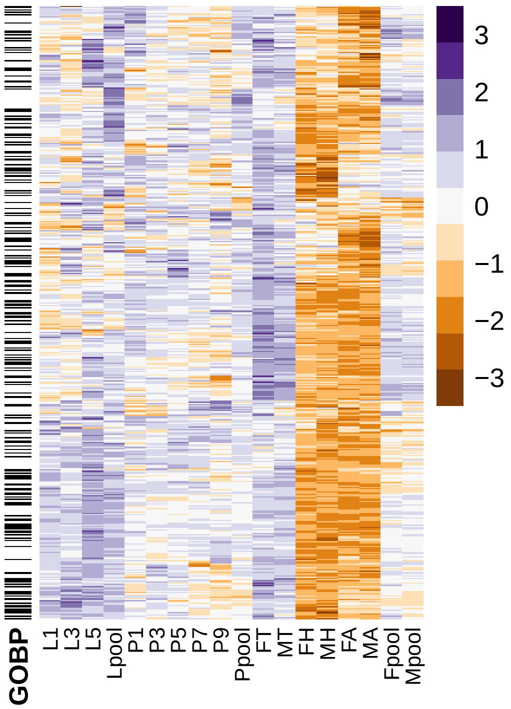

b **Average expression of clustered genes**

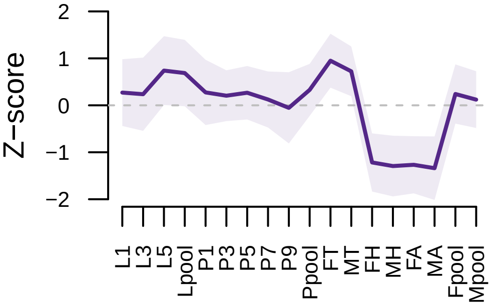

c **GO enrichment of clustered genes (190/519)**

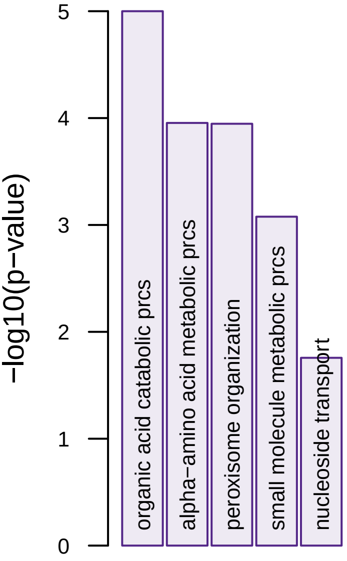

Supplementary Figure 19. *clust09*

a **Expression of all 504 genes**

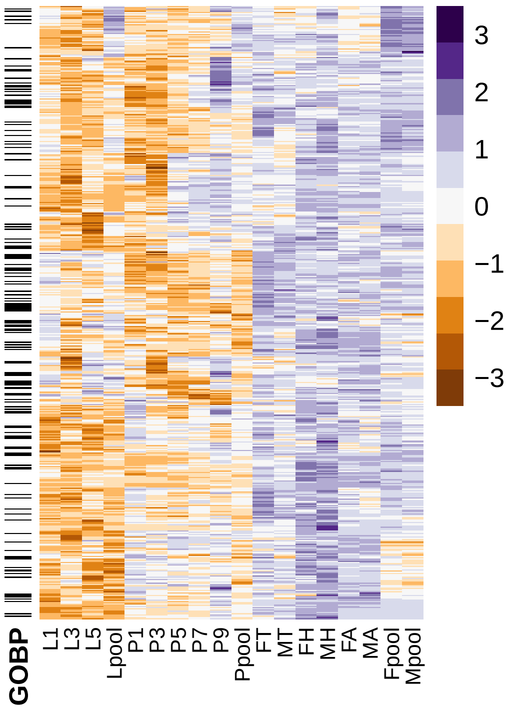

b **Average expression of clustered genes**

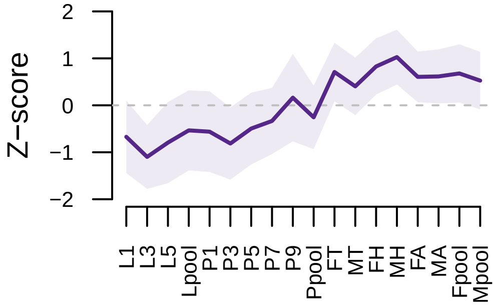

c **GO enrichment of clustered genes (137/504)**

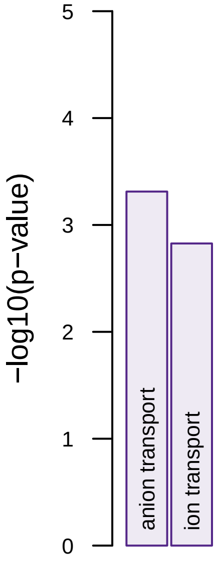

Supplementary Figure 19. *clust10*

a Expression of all 429 genes

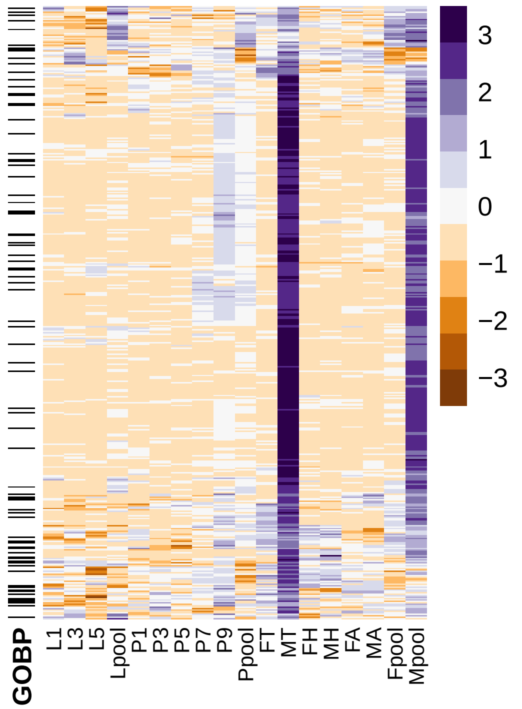

b Average expression of clustered genes

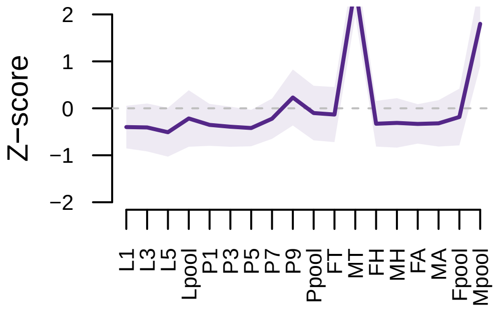

c GO enrichment of clustered genes (81/429)

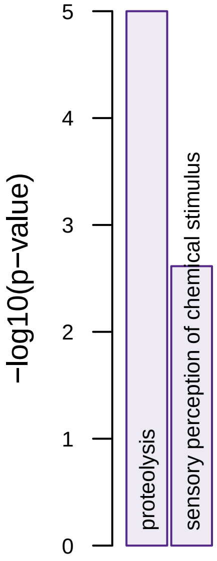

Supplementary Figure 19. *clust11*

a Expression of all 390 genes

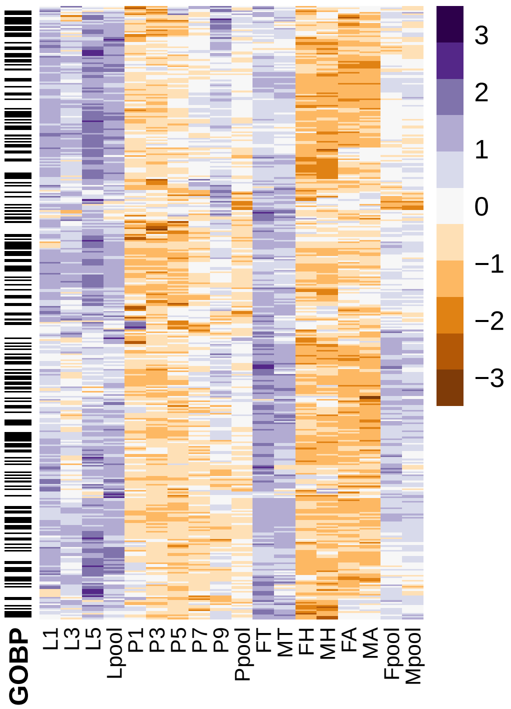

b Average expression of clustered genes

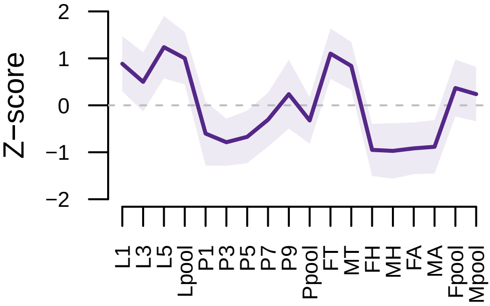

c GO enrichment of clustered genes (178/390)

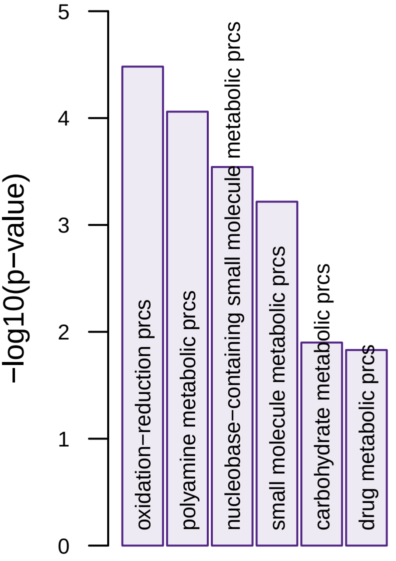

Supplementary Figure 19. *clust12*

a Expression of all 384 genes

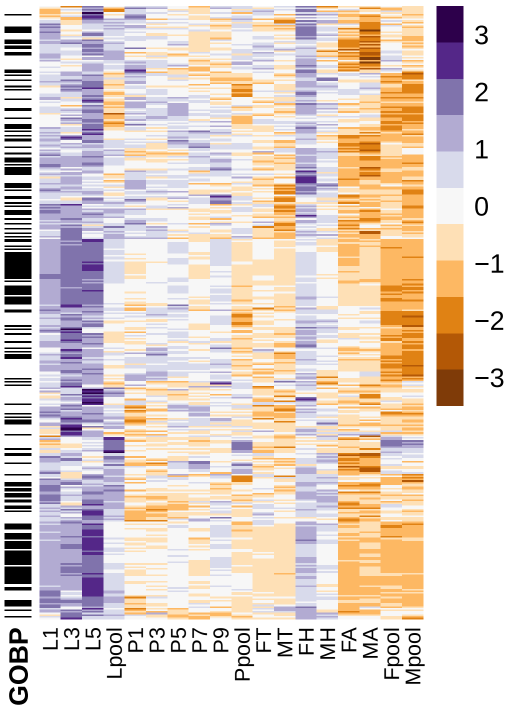

b Average expression of clustered genes

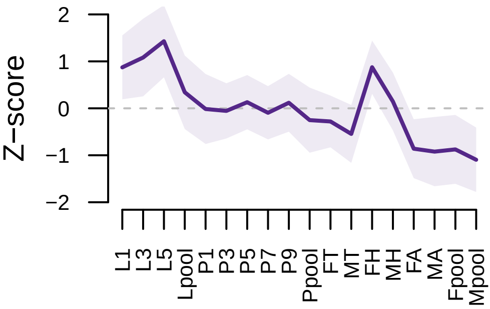

c GO enrichment of clustered genes (169/384)

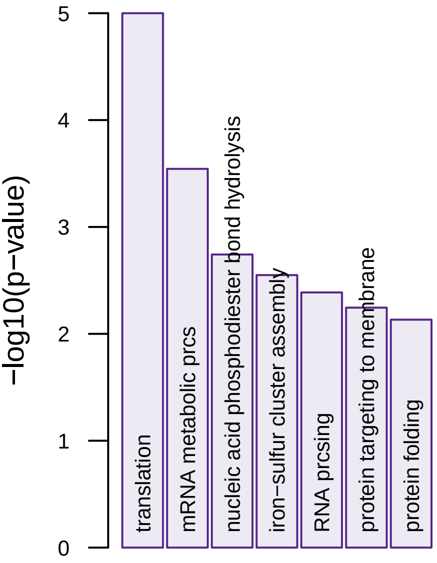

Supplementary Figure 19. *clust13*

a Expression of all 318 genes

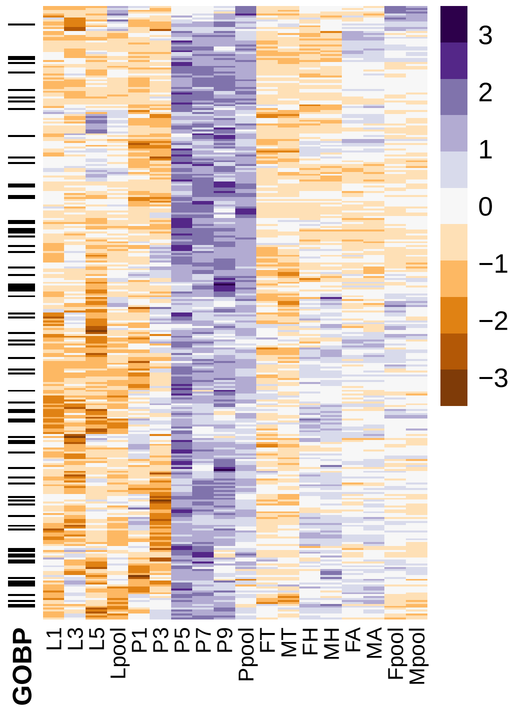

b Average expression of clustered genes

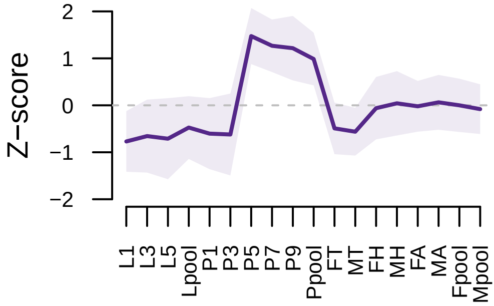

c GO enrichment of clustered genes (72/318)

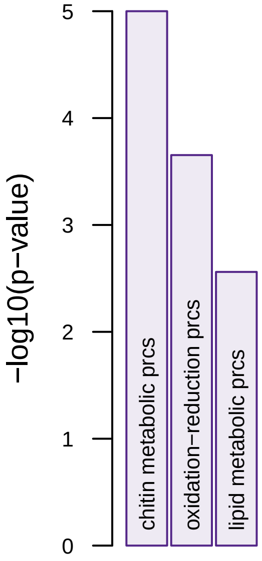

Supplementary Figure 19. *clust14*

a **Expression of all 265 genes**

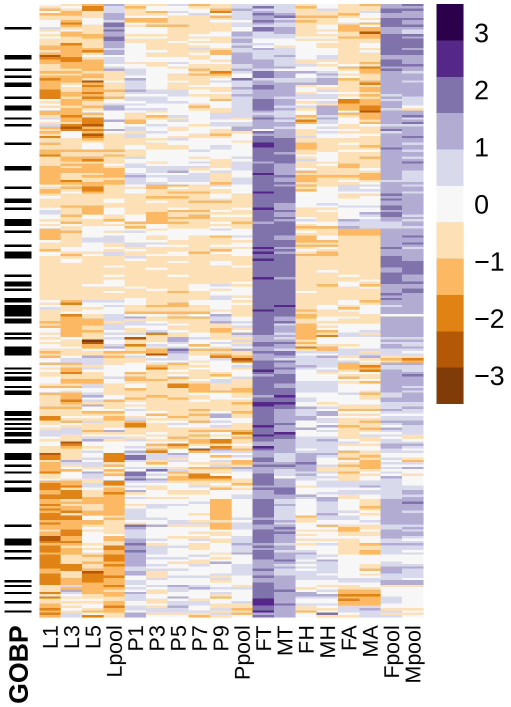

b **Average expression of clustered genes**

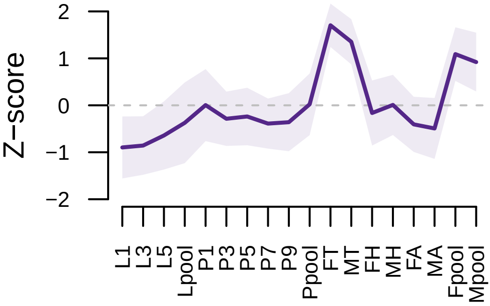

c **GO enrichment of clustered genes (81/265)**

Supplementary Figure 19. ***clust15***

a Expression of all 215 genes

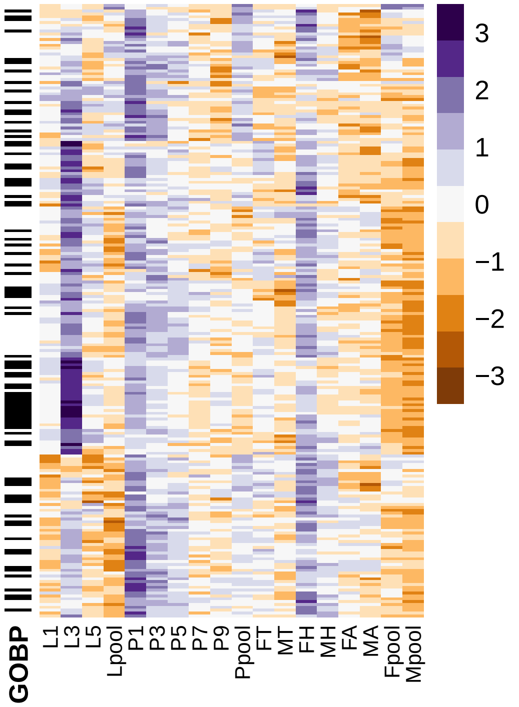

b Average expression of clustered genes

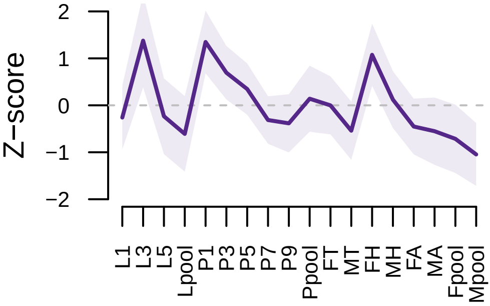

c GO enrichment of clustered genes (81/215)

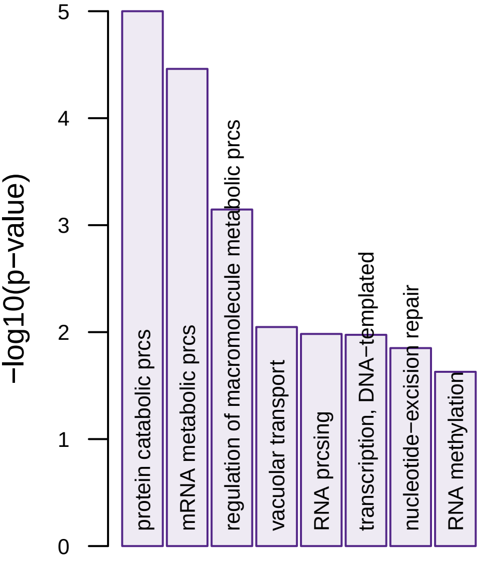

Supplementary Figure 19. *clust16*

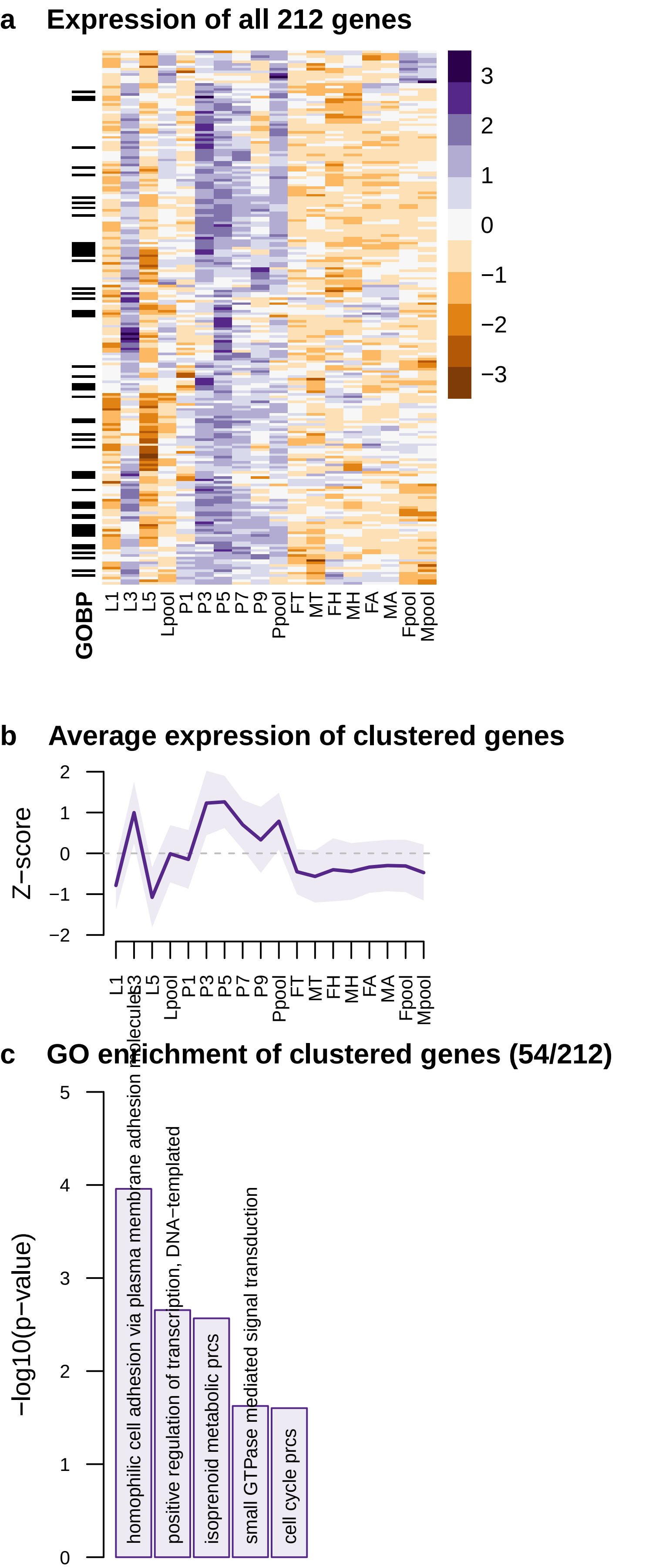

Supplementary Figure 19. *clust17*

a Expression of all 187 genes

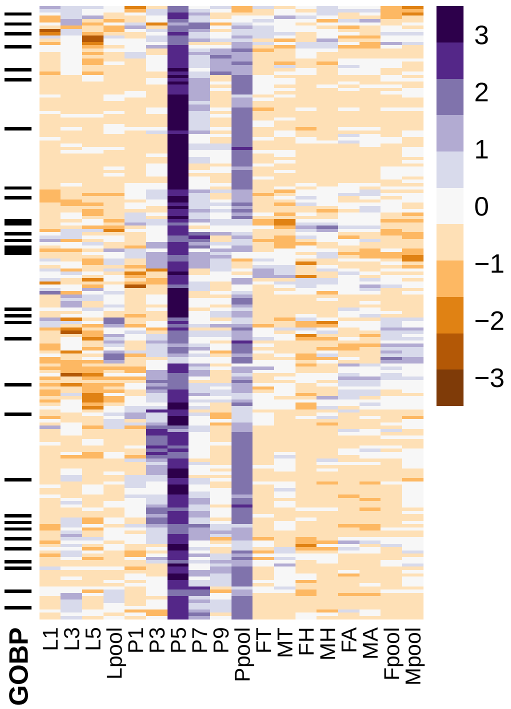

b Average expression of clustered genes

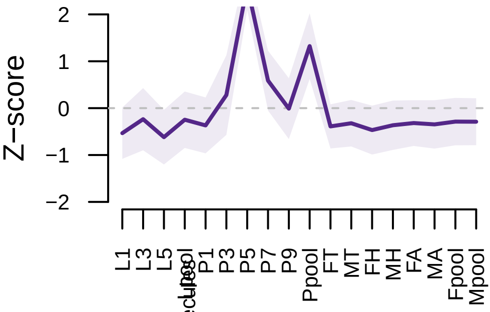

c GO enrichment of clustered genes (32/187)

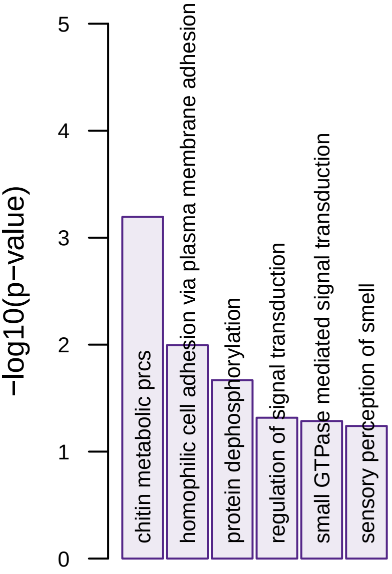

Supplementary Figure 19. *clust18*

a Expression of all 172 genes

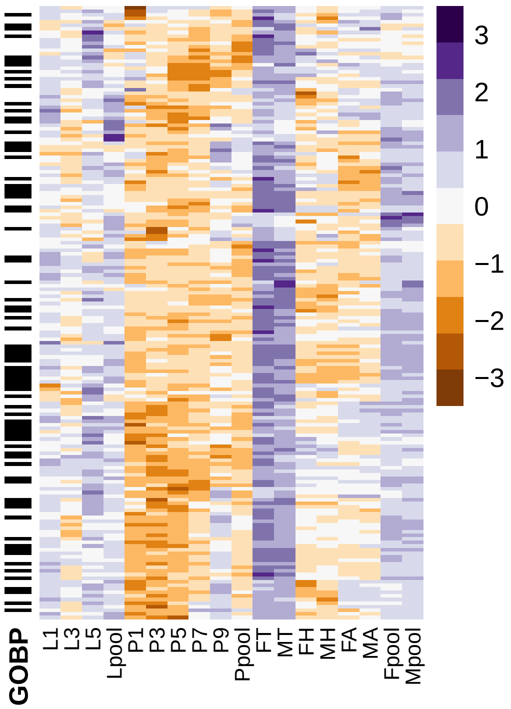

b Average expression of clustered genes

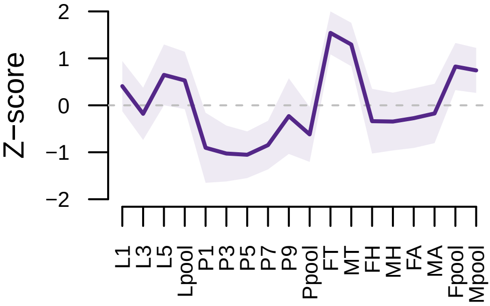

c GO enrichment of clustered genes (77/172)

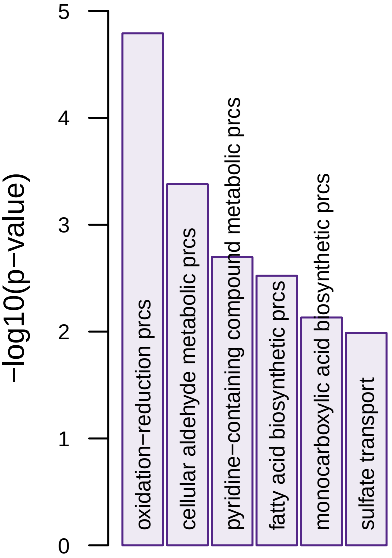

Supplementary Figure 19. *clust19*

a **Expression of all 147 genes**

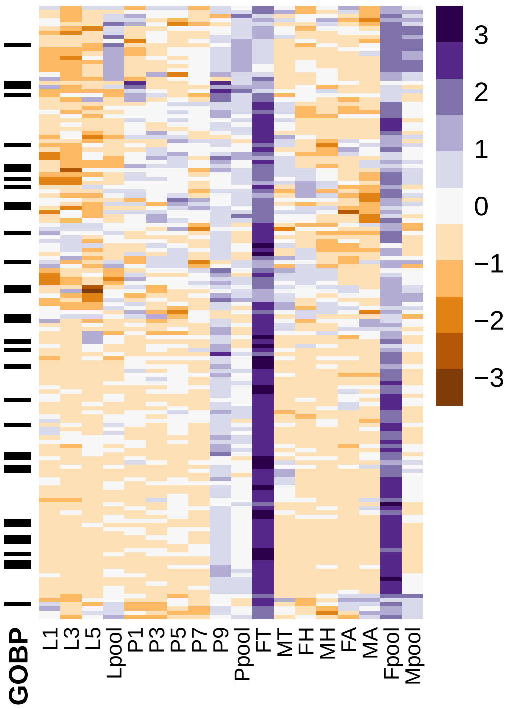

b **Average expression of clustered genes**

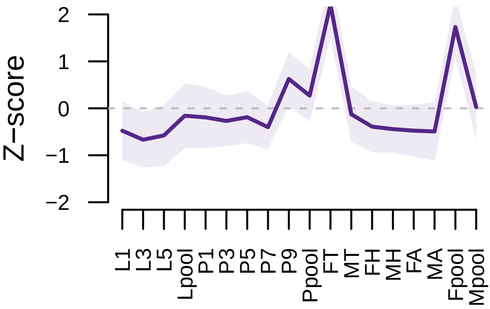

c **GO enrichment of clustered genes (34/147)**

Supplementary Figure 19. *clust20*

a Expression of all 109 genes

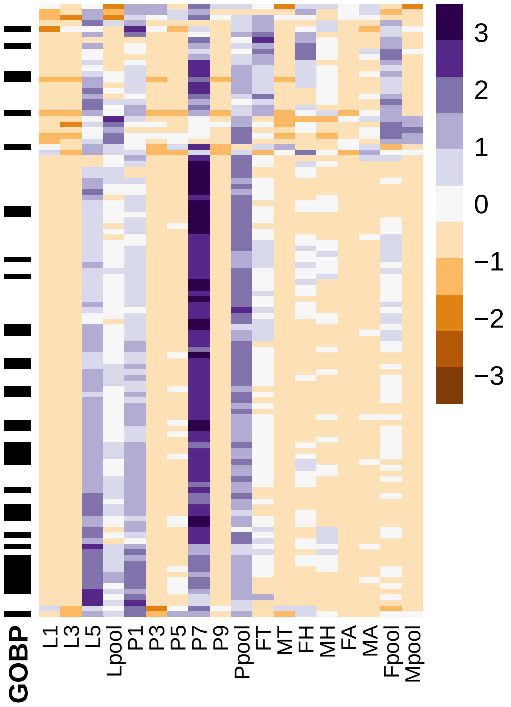

b Average expression of clustered genes

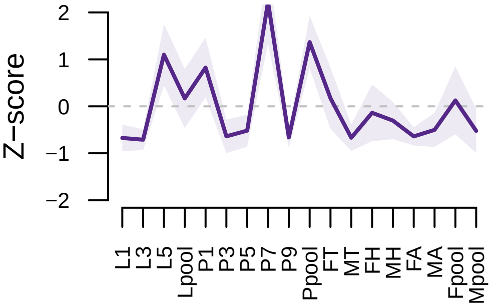

c GO enrichment of clustered genes (36/109)

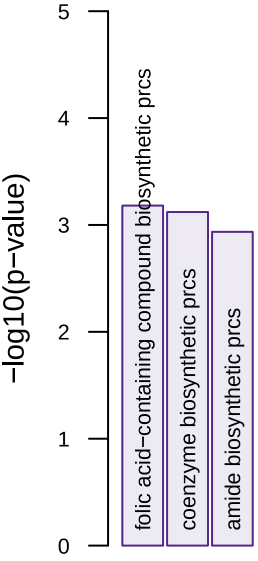

Supplementary Figure 19. *clust21*

a Expression of all 80 genes

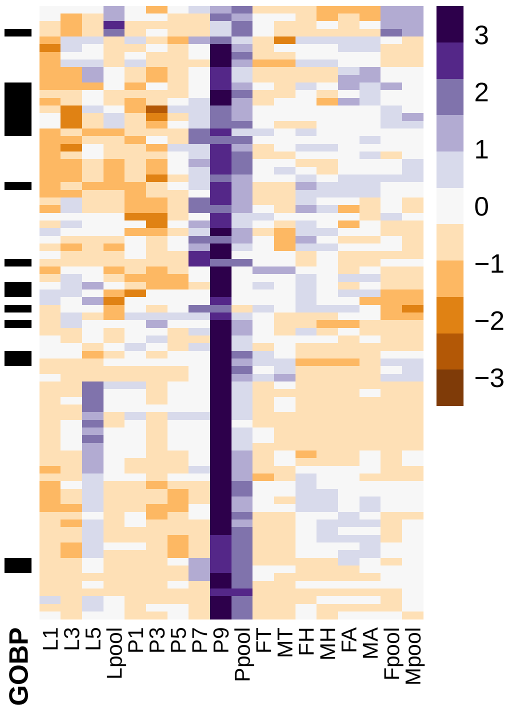

b Average expression of clustered genes

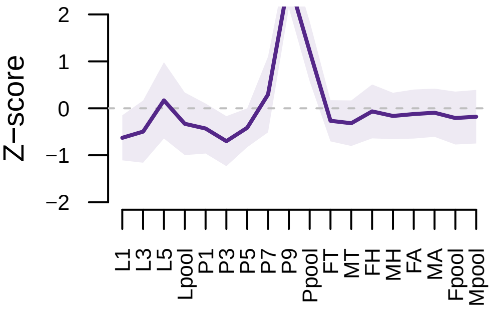

c GO enrichment of clustered genes (18/80)

Supplementary Figure 19. *clust22*

a Expression of all 66 genes

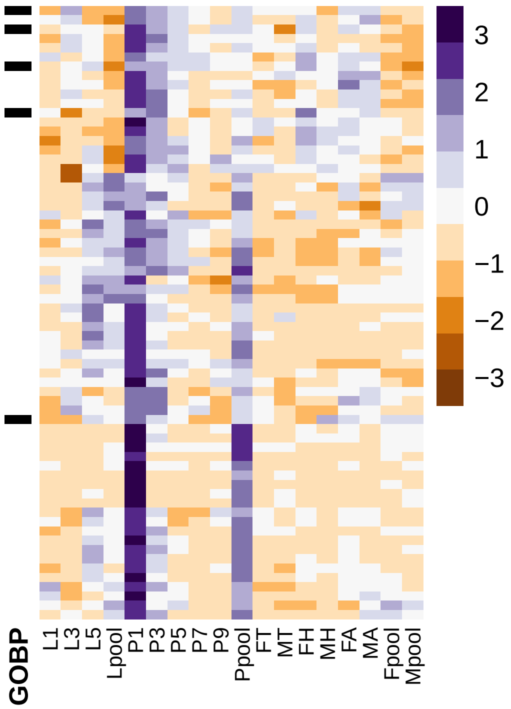

b Average expression of clustered genes

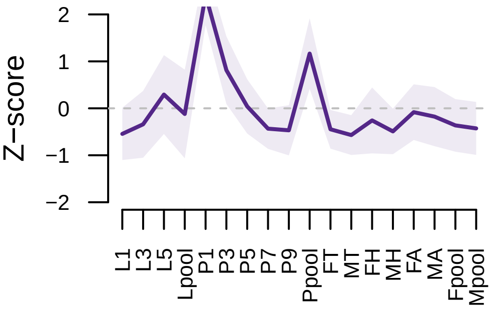

c GO enrichment of clustered genes (5/66)

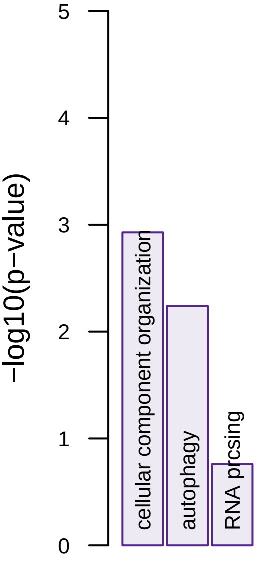

Supplementary Figure 19. *clust23*

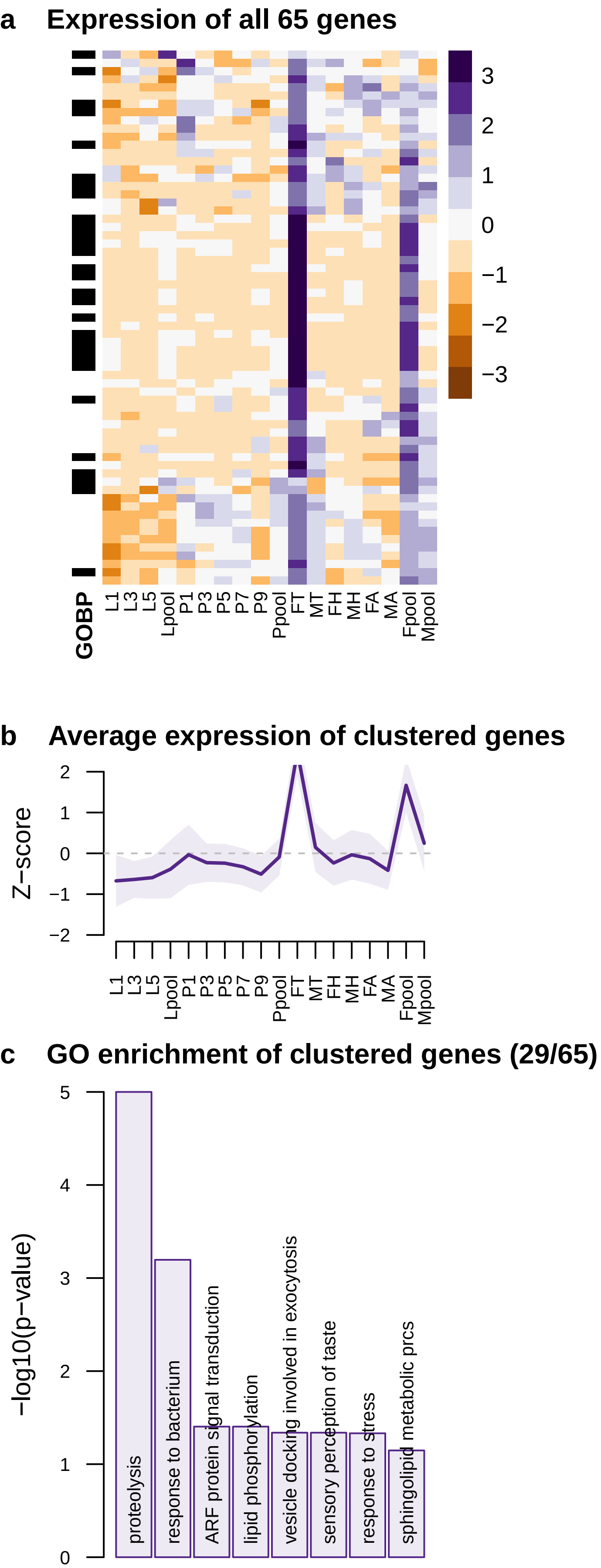

Supplementary Figure 19. *clust24*

a Expression of all 64 genes

b Average expression of clustered genes

c GO enrichment of clustered genes (8/64)

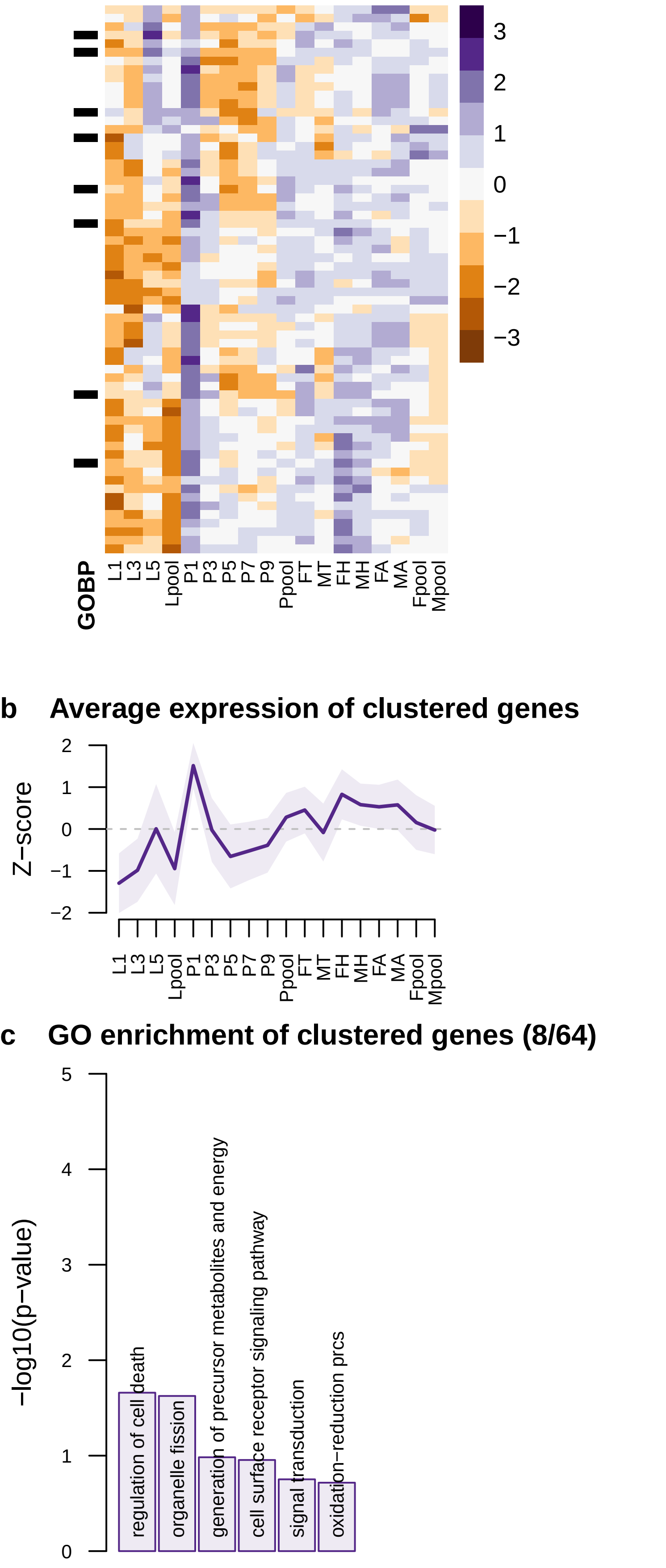

Supplementary Figure 19. *clust25*

a Expression of all 43 genes

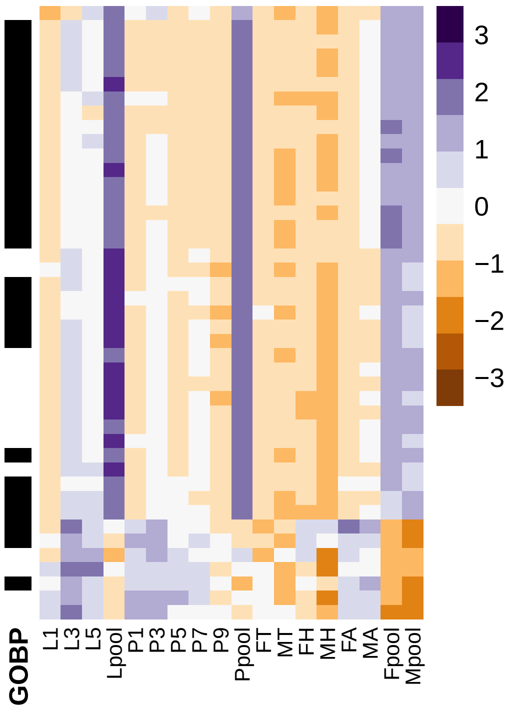

b Average expression of clustered genes

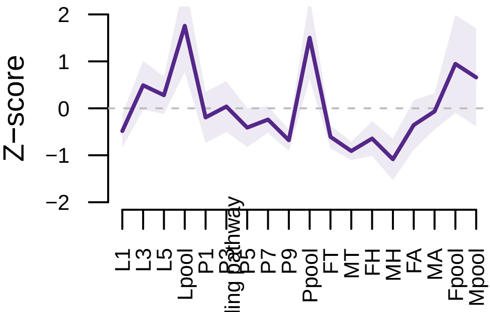

c GO enrichment of clustered genes (28/43)

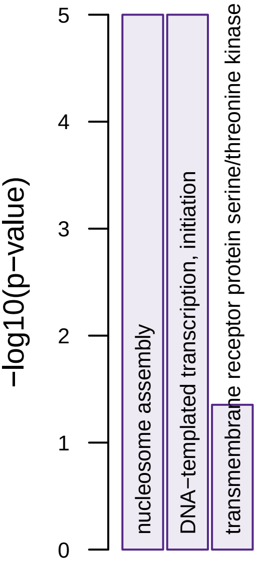

Supplementary Figure 19. *clust26*

a Expression of all 37 genes

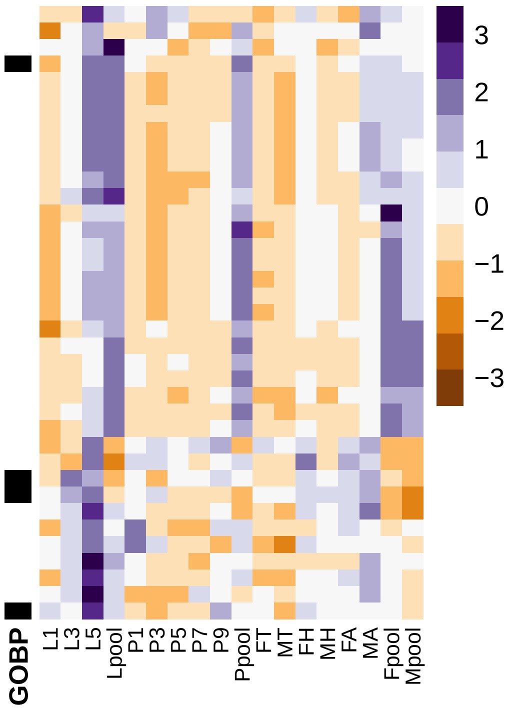

b Average expression of clustered genes

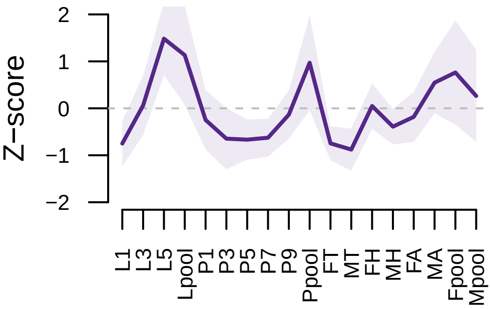

c GO enrichment of clustered genes (4/37)

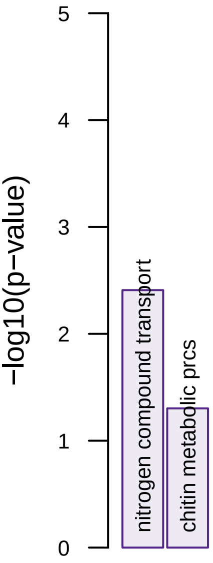

Supplementary Figure 19. ***clust27***

a Expression of all 26 genes

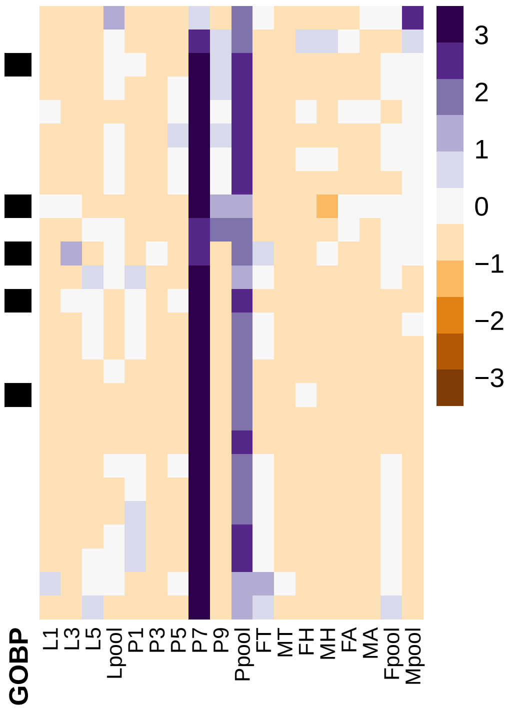

b Average expression of clustered genes

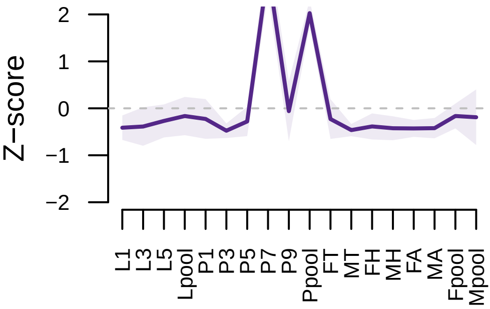

c GO enrichment of clustered genes (5/26)

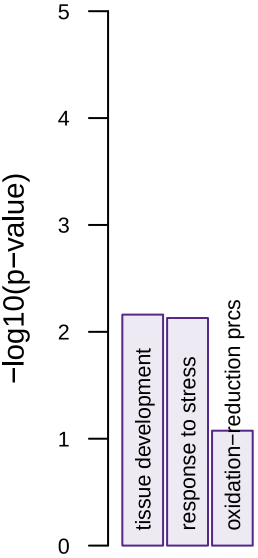

Supplementary Figure 19. Expression attributes of the 25 clusters delineated with Weighted Gene Correlation Network Analysis (WGCNA). a Heatmap of the genes associated with the cluster in question across the sample types assayed. The average of each pair of replicates for each sample type is used for the columns, and the rows are scaled using a Z-score. **b** Average gene expression trend based on the Z-score scaled expression of all genes in the cluster. The shaded area represents ±1 SD from the mean. **c** Topmost significantly enriched Biological Process GO terms amongst the genes in cluster. Contributing genes to enrichment patterns are indicated in the leftmost column of **a**. Only GO terms with an associated q-value cutoff smaller or equal to 0.2 are plotted.

437 Female-biased genes, FDR <5% & FC >2 **1747 Male-enriched genes, FDR <5% & FC >2**

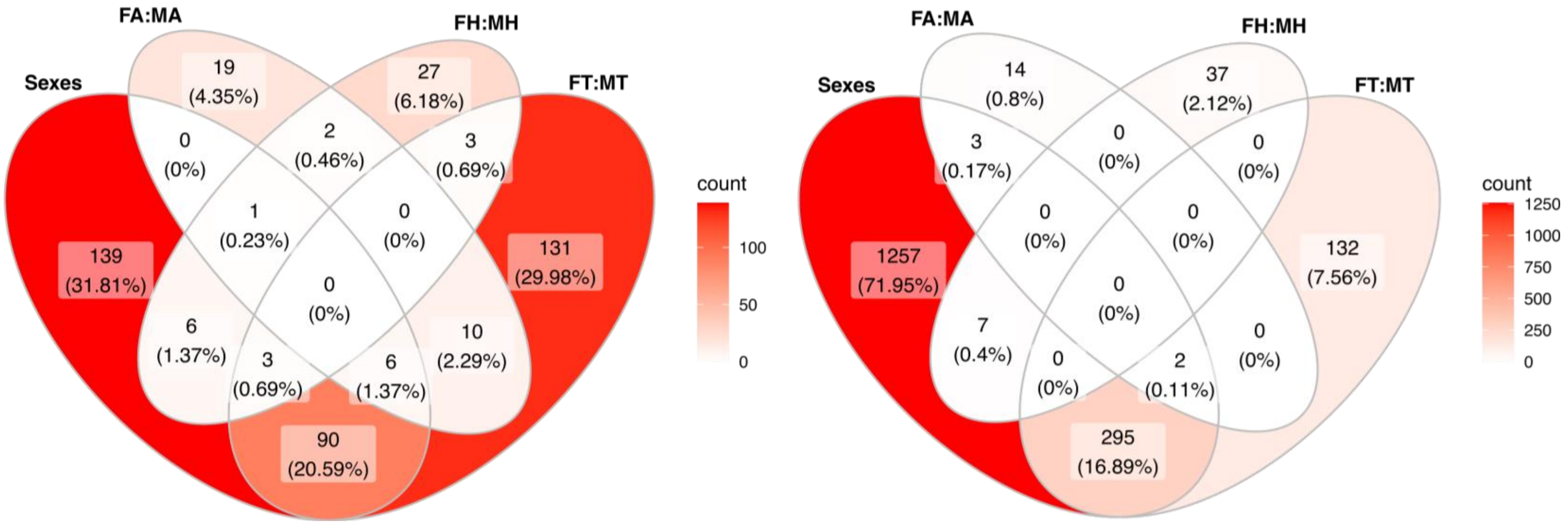

Supplementary Figure 20. Venn diagrams showing the degree of overlap of genes determined to be sex-biased across different types of adult samples. Samples assayed: pools of whole-body males and females (Sexes: Fpool vs Mpool); individual samples of thorax (FT:MT), abdomen (FA:MA), and heads (FH:MH). Differential expression between the sexes was deemed based on a higher than a 2-fold expression difference at a 5% FDR. Most female-biased genes are identified in the Sexes and FT:MT contrasts, while male-biased genes are mostly identified in the *Sexes* contrast.

Supplementary Figure 21. Distribution of expression of all genes on autosomes or sex chromosomes, according to tissue (FPKM > 0.01)

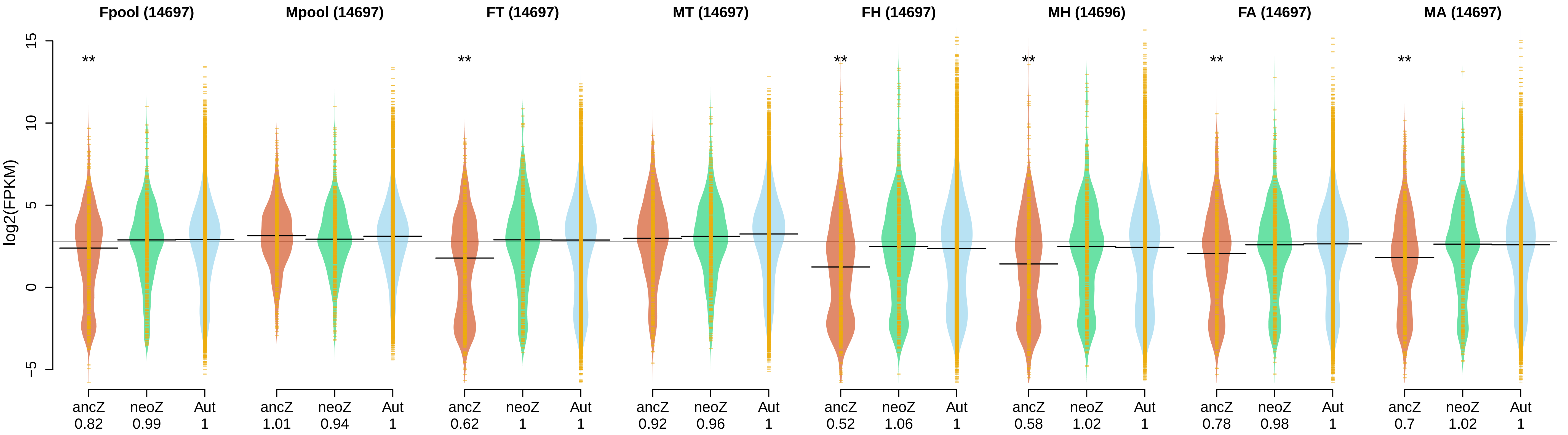

Wilcoxon test: are expression values on sex−chromosome lower than autosomes? Bonferroni−adjusted: ** p < 0.01, * p < 0.05

Supplementary Figure 21. Distribution of expression of all genes on autosomes or sex chromosomes, according to tissue (FPKM > 0.1)

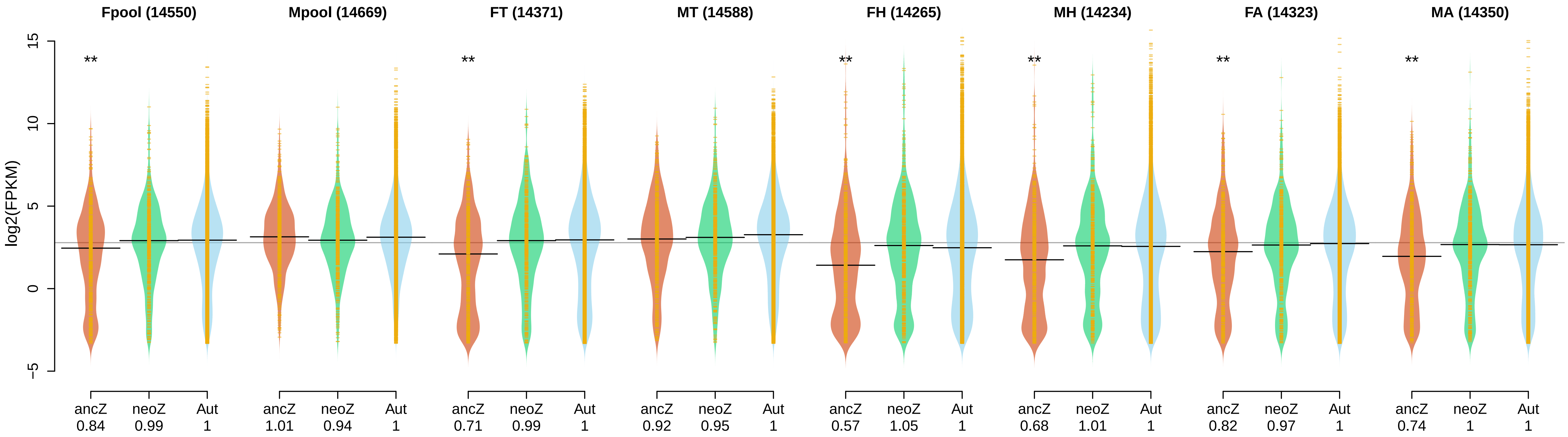

Wilcoxon test: are expression values on sex−chromosome lower than autosomes? Bonferroni−adjusted: ** p < 0.01, * p < 0.05

Supplementary Figure 21. Distribution of expression of all genes on autosomes or sex chromosomes, according to tissue (FPKM > 0.5)

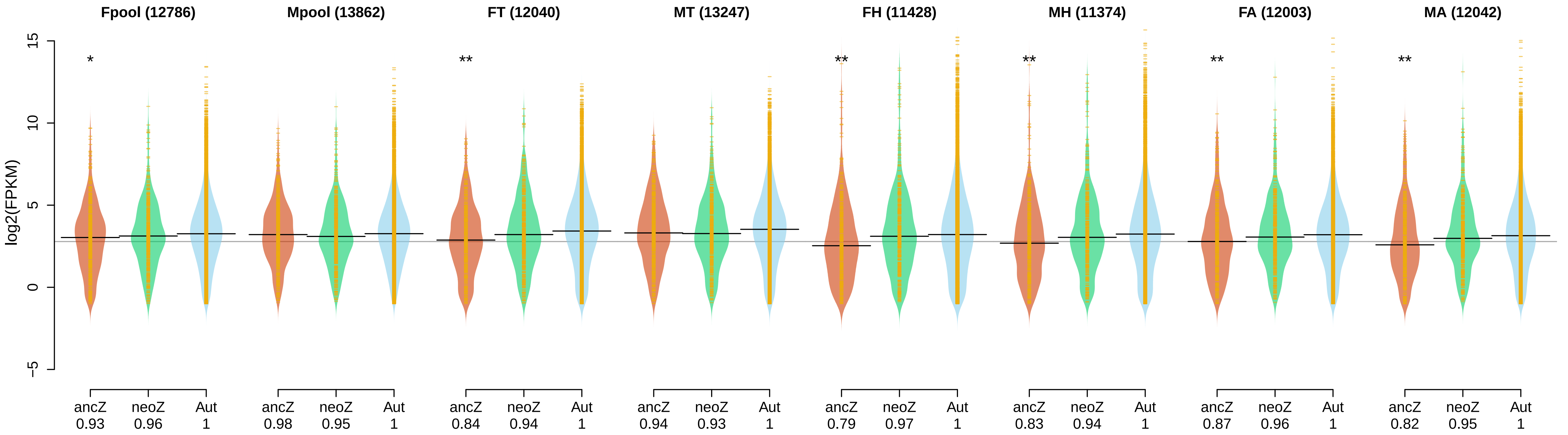

Wilcoxon test: are expression values on sex−chromosome lower than autosomes? Bonferroni−adjusted: ** $p < 0.01$, * $p < 0.05$

Supplementary Figure 21. Distribution of expression of all genes on autosomes or sex chromosomes, according to tissue (FPKM > 1)

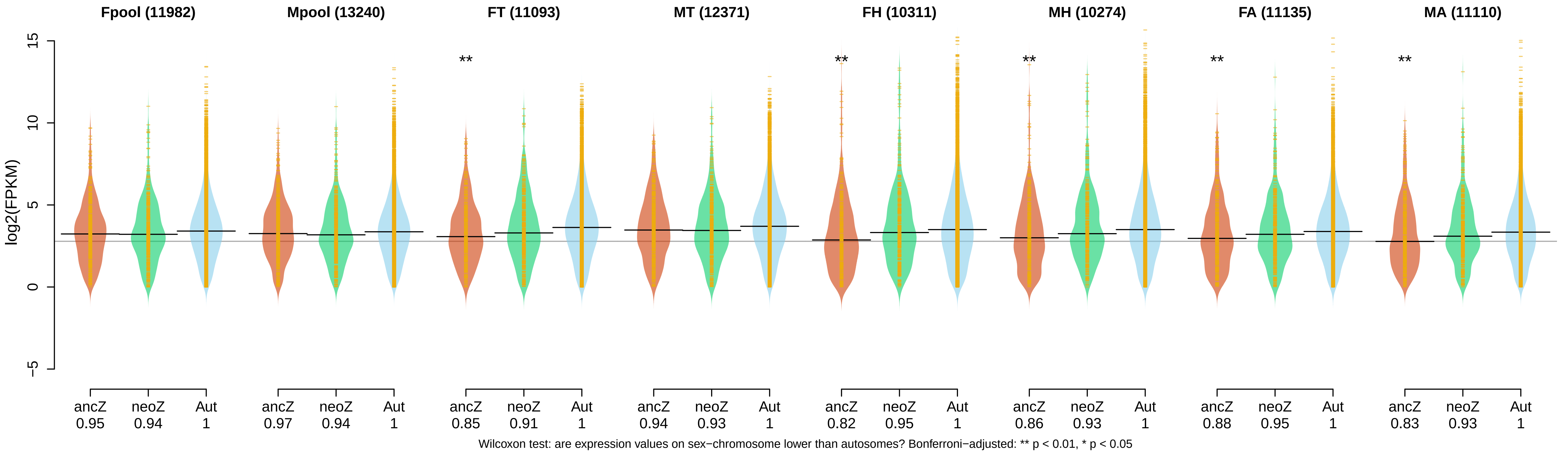

Supplementary Figure 21. Whole-chromosome expression in females (*ZW*) and males (*ZZ*) in four sample types of *D. plexippus* across four thresholds of minimum expression. The four minimum expression thresholds considered are: 0.01 FPKM, 0.1 FPKM, 0.5 FPKM, and 1 FPKM. Sampled assayed: pools of whole-body males and females (Mpool, Fpool); individual samples of thorax (MT, FT), abdomen (MA, FA), and heads (MH, FH). The bean plots show the distribution of absolute normalized log_2 expression values in FPKM for the ancestral (anc-) and neo (neo-) portions of chromosome *1* (*i.e.* the heterochromosome *Z*) and the autosomes. The horizontal line in each plot corresponds to the median expression value. A global median value across sample types is shown with a grey line in the background. The median *Z*:*A* ratios are shown at the bottom. For each sample type, statistical significance was established according to Wilcoxon signed-rank tests and upon applying the Bonferroni correction. The number of genes considered is indicated on top of each bean plot.

Supplementary Figure 22. Distribution of expression of all genes on each chromosome, according to tissue (FPKM > 0.01

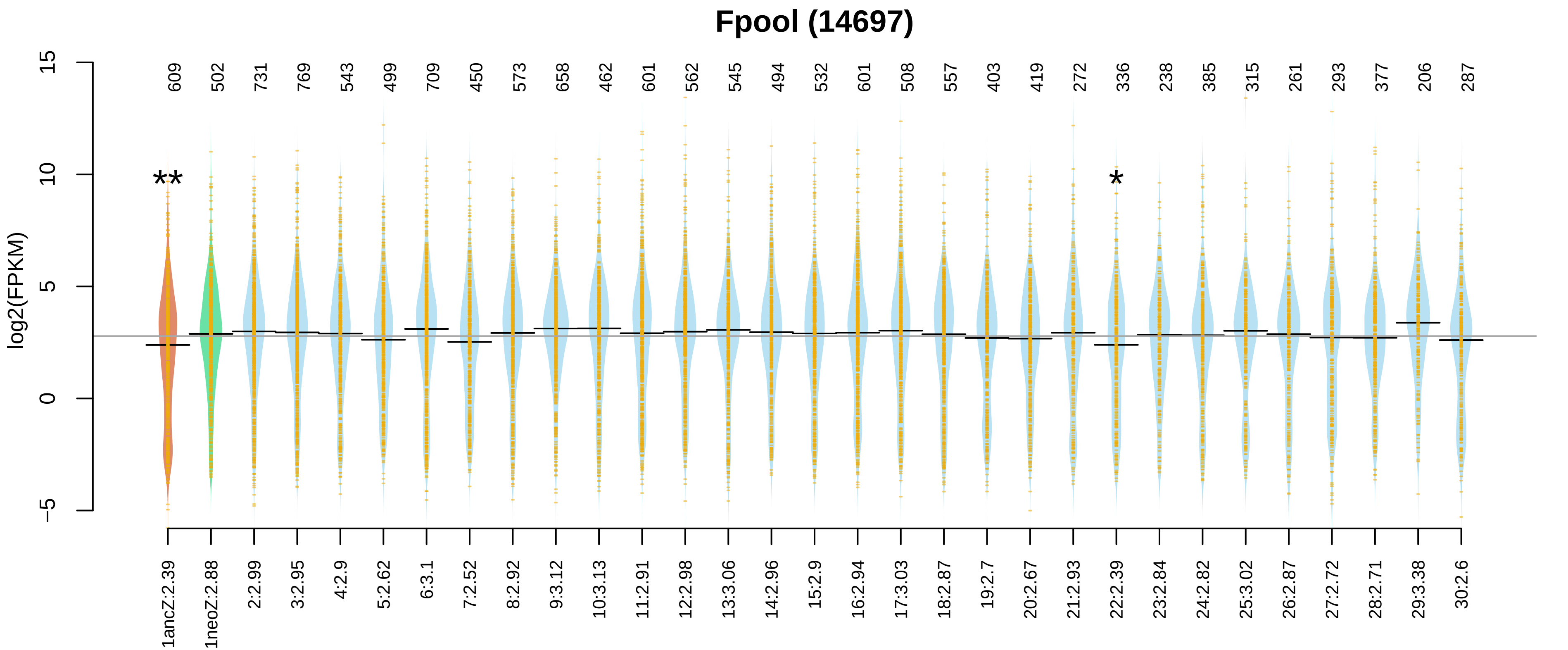

Wilcoxon test: are expression values on each chromosome different than the rest? Bonferroni−adjusted: ** p < 0.01, * p < 0.05

Supplementary Figure 22. Distribution of expression of all genes on each chromosome, according to tissue (FPKM > 0.01

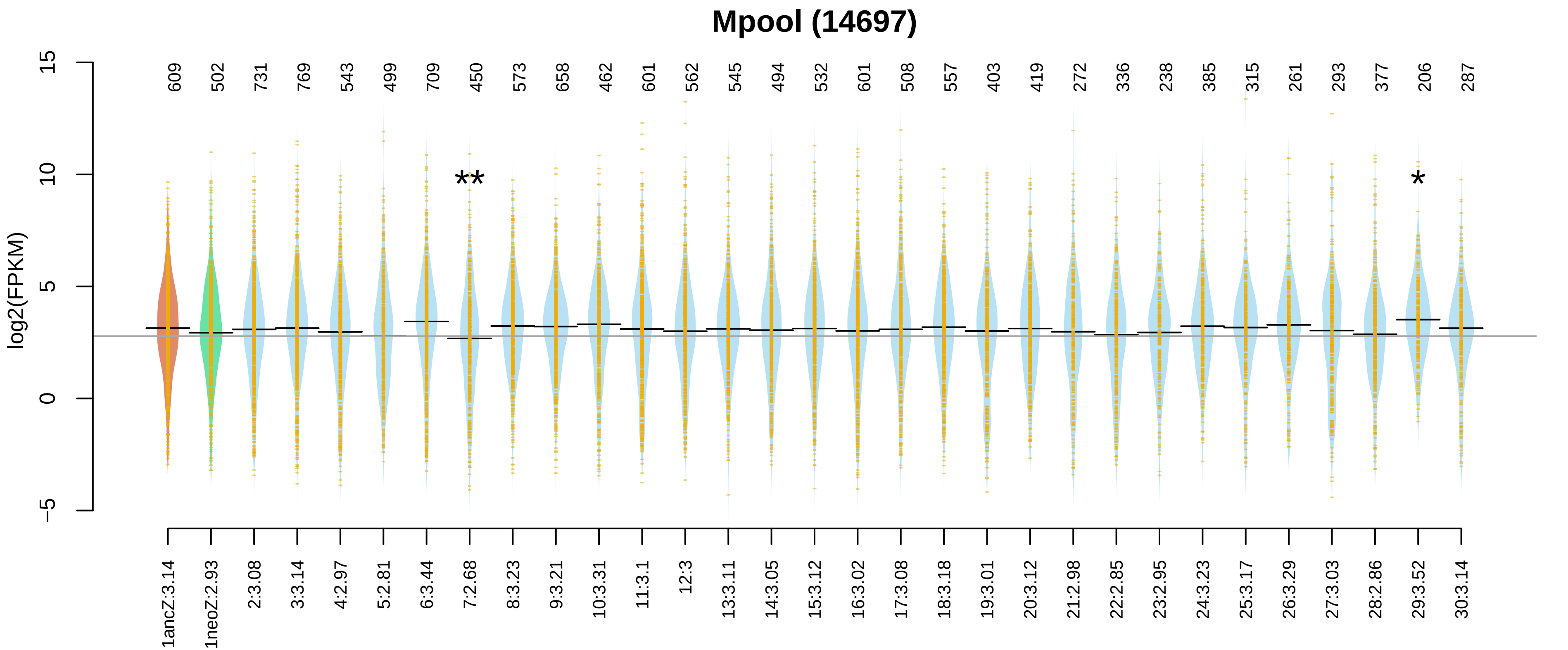

Wilcoxon test: are expression values on each chromosome different than the rest? Bonferroni−adjusted: ** $p < 0.01$, * $p < 0.05$

Supplementary Figure 22. Distribution of expression of all genes on each chromosome, according to tissue (FPKM > 0.01

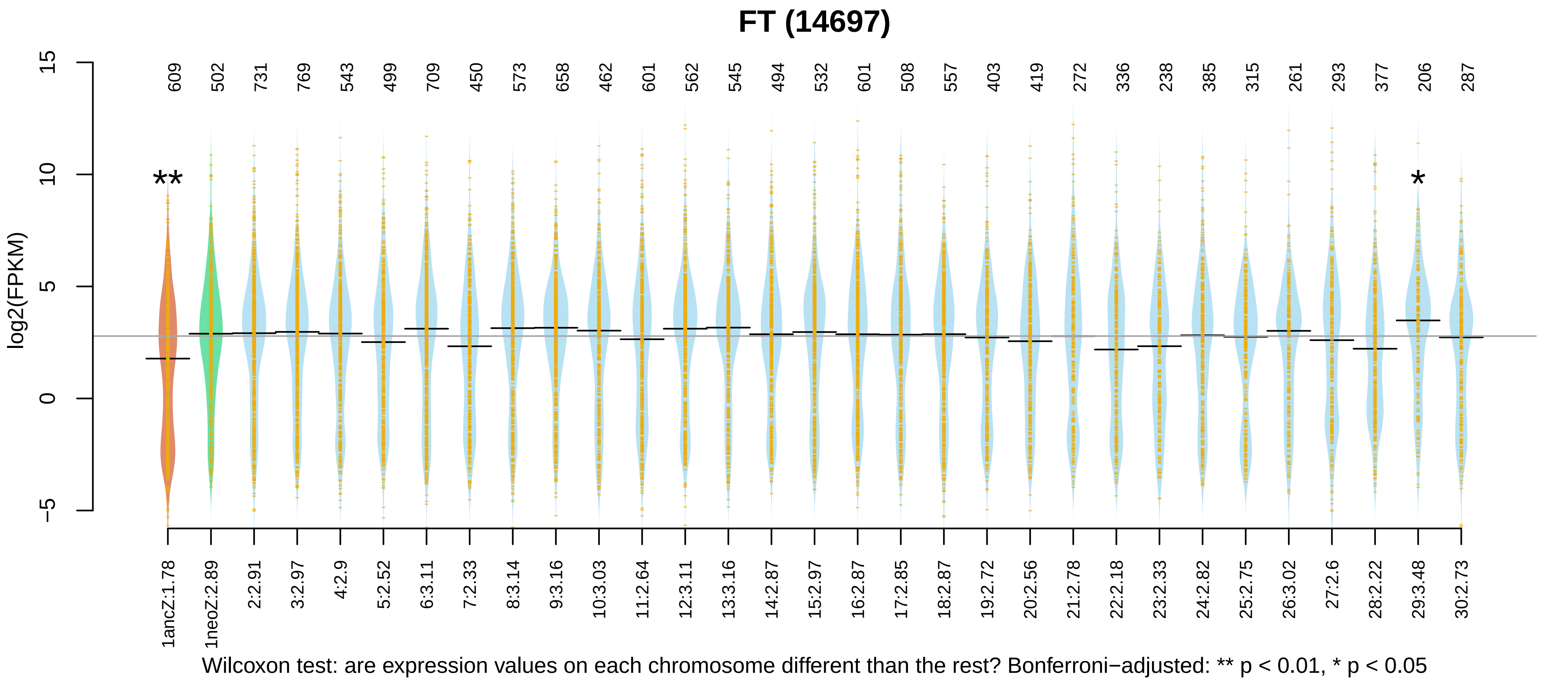

Supplementary Figure 22. Distribution of expression of all genes on each chromosome, according to tissue (FPKM > 0.01

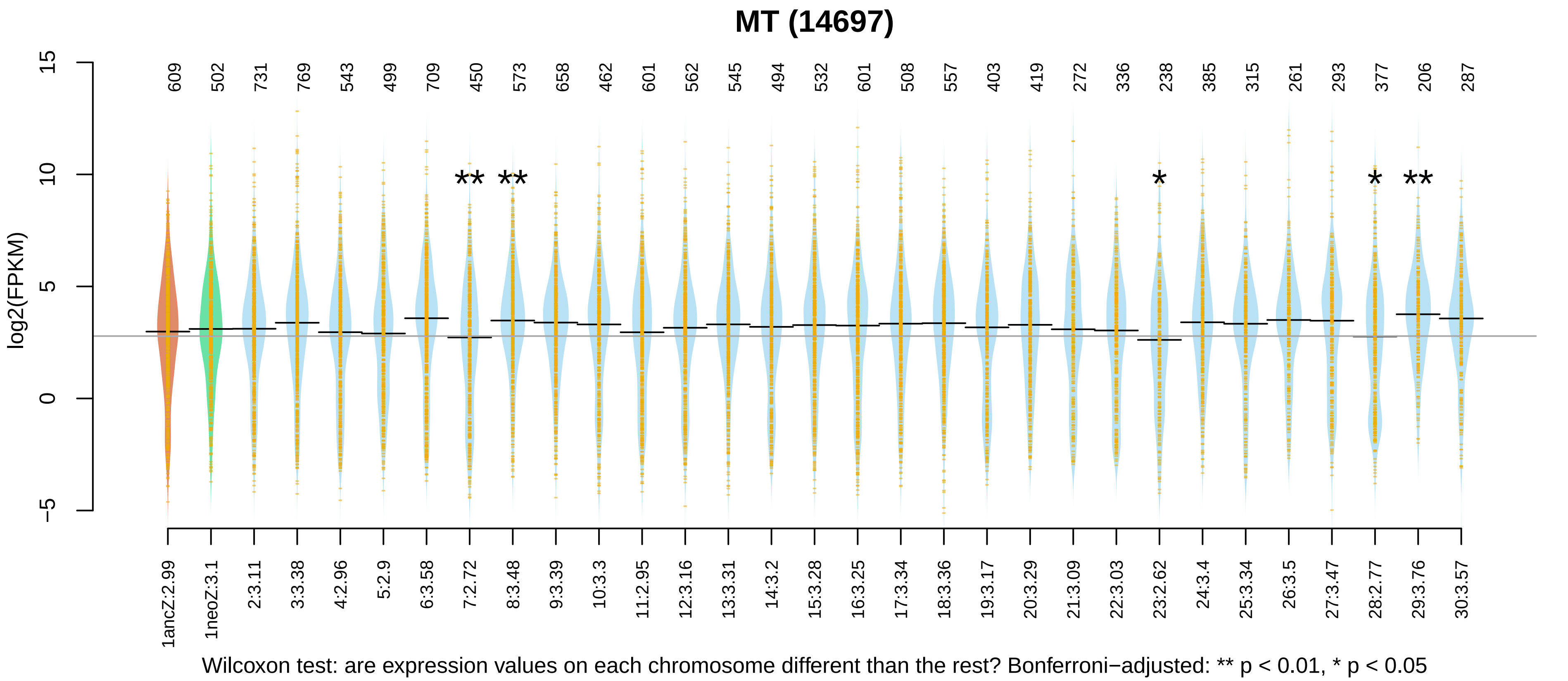

Supplementary Figure 22. Distribution of expression of all genes on each chromosome, according to tissue (FPKM > 0.01

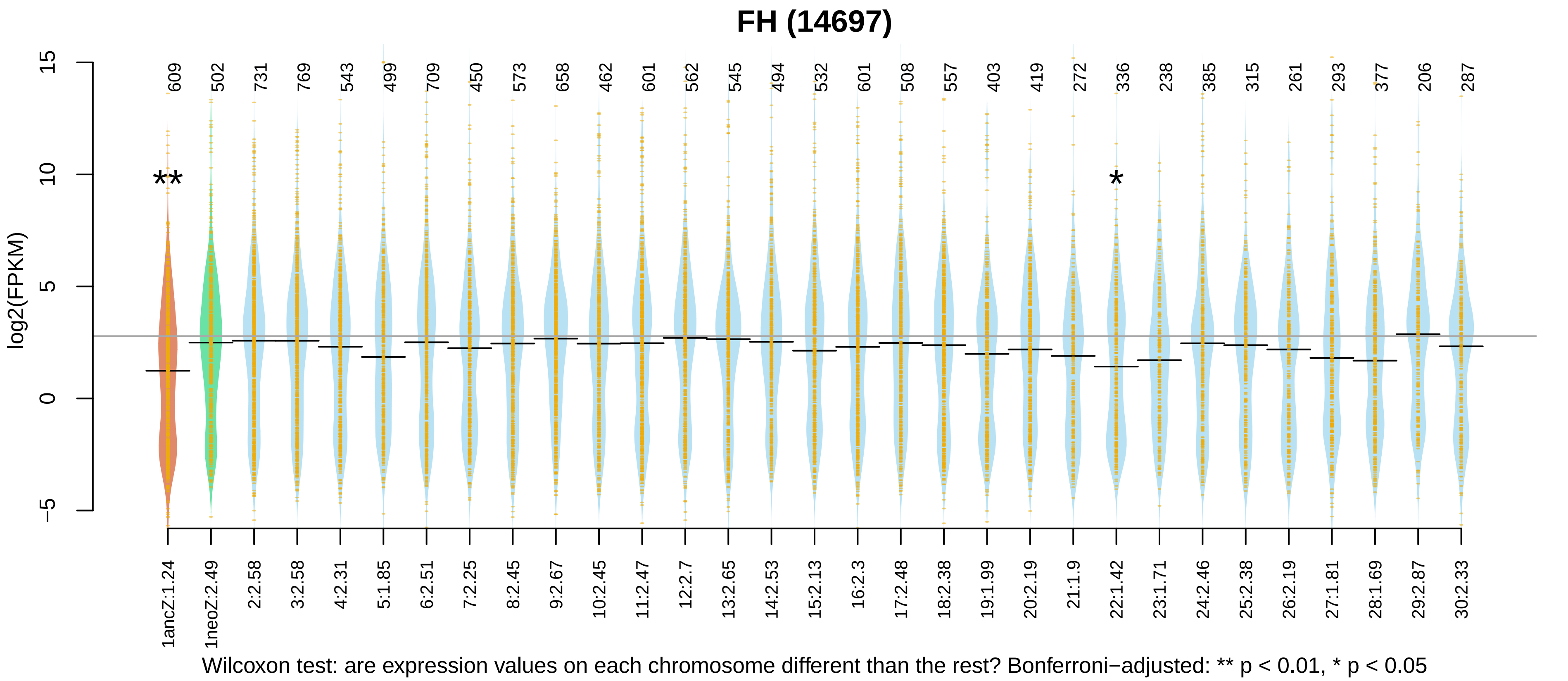

Supplementary Figure 22. Distribution of expression of all genes on each chromosome, according to tissue (FPKM > 0.01

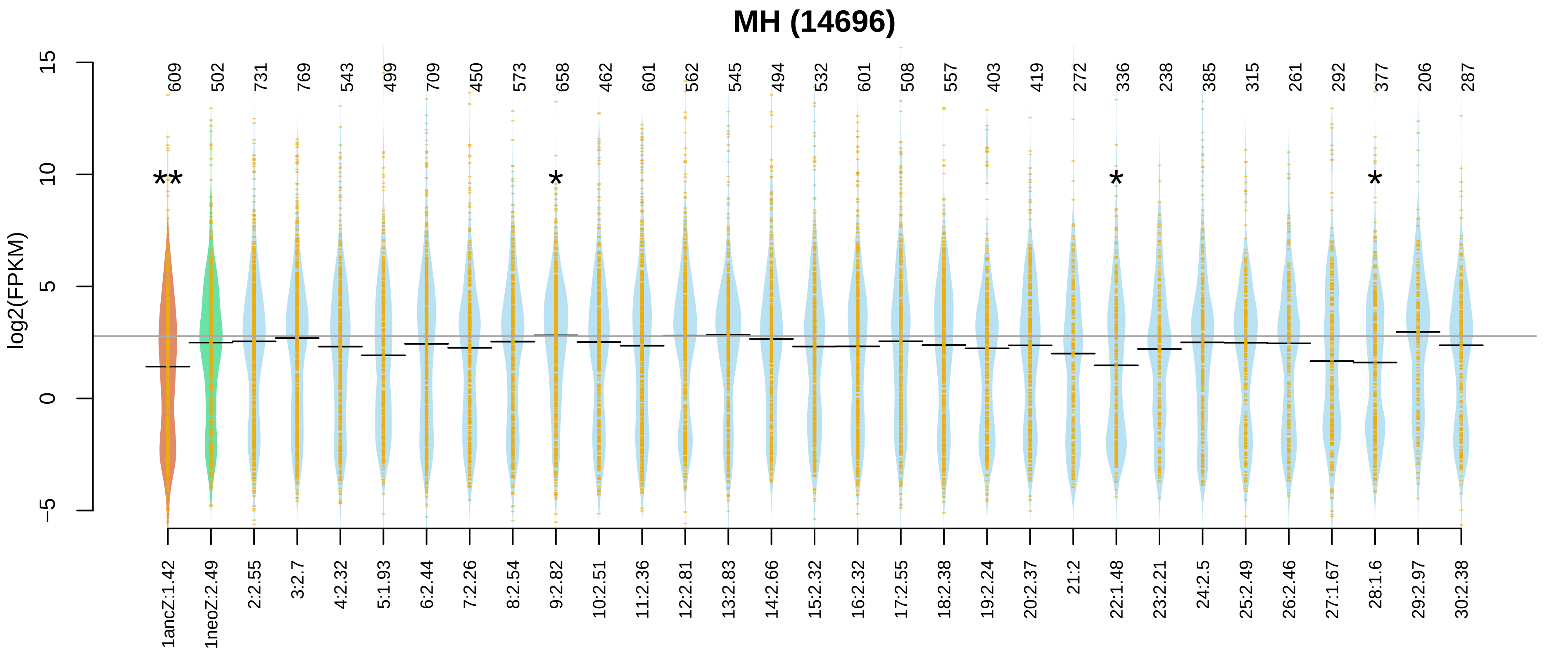

Wilcoxon test: are expression values on each chromosome different than the rest? Bonferroni−adjusted: ** p < 0.01, * p < 0.05

Supplementary Figure 22. Distribution of expression of all genes on each chromosome, according to tissue (FPKM > 0.01

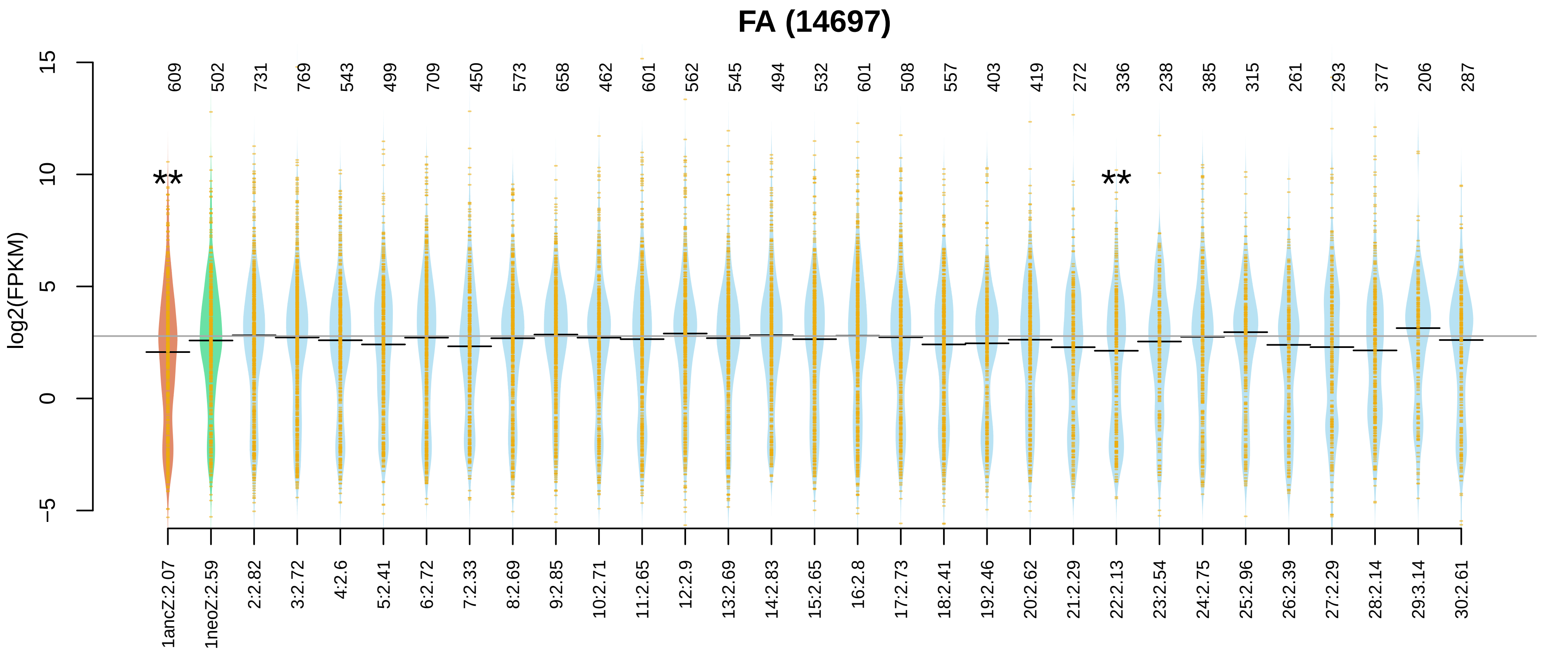

Wilcoxon test: are expression values on each chromosome different than the rest? Bonferroni−adjusted: ** p < 0.01, * p < 0.05

Supplementary Figure 22. Distribution of expression of all genes on each chromosome, according to tissue (FPKM > 0.01

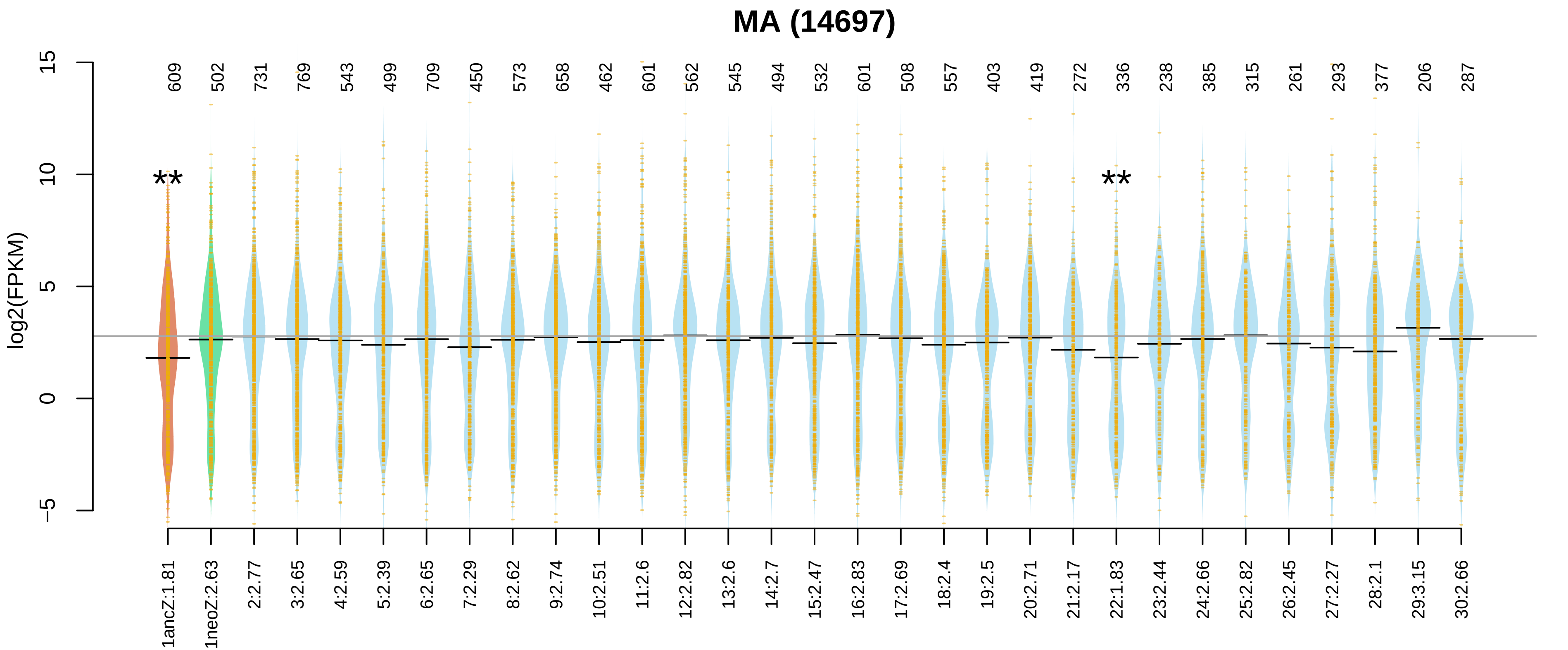

Wilcoxon test: are expression values on each chromosome different than the rest? Bonferroni−adjusted: ** p < 0.01, * p < 0.05

Supplementary Figure 22. Distribution of expression of all genes on each chromosome, according to tissue (FPKM > 0.1)

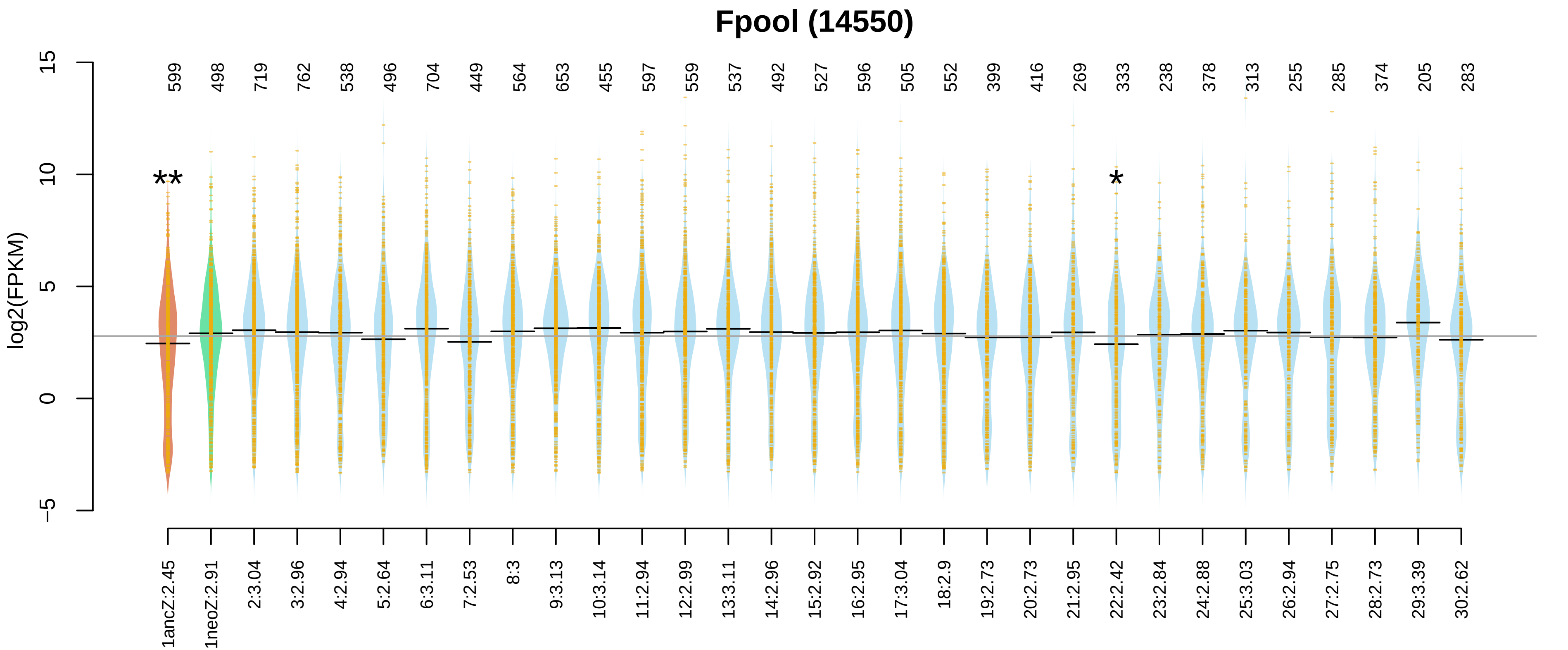

Wilcoxon test: are expression values on each chromosome different than the rest? Bonferroni−adjusted: ** p < 0.01, * p < 0.05

Supplementary Figure 22. Distribution of expression of all genes on each chromosome, according to tissue (FPKM > 0.1)

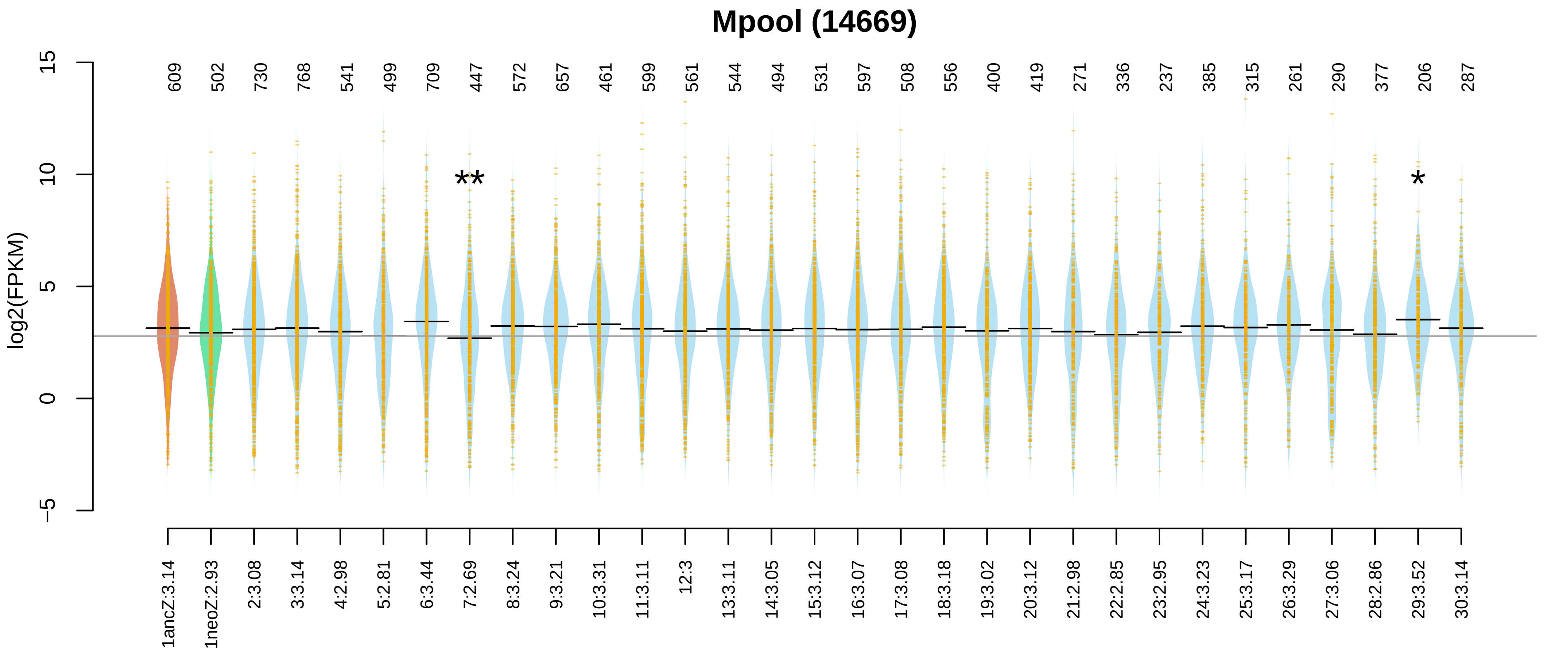

Wilcoxon test: are expression values on each chromosome different than the rest? Bonferroni−adjusted: ** $p < 0.01$, * $p < 0.05$

Supplementary Figure 22. Distribution of expression of all genes on each chromosome, according to tissue (FPKM > 0.1)

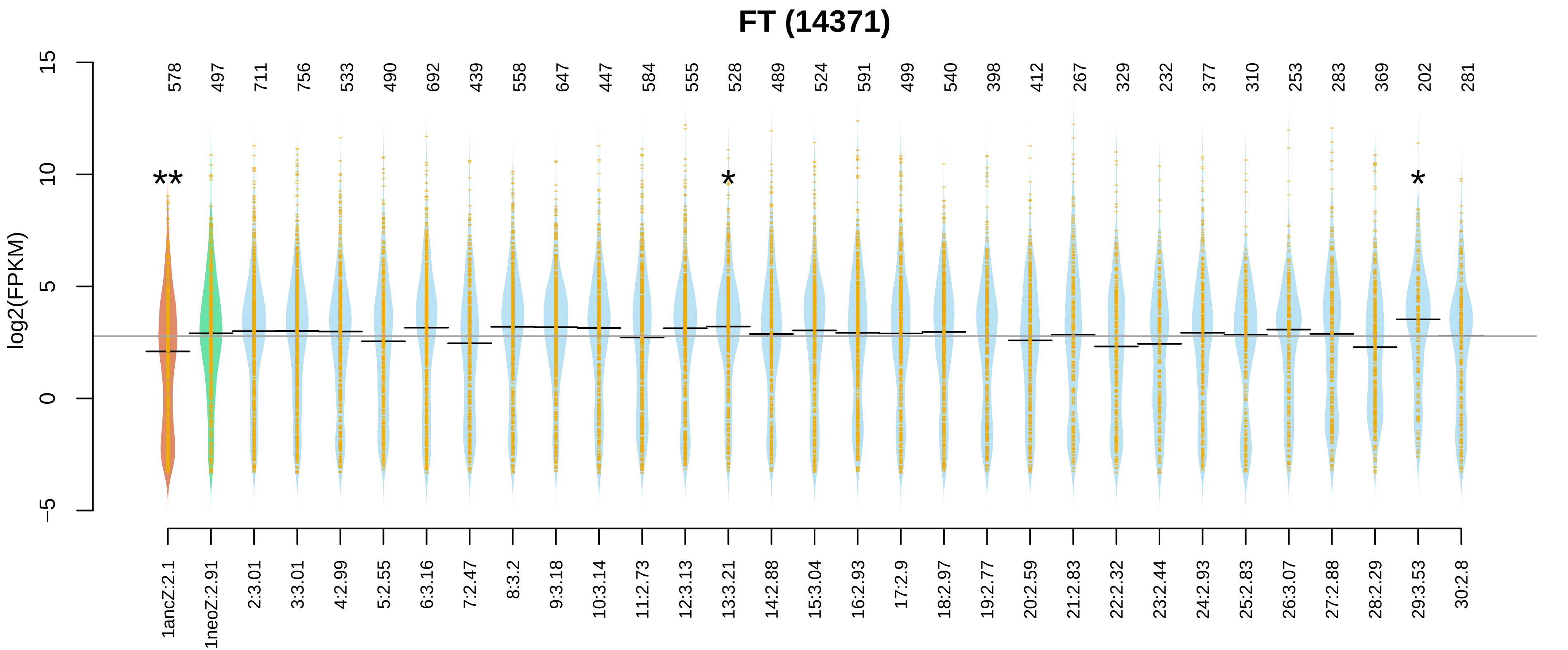

Wilcoxon test: are expression values on each chromosome different than the rest? Bonferroni−adjusted: ** p < 0.01, * p < 0.05

Supplementary Figure 22. Distribution of expression of all genes on each chromosome, according to tissue (FPKM > 0.1)

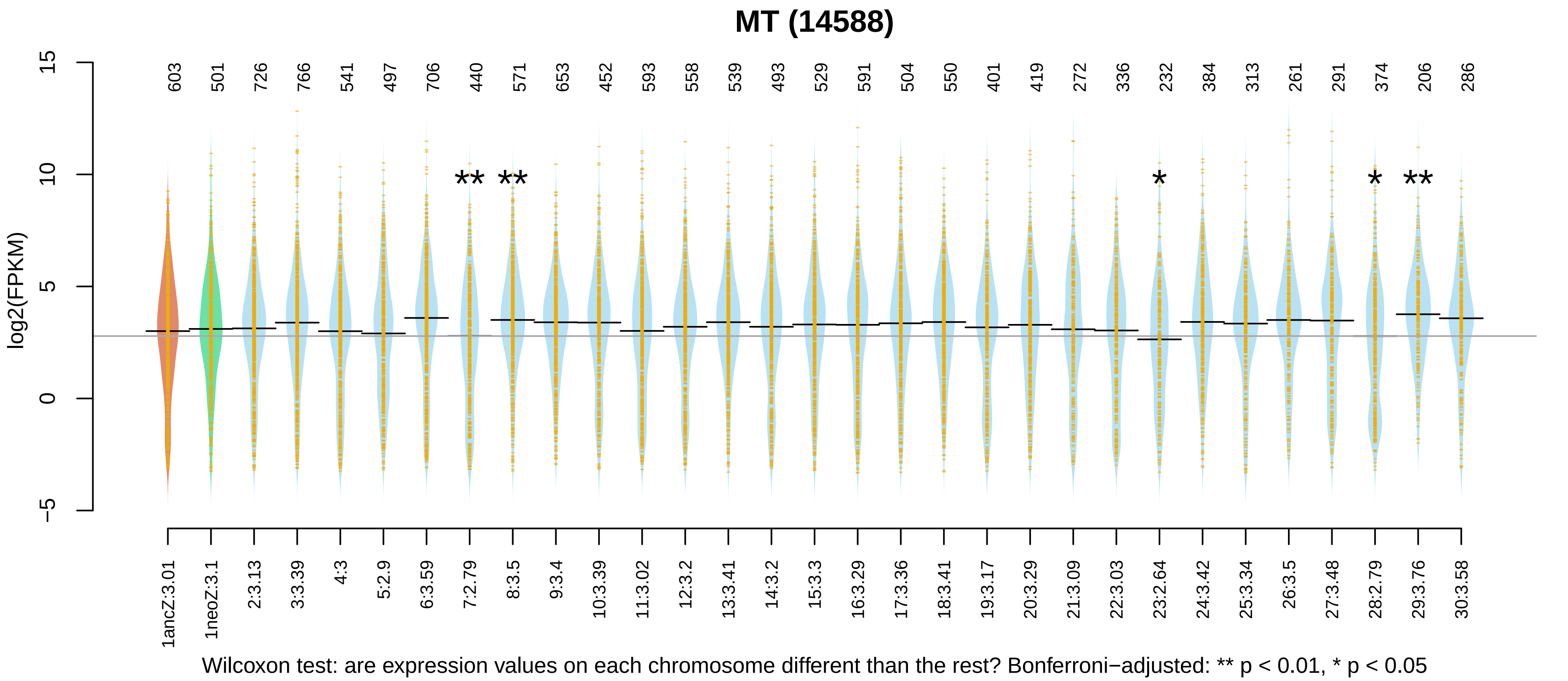

Supplementary Figure 22. Distribution of expression of all genes on each chromosome, according to tissue (FPKM > 0.1)

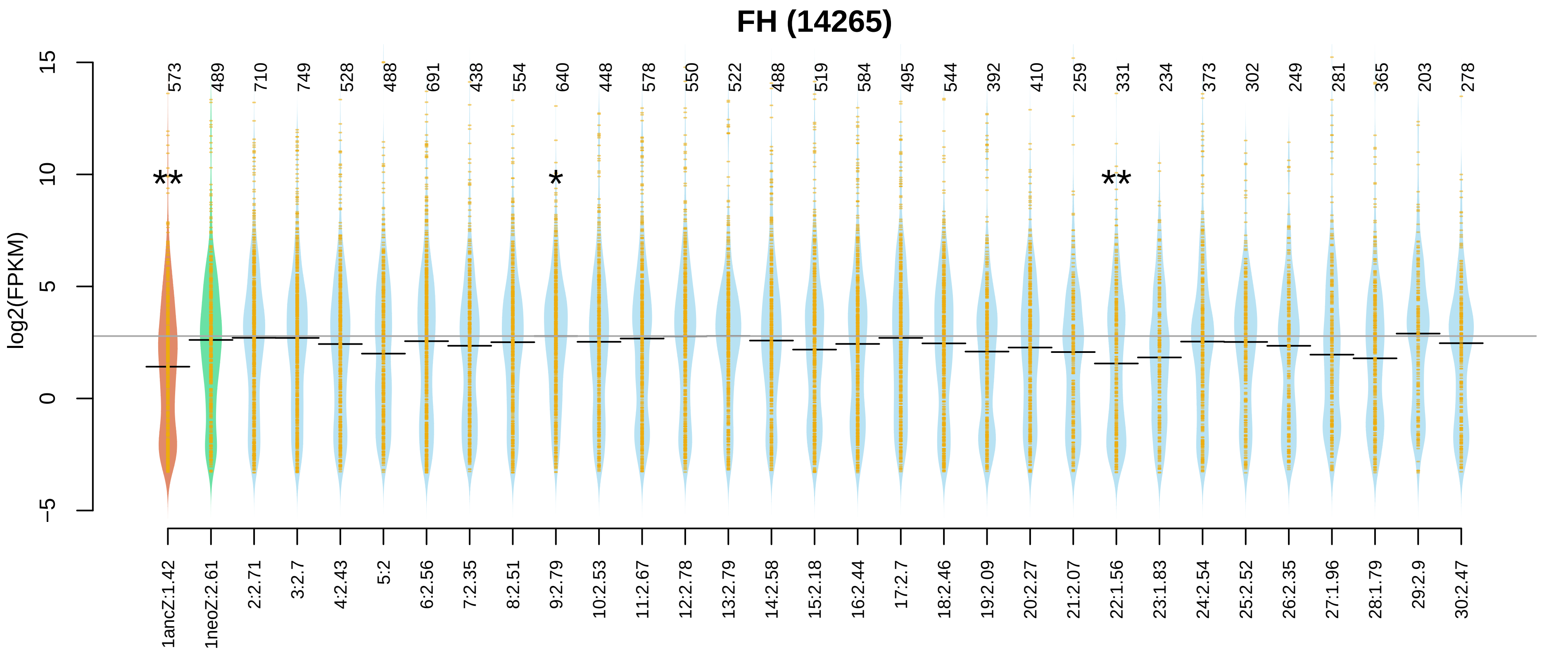

Wilcoxon test: are expression values on each chromosome different than the rest? Bonferroni−adjusted: ** p < 0.01, * p < 0.05

Supplementary Figure 22. Distribution of expression of all genes on each chromosome, according to tissue (FPKM > 0.1)

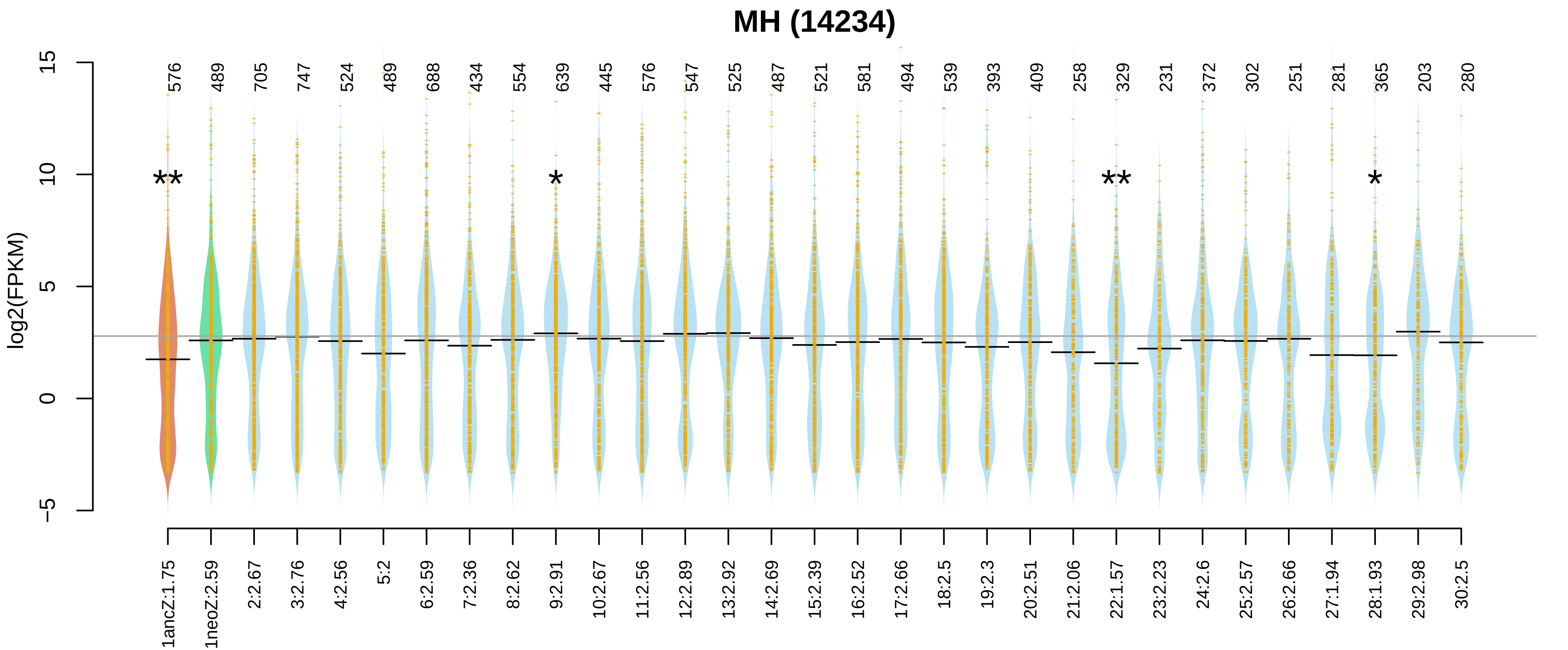

Wilcoxon test: are expression values on each chromosome different than the rest? Bonferroni−adjusted: ** $p < 0.01$, * $p < 0.05$

Supplementary Figure 22. Distribution of expression of all genes on each chromosome, according to tissue (FPKM > 0.1)

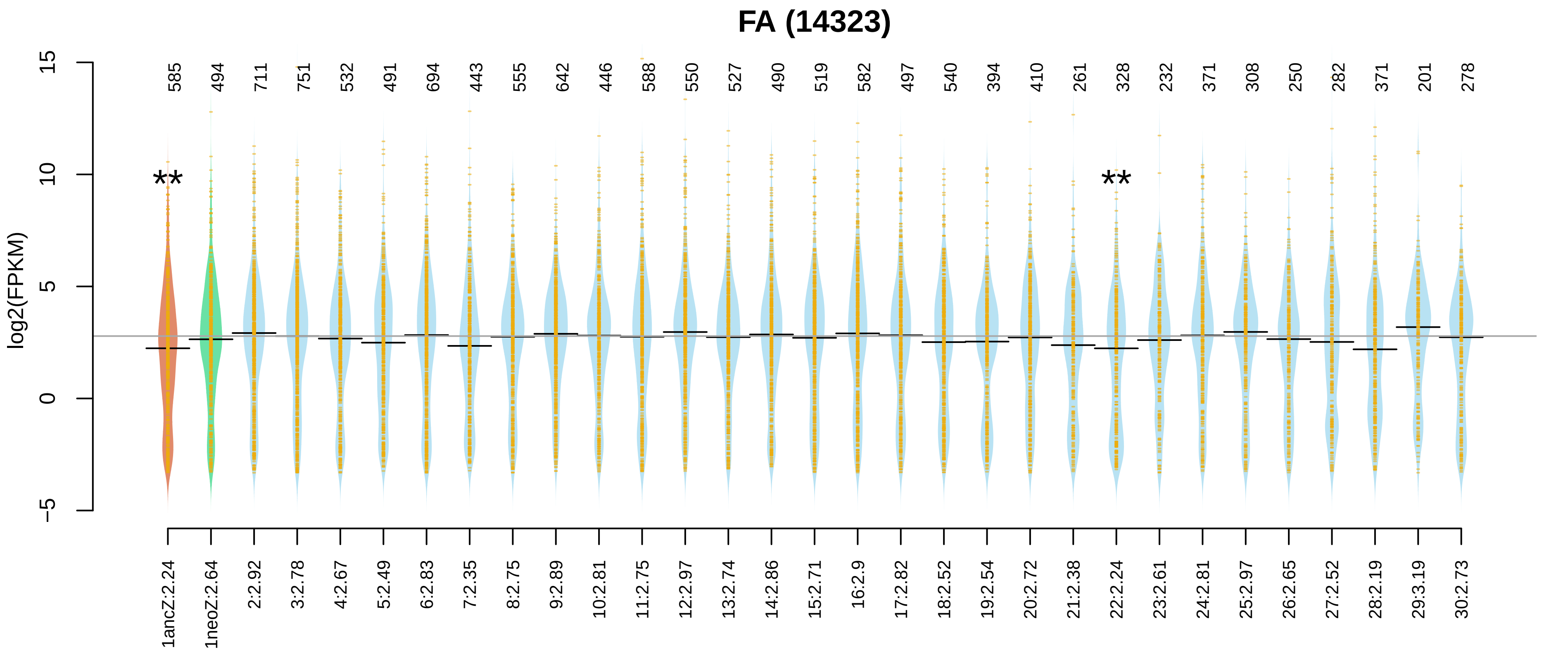

Wilcoxon test: are expression values on each chromosome different than the rest? Bonferroni−adjusted: ** p < 0.01, * p < 0.05

Supplementary Figure 22. Distribution of expression of all genes on each chromosome, according to tissue (FPKM > 0.1)

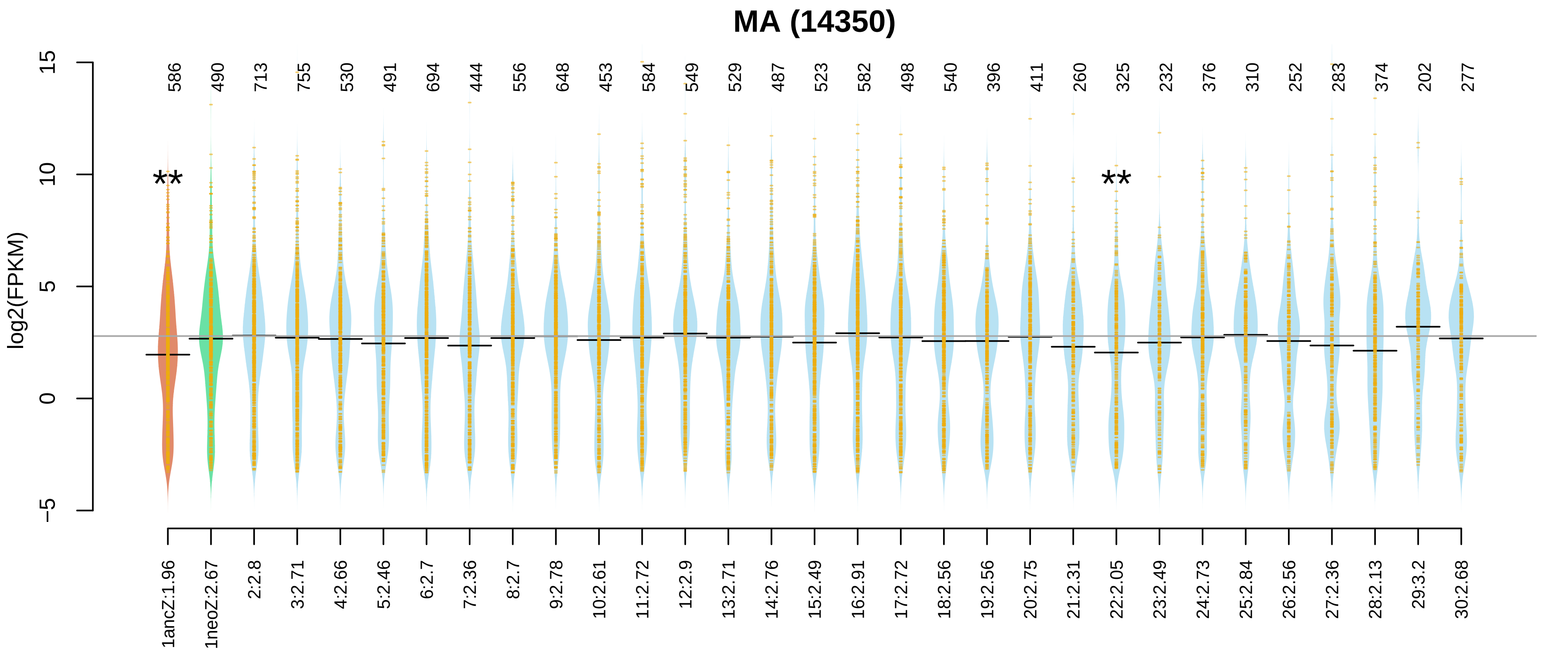

Wilcoxon test: are expression values on each chromosome different than the rest? Bonferroni−adjusted: ** p < 0.01, * p < 0.05

Supplementary Figure 22. Distribution of expression of all genes on each chromosome, according to tissue (FPKM > 0.5)

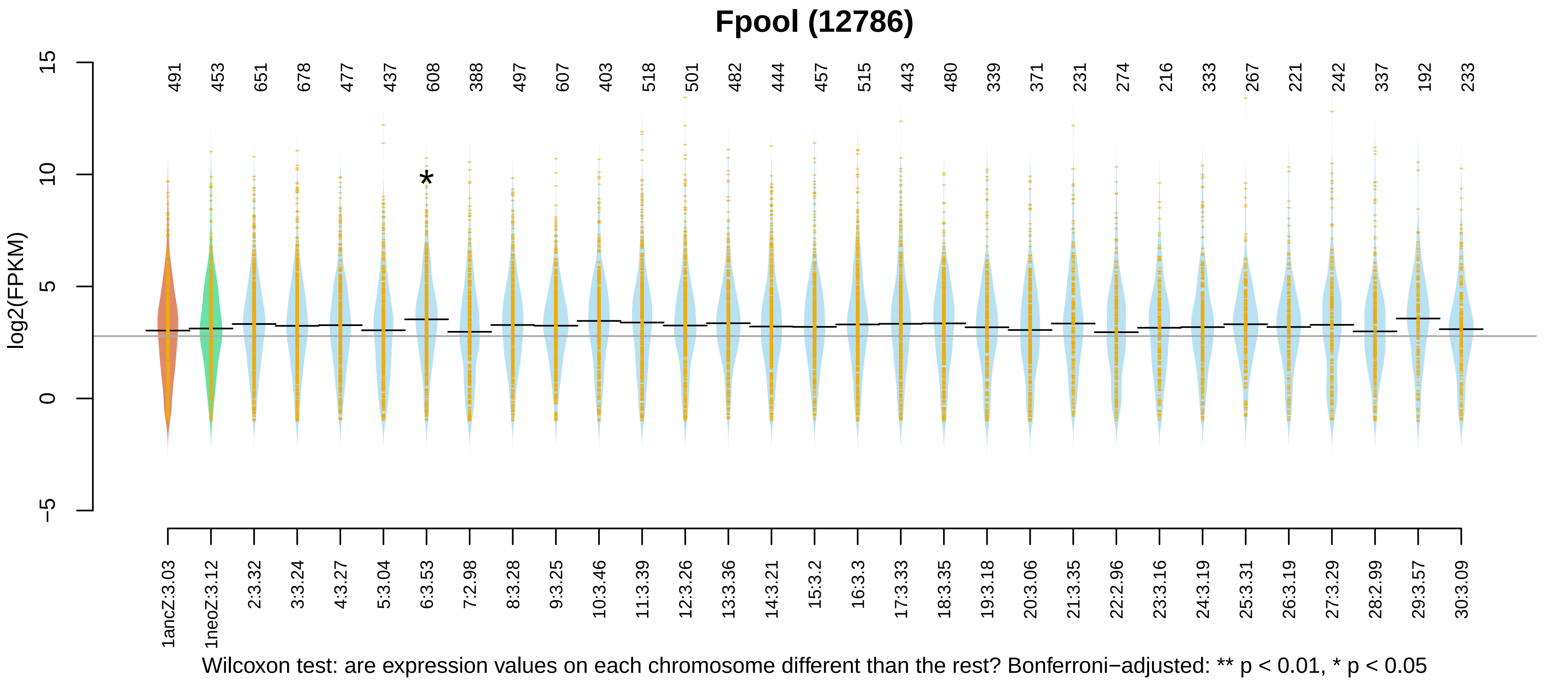

Supplementary Figure 22. Distribution of expression of all genes on each chromosome, according to tissue (FPKM > 0.5)

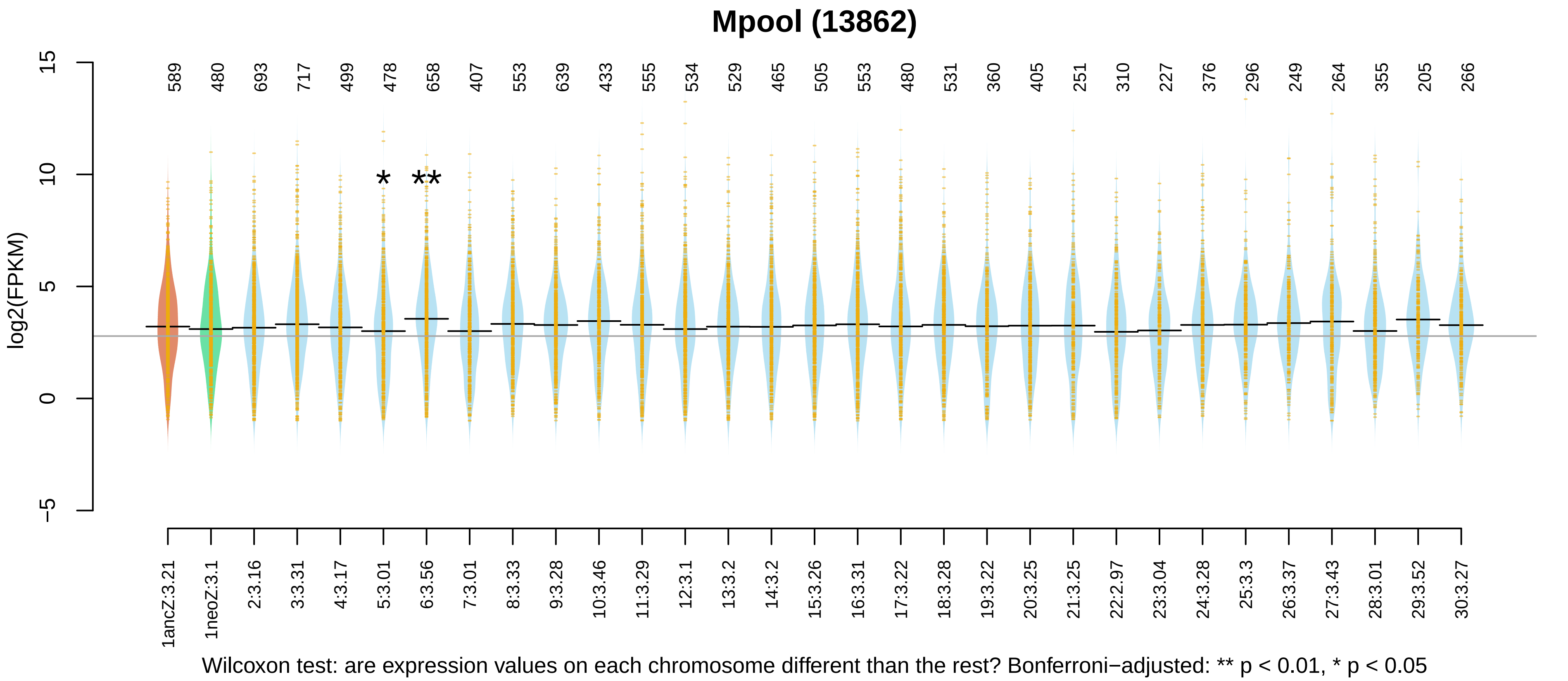

Supplementary Figure 22. Distribution of expression of all genes on each chromosome, according to tissue (FPKM > 0.5)

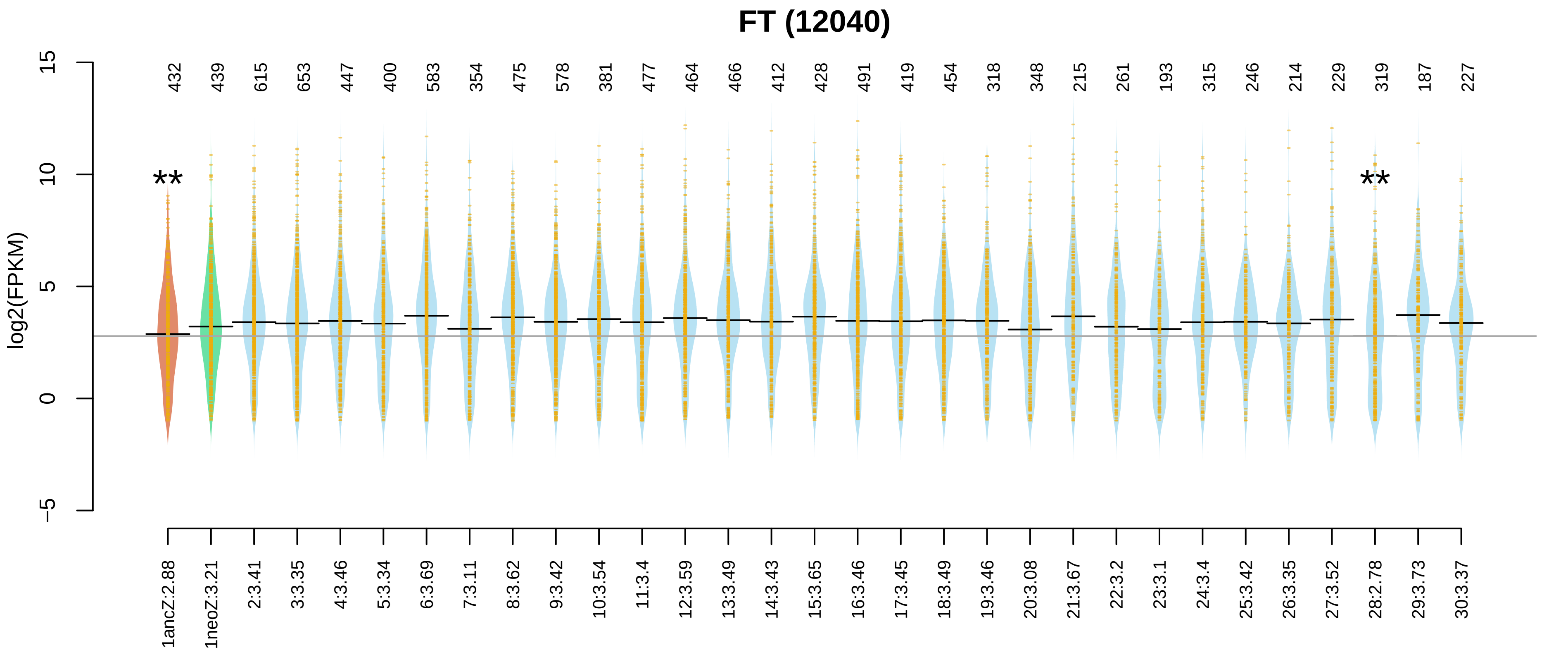

Wilcoxon test: are expression values on each chromosome different than the rest? Bonferroni−adjusted: ** p < 0.01, * p < 0.05

Supplementary Figure 22. Distribution of expression of all genes on each chromosome, according to tissue (FPKM > 0.5)

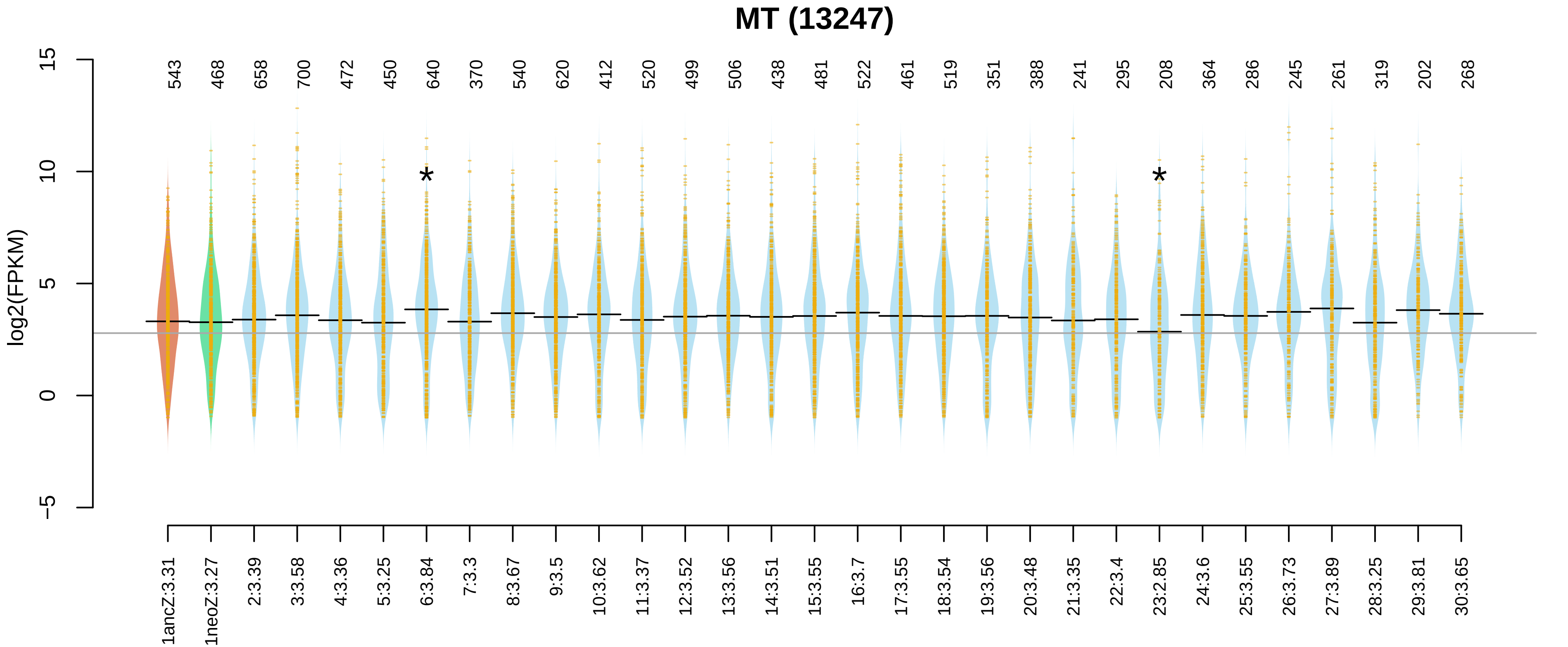

Wilcoxon test: are expression values on each chromosome different than the rest? Bonferroni−adjusted: ** p < 0.01, * p < 0.05

Supplementary Figure 22. Distribution of expression of all genes on each chromosome, according to tissue (FPKM > 0.5)

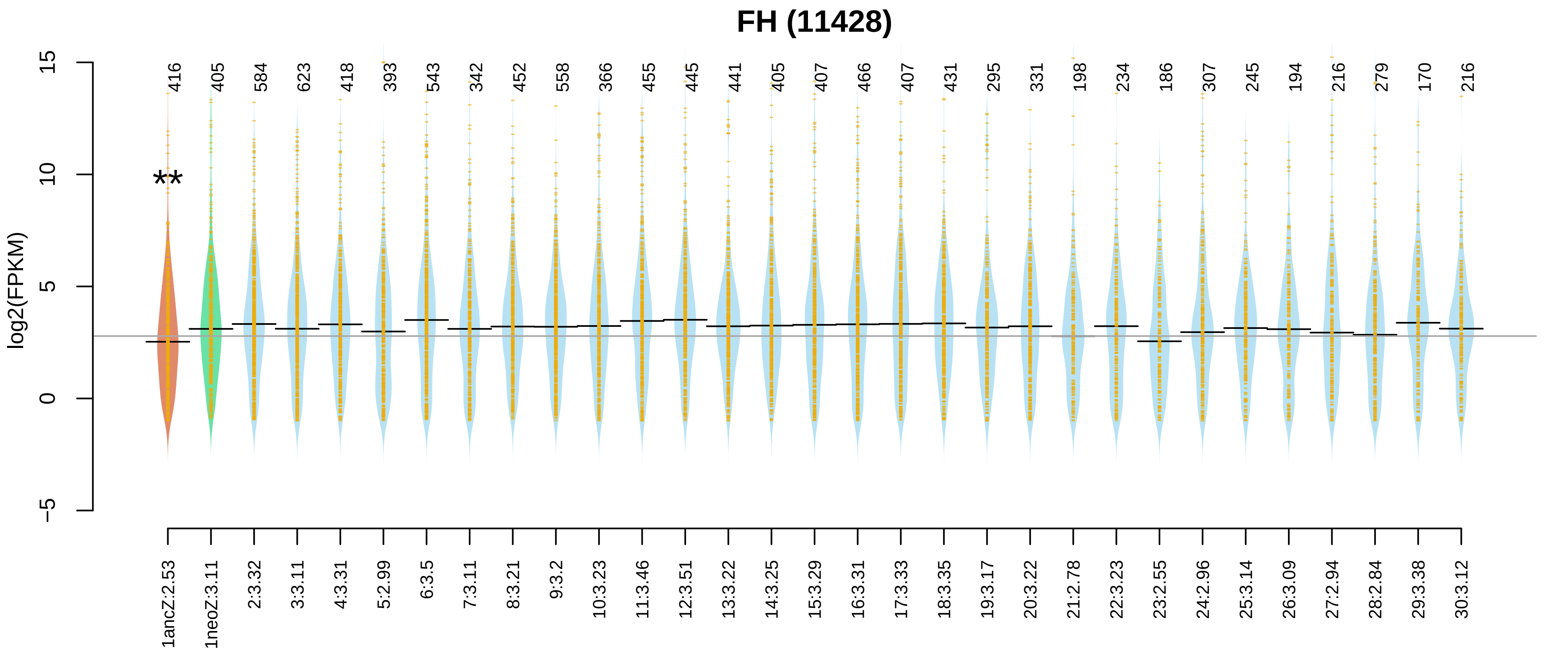

Wilcoxon test: are expression values on each chromosome different than the rest? Bonferroni−adjusted: ** $p < 0.01$, * $p < 0.05$

Supplementary Figure 22. Distribution of expression of all genes on each chromosome, according to tissue (FPKM > 0.5)

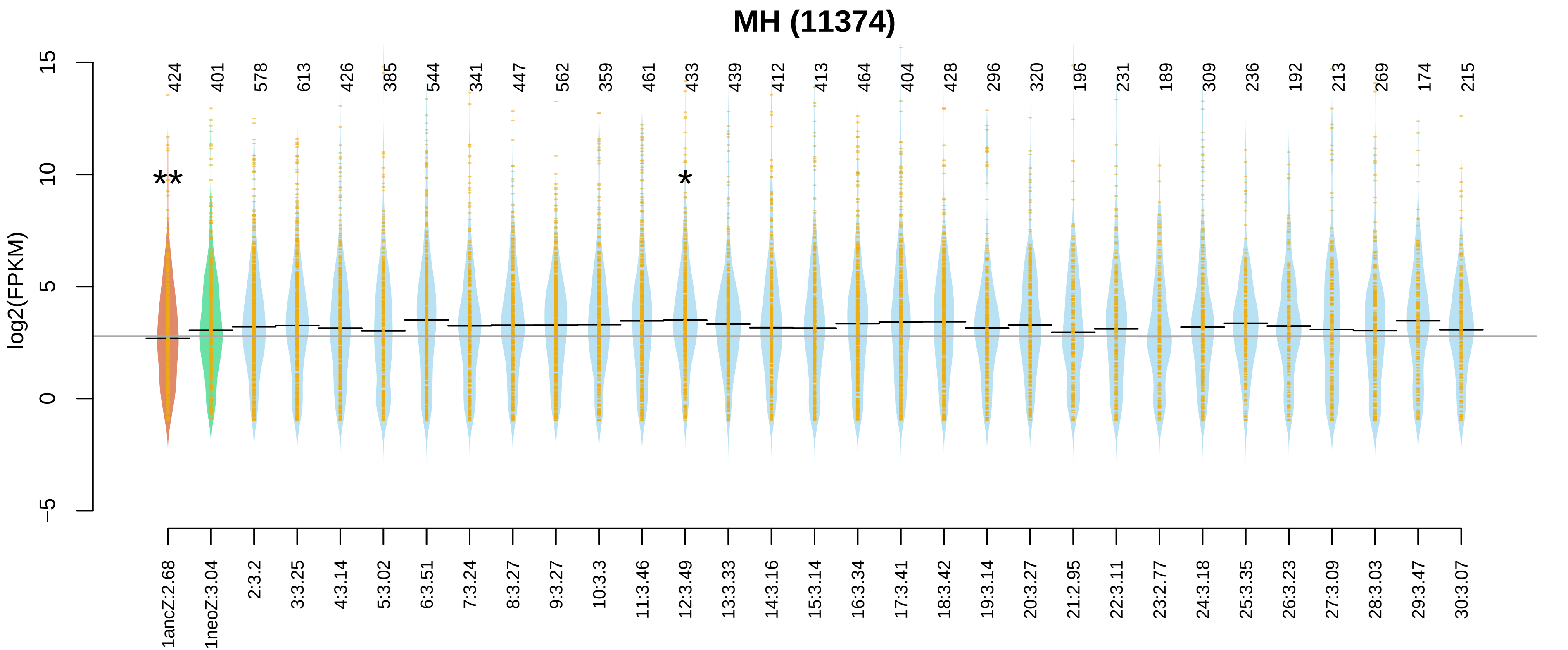

Wilcoxon test: are expression values on each chromosome different than the rest? Bonferroni−adjusted: ** p < 0.01, * p < 0.05

Supplementary Figure 22. Distribution of expression of all genes on each chromosome, according to tissue (FPKM > 0.5)

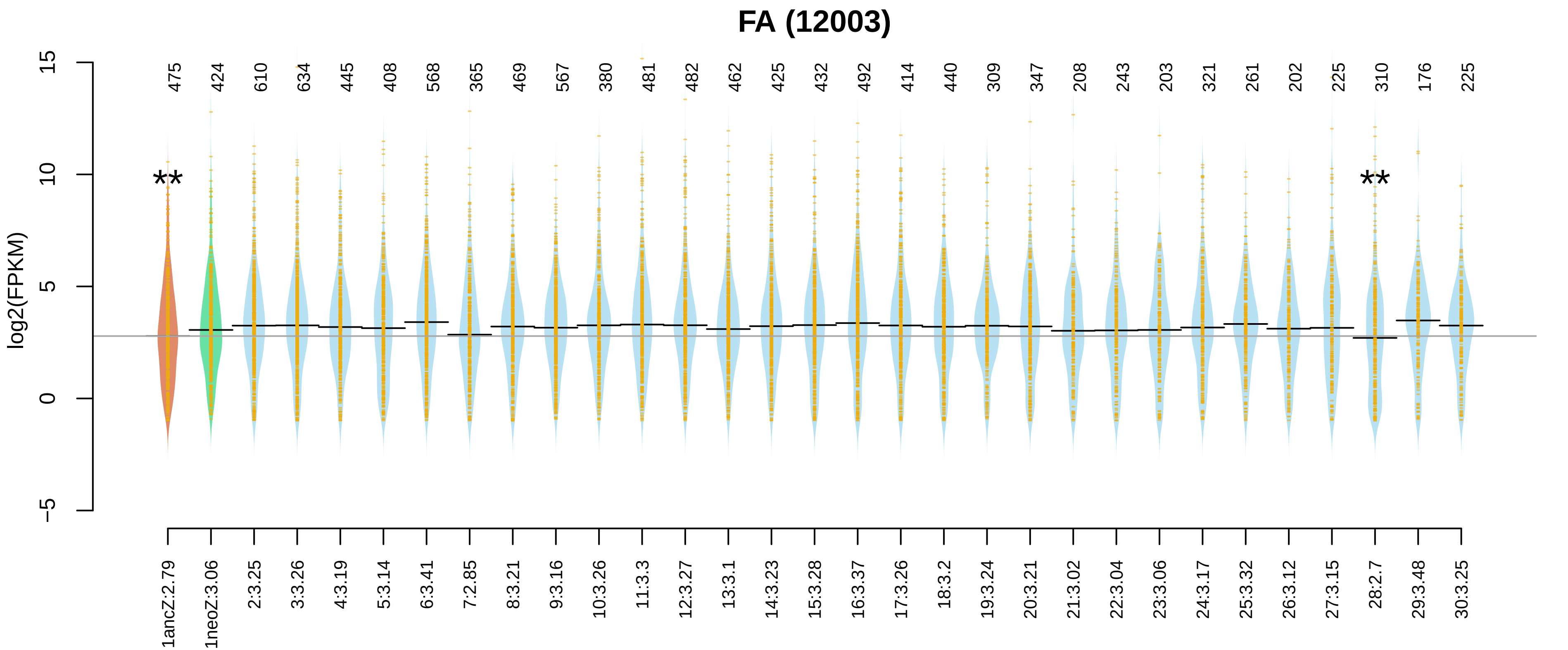

Wilcoxon test: are expression values on each chromosome different than the rest? Bonferroni−adjusted: ** p < 0.01, * p < 0.05

Supplementary Figure 22. Distribution of expression of all genes on each chromosome, according to tissue (FPKM > 0.5)

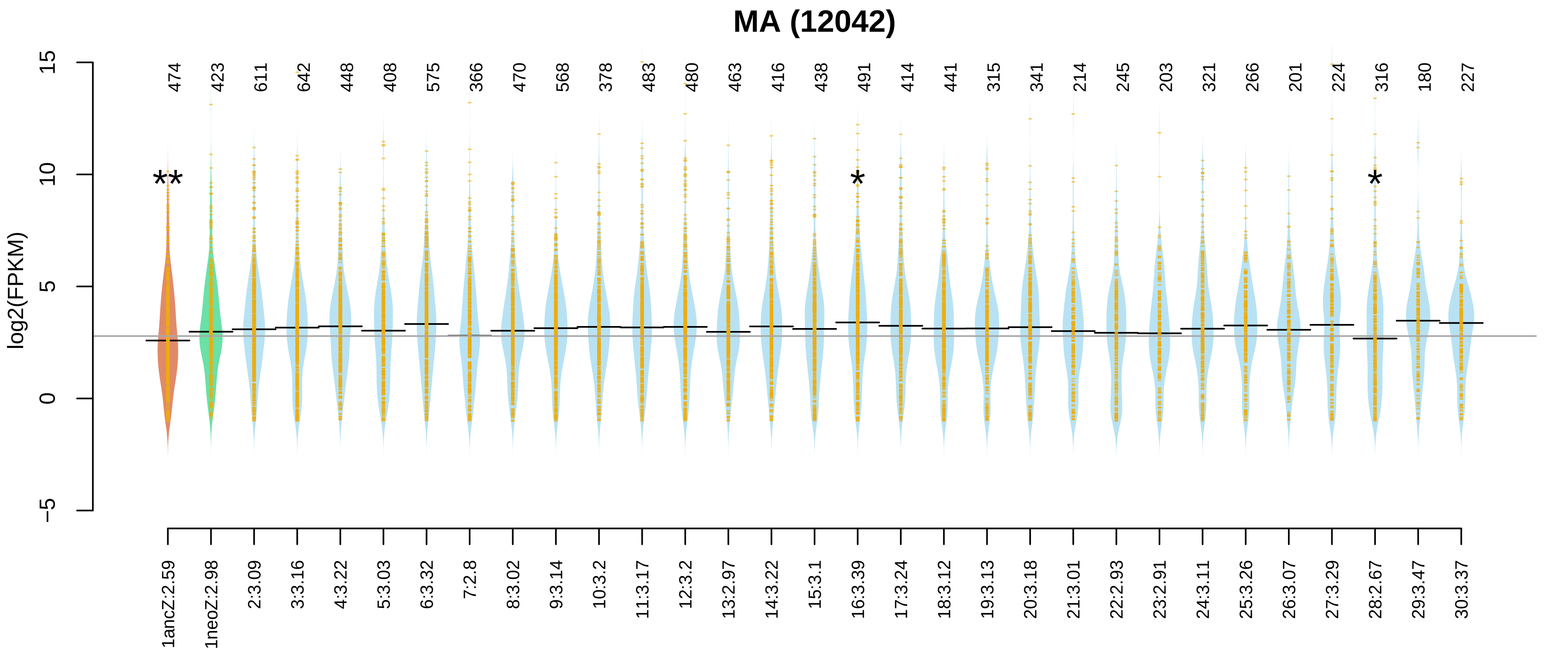

Wilcoxon test: are expression values on each chromosome different than the rest? Bonferroni−adjusted: ** p < 0.01, * p < 0.05

Supplementary Figure 22. Distribution of expression of all genes on each chromosome, according to tissue (FPKM > 1)

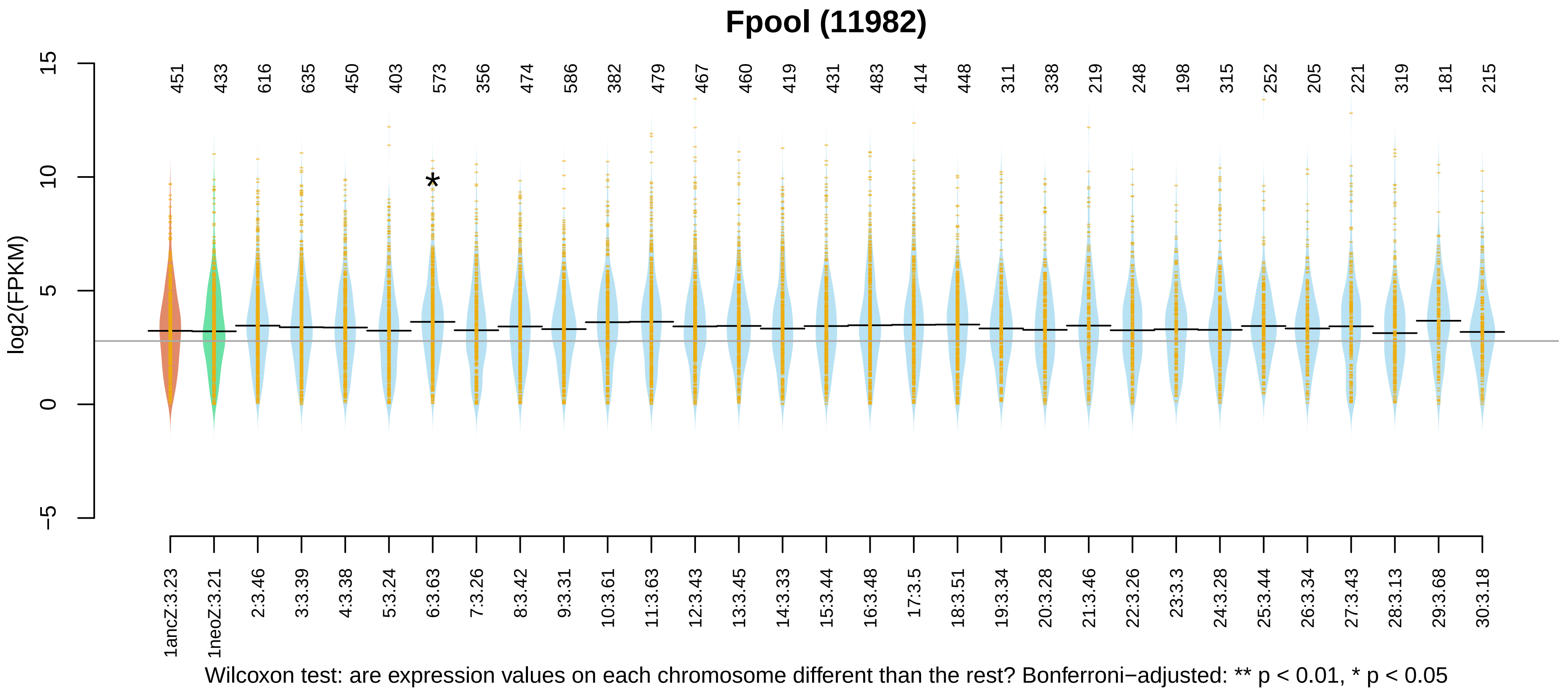

Supplementary Figure 22. Distribution of expression of all genes on each chromosome, according to tissue (FPKM > 1)

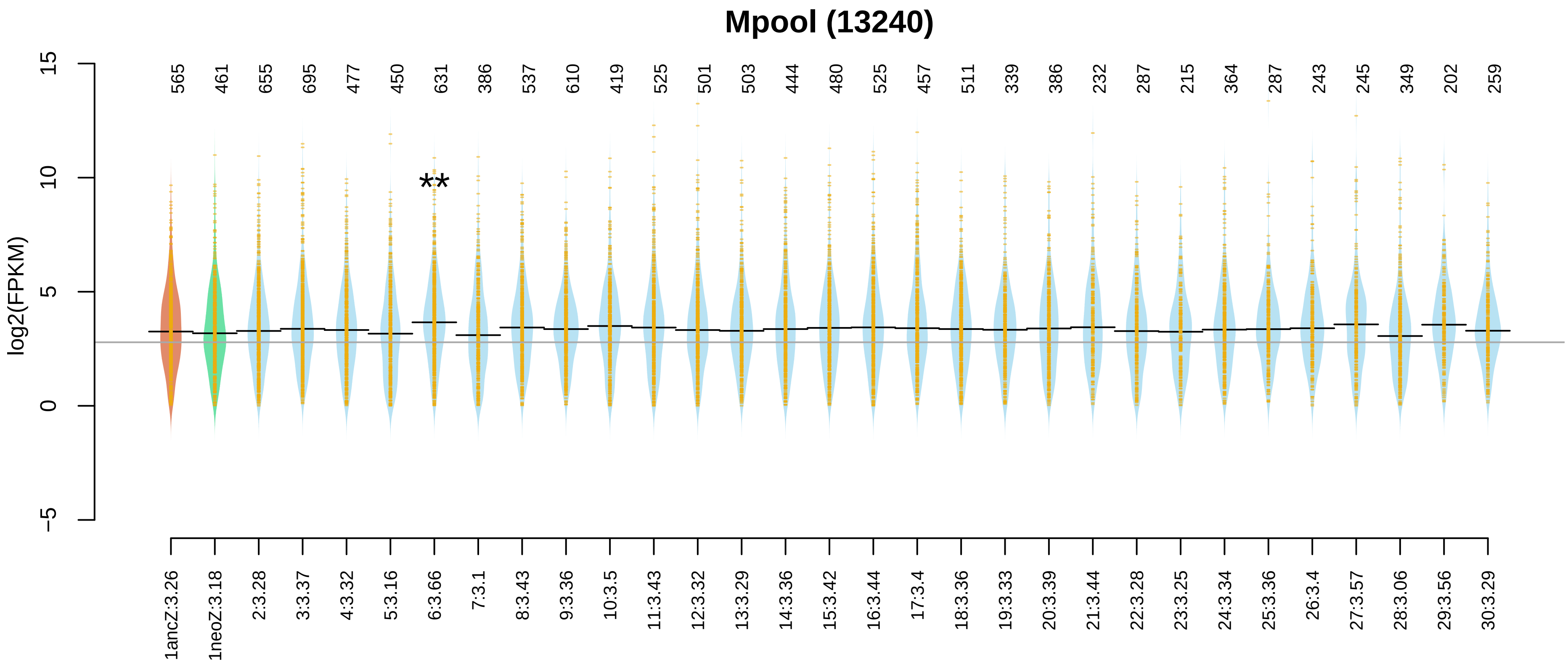

Wilcoxon test: are expression values on each chromosome different than the rest? Bonferroni−adjusted: ** $p < 0.01$, * $p < 0.05$

Supplementary Figure 22. Distribution of expression of all genes on each chromosome, according to tissue (FPKM > 1)

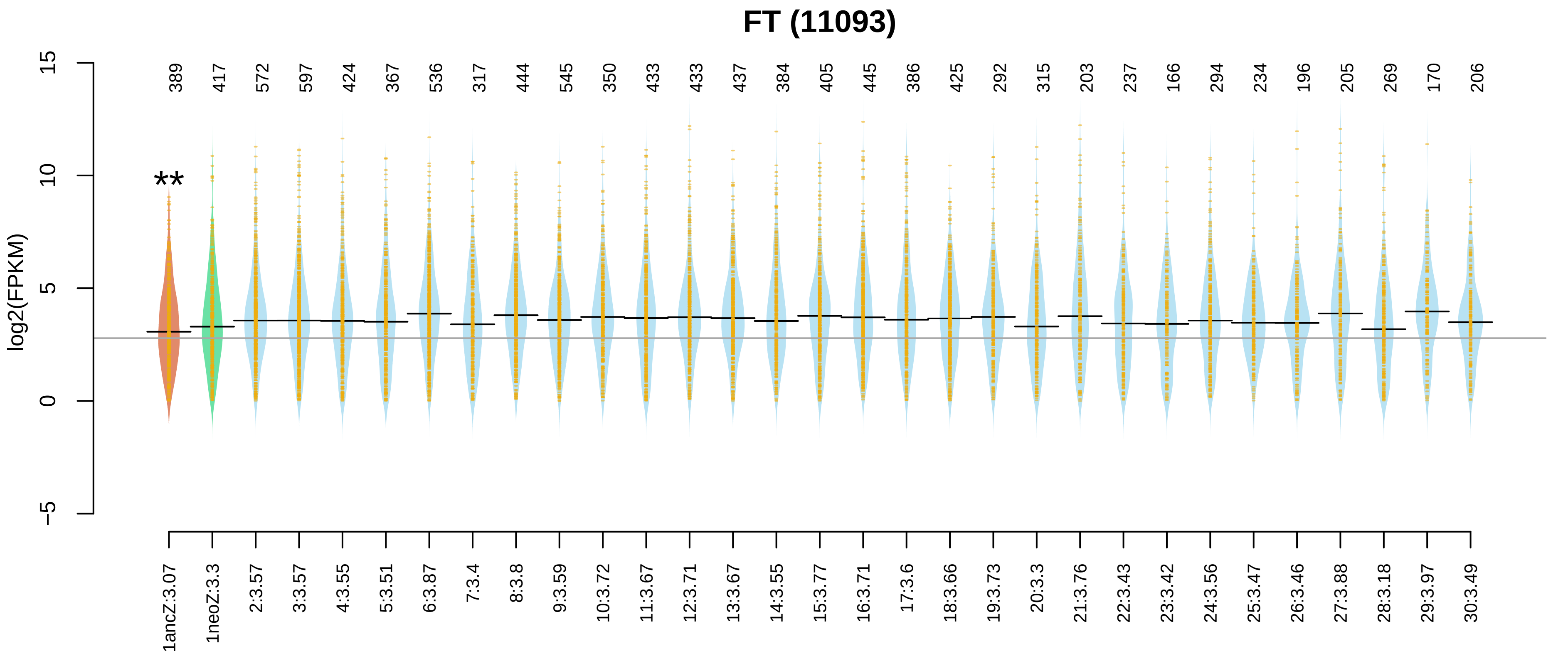

Wilcoxon test: are expression values on each chromosome different than the rest? Bonferroni−adjusted: ** p < 0.01, * p < 0.05

Supplementary Figure 22. Distribution of expression of all genes on each chromosome, according to tissue (FPKM > 1)

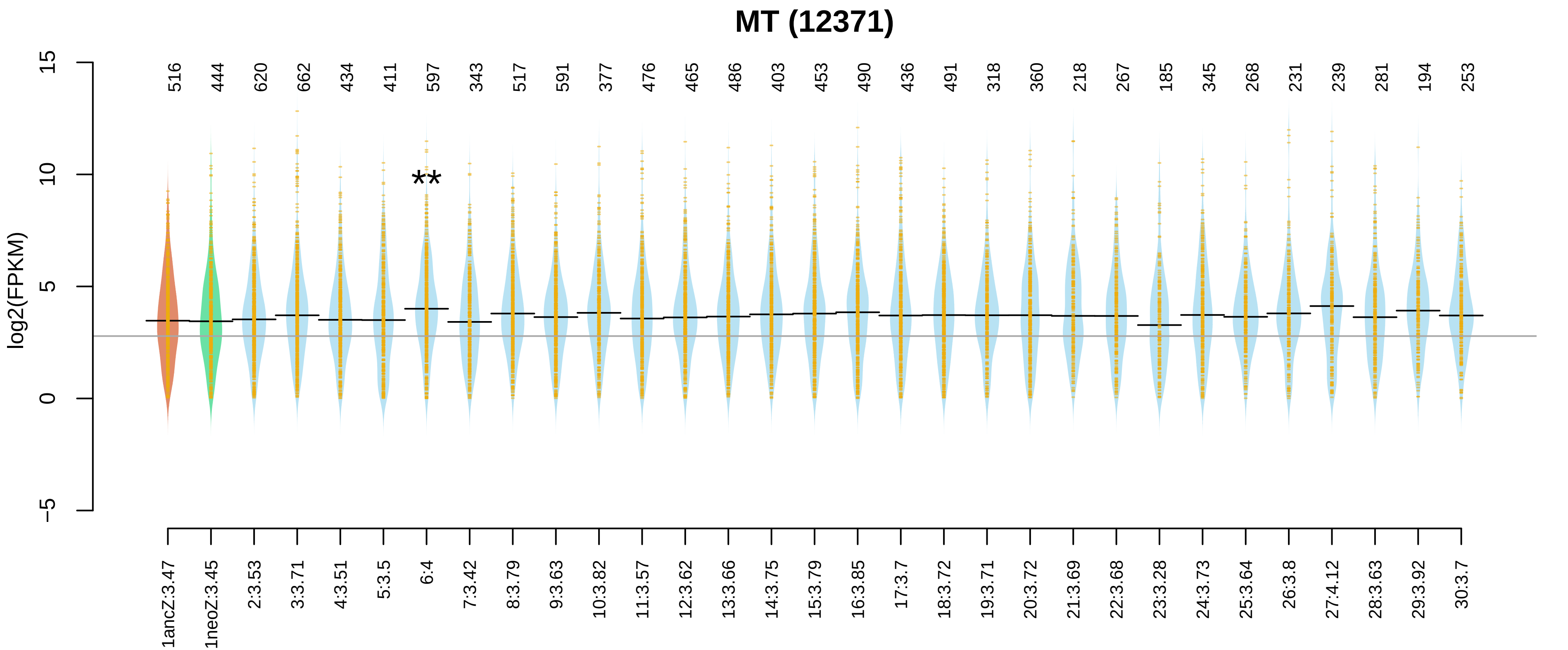

Wilcoxon test: are expression values on each chromosome different than the rest? Bonferroni−adjusted: ** p < 0.01, * p < 0.05

Supplementary Figure 22. Distribution of expression of all genes on each chromosome, according to tissue (FPKM > 1)

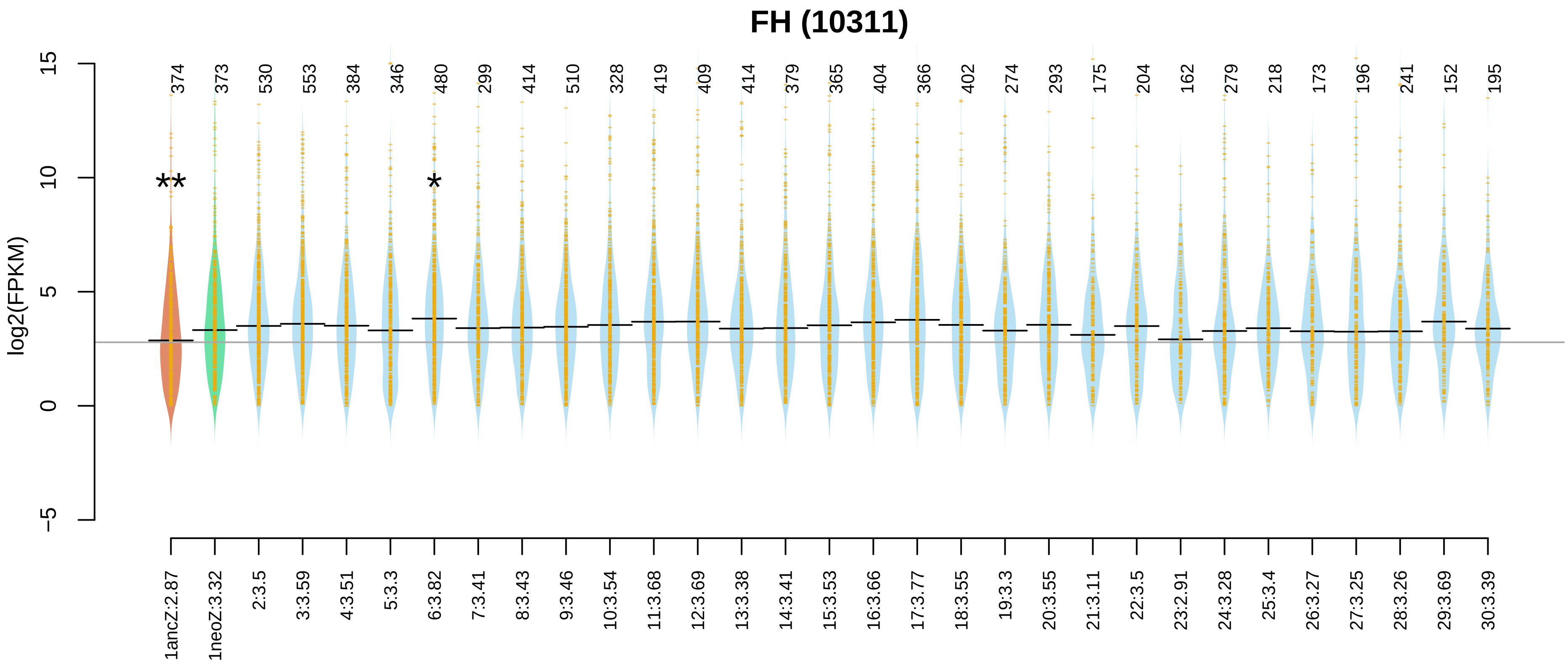

Wilcoxon test: are expression values on each chromosome different than the rest? Bonferroni−adjusted: ** p < 0.01, * p < 0.05

Supplementary Figure 22. Distribution of expression of all genes on each chromosome, according to tissue (FPKM > 1)

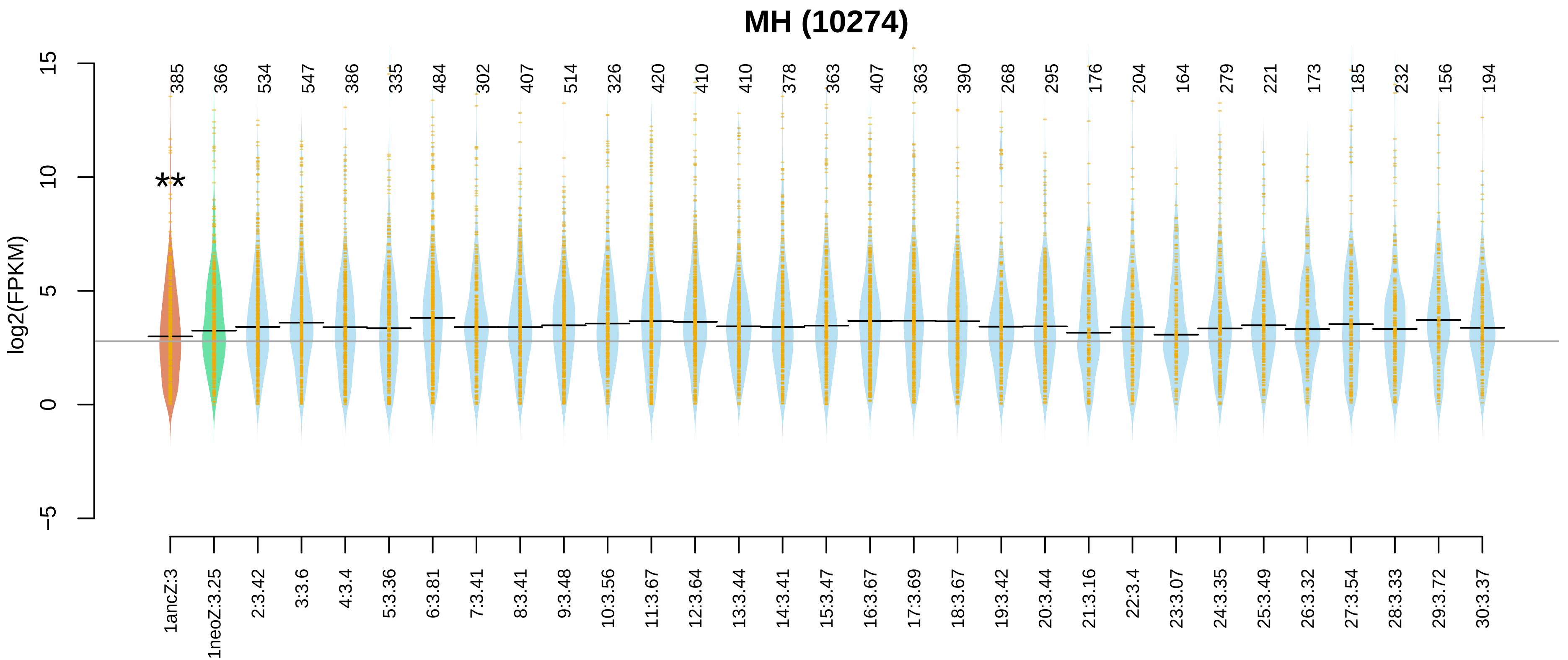

Wilcoxon test: are expression values on each chromosome different than the rest? Bonferroni−adjusted: ** p < 0.01, * p < 0.05

Supplementary Figure 22. Distribution of expression of all genes on each chromosome, according to tissue (FPKM > 1)

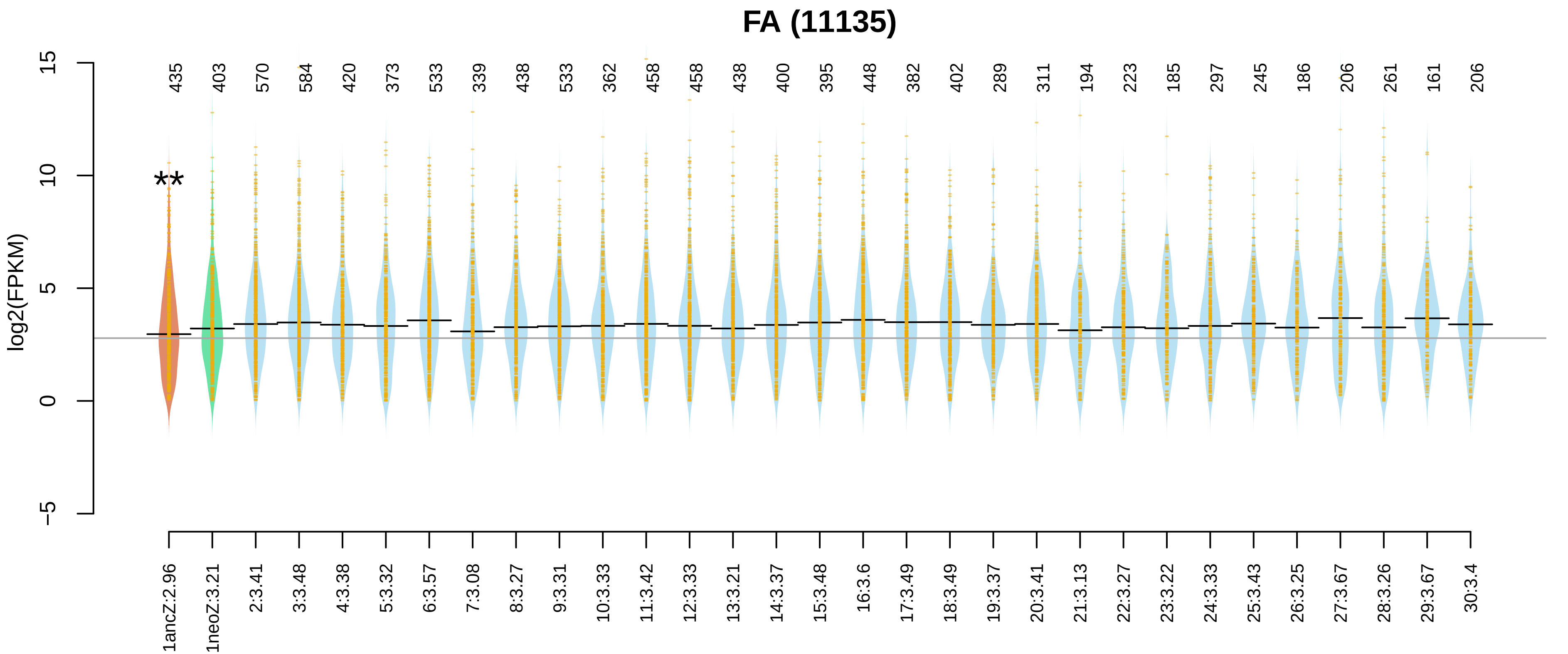

Wilcoxon test: are expression values on each chromosome different than the rest? Bonferroni−adjusted: ** p < 0.01, * p < 0.05

Supplementary Figure 22. Distribution of expression of all genes on each chromosome, according to tissue (FPKM > 1)

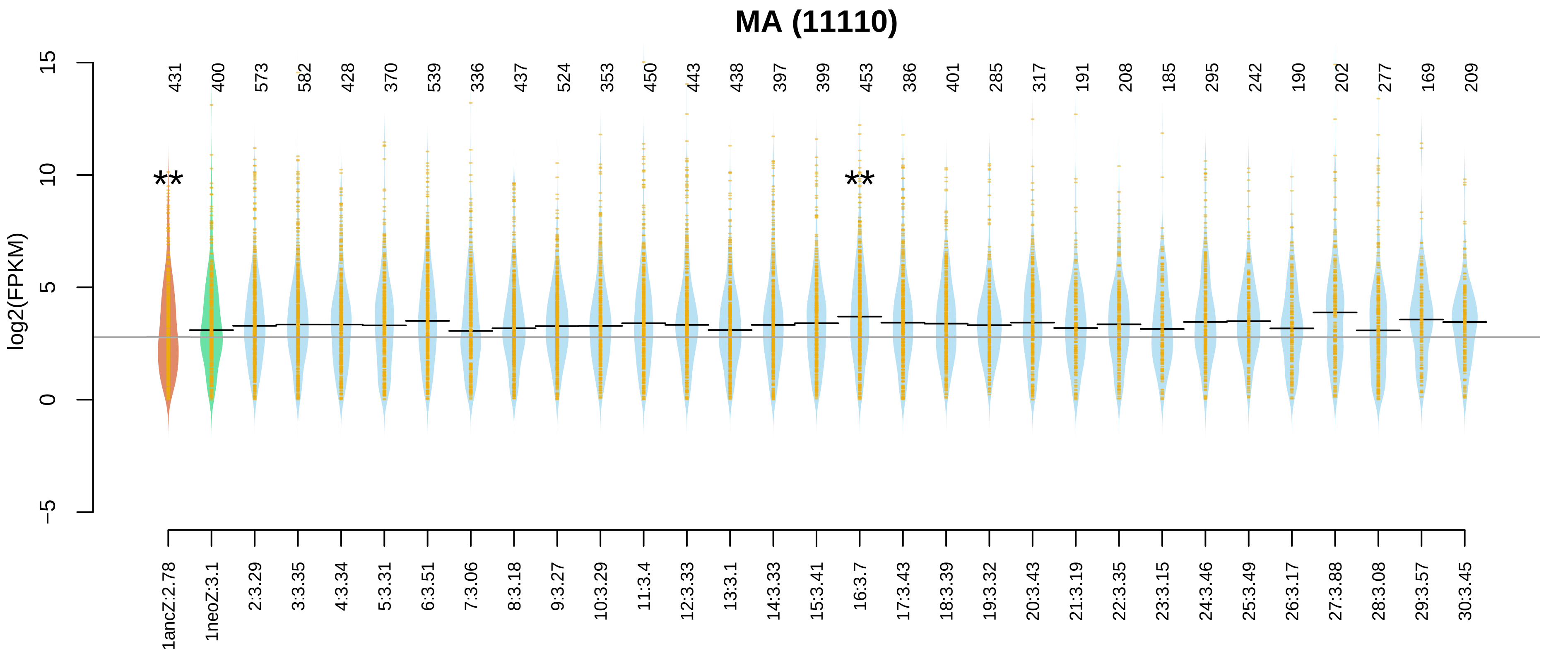

Wilcoxon test: are expression values on each chromosome different than the rest? Bonferroni−adjusted: ** p < 0.01, * p < 0.05

Supplementary Figure 22. Whole-chromosome expression in females (*ZW*) and males (*ZZ*) in four sample types of *D. plexippus* across four thresholds of minimum expression consider each chromosome separately. The four minimum expression thresholds considered are: 0.01 FPKM, 0.1 FPKM, 0.5 FPKM, and 1 FPKM. Sampled assayed: pools of whole-body males and females (Mpool, Fpool); individual samples of thorax (MT, FT), abdomen (MA, FA), and heads (MH, FH). The bean plots show the distribution of absolute normalized $\log_2$ expression values in FPKM for the ancestral (anc-) and neo (neo-) portions of chromosome *1* (*i.e.* the heterochromosome *Z*) and the autosomes. The horizontal line in each plot corresponds to the median expression value. A global median value across sample types is shown with a grey line in the background. The median *Z*:*A* ratios are shown at the bottom. For each sample type, statistical significance was established according to Wilcoxon signed-rank tests and upon applying the Bonferroni correction. The number of genes considered is indicated on top of each bean plot.

Supplementary Figure 23. Female/Male expression ratio on autosomes or sex chromosomes, according to tissue (FPKM > 0.01)

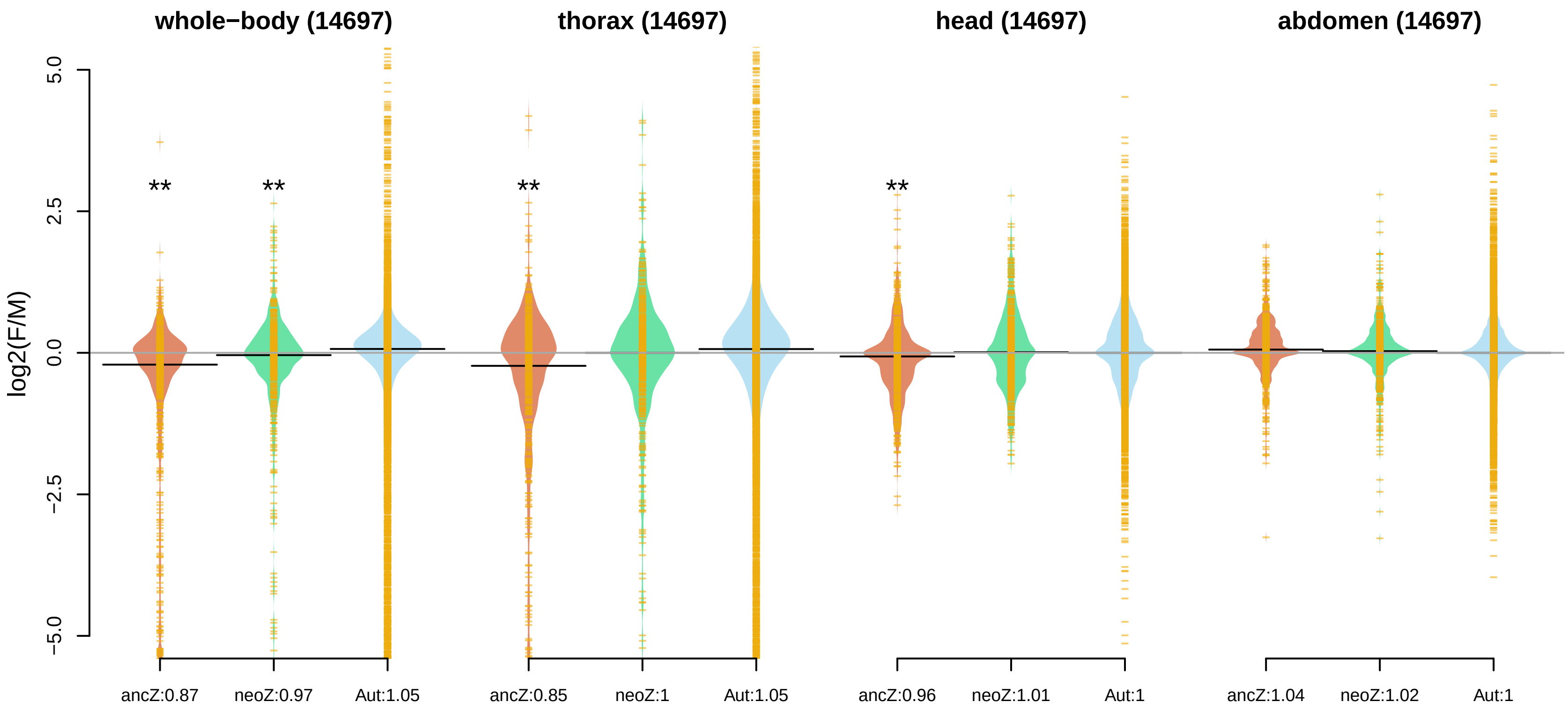

Wilcoxon test: are ratio values on sex−chromosome lower than autosomes? Bonferroni−adjusted: ** p < 0.01, * p < 0.05

Supplementary Figure 23. Female/Male expression ratio on autosomes or sex chromosomes, according to tissue (FPKM > 0.1)

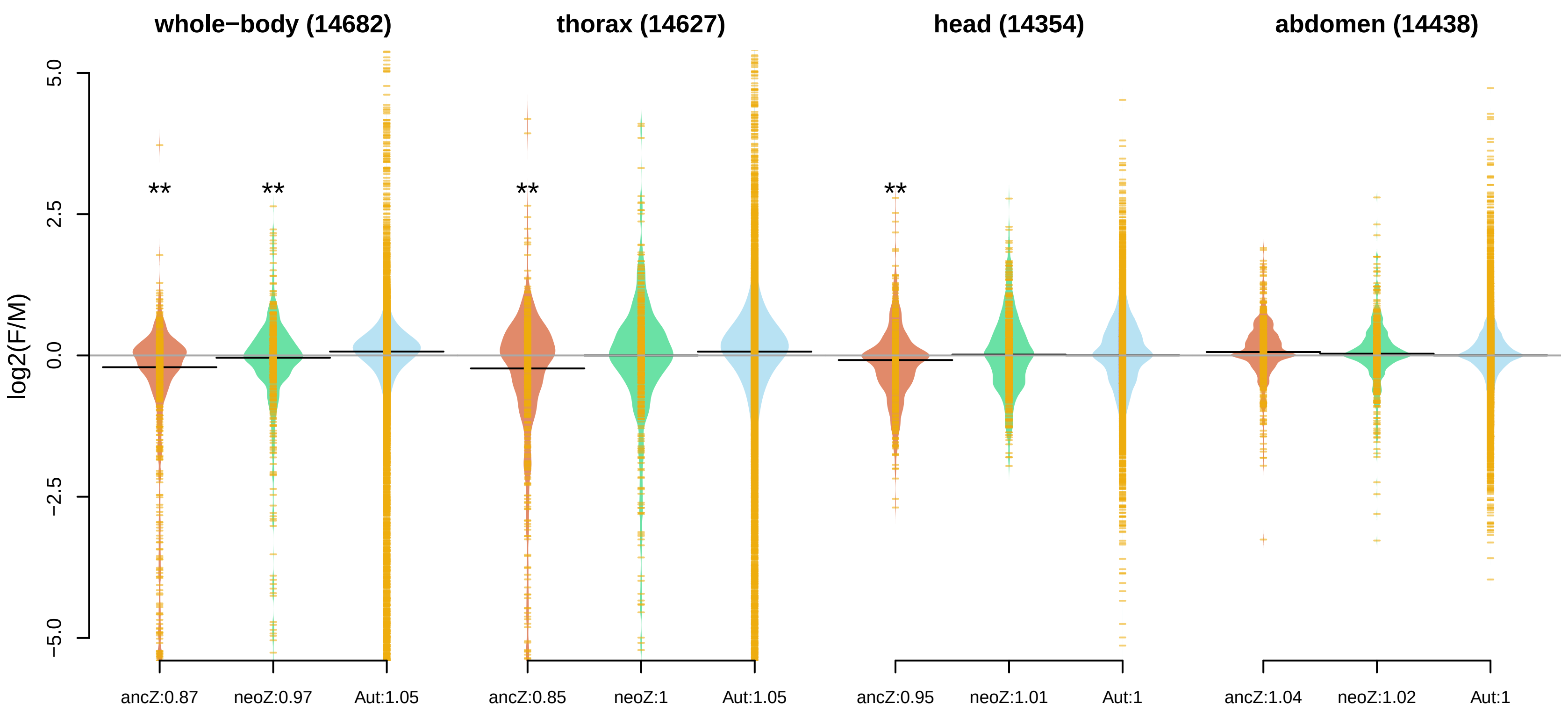

Wilcoxon test: are ratio values on sex−chromosome lower than autosomes? Bonferroni−adjusted: ** $p < 0.01$, * $p < 0.05$

Supplementary Figure 23. Female/Male expression ratio on autosomes or sex chromosomes, according to tissue (FPKM > 0.5)

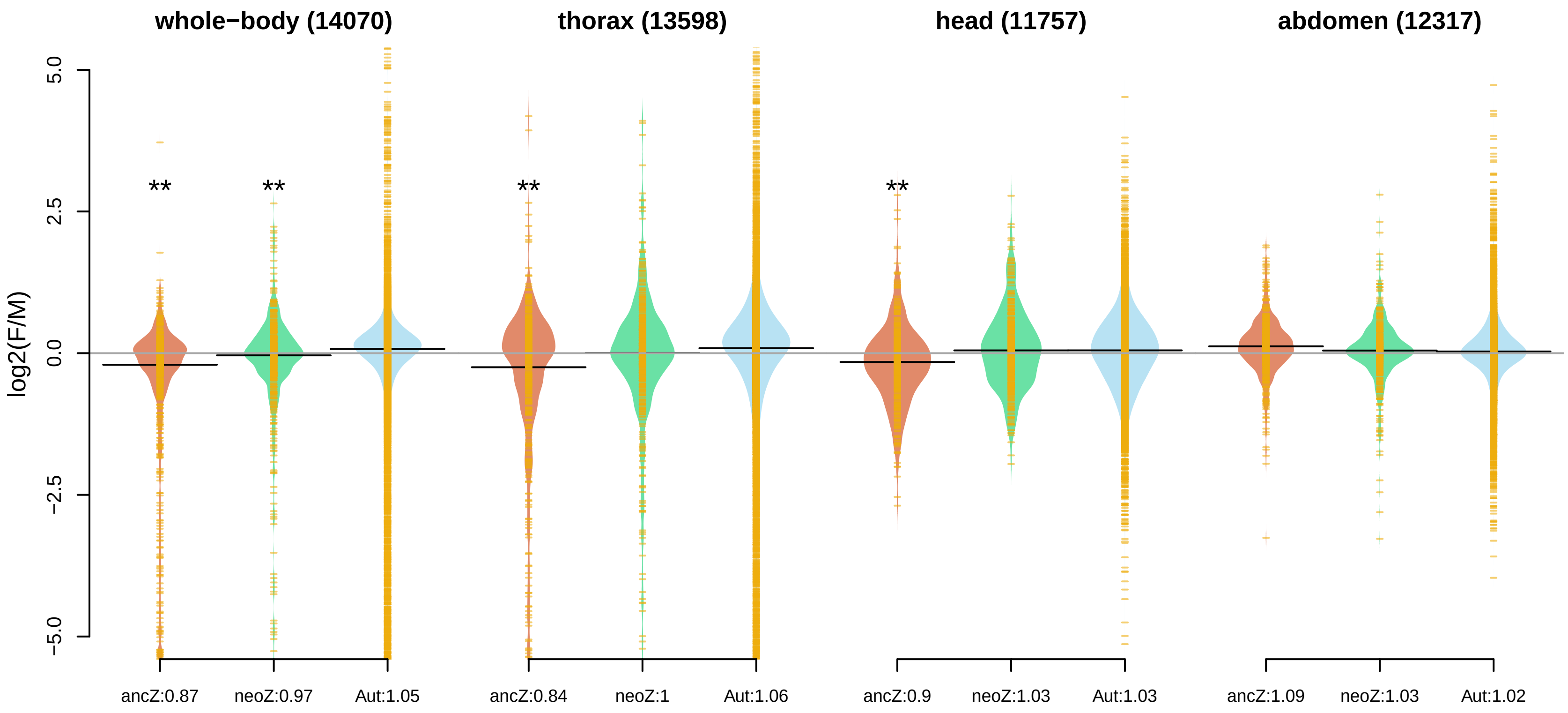

Wilcoxon test: are ratio values on sex−chromosome lower than autosomes? Bonferroni−adjusted: ** p < 0.01, * p < 0.05

Supplementary Figure 23. Female/Male expression ratio on autosomes or sex chromosomes, according to tissue (FPKM > 1)

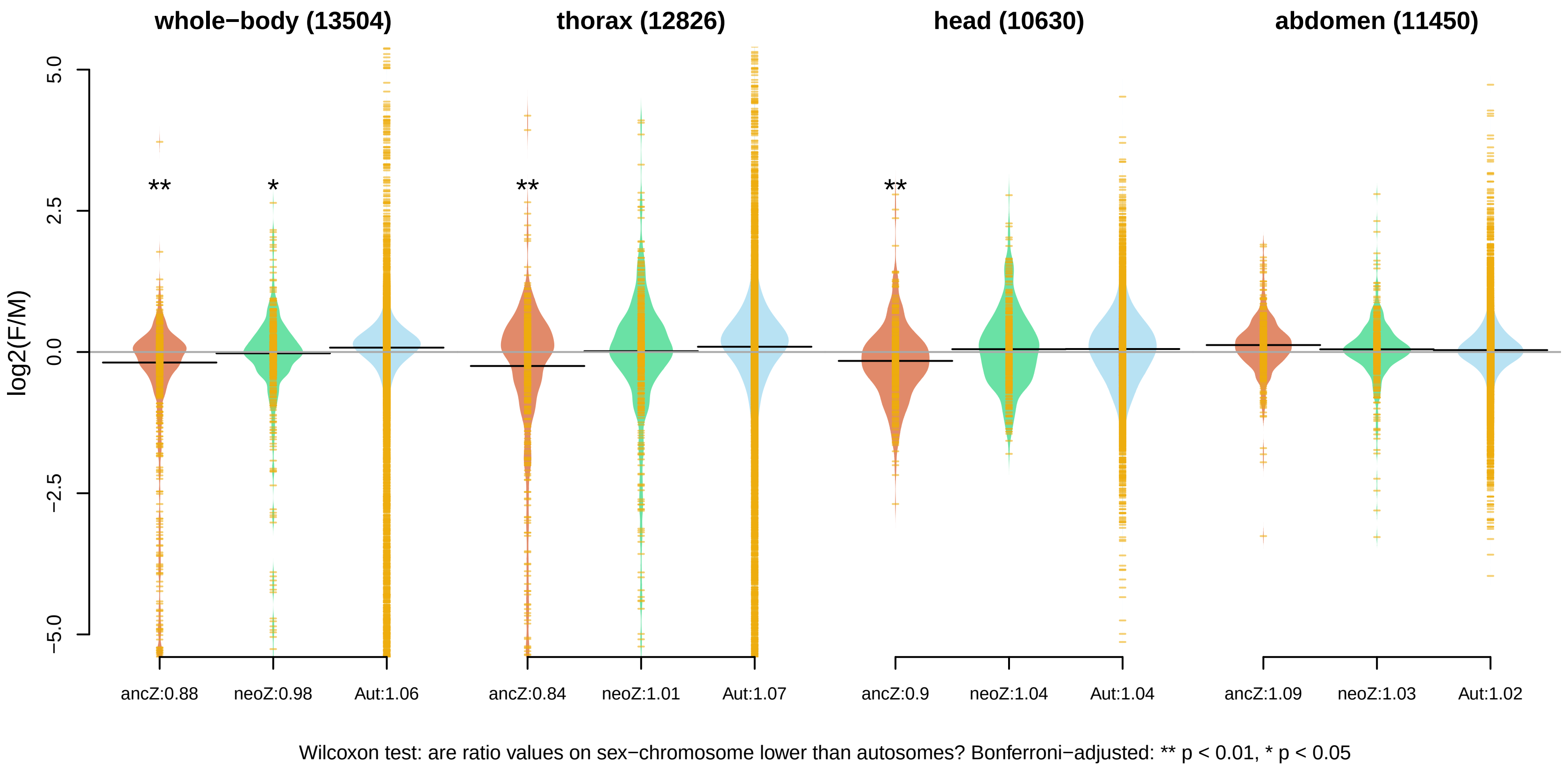

Supplementary Figure 23. Whole-chromosome median female (*ZW*) to male (*ZZ*) ratio across four sample types of *D. plexippus* across four thresholds of minimum expression. The four minimum expression thresholds considered are: 0.01 FPKM, 0.1 FPKM, 0.5 FPKM, and 1 FPKM**.**. Sampled assayed: pools of whole-body males and females; individual samples of thorax (MT, FT), abdomen (MA, FA), and heads (MH, FH). The bean plots show the distribution of absolute normalized $\log_2$ female to male expression ratios in FPKM for the ancestral (anc-) and neo (neo-) portions of chromosome *1* (*i.e.* the heterochromosome *Z*) and the autosomes. The horizontal line in each plot corresponds to the median of the expression ratios. A global median ratio across sample types (log2 F/M = 0, *i.e.* F/M=1) is shown with a grey line in the background. The median female to male expression ratios are shown at the bottom. For each sample type, statistical significance was established according to Wilcoxon signed-rank tests and upon applying the Bonferroni correction (**, P<0.01). The number of genes considered is indicated on top of each bean plot.

Supplementary Figure 24. Female/Male expression ratio all chromosomes, for one tissue (FPKM > 0.01)

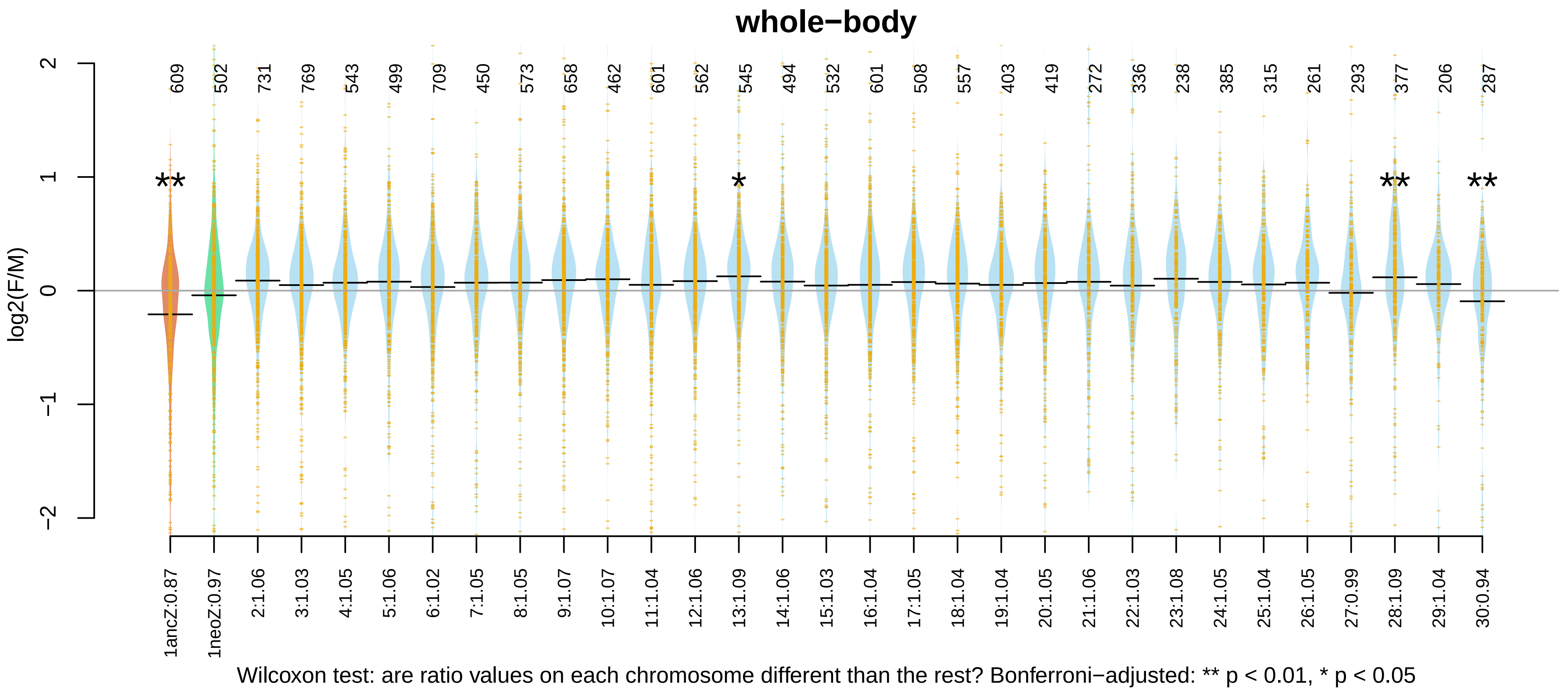

Supplementary Figure 24. Female/Male expression ratio all chromosomes, for one tissue (FPKM > 0.01)

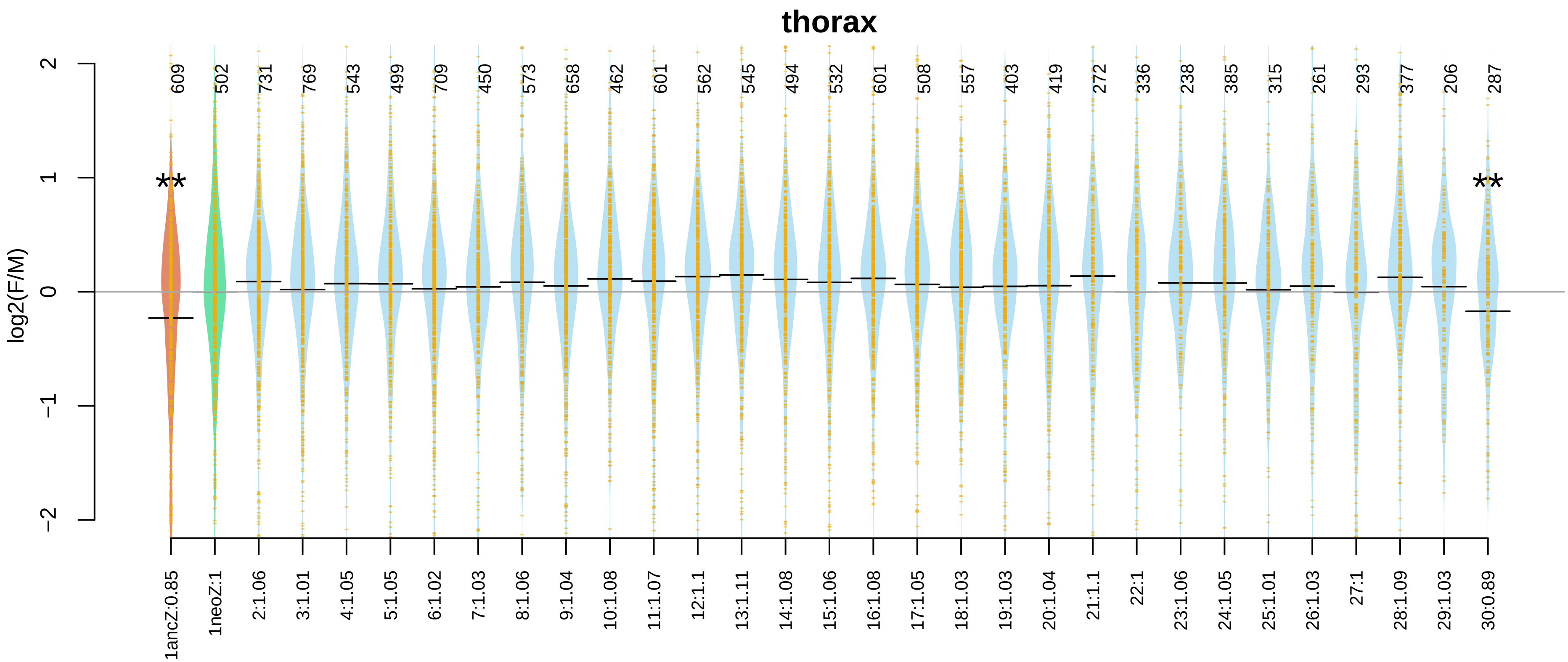

Wilcoxon test: are ratio values on each chromosome different than the rest? Bonferroni−adjusted: ** p < 0.01, * p < 0.05

Supplementary Figure 24. Female/Male expression ratio all chromosomes, for one tissue (FPKM > 0.01)

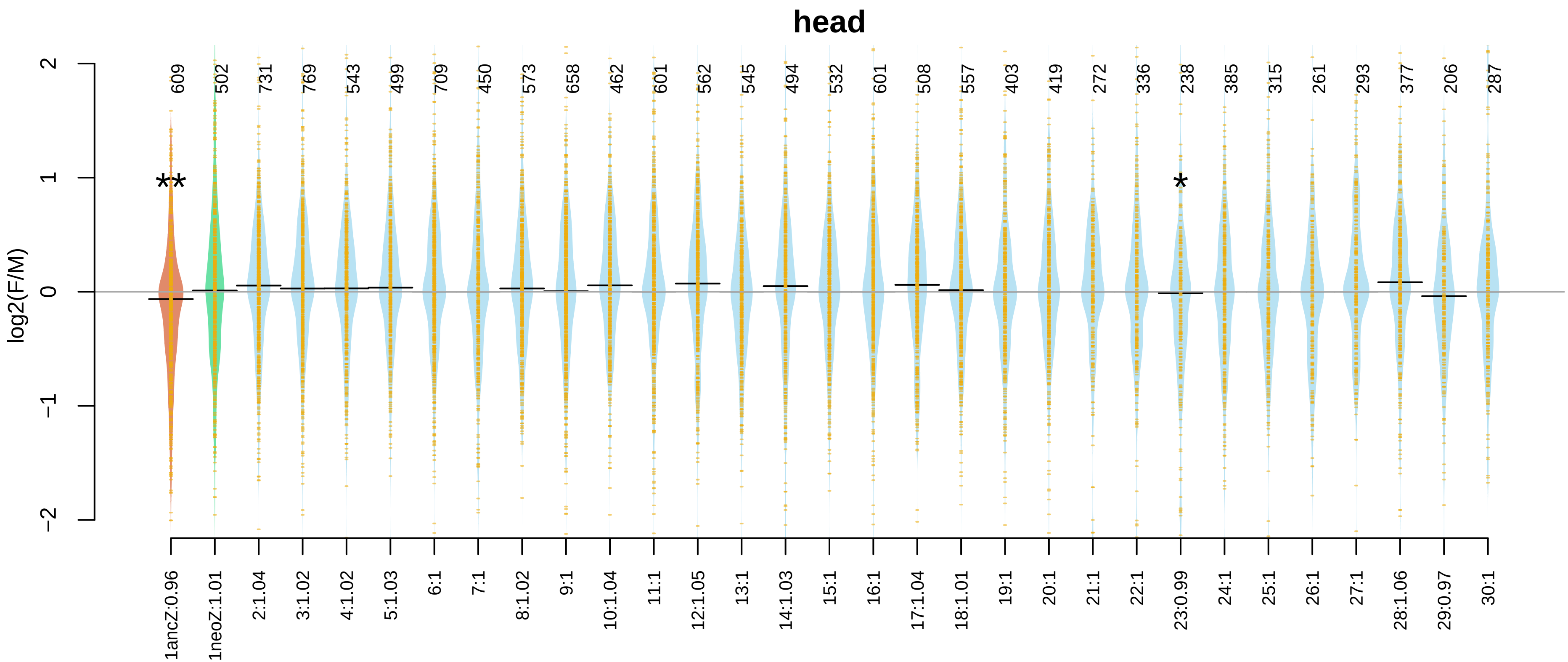

Wilcoxon test: are ratio values on each chromosome different than the rest? Bonferroni−adjusted: ** p < 0.01, * p < 0.05

Supplementary Figure 24. Female/Male expression ratio all chromosomes, for one tissue (FPKM > 0.01)

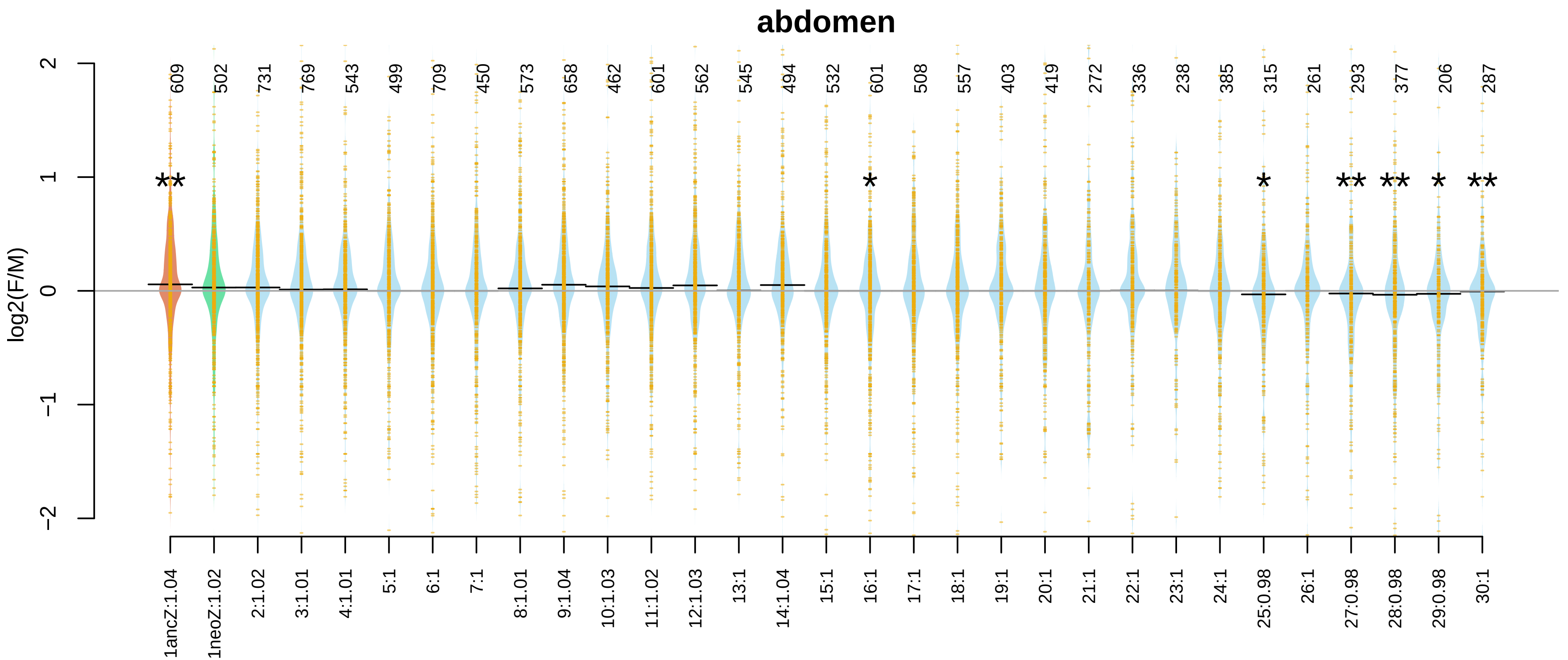

Wilcoxon test: are ratio values on each chromosome different than the rest? Bonferroni−adjusted: ** $p < 0.01$, * $p < 0.05$

Supplementary Figure 24. Female/Male expression ratio all chromosomes, for one tissue (FPKM > 0.1)

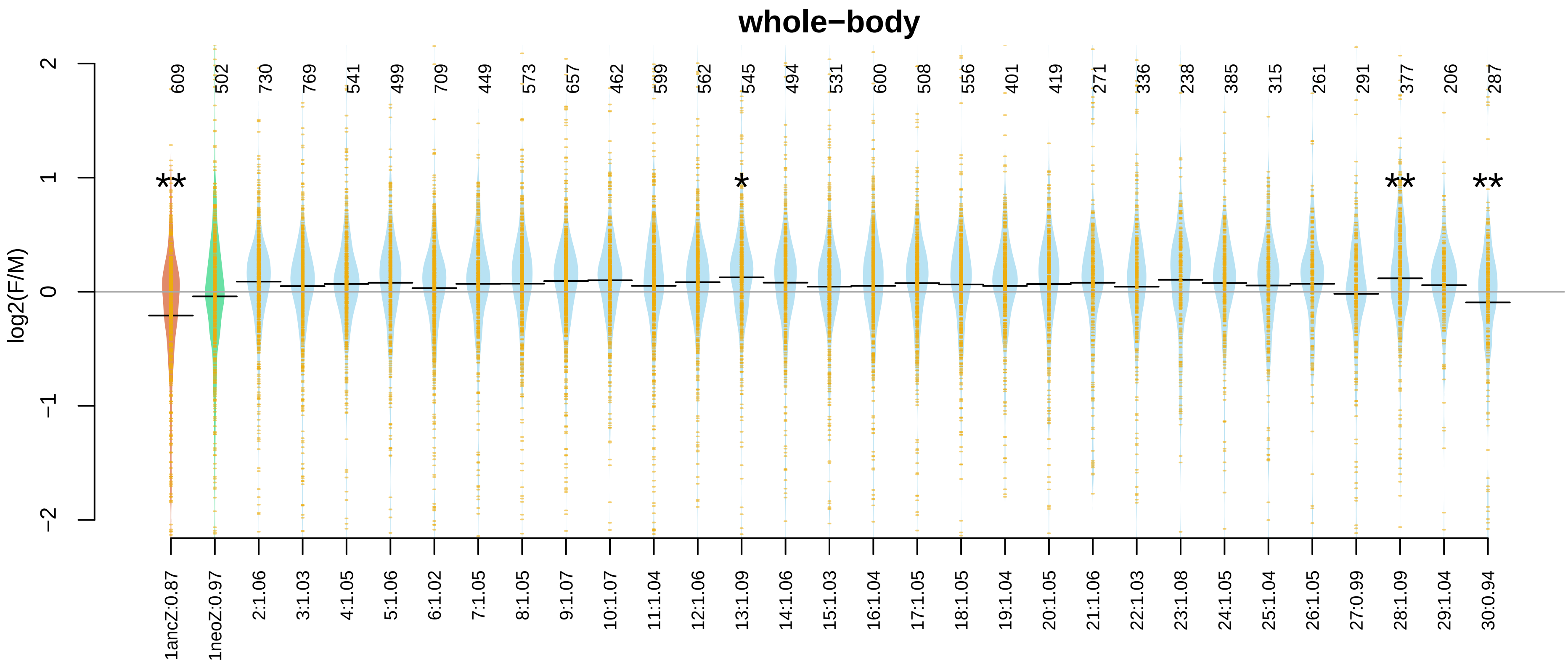

Wilcoxon test: are ratio values on each chromosome different than the rest? Bonferroni−adjusted: ** $p < 0.01$, * $p < 0.05$

Supplementary Figure 24. Female/Male expression ratio all chromosomes, for one tissue (FPKM > 0.1)

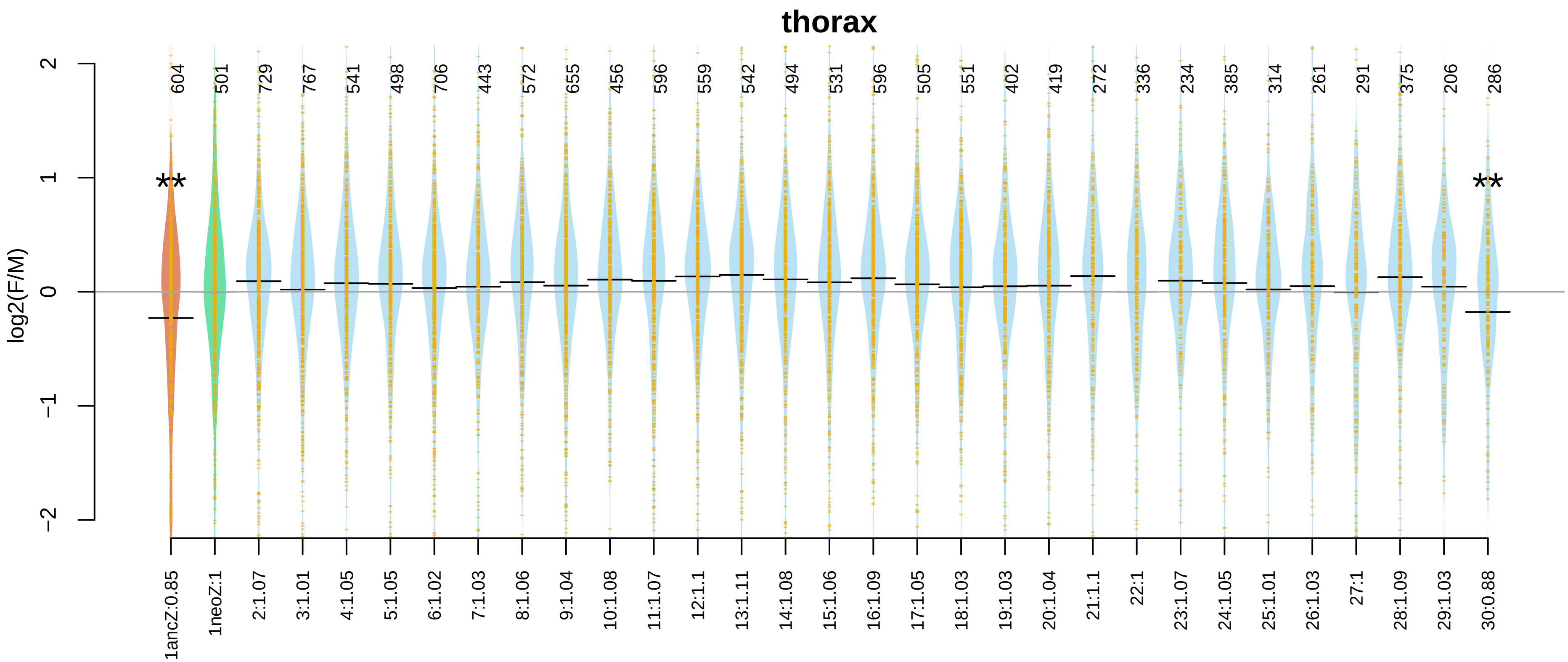

Wilcoxon test: are ratio values on each chromosome different than the rest? Bonferroni−adjusted: ** p < 0.01, * p < 0.05

Supplementary Figure 24. Female/Male expression ratio all chromosomes, for one tissue (FPKM > 0.1)

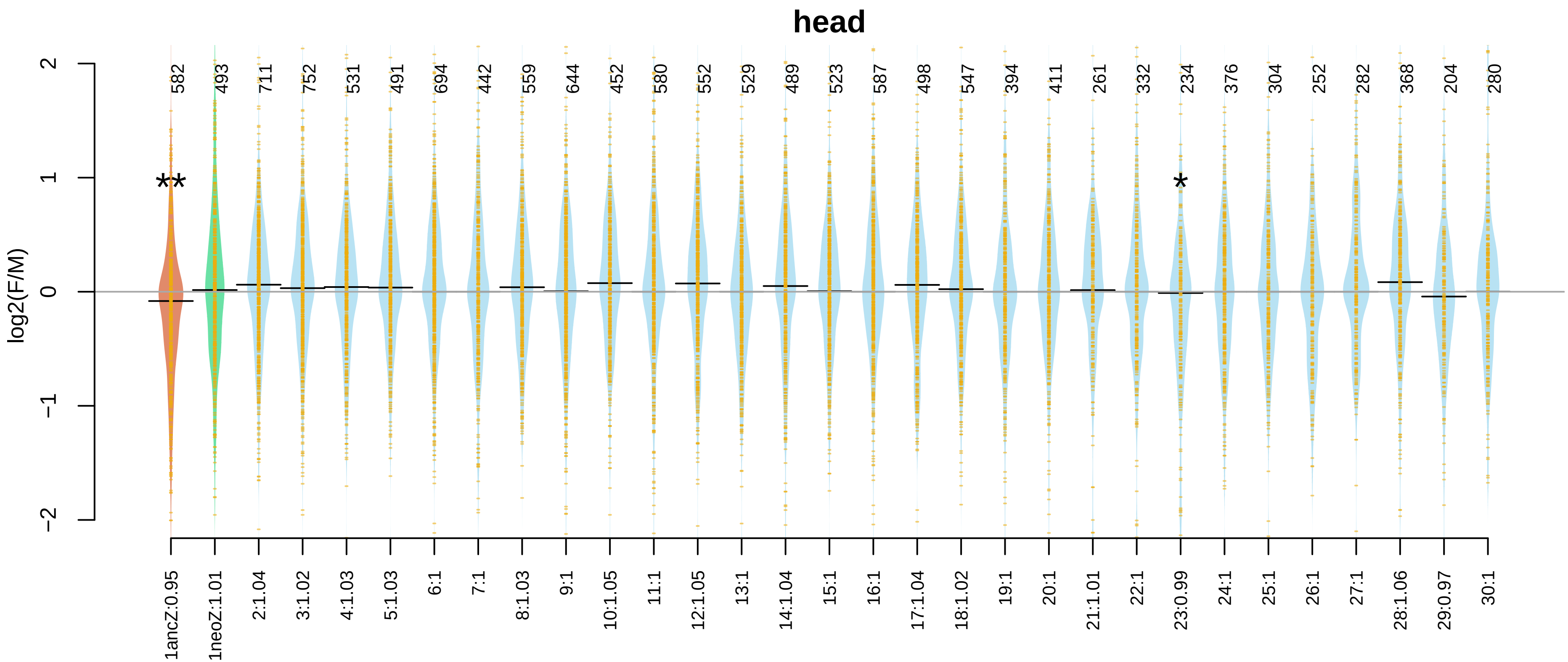

Wilcoxon test: are ratio values on each chromosome different than the rest? Bonferroni−adjusted: ** p < 0.01, * p < 0.05

Supplementary Figure 24. Female/Male expression ratio all chromosomes, for one tissue (FPKM > 0.1)

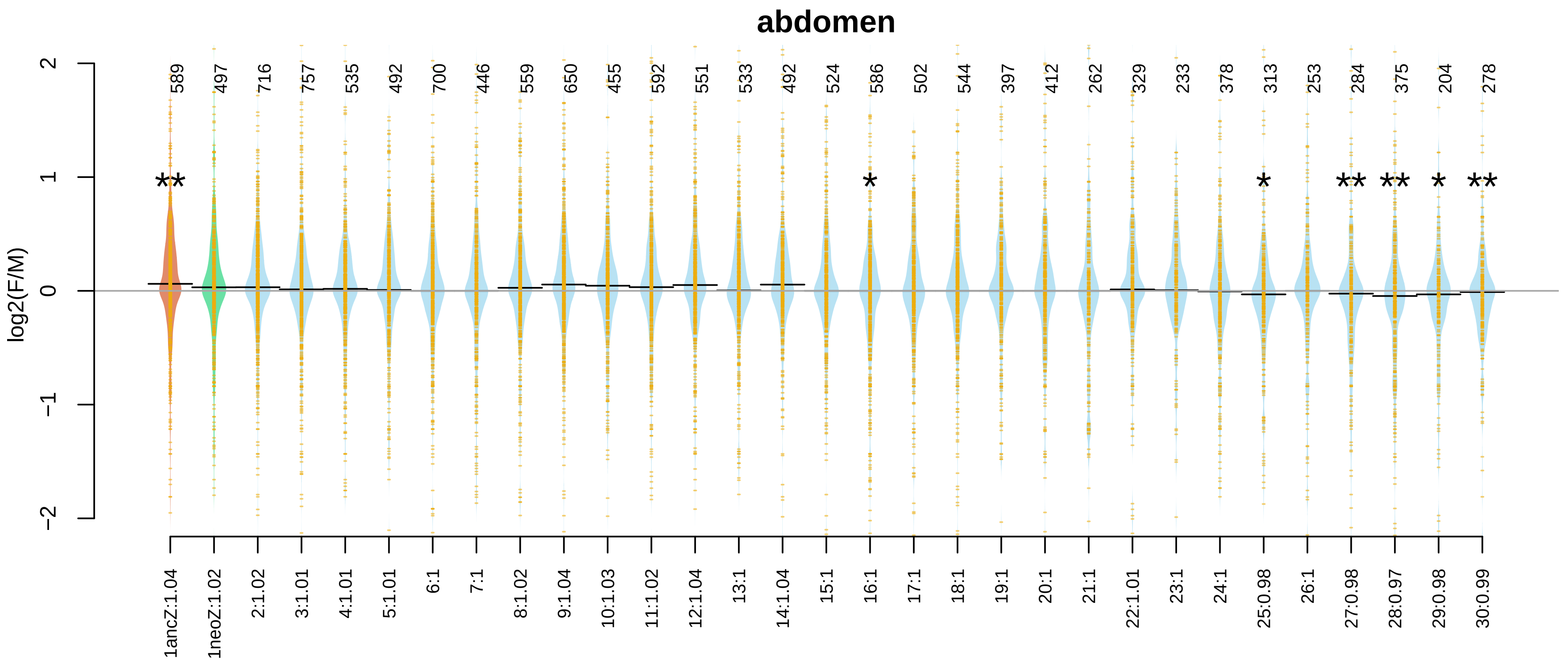

Wilcoxon test: are ratio values on each chromosome different than the rest? Bonferroni−adjusted: ** $p < 0.01$, * $p < 0.05$

Supplementary Figure 24. Female/Male expression ratio all chromosomes, for one tissue (FPKM > 0.5)

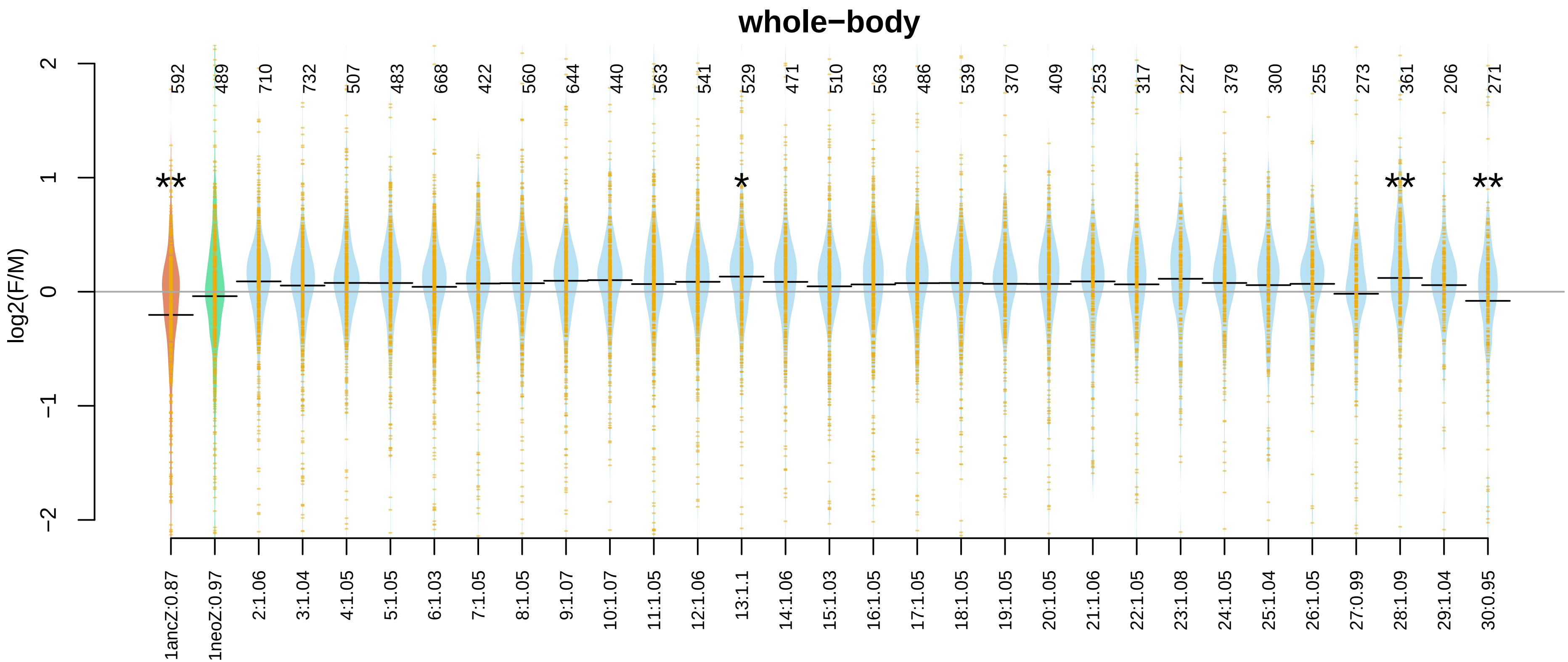

Wilcoxon test: are ratio values on each chromosome different than the rest? Bonferroni−adjusted: ** $p < 0.01$, * $p < 0.05$

Supplementary Figure 24. Female/Male expression ratio all chromosomes, for one tissue (FPKM > 0.5)

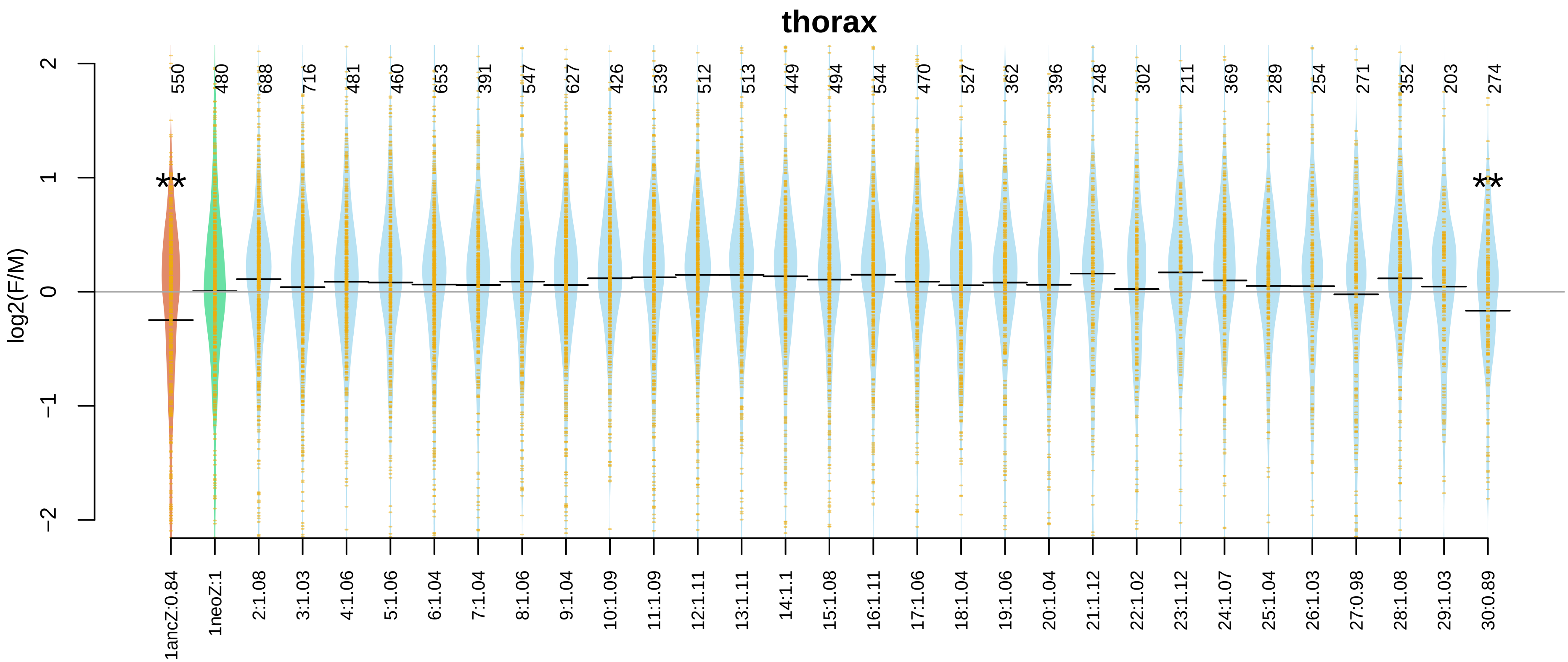

Wilcoxon test: are ratio values on each chromosome different than the rest? Bonferroni−adjusted: ** p < 0.01, * p < 0.05

Supplementary Figure 24. Female/Male expression ratio all chromosomes, for one tissue (FPKM > 0.5)

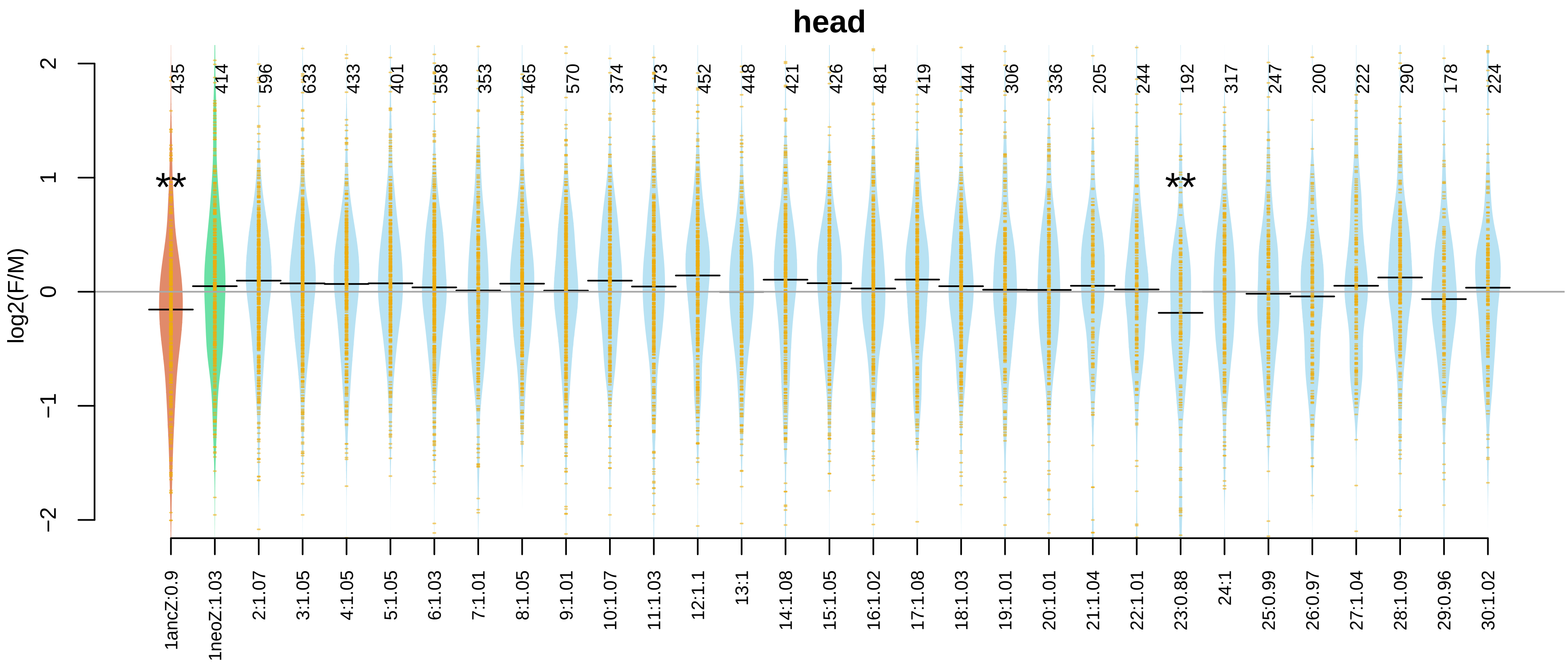

Wilcoxon test: are ratio values on each chromosome different than the rest? Bonferroni−adjusted: ** p < 0.01, * p < 0.05

Supplementary Figure 24. Female/Male expression ratio all chromosomes, for one tissue (FPKM > 0.5)

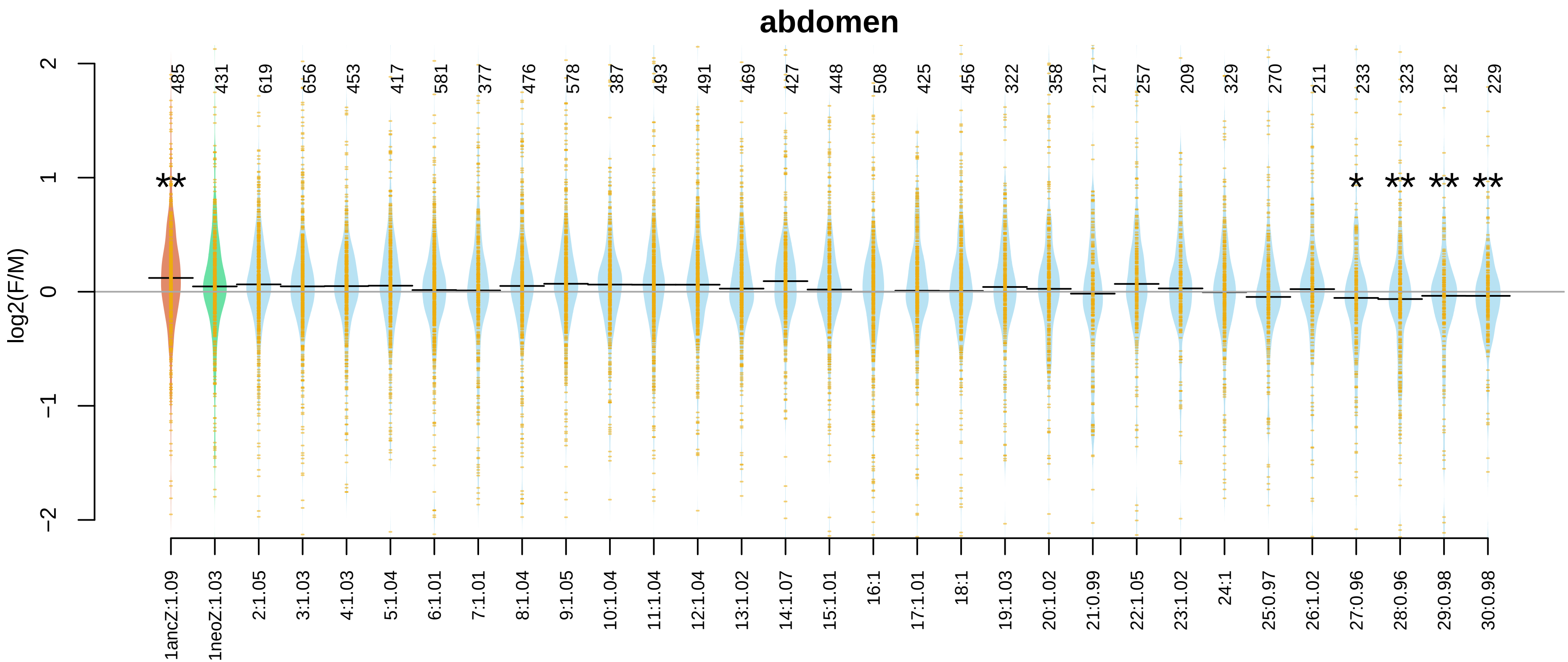

Wilcoxon test: are ratio values on each chromosome different than the rest? Bonferroni−adjusted: ** p < 0.01, * p < 0.05

Supplementary Figure 24. Female/Male expression ratio all chromosomes, for one tissue (FPKM > 1)

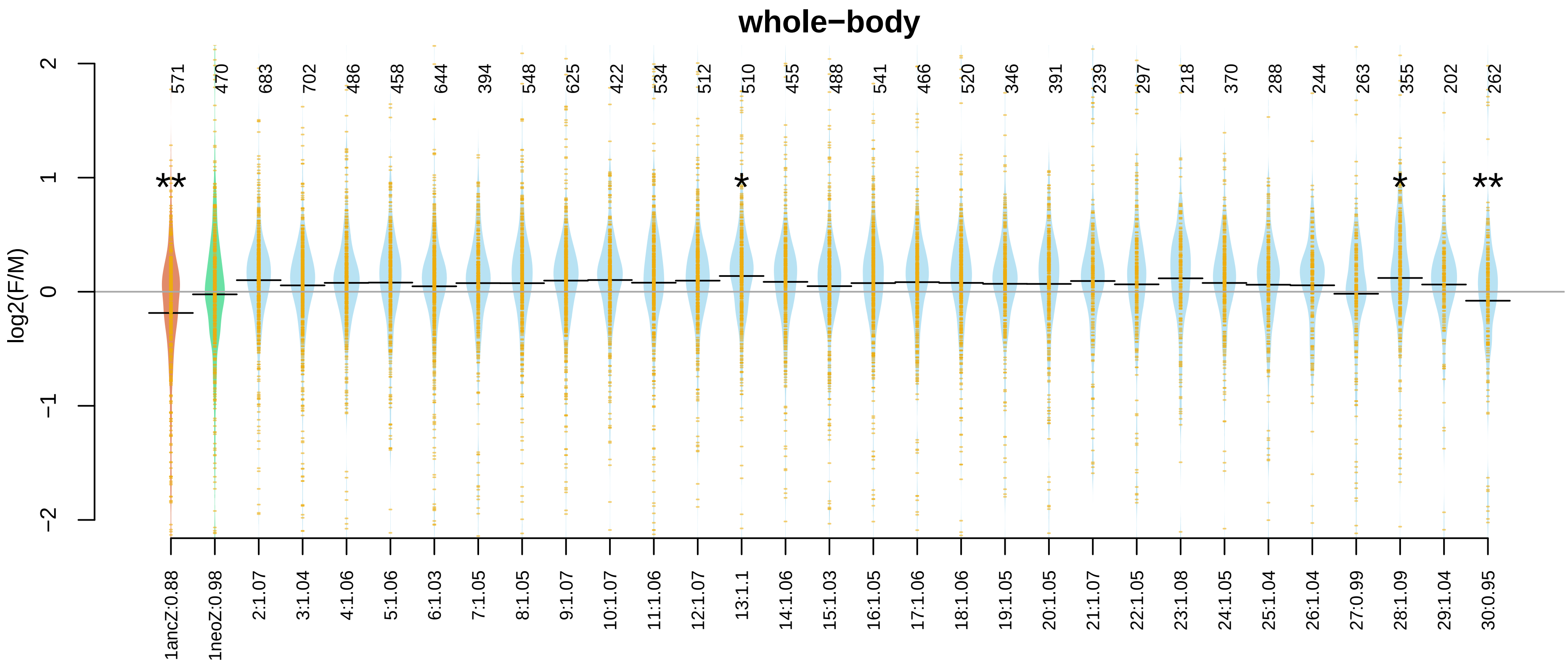

Wilcoxon test: are ratio values on each chromosome different than the rest? Bonferroni−adjusted: ** p < 0.01, * p < 0.05

Supplementary Figure 24. Female/Male expression ratio all chromosomes, for one tissue (FPKM > 1)

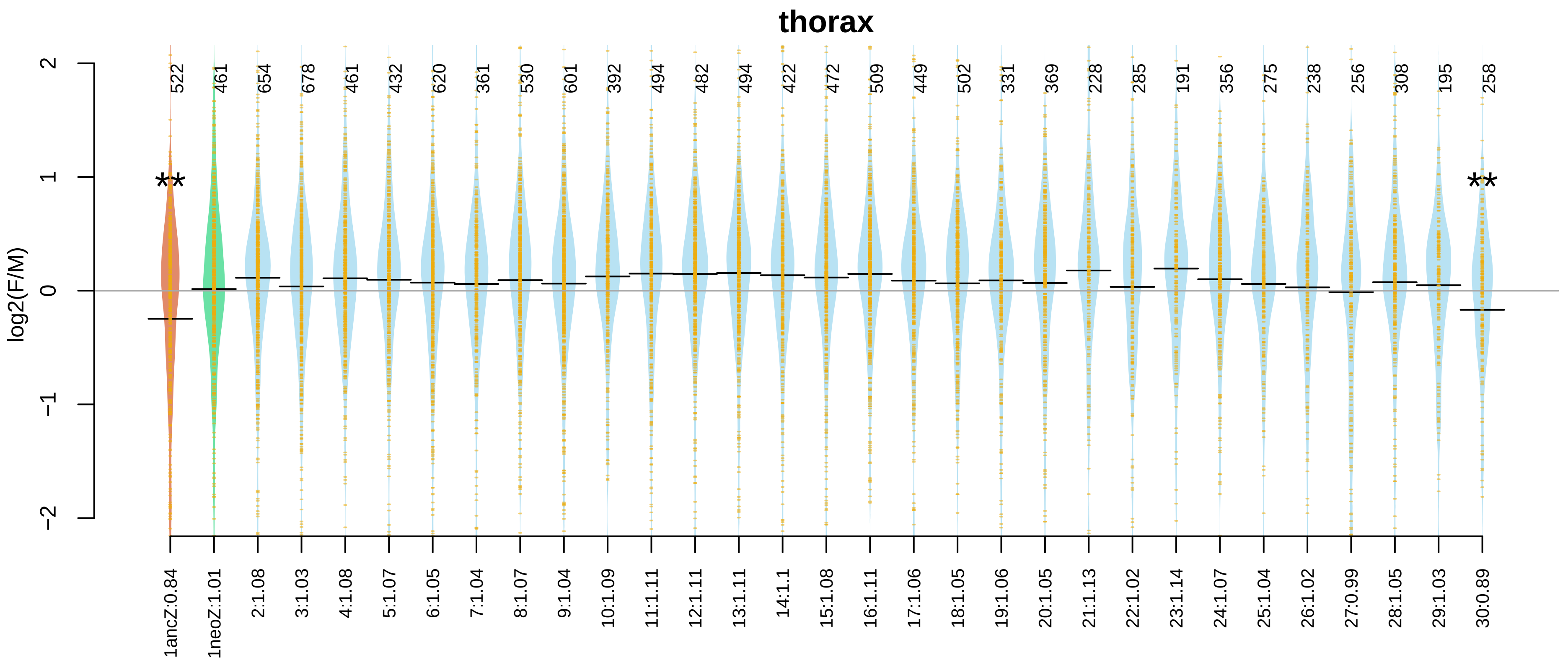

Wilcoxon test: are ratio values on each chromosome different than the rest? Bonferroni−adjusted: ** p < 0.01, * p < 0.05

Supplementary Figure 24. Female/Male expression ratio all chromosomes, for one tissue (FPKM > 1)

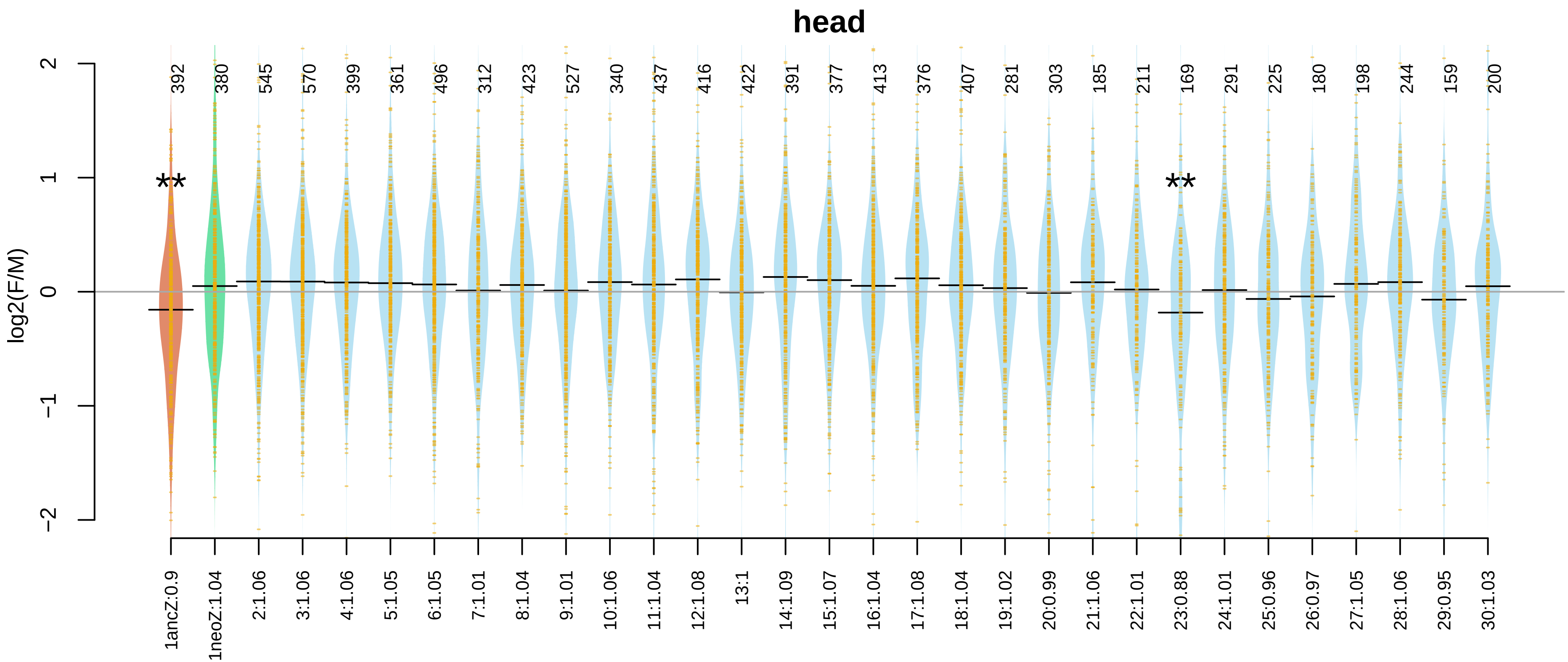

Wilcoxon test: are ratio values on each chromosome different than the rest? Bonferroni−adjusted: ** $p < 0.01$, * $p < 0.05$

Supplementary Figure 24. Female/Male expression ratio all chromosomes, for one tissue (FPKM > 1)

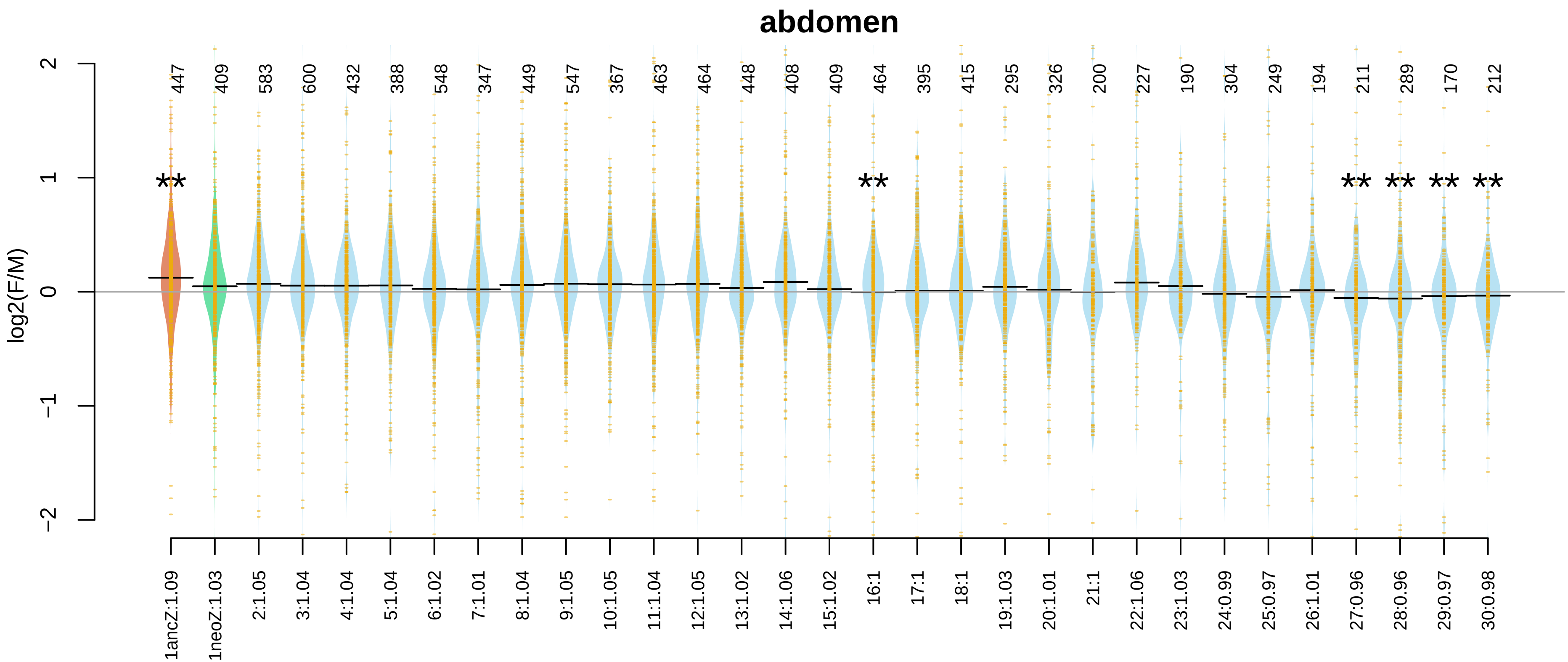

Wilcoxon test: are ratio values on each chromosome different than the rest? Bonferroni−adjusted: ** p < 0.01, * p < 0.05

Supplementary Figure 24. Whole-chromosome median female (ZW) to male (ZZ) ratio across four sample types of *D. plexippus* across four thresholds of minimum expression consider each chromosome separately. Sampled assayed: pools of whole-body males and females; individual samples of thorax (MT, FT), abdomen (MA, FA), and heads (MH, FH). The bean plots show the distribution of absolute normalized $\log_2$ female to male expression ratios in FPKM for the ancestral (anc-) and neo (neo-) portions of chromosome *1* (*i.e.* the heterochromosome *Z*) and the autosomes. The horizontal line in each plot corresponds to the median of the expression ratios. A global median ratio across sample types (log2 F/M = 0, *i.e.* F/M=1) is shown with a dotted line in the background. The median female to male expression ratios are shown at the bottom. For each sample type, statistical significance was established according to Wilcoxon signed-rank tests and upon applying the Bonferroni correction. The number of genes considered is indicated on top of each bean plot.

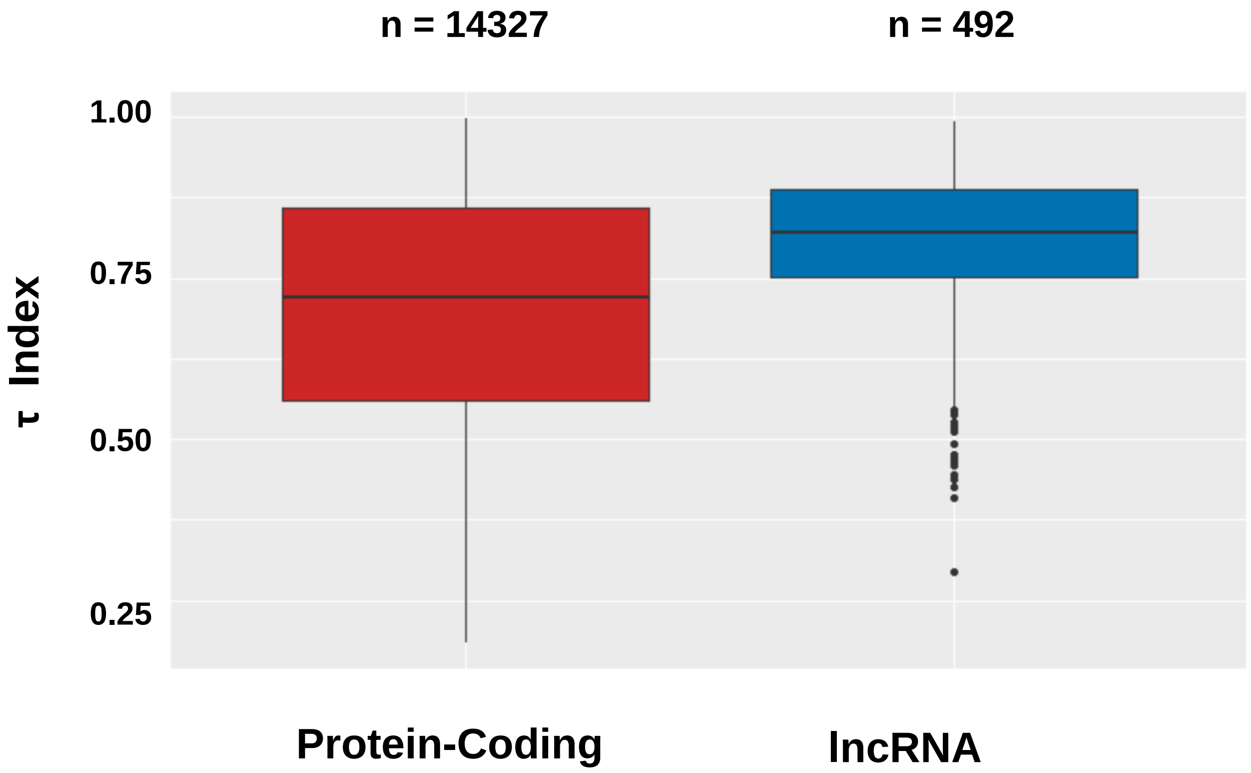

Supplementary Figure 25. Breadth of expression of protein-coding and lncRNA genes across the samples of *D. plexippus* assayed. The box plots show the distribution of the tau index values for both types of genes. Boxes represent the interquartile range (IQR) around the median (horizontal black line) and whiskers extend to 1.5 times the IQR; outlier data points beyond the 1.5 IQR are shown as solid dots. The tau index was calculated considering the 36 RNA-sequenced samples and using $\log_2$CPM values. An index value close to 1 indicates restricted expression while a value close to 0 indicates widespread expression. Top, number of genes considered. lncRNAs exhibit more restricted expression profiles compared to protein-coding genes, a conclusion that holds when the comparison is done separately per stages omitting or not non-expressed genes. (*i.e.* larva, pupa, and adulthood; not shown), and when is done separately per stages plus grouping genes per quartile based on expression levels (not shown).

SUPPLEMENTARY REFERENCES

1. Laetsch, D.R. & Blaxter, M.L. BlobTools: Interrogation of genome assemblies. *F1000Res* **6**, 1287 (2017).

2. Altschul, S.F. *et al.* Gapped BLAST and PSI-BLAST: a new generation of protein database search programs. *Nucleic Acids Res* **25**, 3389-402 (1997).

3. Nowell, R.W. *et al.* A high-coverage draft genome of the mycalesine butterfly Bicyclus anynana. *Gigascience* **6**, 1-7 (2017).

4. Koutsovoulos, G. *et al.* No evidence for extensive horizontal gene transfer in the genome of the tardigrade Hypsibius dujardini. *Proc Natl Acad Sci U S A* **113**, 5053-8 (2016).

5. Bemm, F., Weiss, C.L., Schultz, J. & Forster, F. Genome of a tardigrade: Horizontal gene transfer or bacterial contamination? *Proc Natl Acad Sci U S A* **113**, E3054-6 (2016).

6. Zhan, S. & Reppert, S.M. MonarchBase: the monarch butterfly genome database. *Nucleic acids research* **41**, D758-63 (2013).

7. Gu, L. *et al.* Dichotomy of Dosage Compensation along the Neo Z Chromosome of the Monarch Butterfly. *Curr Biol* **29**, 4071-4077 e3 (2019).

8. Mongue, A.J., Nguyen, P., Volenikova, A. & Walters, J.R. Neo-sex Chromosomes in the Monarch Butterfly, Danaus plexippus. *G3 (Bethesda)* **7**, 3281-3294 (2017).

9. Pringle, E.G. *et al.* Synteny and chromosome evolution in the lepidoptera: evidence from mapping in Heliconius melpomene. *Genetics* **177**, 417-26 (2007).

10. Ahola, V. *et al.* The Glanville fritillary genome retains an ancient karyotype and reveals selective chromosomal fusions in Lepidoptera. *Nat Commun* **5**, 4737 (2014).

11. Davey, J.W. *et al.* Major Improvements to the Heliconius melpomene Genome Assembly Used to Confirm 10 Chromosome Fusion Events in 6 Million Years of Butterfly Evolution. *G3 (Bethesda)* **6**, 695-708 (2016).

12. Zhan, S. *et al.* The genetics of monarch butterfly migration and warning colouration. *Nature* **514**, 317-21 (2014).

13. Martin, S.H. *et al.* Genome-wide evidence for speciation with gene flow in Heliconius butterflies. *Genome Res* **23**, 1817-28 (2013).

14. Langfelder, P. & Horvath, S. WGCNA: an R package for weighted correlation network analysis. *BMC Bioinformatics* **9**, 559 (2008).

15. Simao, F.A., Waterhouse, R.M., Ioannidis, P., Kriventseva, E.V. & Zdobnov, E.M. BUSCO: assessing genome assembly and annotation completeness with single-copy orthologs. *Bioinformatics (Oxford, England)* **31**, 3210-2 (2015).

16. Bewick, V., Cheek, L. & Ball, J. Statistics review 8: Qualitative data - tests of association. *Crit Care* **8**, 46-53 (2004).
