# Supplementary material for: A de novo transcriptional atlas in Danaus plexippus reveals variability in dosage compensation across tissues
Source: Commun Biol. 2021 Jun 25;4:791. doi: 10.1038/s42003-021-02335-3 (PMC8233437; doi:10.1038/s42003-021-02335-3)
Supplement: Supplementary file 2 — Details of Supplementary Data files [file 42003_2021_2335_MOESM2_ESM.pdf]

Description of Additional Supplementary Files

File name: Supplementary Data 1

Description: Differential joinings detected between Dpv3 scaffolds and DpMex_v1 contigs using RaGOO.

File name: Supplementary Data 2

Description: Missing BUSCOs in DpMex_v1.

File name: Supplementary Data 3

Description: Salient features of the constituent contigs of the genome assembly DpMex_v1 of *D. plexippus*.

File name: Supplementary Data 4

Description: Coverage and mapping statistics for 80 *D. plexippus* DNA-sequencing samples assessed in this study.

File name: Supplementary Data 5

Description: Salient features of the libraries sequenced to construct the transcriptome atlas of *D. plexippus*.

File name: Supplementary Data 6

Description: Per-gene expression levels across the sequenced libraries used to construct the transcriptome atlas.

File name: Supplementary Data 7

Description: Expression trends of *D. plexippus* genes.

File name: Supplementary Data 8

Description: Functional enrichment patterns in different sets of genes that show consistent expression patterns.
