# Supplementary material for: A de novo transcriptional atlas in Danaus plexippus reveals variability in dosage compensation across tissues
Source: Commun Biol. 2021 Jun 25;4:791. doi: 10.1038/s42003-021-02335-3 (PMC8233437; doi:10.1038/s42003-021-02335-3)
Supplement: Supplementary file 11 — Reporting Summary [file 42003_2021_2335_MOESM11_ESM.pdf]

nature research

Corresponding author(s): Jose Ranz and Cei Abreu-Goodger

Last updated by author(s): Jun 4, 2021

Reporting Summary

Nature Research wishes to improve the reproducibility of the work that we publish. This form provides structure for consistency and transparency in reporting. For further information on Nature Research policies, see our Editorial Policies and the Editorial Policy Checklist.

Statistics

For all statistical analyses, confirm that the following items are present in the figure legend, table legend, main text, or Methods section.

n/a	Confirmed	
☐	☒	The exact sample size (*n*) for each experimental group/condition, given as a discrete number and unit of measurement
☒	☐	A statement on whether measurements were taken from distinct samples or whether the same sample was measured repeatedly
☐	☒	The statistical test(s) used AND whether they are one- or two-sided<br>*Only common tests should be described solely by name; describe more complex techniques in the Methods section.*
☒	☐	A description of all covariates tested
☐	☒	A description of any assumptions or corrections, such as tests of normality and adjustment for multiple comparisons
☐	☒	A full description of the statistical parameters including central tendency (e.g. means) or other basic estimates (e.g. regression coefficient) AND variation (e.g. standard deviation) or associated estimates of uncertainty (e.g. confidence intervals)
☐	☒	For null hypothesis testing, the test statistic (e.g. *F*, *t*, *r*) with confidence intervals, effect sizes, degrees of freedom and *P* value noted<br>*Give P values as exact values whenever suitable.*
☒	☐	For Bayesian analysis, information on the choice of priors and Markov chain Monte Carlo settings
☐	☒	For hierarchical and complex designs, identification of the appropriate level for tests and full reporting of outcomes
☐	☒	Estimates of effect sizes (e.g. Cohen's *d*, Pearson's *r*), indicating how they were calculated

Our web collection on statistics for biologists contains articles on many of the points above.

Software and code

Policy information about availability of computer code

Data collection	Most data analyzed are original to this work. The Methods section describes how the genomic and RNA-seq data were obtained. In addition, we used several other data sets external to this manuscript and we indicate them in the manuscript. The latter include: a previously published genome assembly, a collection of DNA-seq data sets, and translated sequences from different insects -primarily Lepidoptera- (see Supplementary Table 7).
Data analysis	No novel algorithm was designed for this project. Instead, we used several publicly available tools and R/Bioconductor packages, which are all described and referenced in the corresponding Methods section. The R packages were called from within R scripts that we wrote, and Linux command line tools were used from within Bash scripts. Although they are not part of any formal pipeline, any of the used in-home scripts can be shared upon request; we include a specific statement in the section Statistics and Reproducibility.

For manuscripts utilizing custom algorithms or software that are central to the research but not yet described in published literature, software must be made available to editors and reviewers. We strongly encourage code deposition in a community repository (e.g. GitHub). See the Nature Research guidelines for submitting code & software for further information.

Data

Policy information about availability of data

All manuscripts must include a data availability statement. This statement should provide the following information, where applicable:

- Accession codes, unique identifiers, or web links for publicly available datasets
- A list of figures that have associated raw data
- A description of any restrictions on data availability

All raw sequencing data were deposited as part of the NCBI BioProject PRJNA663267. The Whole Genome Shotgun project has been deposited at DDBJ/ENA/GenBank under the accession JAEQBL000000000. The version described in this paper is version JAEQBL010000000. The annotation file for the sequenced assembly

is available at Zenodo

Field-specific reporting

Please select the one below that is the best fit for your research. If you are not sure, read the appropriate sections before making your selection.

- [x] Life sciences
- [ ] Behavioural & social sciences
- [ ] Ecological, evolutionary & environmental sciences

For a reference copy of the document with all sections, see nature.com/documents/nr-reporting-summary-flat.pdf

Life sciences study design

All studies must disclose on these points even when the disclosure is negative.

Sample size	The monarch is an endangered species. This fact, plus availability and costs, determined our sample size choices. Consequently, for RNA-seq, we used single individuals for all stages with the exception of 1st instar larvae, for which we used pools of six individuals. In addition, for genome sequencing, one individual was chosen to minimize the effects of heterozygosity.
Data exclusions	No data were excluded.
Replication	For the construction of our transcriptome atlas, we included 2 biological replicates for each developmental stage surveyed. Several analyses, e.g. our multidimensional scaling analysis, confirmed the reproducibility of our results.
Randomization	Individuals at a particular developmental stage were chosen randomly among those available.
Blinding	Blinding was not relevant for our study.

Reporting for specific materials, systems and methods

We require information from authors about some types of materials, experimental systems and methods used in many studies. Here, indicate whether each material, system or method listed is relevant to your study. If you are not sure if a list item applies to your research, read the appropriate section before selecting a response.

Materials & experimental systems

n/a	Involved in the study	
x		Antibodies
x		Eukaryotic cell lines
x		Palaeontology and archaeology
	x	Animals and other organisms
x		Human research participants
x		Clinical data
x		Dual use research of concern

Methods

n/a	Involved in the study	
x		ChIP-seq
x		Flow cytometry
x		MRI-based neuroimaging

Animals and other organisms

Policy information about studies involving animals; ARRIVE guidelines recommended for reporting animal research

Laboratory animals	Not applicable
Wild animals	Not applicable
Field-collected samples	Newly hatched larvae of D. plexippus were collected from wild Asclepias curassavica on the campus of the National Laboratory for Genomics of Biodiversity in Irapuato, Guanajuato, México. Collection was made under collection permit OFICIO NÚM. SGPA/DGVS/02879/15, which was issued by the Secretaría de Medio Ambiente y Recursos Naturales (SEMARNAT), Subsecretaría de Gestión para la Protección Ambiental, Dirección General de Vida Silvestre, México. Early first instar caterpillars were placed in individual vials and fed with fresh A. curassavica leaves ad libitum on a 12-12h light-dark cycle at 25°C and ~50% relative humidity until adults emerged. All stages were precisely identified measuring head capsules left after molting.
Ethics oversight	No ethical approval was required for this species.

Note that full information on the approval of the study protocol must also be provided in the manuscript.
